# Relationship between gut microbiota and thyroid function: a two-sample Mendelian randomization study

**DOI:** 10.3389/fendo.2023.1240752

**Published:** 2023-09-26

**Authors:** Liangzhuo Xie, Huaye Zhao, Wei Chen

**Affiliations:** ^1^ Graduate School, Liaoning University of Traditional Chinese Medicine, Shenyang, China; ^2^ School of Tranditional Chinese, Liaoning University of Traditional Chinese Medicine, Shenyang, China; ^3^ Department of Geriatrics, The Second Affiliated Hospital of Liaoning University of Traditional Chinese Medicine, Shenyang, China

**Keywords:** gut microbiota, thyroid function, causal effect, hypothyroidism, hyperthyroidism, Mendelian randomization

## Abstract

**Background:**

Numerous observational studies have indicated a link between the composition of gut microbiota and thyroid function. Nevertheless, the precise causal relationship between gut microbiota and thyroid function remains uncertain.

**Methods:**

In this two-sample Mendelian randomization study, we utilized summary data from a genome-wide association study of gut microbiota composition in 18,340 participants from 24 cohorts, as well as summary statistics on thyroid hormones and thyroid-stimulating hormone from the ThyroidOmics Consortium and summary statistics on hypothyroidism and hyperthyroidism from the FinnGen R8 release. Five different methods, including inverse variance weighting, MR-Egger, weighted median, weighted mode, and simple mode, were employed to examine the causal relationship between gut microbiota and thyroid function. Reverse Mendelian randomization analysis was conducted for taxa identified as having a causal relationship with thyroid function in the Mendelian randomization analysis. To assess the robustness of the results, sensitivity analyses were conducted employing Cochran’s Q test, MR-Egger intercept test, MR-PRESSO global test, and leave-one-out analysis.

**Results:**

Through MR analysis of 211 microbial taxa and 4 phenotypes, we identified a total of 34 gut microbiota taxa that were associated with the outcomes. After using the bonferroni method for multiple testing correction, phylum *Actinobacteria* (id.400) had a protective effect on hypothyroidism (OR=0.883, 95% CI: 0.817-0.955, *P*=0.002), and class *Deltaproteobacteria* (id.3087) had a protective effect on hyperthyroidism (OR=0.549, 95% CI: 0.374-0.805, *P*=0.002). According to the results of reverse MR analysis, no significant causal effect of the four phenotypes was found on gut microbiota. No significant horizontal pleiotropy was detected based on MR-Egger intercept test and MR-PRESSO global test.

**Conclusion:**

Through two-sample MR analysis, we identified specific gut microbiota taxa at the genetic level that are predicted to have a causal relationship with thyroid function, which may serve as useful biomarkers for early disease diagnosis.

## 1 Introduction

The thyroid gland is the largest endocrine gland in the human body, and its secreted thyroid hormones(TH) play a crucial role in growth, development, and metabolism (1). Thyroid-stimulating hormone (TSH) is secreted by the anterior pituitary gland and regulates thyroid hormone production by acting on the TSH receptor (TSH-R) on the basolateral membrane of thyroid follicular cells (2). In clinical practice, thyroid function is assessed by measuring circulating levels of TSH and free thyroxine (FT4). Elevated TSH levels indicate hypothyroidism, while decreased TSH levels suggest hyperthyroidism (3). The NHANES III study in the United States found that the overall prevalence of hyperthyroidism was 1.3% (0.5% overt hyperthyroidism and 0.7% subclinical hyperthyroidism), while the overall prevalence of hypothyroidism was 4.5% (0.3% overt hypothyroidism and 4.3% subclinical hypothyroidism) (4). Hypothyroidism is associated with cardiovascular dysfunction and is a risk factor for heart failure (5), as well as affecting the nervous, musculoskeletal, and gastrointestinal systems to varying degrees (6). Hyperthyroidism is associated with conditions such as atrial fibrillation, stroke, pulmonary embolism, and hypercoagulability (7). The etiology of hypothyroidism and hyperthyroidism is still not fully understood, but Graves’ disease and nodular thyroid disease are considered major causes of hyperthyroidism (8), while immune abnormalities, thyroidectomy, and excessive treatment for hyperthyroidism are considered major causes of hypothyroidism (6).

The concept of the thyroid-gut axis has been proposed and studied in relation to thyroid diseases and gut microbiota (9–11). According to a study, patients with hyperthyroidism exhibited a notable decrease in the abundance of *bifidobacteria* and *lactobacilli* in their gut compared to healthy individuals, along with an increase in *enterococci (* 9). Another study analyzed the differences in gut microbiota between patients with primary hypothyroidism and healthy individuals using 16S rRNA sequencing and found differences in taxa such as *Veillonella*, *Paraprevotella*, *Neisseria*, and *Rheinheimera (* 10). Although these studies confirmed the association between the thyroid and gut microbiota, it is difficult to determine whether dysbiosis of the gut microbiota is the cause or result of thyroid dysfunction. Moreover, observational studies have limitations in ruling out confounding factors between thyroid function and gut microbiota, such as lifestyle, environment, and age, making it challenging to establish a causal relationship.

Mendelian randomization (MR) employs genetic variation as instrumental variables to infer causal connections between exposures and outcomes. Since genotypes are randomly assigned from parents to offspring, Compared to traditional observational studies, MR has the distinct advantage of effectively reducing the influence of confounding factors and reverse causality, leading to enhanced credibility of research findings (12). MR has been widely used to explore the causal relationships between thyroid function and various diseases, such as the relationship between thyroid function and atrial fibrillation (13), lipid metabolism (14), and Alzheimer’s disease (15). In this study, we conducted a two-sample MR analysis using summary-level statistics from the MiBioGen Consortium, ThyroidOmics Consortium, and FinnGen Consortium genome-wide association studies (GWAS). The primary objective was to investigate the causal relationships between gut microbiota and FT4, TSH, hypothyroidism, and hyperthyroidism.

## 2 Methods

### 2.1 Study design

Mendelian randomization (MR) is an approach employed to evaluate causal connections between variables. This analysis necessitates meeting three core assumptions: (i) the instrumental variables (IVs) possess a robust association with the exposure factor, (ii) the IVs remain unaffected by confounding factors, and (iii) the IVs solely impact the outcome through the exposure factor (16). In this study, we used gut microbiota GWAS data as the exposure factor and FT4, TSH, hypothyroidism, and hyperthyroidism GWAS data as the outcomes. Based on inclusion criteria, we selected suitable SNPs as instrumental variables and conducted a two-sample MR analysis to examine the causal relationships between gut microbiota and FT4, TSH, hypothyroidism, and hyperthyroidism. It is worth mentioning that this study strictly adhered to the requirements of the STROBE-MR guidelines (17).

### 2.2 Data sources

The GWAS data on the gut microbiota originates from the comprehensive meta-analysis conducted by the MiBioGen consortium (18). The study included 18,340 individuals from 24 distinct cohorts. The primary objective was to analyze the microbial composition by focusing on variable regions V4, V3-V4, and V1-V2 of the 16S rRNA gene (18). The direct taxonomic binning method was used to classify the microbiota and performed microbial quantitative trait locus (mbQTL) analysis to identify host genetic variation loci associated with the abundance levels of bacterial taxa in the gut. And the study identified genetic variations associated with 9 phyla, 16 classes, 20 orders, 35 families, and 131 genera (18).

Thyroid function-related genetic variant data were obtained from the ThyroidOmics Consortium (3). Among these, TSH data were derived from 54,288 individuals across 22 cohorts, while FT4 data were obtained from 49,269 individuals across 19 cohorts.

Thyroid disease datasets were obtained from the FinnGen consortium R8 release data (19). The data for hypothyroidism included 42,000 cases and 292,316 controls, whereas the data for hyperthyroidism included 1,621 cases and 255,931 controls. **Supplementary Table 1** shows the details of the exposure and outcome analyzed in this MR study.

### 2.3 Selection of instrumental variables

To meet the requirements of Mendelian randomization (MR) studies, we first selected SNPs that were strongly associated with the exposure factor. Since very few instrumental variables (IVs) are obtained when the threshold is set at (*p*<5×10^-8^), we chose a threshold of (*p*<1×10^-5^) for SNP selection to obtain more IVs and obtain robust results.

Secondly, to account for linkage disequilibrium and avoid biased results, we set the linkage disequilibrium parameter (R^2^) for SNPs at 0.001 and the genetic distance at 10,000 kb. This ensured that each IV was independently present. We set the minor allele frequency level at 0.01 and excluded palindromic SNPs and those that were not present in the outcome.

Considering that IVs used as instrumental variables need to be strongly correlated with the exposure, we assessed the correlation between instrumental variables and the exposure using the F-statistic. We considered an F-statistic greater than 10 as indicating a strong correlation between the two. The formula for calculating the F-statistic is


$$F=\frac{R^{2}\left(N-K-1\right)}{K\left(1-R^{2}\right)}$$

,where


$$R^{2}=\frac{2\times EAF\times \left(1-EAF\right)\times Beta^{2}}{2\times EAF\times \left(1-EAF\right)\times Beta^{2}+\left[2\times EAF\times \left(1-EAF\right)\times N\times SE^{2}\right]}$$

(20).

### 2.4 Mendelian randomization analysis

The inverse-variance weighted (IVW) method is a primary approach used in Mendelian randomization (MR) analysis. This method uses a meta-analytic approach to synthesize the Wald estimates associated with each individual SNP, yielding an aggregate estimate of the collective influence of the gut microbiota on thyroid function. One fundamental assumption of Mendelian randomization (MR) is that there is no horizontal pleiotropy between the instrumental variables (genes) and the outcome variable. This means that the effects of these genes on the outcome are solely mediated through the exposure and not through any other pathways. If this assumption holds true, the IVW method can provide consistent and efficient estimates (21). If a causal relationship is identified by the IVW method (p<0.05), four additional methods, namely MR-Egger, weighted median method, simple mode method, and weighted mode method, are employed to complement the IVW results. The MR-Egger method does not enforce the intercept to be zero, allowing estimation of the causal effect even in the presence of invalid instruments (SNPs that can affect the outcome through non-exposure pathways), and the intercept can indicate the degree of horizontal pleiotropy (22). The weighted median method calculates the median of the ratio estimates after ranking the SNP estimates by their weights. This method requires a minimum of three SNPs, and if more than 50% of the SNPs have effects on the outcome that are not mediated through the exposure, the estimate provided by this method may be biased (23). The weighted mode method provides an consistent estimate if more than half of the SNPs were valid IVs (24). The simple mode method is similar to the weighted mode method, and if the causal effect estimates is derived from a majority of invalid SNPs, the estimate provided by this method may be biased (25). We set the significance threshold at *p*<0.05. Additionally, we performed Bonferroni correction for multiple testing, and causal relationships were represented by odds ratios (OR) and 95% confidence intervals (CI). When the IVW method yielded significant results and the directions of the other four methods were consistent, we considered a causal relationship between the exposure and the outcome. In the results with causal relationships, unknown taxa were excluded, and sensitivity analyses were subsequently conducted to ensure the stability of the results.

### 2.5 Heterogeneity and pleiotropy testing

Heterogeneity was assessed using Cochran’s Q method, with a significance level of *p<* 0.05 indicating significant heterogeneity (23, 26). The presence of horizontal pleiotropy was examined using the MR-Egger intercept test (27) and the MR-PRESSO global test (26). Outliers identified by the MR-PRESSO global test were excluded, and a robustness analysis was performed using the leave-one-out method to validate the results. A significance threshold of *p*< 0.05 was chosen for the analysis. To account for multiple testing, the Bonferroni method was employed. For each specific level, the significance threshold was adjusted to *p<* 0.05/n, where n represents the number of taxa present at that level. All statistical analyses and data visualizations were conducted using R software version 4.2.3^1^.

## 3 Results

### 3.1 Selection of instrumental variables

According to the selection criteria for instrumental variables (IV), a total of 1764 SNPs were chosen as instrumental variables. The F-statistics for the IVs are all greater than 10, indicating that the estimated coefficients are unlikely to be affected by weak instrument bias. Details of the selected IVs are shown in **Supplementary Table 2**.

### 3.2 FT4

**Figure 1:**
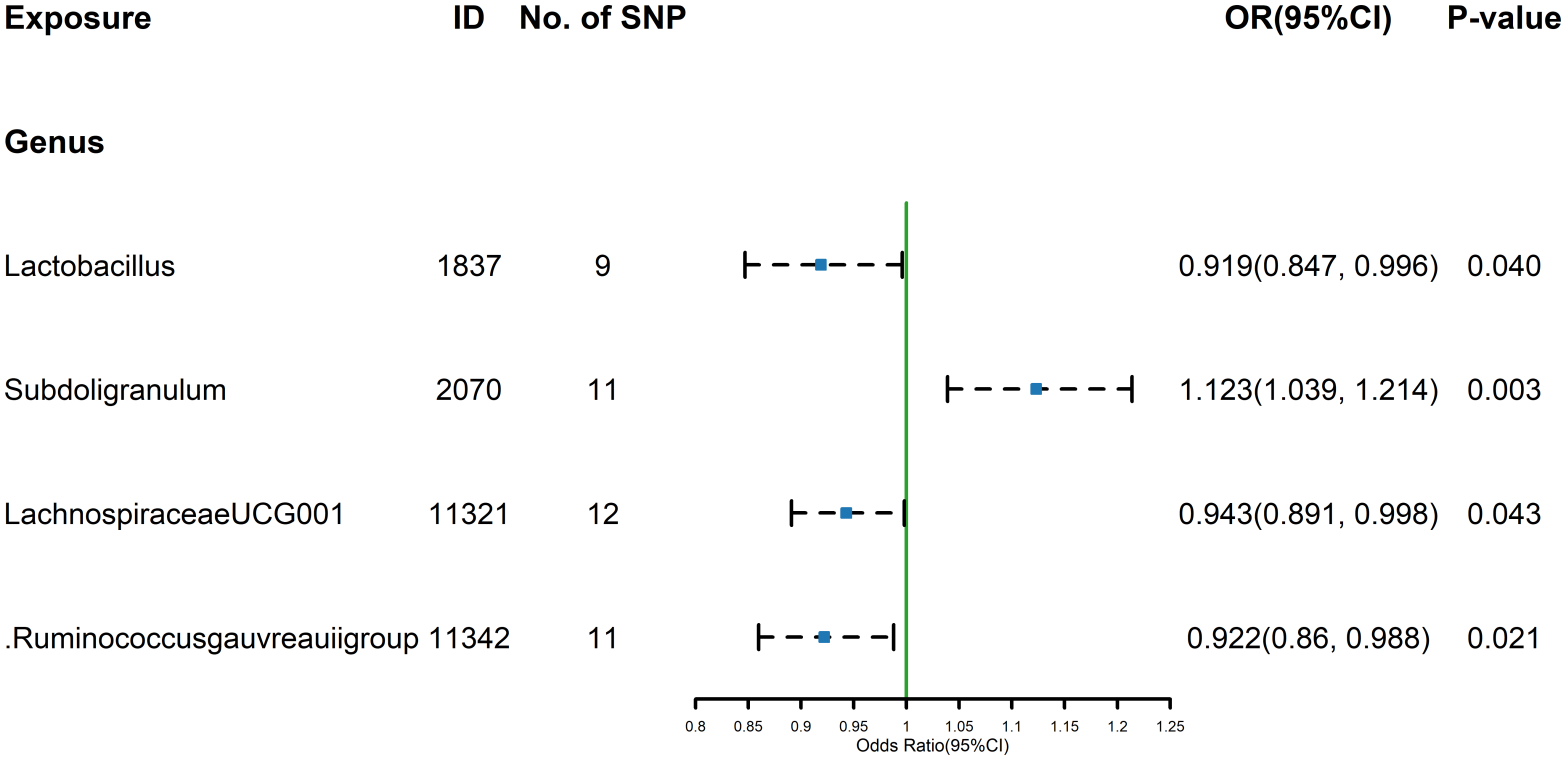
Forest plot of GM taxa associated with FT4 identified by IVW method.

The estimates calculated using the IVW test indicated that the genetically predicted relative abundance of genus *Lactobacillus* (id.1837) (OR=0.919, 95% CI: 0.847-0.996, P=0.040), genus *Lachnospiraceae UCG-001* (id.11321) (OR=0.943, 95%CI:0.891-0.998, P=0.043), genus *Ruminococcus gauvreauii group* (id.11342) (OR= 0.922, 95%CI: 0.860-0.988, P= 0.021) were associated with decreased FT4 levels, while genus *Subdoligranulum* (id.2070) (OR= 1.123,95%CI: 1.039-1.214, P= 0.003)was associated with increased FT4 levels (**Figure 1**). The other four methods, MR-Egger, weighted median, simple mode, and weighted mode were used to supplement the results of IVW. For details, refer to the table (**Supplementary Table 3**). Similarly, the results were parallel to the IVW results (**Supplementary Figure 1**). The result of Cochrane’s Q test showed that except for Lactobacillus (id.1837), which had a result of p< 0.05, indicating heterogeneity among SNPs, no significant heterogeneity was found among the other SNPs (p > 0.05).

### 3.3 TSH

**Figure 2:**
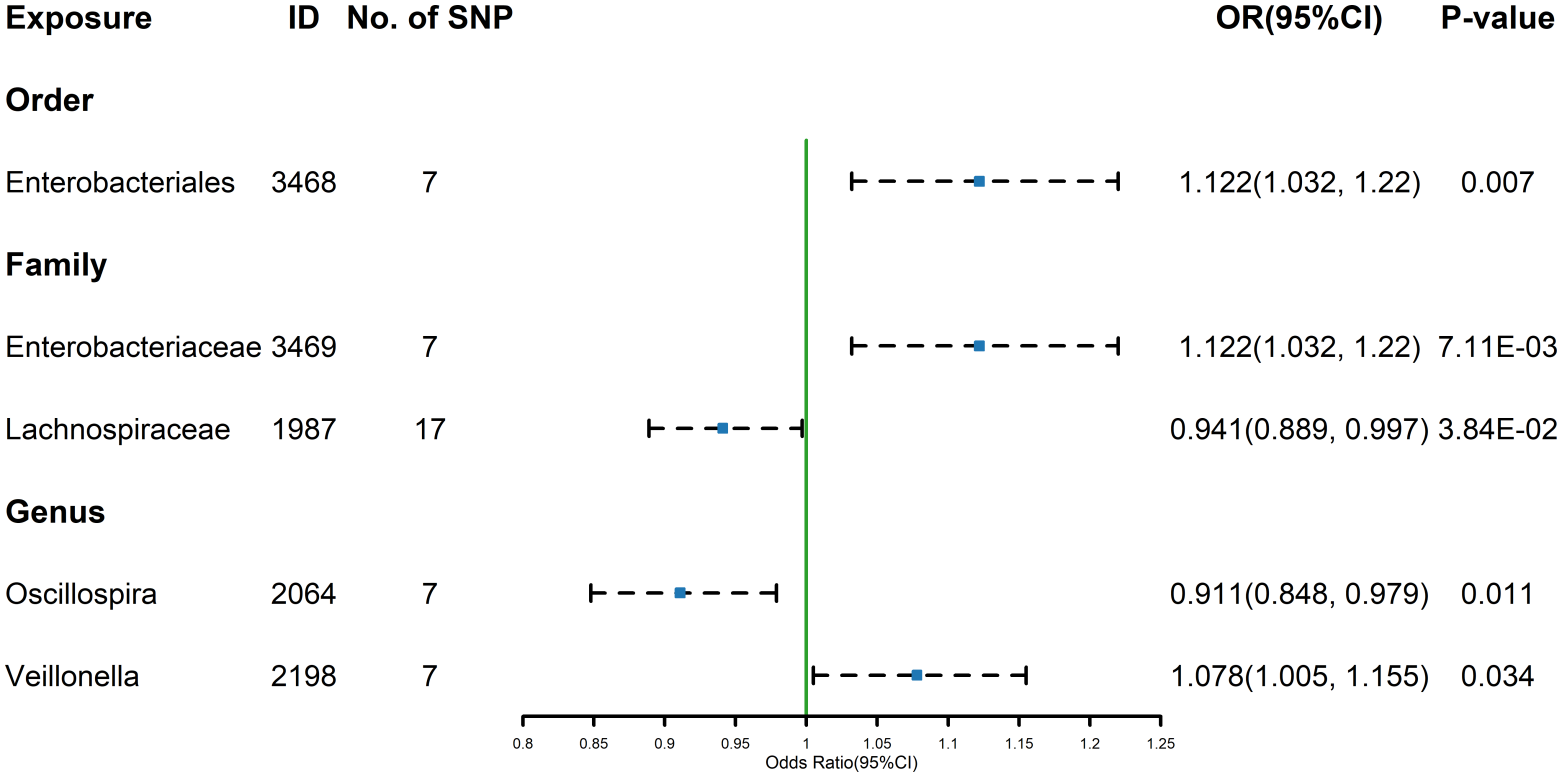
Forest plot of GM taxa associated with TSH identified by IVW method.

The estimates calculated using the IVW test indicated that the genetically predicted relative abundance of order *Enterobacteriales* (id.3468) (OR=1.122, 95%CI: 1.032-1.220, P=0.007) family *Enterobacteriaceae* (id.3469) (OR=1.122, 95%CI: 1.032-1.220, P=7.11E-03), genus *Veillonella* (id.2198) (OR=1.078, 95%CI: 1.005-1.155, P=0.034) were associated with increased TSH levels, while family *Lachnospiraceae* (id.1987) (OR=0.941, 95%CI: 0.889-0.997, P=3.84E-02)and genus *Oscillospira* (id.2064) (OR=0.911, 95%CI: 0.848-0.979, P=0.011) were associated with decreased TSH levels (**Figure 2**). Similarly, the results of the other four methods can be seen in the table (**Supplementary Table 4**). And the results were parallel to the IVW results (**Supplementary Figure 2**). The result of Cochrane’s Q test showed that no significant heterogeneity was found among the selected SNPs (p > 0.05).

### 3.4 Hypothyroidism

**Figure 3:**
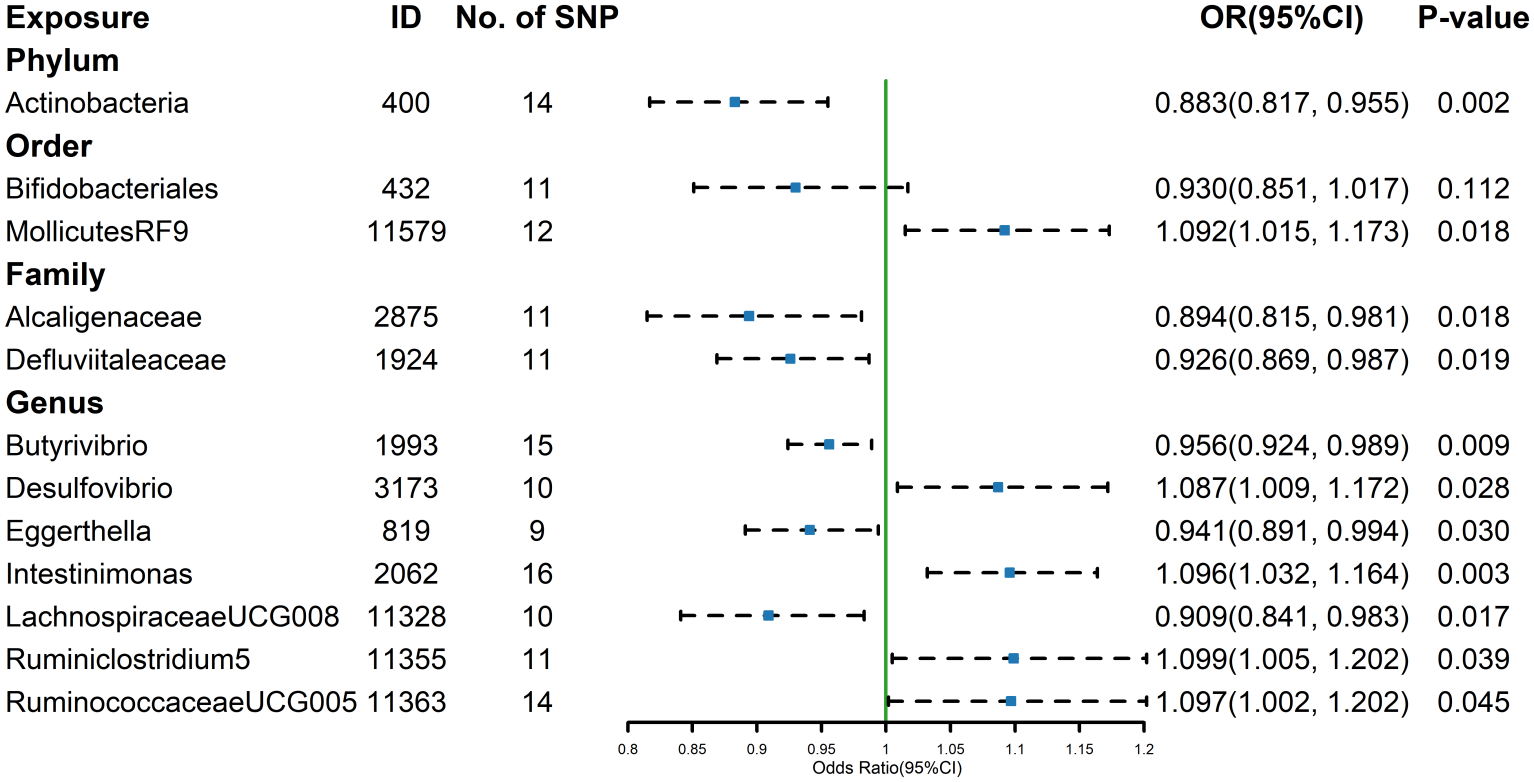
Forest plot of GM taxa associated with hypothyroidism identified by IVW method.

The estimates calculated using the IVW test indicated that the genetically predicted relative abundance of order *Mollicutes RF9* (id.11579) (OR=1.092, 95%CI: 1.015-1.173, P=0.018), genus *Desulfovibrio* (id.3173) (OR=1.087, 95%CI: 1.009-1.172, P=0.028), genus *Intestinimonas* (id.2062) (OR=1.096, 95%CI: 1.032-1.164, P=0.003), genus *Ruminiclostridium 5* (id.11355) (OR=1.099, 95%CI: 1.005-1.202, P=0.039), genus *Ruminococcaceae UCG-005* (id.11363) (OR=1.097, 95%CI: 1.002-1.202, P=0.045) were positively associated with the risk of hypothyroidism, while phylum *Actinobacteria* (id.400) (OR=0.883, 95%CI: 0.817-0.955, P=0.002), family *Alcaligenaceae* (id.2875) (OR=0.894, 95%CI: 0.815-0.981, P=0.018), family *Defluviitaleaceae* (id.1924) (OR=0.926, 95%CI: 0.869-0.987, P=0.019), genus *Butyrivibrio* (id.1993) (OR=0.956, 95%CI: 0.924-0.989, P=0.009), genus *Eggerthella* (id.819) (OR=0.941, 95%CI: 0.891-0.994, P=0.030), genus *Lachnospiraceae UCG-008* (id.11328) (OR=0.909, 95%CI: 0.841-0.983, P=0.017) were negatively associated with the risk of hypothyroidism (**Figure 3**). The results of the other four methods can be seen in the table (**Supplementary Table 5**). And the results were parallel to the IVW results (**Supplementary Figure 3**). The result of Cochrane’s Q test showed that there was heterogeneity among the selected SNPs for *Lachnospiraceae UCG-008* (id.11328) and *Ruminococcaceae UCG-005* (id.11363), while no significant heterogeneity was found among the other selected SNPs (p > 0.05).

### 3.5 Hyperthyroidism

**Figure 4:**
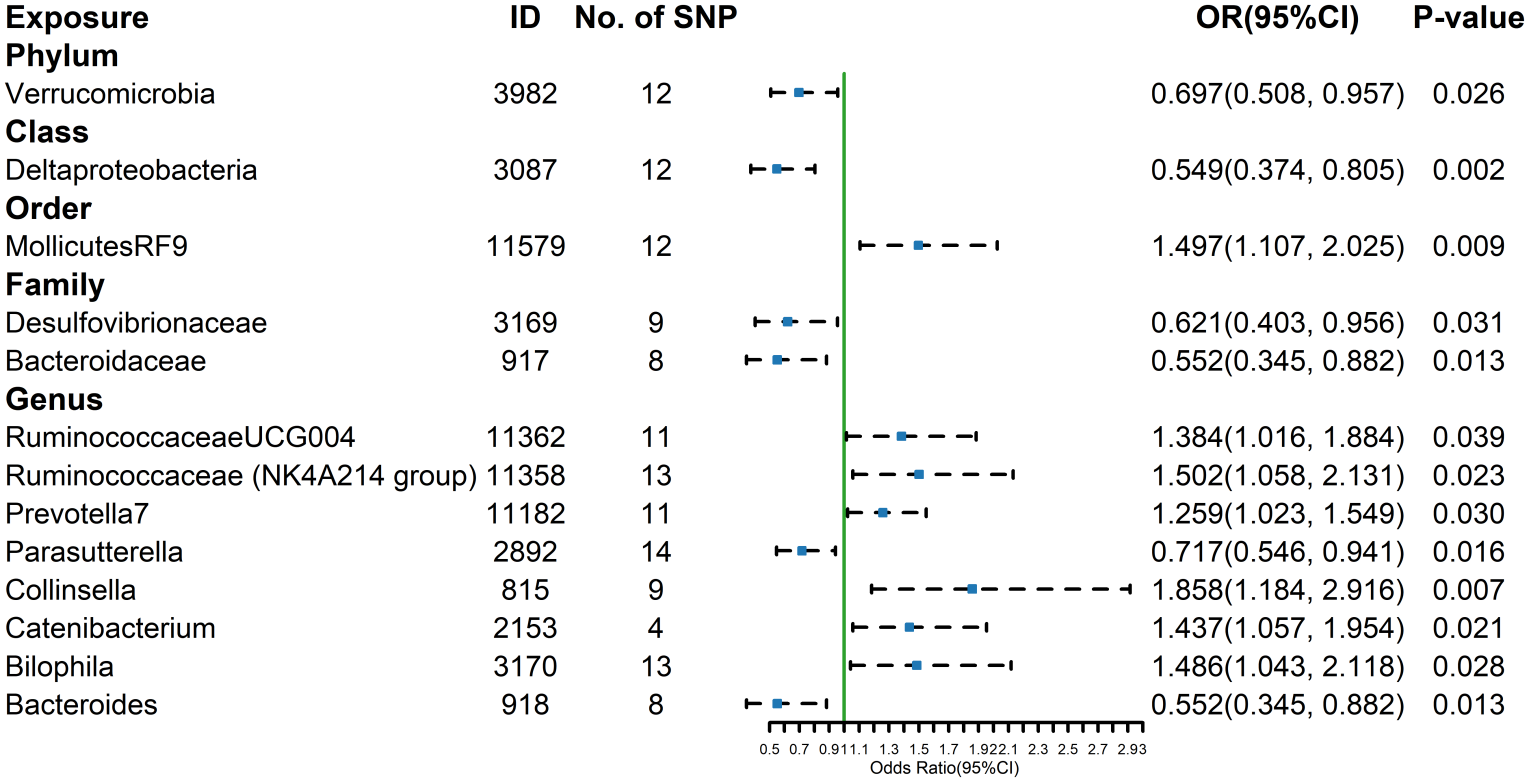
Forest plot of GM taxa associated with hypethyroidism identified by IVW method.

The estimates calculated using the IVW test indicated that the genetically predicted relative abundance of order *Mollicutes RF9* (id.11579) (OR= 1.497, 95%CI: 1.107-2.025, P=0.009), genus *Ruminococcaceae UCG-004* (id.11362) (OR= 1.384, 95%CI: 1.016-1.884, P=0.039), genus *Ruminococcaceae NK4A214 group* (id.11358) (OR= 1.502, 95%CI: 1.058-2.131, P= 0.023), *genus Prevotella7* (id.11182) (OR= 1.259, 95%CI: 1.023-1.549, P=0.030), *genus Collinsella* (id.815) (OR= 1.858, 95%CI: 1.184-2.916, P=0.007), genus *Catenibacterium* (id.2153) (OR= 1.437, 95%CI: 1.057-1.954, P=0.021), genus *Bilophila* (id.3170) (OR= 1.486, 95%CI: 1.043-2.118, P=0.028) were positively associated with the risk of hyperthyroidism, while Phylum *Verrucomicrobia* (id.3982) (OR=0.697,95%CI: 0.508-0.957, P=0.026), class *Deltaproteobacteria* (id.3087) (OR=0.549, 95%CI: 0.374-0.805, P=0.002), family *Desulfovibrionaceae* (id.3169) (OR=0.621, 95%CI: 0.403-0.956, P=0.031), family *Bacteroidaceae* (id.917) (OR=0.552, 95%CI: 0.345-0.882, P=0.013), genus *Parasutterella* (id.2892) (OR=0.717, 95%CI: 0.546-0.941, P=0.016), genus *Bacteroides* (id.918) (OR=0.552, 95%CI: 0.345-0.882, P=0.013) were negatively associated with the risk of hyperthyroidism (**Figure 4**). And the results were parallel to the IVW results (**Supplementary Figure 4**).The results of the other four methods can be seen in the table (**Supplementary Table 6**). The result of Cochrane’s Q test showed that no significant heterogeneity was found among the selected SNPs (p > 0.05).

### 3.6 Sensitivity analysis

The intercept tests of MR-Egger and the MR-PRESSO global test did not indicate the presence of horizontal pleiotropy (**Tables 1**, **2**, **3**, **4**). Additionally, the leave-one-out analysis did not reveal any SNP that would significantly alter the overall results, ensuring the stability of the outcomes (**Supplementary Figures 5-8**). And the results of Cochrane’s Q test can be found in **Tables 1**–**4**. The results of the Bonferroni correction test showed that phylum *Actinobacteria* (id.400) (OR=0.883, 95% CI: 0.817-0.955, P=0.002) exhibited a protective effect against hypothyroidism, while class *Deltaproteobacteria* (id.3087) (OR=0.549, 95% CI: 0.374-0.805, P=0.002) exhibited a protective effect against hyperthyroidism. Furthermore, the funnel plot for all the results can be seen in **Supplementary Figures 9-12**. The analysis of reverse Mendelian randomization showed no significant causal relationship. However, there were two nominally significant causal relationships that hyperthyroidism was associated with an increased risk of order *MollicutesRF9* (id.11579) (OR=1.067, 95% CI: 1.0003-1.138, *P*=0.0488), while hypothyroidism was associated with an increased risk of genus *Ruminococcaceae UCG005* (OR=1.038, 95% CI: 1.001-1.078, *P*=0.0497).

**Table 1:** Horizontal pleiotropy analysis for IVs of 4 GM taxa associated with FT4.

Exposure	MR-Egger intercept test			MR-PRESSO global test		Cochran’s Q
	Egger_ intercept	SE	*p*-value	RSS obs	*p*-value	*p*-value
Genus						
Lactobacillus(ID:1837)	-5.56E-03	1.54E-02	0.730	21.053	0.050	0.027
Subdoligranulum(ID:2070)	1.32E-03	7.27E-03	0.860	9.163	0.703	0.673
LachnospiraceaeUCG001(ID:11321)	-4.84E-03	1.23E-02	0.702	12.957	0.466	0.454
Ruminococcusgauvreauiigroup(ID:11342)	-3.84E-03	1.22E-02	0.760	6.231	0.879	0.883

SE, standard error; RSS, residual sum of squares; Cochran’s Q-derived.

**Table 2:** Horizontal pleiotropy analysis for IVs of 5 GM taxa associated with TSH.

Exposure	MR-Egger intercept test			MR-PRESSO global test		Cochran’s Q
	Egger_ intercept	SE	*p*-value	RSS obs	*p*-value	*p*-value
Order						
Enterobacteriales(ID:3468)	-1.84E-02	1.89E-02	0.375	4.236	0.817	0.787
Family						
Enterobacteriaceae(ID:3469)	-1.84E-02	1.89E-02	0.375	4.236	0.825	0.787
Lachnospiraceae(ID:1987)	-9.33E-04	4.77E-03	0.848	9.527	0.931	0.930
Genus						
Oscillospira(ID:2064)	3.06E-03	1.38E-02	0.833	4.115	0.829	0.792
Veillonella(ID:2198)	-2.60E-02	2.26E-02	0.302	6.152	0.637	0.596

SE, standard error; RSS, residual sum of squares; Cochran’s Q-derived.

**Table 3:** Horizontal pleiotropy analysis for IVs of 12 GM taxa associated with HYPOTHYROIDISM.

Exposure	MR-Egger intercept test			MR-PRESSO global test		Cochran’s Q
	Egger_ intercept	SE	*p*-value	RSS obs	*p*-value	*p*-value
Phylum						
Actinobacteria(ID:400)	0.016	0.010	0.135	13.397	0.579	0.569
Order						
Bifidobacteriales(ID:432)	-0.004	0.011	0.700	18.457	0.151	0.135
MollicutesRF9(ID:11579)	-0.008	0.009	0.398	15.689	0.308	0.267
Family						
Alcaligenaceae(ID:2875)	0.010	0.014	0.502	12.874	0.423	0.393
Defluviitaleaceae(ID:1924)	0.007	0.011	0.526	4.932	0.941	0.944
Genus						
Butyrivibrio(ID:1993)	-0.001	0.011	0.925	15.269	0.498	0.487
Desulfovibrio(ID:3173)	-0.010	0.011	0.403	12.710	0.385	0.332
Eggerthella(ID:819)	0.011	0.014	0.446	7.656	0.652	0.619
Intestinimonas(ID:2062)	-0.005	0.007	0.516	15.492	0.578	0.568
LachnospiraceaeUCG008(ID:11328)	0.044	0.016	0.026	21.512	0.061	0.050
Ruminiclostridium5(ID:11355)	-0.015	0.012	0.234	12.236	0.466	0.468
RuminococcaceaeUCG005(ID:11363)	0.008	0.011	0.465	26.541	0.055	0.039

SE, standard error; RSS, residual sum of squares; Cochran’s Q-derived.

**Table 4:** Horizontal pleiotropy analysis for IVs of 13 GM taxa associated with HYPETHYROIDISM.

Exposure	MR-Egger intercept test			MR-PRESSO global test		Cochran’s Q
	Egger_ intercept	SE	*p*-value	RSS obs	*p*-value	*p*-value
Phylum						
Verrucomicrobia(ID:3982)	-0.001	0.037	0.977	12.373	0.539	0.572
Class						
Deltaproteobacteria(ID:3087)	-0.014	0.058	0.814	10.072	0.699	0.671
Order						
MollicutesRF9(ID:11579)	0.016	0.038	0.678	9.383	0.767	0.730
Family						
Desulfovibrionaceae(ID:3169)	0.006	0.061	0.928	6.658	0.738	0.729
Bacteroidaceae(ID:917)	-0.026	0.080	0.754	4.070	0.890	0.875
Genus						
RuminococcaceaeUCG004(ID:11362)	-0.013	0.077	0.873	12.308	0.447	0.424
RuminococcaceaeNK4A214group(ID:11358)	-0.063	0.043	0.173	13.66	0.493	0.472
Prevotella7(ID:11182)	-0.089	0.086	0.326	14.108	0.338	0.321
Parasutterella(ID:2892)	-0.012	0.031	0.713	8.297	0.912	0.910
Collinsella(ID:815)	-0.020	0.063	0.765	9.920	0.484	0.450
Catenibacterium(ID:2153)	-0.225	0.252	0.467	2.427	0.737	0.708
Bilophila(ID:3170)	-0.079	0.063	0.236	14.727	0.407	0.397
Bacteroides(ID:918)	-0.026	0.080	0.754	4.070	0.893	0.875

SE, standard error; RSS, residual sum of squares; Cochran’s Q-derived.

## 4 Discussion

This study comprehensively assessed the causal effects of 211 taxonomic groups within the domain of Genus and Species (from phylum to genus levels) on thyroid function, encompassing parameters such as FT4, TSH, hypothyroidism, and hyperthyroidism. Ultimately, a total of 34 causal relationships were identified, comprising 32 nominal causal associations and 2 strong causal connections. These findings underscore the significance of the gut microbiota in influencing thyroid functionality.

An increasing body of evidence suggests that the gut microbiota plays a pivotal role in the pathogenesis of thyroid disorders. Primarily, the gut microbiota may modulate thyroid function by influencing the uptake of thyroid-relevant micronutrients (28). The synthesis of thyroid hormones necessitates the presence of iodine, and inadequate iodine intake can impair thyroid function (29). Iodine is required to be absorbed through the gastrointestinal tract and transferred to the thyroid. Hence, the gut microbiota exerts a significant influence on iodine metabolism regulation. Within the human gastrointestinal tract, iodine uptake is predominantly facilitated by the sodium-iodide symporter (NIS) (30). Lipopolysaccharides (LPS) and short-chain fatty acids (SCFAs) released by the gut microbiota can modulate iodine uptake by affecting the expression and activity of NIS (31, 32). Additionally, lipopolysaccharides (LPS) can stimulate the synthesis of type 2 iodothyronine deiodinase (D2) in the rat hypothalamic paraventricular nucleus, thus promoting the conversion of T4 to T3 and inhibiting the secretion of pituitary TSH (33). Besides iodine, iron is also an essential trace element for thyroid hormone synthesis, and iron deficiency is a common symptom of hypothyroidism (34). Moreover, the gut microbiota may lower intestinal pH and enhance the bioavailability of colonic iron through the production of short-chain fatty acids (SCFAs), thereby influencing thyroid function (35). The thyroid, as the organ with the highest selenium content per unit tissue, contains selenium in the form of selenoproteins. Enzymes such as glutathione peroxidase, deiodinases (D1, D2, and D3), and thioredoxin reductase, which are selenium-dependent, maintain the stability and activity of thyroid hormones within the body (36). The gut microbiota can influence selenium metabolism and absorption in the colon (37). Furthermore, the gut microbiota can impact the secretion of the neurotransmitter dopamine in the brain and regulate the hypothalamic-pituitary-adrenal (HPA) axis (38). Dopamine, in turn, can inhibit the secretion of TSH within the body, thus affecting thyroid function (39). Autoimmune thyroid diseases (AITD) represent the most common group of autoimmune disorders, of which Hashimoto’s thyroiditis (HT) and Graves’ disease (GD) are major causes of hypothyroidism and hyperthyroidism, respectively. Research suggests that the gut microbiota and its metabolites might directly or indirectly impact thyroid immunity, thereby contributing to the development of AITD. These regulatory mechanisms may encompass inducing the shift from type 1 (Th1) to type 2 (Th2) T-helper cell responses (40), activation of toll-like receptor 4 by lipopolysaccharides (LPS) (41), and the induction of changes in transcriptional, proteomic, and metabolic changes.

In our study, we established two strong causal relationships. The phylum *Actinobacteria*, after Bonferroni correction, exhibited a significant reduction in the risk of hypothyroidism, while the class *Deltaproteobacteria*, post Bonferroni correction, demonstrated a significant reduction in the risk of hyperthyroidism. *Actinobacteria* is one of the four major phyla of the intestinal microbiota and is essential in maintaining intestinal homeostasis (42). Numerous *Actinobacteria* participate in the maintenance of microbial homeostasis, with some being considered probiotics that potentially exert an influence on thyroid function. *Bifidobacterium*, as a probiotic strain within the phylum Actinobacteria, is purported to possess the capacity to induce immune regulatory responses and mitigate inflammatory reactions (43). Several observational studies have suggested a diminished prevalence of *Bifidobacteriaceae* within individuals afflicted by hypothyroidism and hyperthyroidism (10, 44). And the administration of a mixture of *Lactobacillus* and *Bifidobacterium* in hypothyroid patients is effective in reducing the patient’s need for LT-4 amounts, resulting in more stable thyroid hormone levels (45, 46). *Deltaproteobacteria*, a class of Gram-negative proteobacteria, comprises obligate anaerobes, including numerous sulfate-reducing bacteria and sulfur-reducing bacteria (47). Among them, *Desulfovibrionaceae* is a prominent bacterial family within the class Deltaproteobacteria and is implicated in several diseases (48). In an animal experiment, iodine supplementation led to elevated levels of TT4, FT3, and TT3 in male ICR mice compared to the control group. However, in female mice, these values decreased in comparison to the control group. Subsequent analysis of intergroup fecal differences in mice revealed that iodine-supplemented female ICR mice exhibited higher abundance of *Desulfovibrionaceae (* 49). Therefore, we postulate that the *Desulfovibrionaceae* family could potentially impact thyroid function through certain pathways. The specific mechanisms underlying this phenomenon should be further validated through experimental design.

Considering the potential for false negatives arising from Bonferroni correction, it becomes imperative to delve into the latent mechanisms underpinning the impact of the 32 nominal causal relationships on thyroid function. Preceding research has already elucidated a notable positive correlation between *Oscillospira* and diets low in fat and lean meat, as well as with human health. Moreover, *Oscillospira* has been demonstrated to engender various short-chain fatty acids, prominently including butyrate (50). Concurrently, there exists a positive correlation between body mass index (BMI) and serum thyroid-stimulating hormone (TSH) levels (51). Based on these findings, it is conceivable that *Oscillospira*, by generating short-chain fatty acids and reducing BMI, might exert regulatory influence on TSH levels. Furthermore, *Veillonella* genus, a non-motile anaerobic Gram-negative diplococcus, resides within the human gastrointestinal tract (52). Discrepancies in microbial composition have been noted between patients afflicted by primary hypothyroidism and healthy individuals (10). An illustrative case-control study has evidenced a significant reduction in *Veillonella* abundance among children with attention deficit hyperactivity disorder (ADHD) (53). Given the association of ADHD with neurotransmitter imbalances like dopamine (53, 54), a plausible hypothesis emerges that *Veillonella* could potentially modulate the hypothalamic-pituitary-adrenal (HPA) axis and dopamine secretion, thereby exerting an inhibitory effect on TSH levels. Expanding further, in patients with hypothyroidism (HT) compared to their euthyroid counterparts, heightened abundances of microbial taxa including *Lachnospiraceae incertae sedis, Lactonifactor, Alistipes*, and *Subdoligranulum* have been observed (55). Integrating our research outcomes, we may conjecture that *Subdoligranulum* and *Lachnospiraceae* might influence free thyroxine (FT4) levels, thus impacting thyroid function. However, the intricate mechanisms necessitate in-depth investigation. The specific mechanisms require further investigation.

The present study has several advantages, firstly this is the first study to investigate the association between gut flora and thyroid function using GWAS data from the Finnish Biosample Repository and the Thyroidomics Consortium and the MiBiogen Consortium, secondly, MR method analysis can provide robust causality estimates by minimizing reverse causal effects or confounding factors, and finally we performed sensitivity analysis of the results by Mr-Egger intercept test and MR-PRESSO global test to ensure the robustness of the results. However, this study has some limitations, first we used summary level statistics, could not analyze specific disease subgroups, and did not explore the non-linear relationship between exposure and outcome. In addition, we studied a European population, which limits generalizability to other ethnicities. Thyroid disease is more prevalent in female populations (56), however our study did not differentiate between genders, which may limit the understanding of the differential impact of gut flora on thyroid disease production by gender.

## 5 Conclusions

In conclusion, in this study, we conducted a two-sample MR analysis to investigate the causal relationships between gut microbiota and FT4, TSH, hypothyroidism, and hyperthyroidism. After using the bonferroni method for multiple testing correction, we identified 2 causal relationships and 32 nominally significant causal relationships. However, further research is needed to explore the specific effects of gut microbiota on thyroid function.

## Data availability statement

The original contributions presented in the study are publicly available. This data can be found at the MiBioGen repository (https://mibiogen.gcc.rug.nl/), the FinnGen repository (https://r8.finngen.fi/) and the ThyroidOmics Consortium (https://transfer.sysepi.medizin.uni-greifswald.de/thyroidomics/datasets/).

## Ethics statement

This research has been conducted using published studies and consortia providing publicly available summary statistics. Therefore, no additional separate ethical approval was required for this study.

## Author contributions

LX designed the study, analyzed the data, and wrote the manuscript. HZ assisted in creating the tables and revising the manuscript. WC critically read and edited the manuscript. All authors contributed to the article and approved the submitted version.

## Footnotes

1
https://www.r-project.org/
