# Supplementary material for: Relationship between gut microbiota and thyroid function: a two-sample Mendelian randomization study
Source: Front Endocrinol (Lausanne). 2023 Sep 26;14:1240752. doi: 10.3389/fendo.2023.1240752 (PMC10562735; doi:10.3389/fendo.2023.1240752)
Supplement: Supplementary file 1 [file DataSheet_1.pdf]

Supplementary Information

Relationship between gut microbiota and thyroid function: a two-sample Mendelian randomization study

Liangzhuo Xie[1],Huaye Zhao[1],Wei Chen[1,2*]

Supplement Table S1. Summary presentation of included studies

Consortium	Trait	Sample size
MiBioGen	211 gut microbiota	18340
Finngen(R8)	hypothyroidism	42,000 cases and 292,316 controls
Finngen(R8)	hypethyroidism	1,621 cases and 255,931 controls
ThyroidOmics	FT4	49,269
ThyroidOmics	TSH	54,288

Supplement Figure S1(1-4)

Scatter plots of significant and nominal significant estimates from genetically predicted microbiotas on FT4:

(1) genus.Ruminococcusgauvreauiigroup
(2) genus.LachnospiraceaeUCG001
(3) genus.Subdoligranulum
(4) genus.Lactobacillus

Supplement Figure S2(5-9)

Scatter plots of significant and nominal significant estimates from genetically predicted microbiotas on TSH:

(5) order.Enterobacteriales
(6) family.Enterobacteriaceae
(7) family.Lachnospiraceae
(8) genus.Oscillospira
(9) genus.Veillonella

Supplement Figure S3(10-21)

Scatter plots of significant and nominal significant estimates from genetically predicted microbiotas on Hypothyroidism:

(10) phylum.Actinobacteria
(11) order.Bifidobacteriales
(12) order.MollicutesRF9
(13) family.Alcaligenaceae
(14) family.Defluviitaleaceae
(15) genus.Butyrivibrio
(16) genus.Desulfovibrio
(17) genus.Eggerthella
(18) genus.Intestinimonas
(19) genus.LachnospiraceaeUCG008
(20) genus.Ruminiclostridium5
(21) genus.RuminococcaceaeUCG005

Supplement Figure S4(22-34)

Scatter plots of significant and nominal significant estimates from genetically predicted microbiotas on Hypethyroidism:

(22) phylum.Verrucomicrobia
(23) class.Deltaproteobacteria
(24) order.MollicutesRF9
(25) family.Bacteroidaceae
(26) family.Desulfovibrionaceae
(27) genus.Bacteroides
(28) genus.Bilophila
(29) genus.Catenibacterium
(30) genus.Collinsella
(31) genus.Parasutterella
(32) genus.Prevotella7
(33) genus.RuminococcaceaeNK4A214
(34) genus.RuminococcaceaeUCG004

Supplement Figure S5(35-38)

Leave-one-out plots of significant and nominal significant estimates from genetically predicted microbiotas on FT4:

(35) genus.Ruminococcusgauvreauiigroup
(36) genus.LachnospiraceaeUCG001
(37) genus.Subdoligranulum
(38) genus.Lactobacillus

Supplement Figure S6(39-43)

Leave-one-out plots of significant and nominal significant estimates from genetically predicted microbiotas on TSH:

(39) order.Enterobacteriales
(40) family.Enterobacteriaceae
(41) family.Lachnospiraceae
(42) genus.Oscillospira
(43) genus.Veillonella

Supplement Figure S7(44-55)

Leave-one-out plots of significant and nominal significant estimates from genetically predicted microbiotas on Hypothyroidism:

(44) phylum.Actinobacteria
(45) order.Bifidobacteriales
(46) order.MollicutesRF9
(47) family.Alcaligenaceae
(48) family.Defluviitaleaceae
(49) genus.Butyrivibrio
(50) genus.Desulfovibrio
(51) genus.Eggerthella
(52) genus.Intestinimonas
(53) genus.LachnospiraceaeUCG008
(54) genus.Ruminiclostridium5
(55) genus.RuminococcaceaeUCG005

Supplement Figure S8(56-68)

Leave-one-out plots of significant and nominal significant estimates from genetically predicted microbiotas on Hypethyroidism:

(56) phylum.Verrucomicrobia
(57) class.Deltaproteobacteria
(58) order.MollicutesRF9
(59) family.Bacteroidaceae
(60) family.Desulfovibrionaceae
(61) genus.Bacteroides
(62) genus.Bilophila
(63) genus.Catenibacterium
(64) genus.Collinsella
(65) genus.Parasutterella
(66) genus.Prevotella7
(67) genus.RuminococcaceaeNK4A214
(68) genus.RuminococcaceaeUCG004

Supplement Figure S9(69-72)

Funnel plots of significant and nominal significant estimates from genetically predicted microbiotas on FT4:

(69) genus.Ruminococcusgauvreauiigroup
(70) genus.LachnospiraceaeUCG001
(71) genus.Subdoligranulum
(72) genus.Lactobacillus

Supplement Figure S10(73-77)

Funnel plots of significant and nominal significant estimates from genetically predicted microbiotas on TSH:

(73) order.Enterobacteriales
(74) family.Enterobacteriaceae
(75) family.Lachnospiraceae
(76) genus.Oscillospira
(77) genus.Veillonella

Supplement Figure S11(78-89)

Funnel plots of significant and nominal significant estimates from genetically predicted microbiotas on Hypothyroidism:

(78) phylum.Actinobacteria
(79) order.Bifidobacteriales
(80) order.MollicutesRF9
(81) family.Alcaligenaceae
(82) family.Defluviitaleaceae
(83) genus.Butyrivibrio
(84) genus.Desulfovibrio
(85) genus.Eggerthella
(86) genus.Intestinimonas
(87) genus.LachnospiraceaeUCG008
(88) genus.Ruminiclostridium5
(89) genus.RuminococcaceaeUCG005

Supplement Figure S12(90-102)

Funnel plots of significant and nominal significant estimates from genetically predicted microbiotas on Hypethyroidism:

(90) phylum.Verrucomicrobia
(91) class.Deltaproteobacteria
(92) order.MollicutesRF9
(93) family.Bacteroidaceae
(94) family.Desulfovibrionaceae
(95) genus.Bacteroides
(96) genus.Bilophila
(97) genus.Catenibacterium
(98) genus.Collinsella
(99) genus.Parasutterella
(100) genus.Prevotella7
(101) genus.RuminococcaceaeNK4A214
(102) genus.RuminococcaceaeUCG004

1

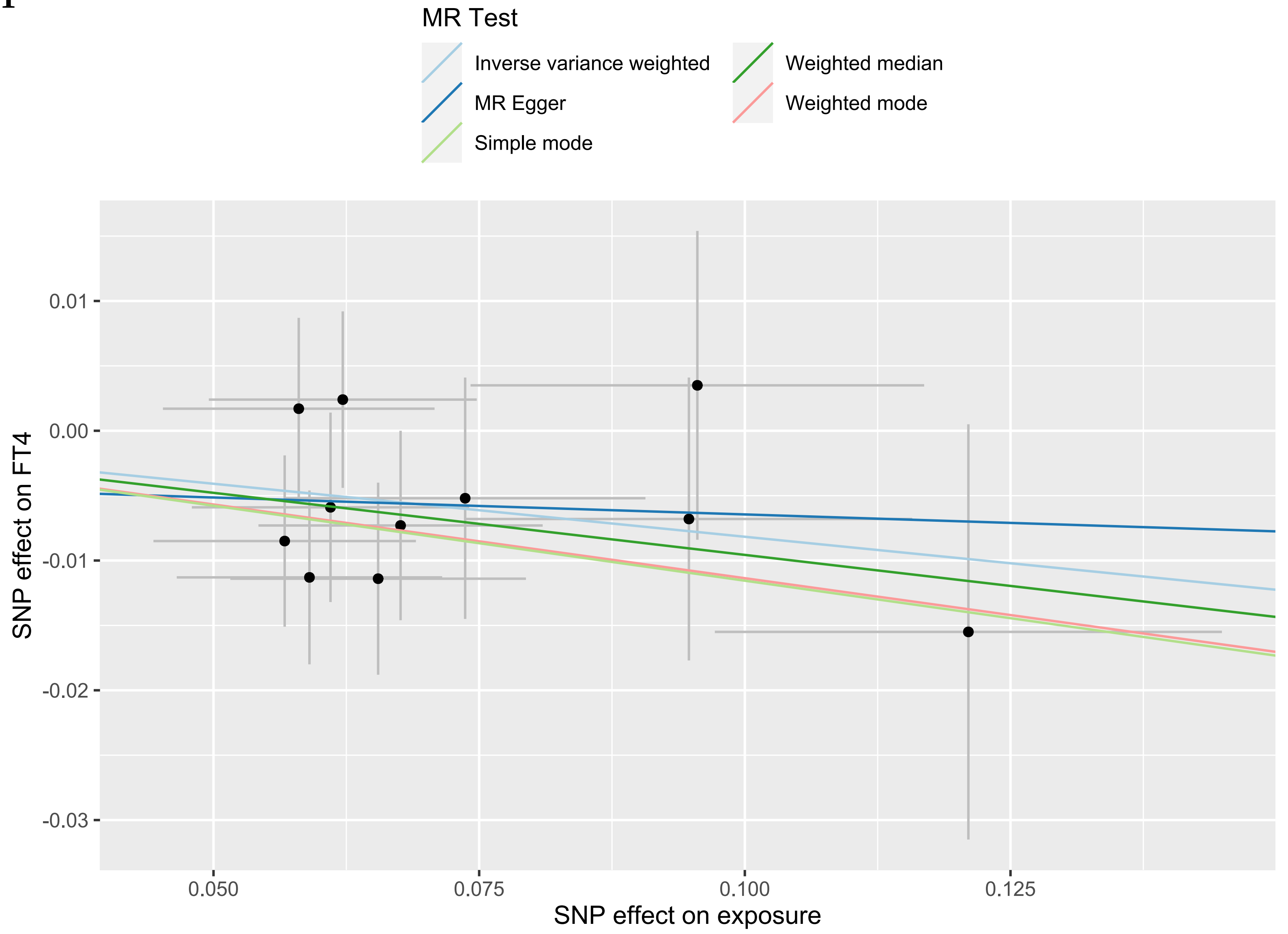

MR Test
Inverse variance weighted
MR Egger
Simple mode
Weighted median
Weighted mode
0.01
0.00
-0.01
-0.02
-0.03
SNP effect on FT4
0.050
0.075
0.100
0.125
SNP effect on exposure

2

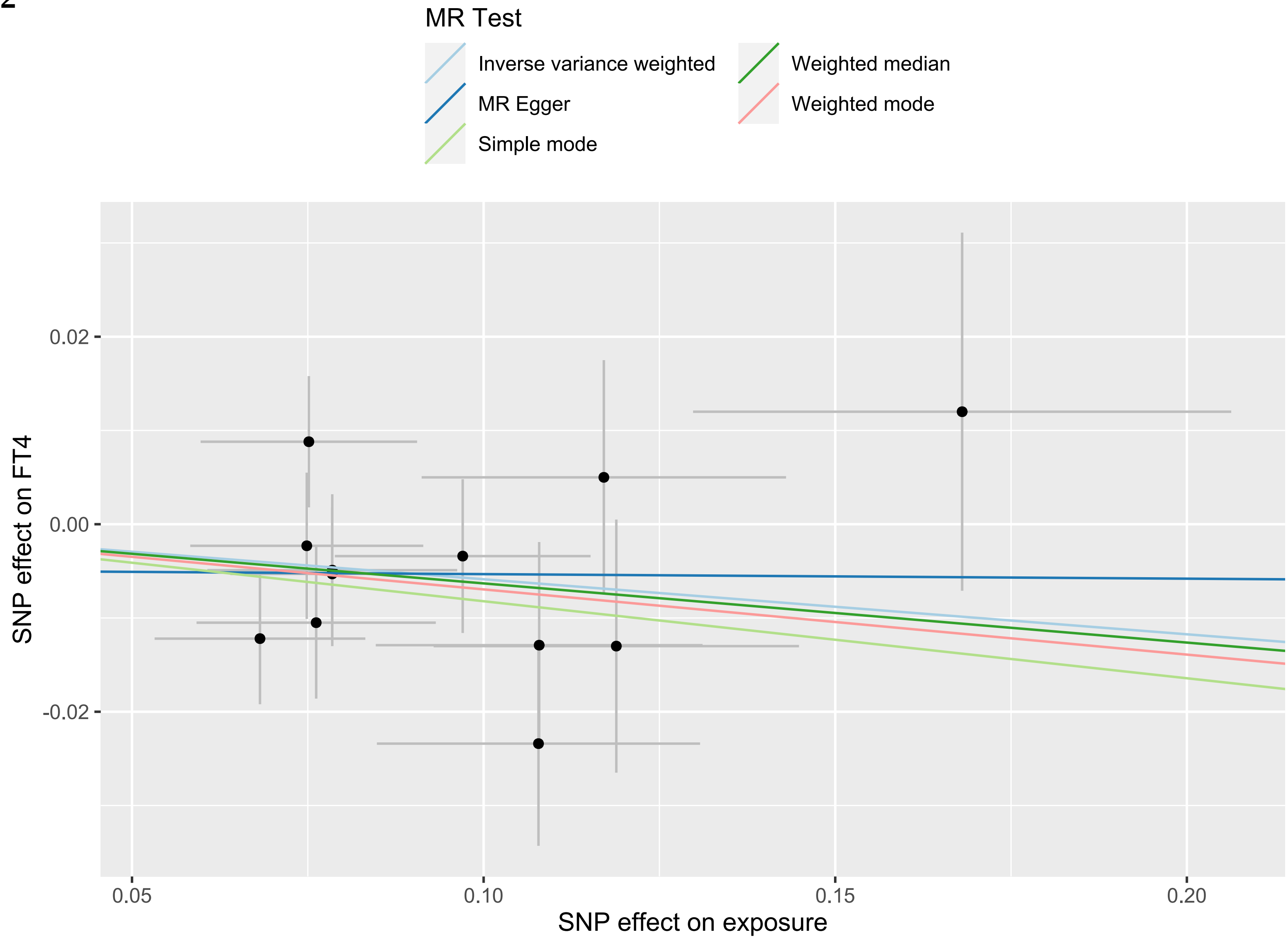
MR Test
Inverse variance weighted
MR Egger
Simple mode
Weighted median
Weighted mode
0.02
0.00
-0.02
SNP effect on FT4
0.05
0.10
0.15
0.20
SNP effect on exposure

3

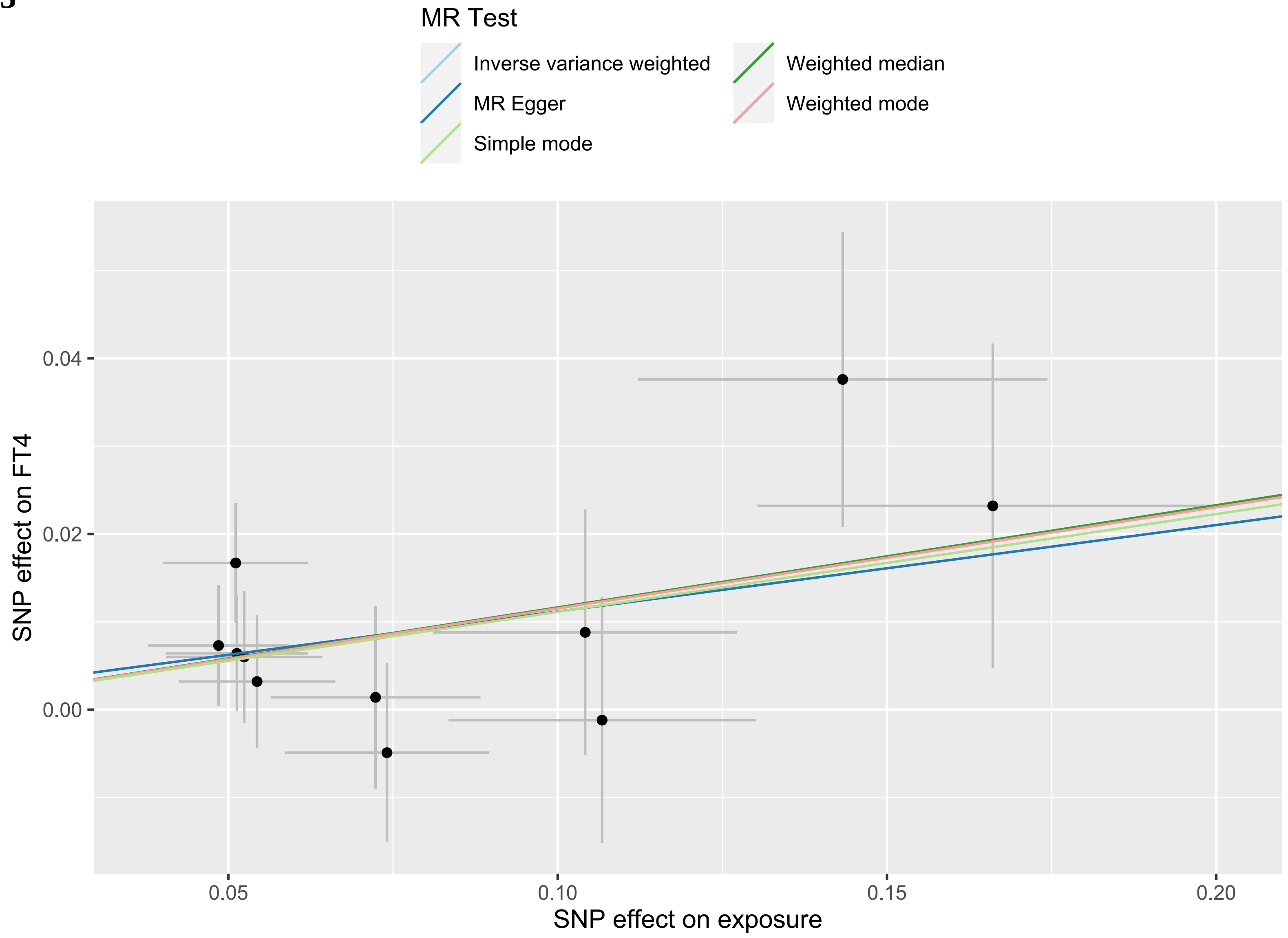

MR Test
Inverse variance weighted
MR Egger
Simple mode
Weighted median
Weighted mode
SNP effect on FT4
0.04
0.02
0.00
0.05
0.10
0.15
0.20
SNP effect on exposure

4

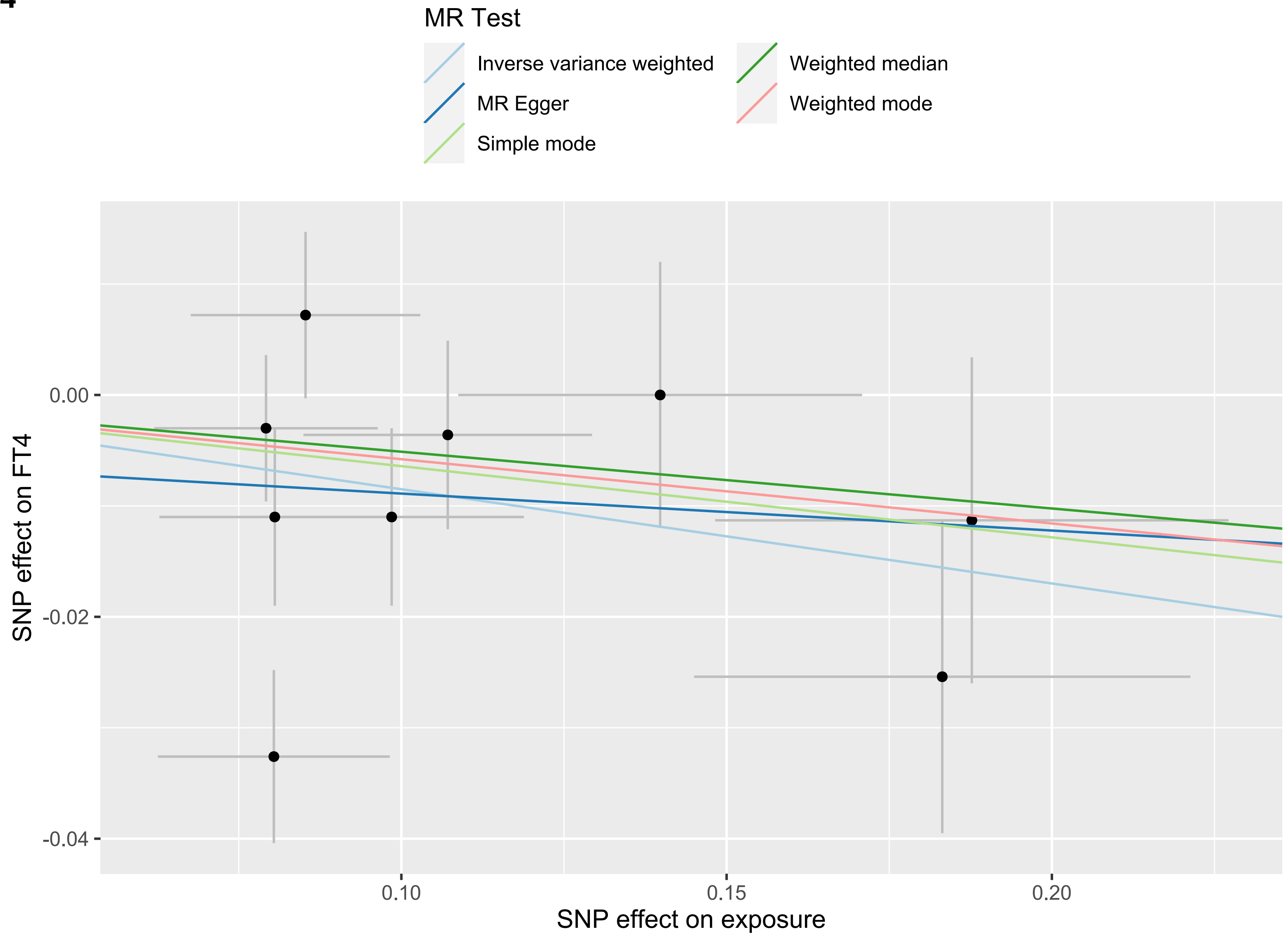
MR Test
Inverse variance weighted
MR Egger
Simple mode
Weighted median
Weighted mode
0.00
-0.02
-0.04
SNP effect on FT4
0.10
0.15
0.20
SNP effect on exposure

5

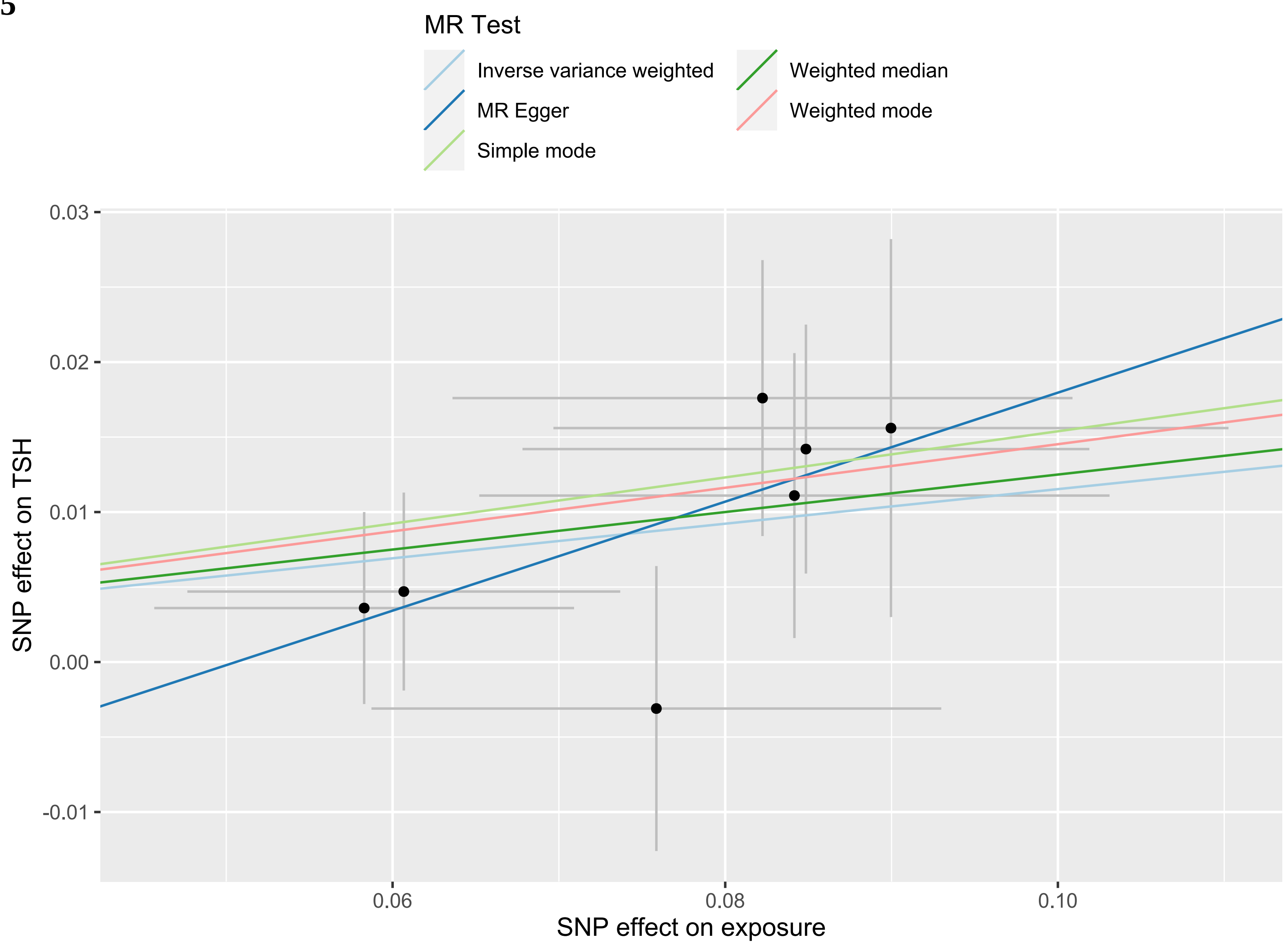

MR Test
Inverse variance weighted
MR Egger
Simple mode
Weighted median
Weighted mode
0.03
0.02
0.01
0.00
-0.01
SNP effect on TSH
0.06
0.08
0.10
SNP effect on exposure

6

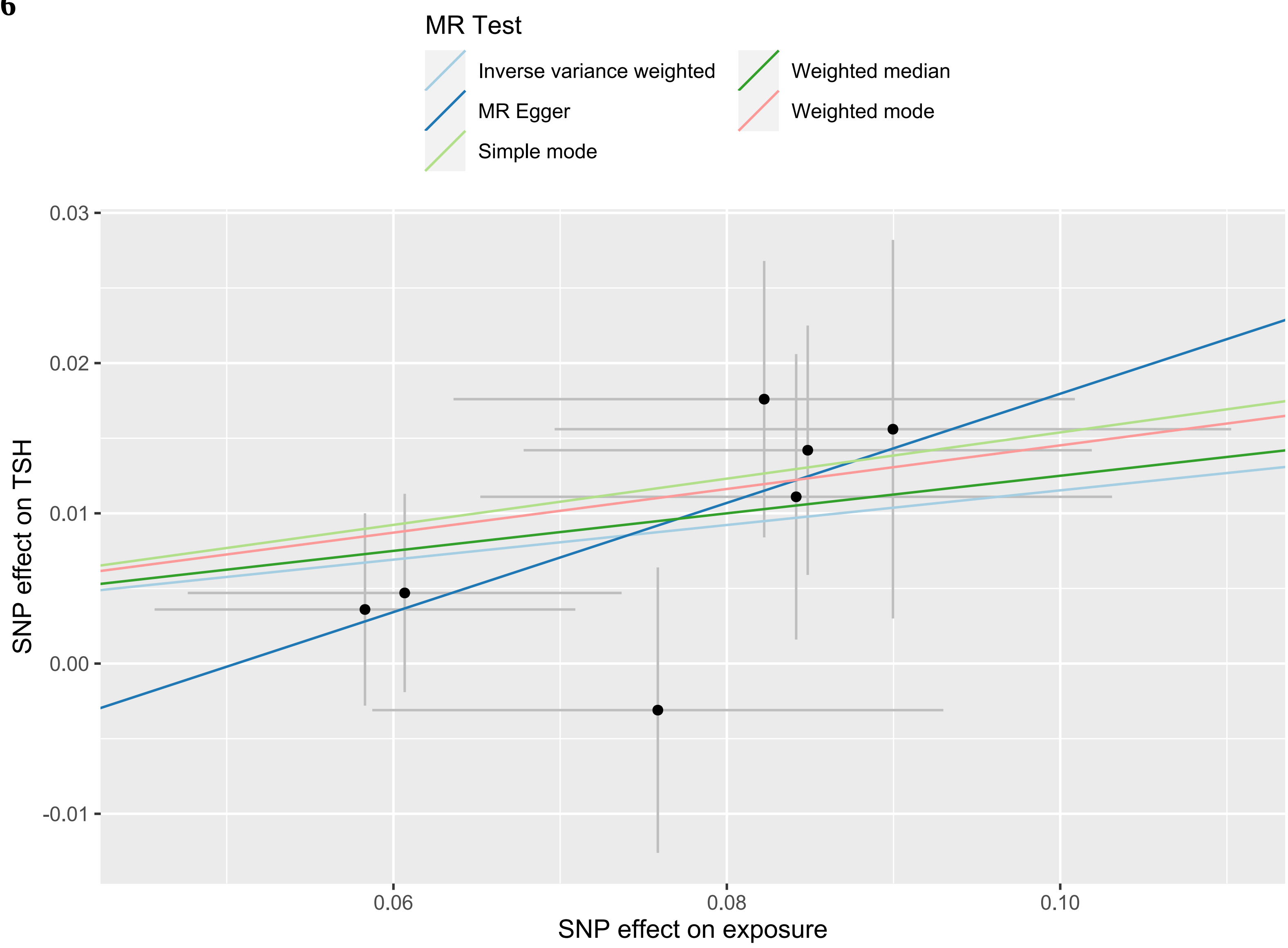

MR Test
Inverse variance weighted
MR Egger
Simple mode
Weighted median
Weighted mode
0.03
0.02
0.01
0.00
-0.01
SNP effect on TSH
0.06
0.08
0.10
SNP effect on exposure

7

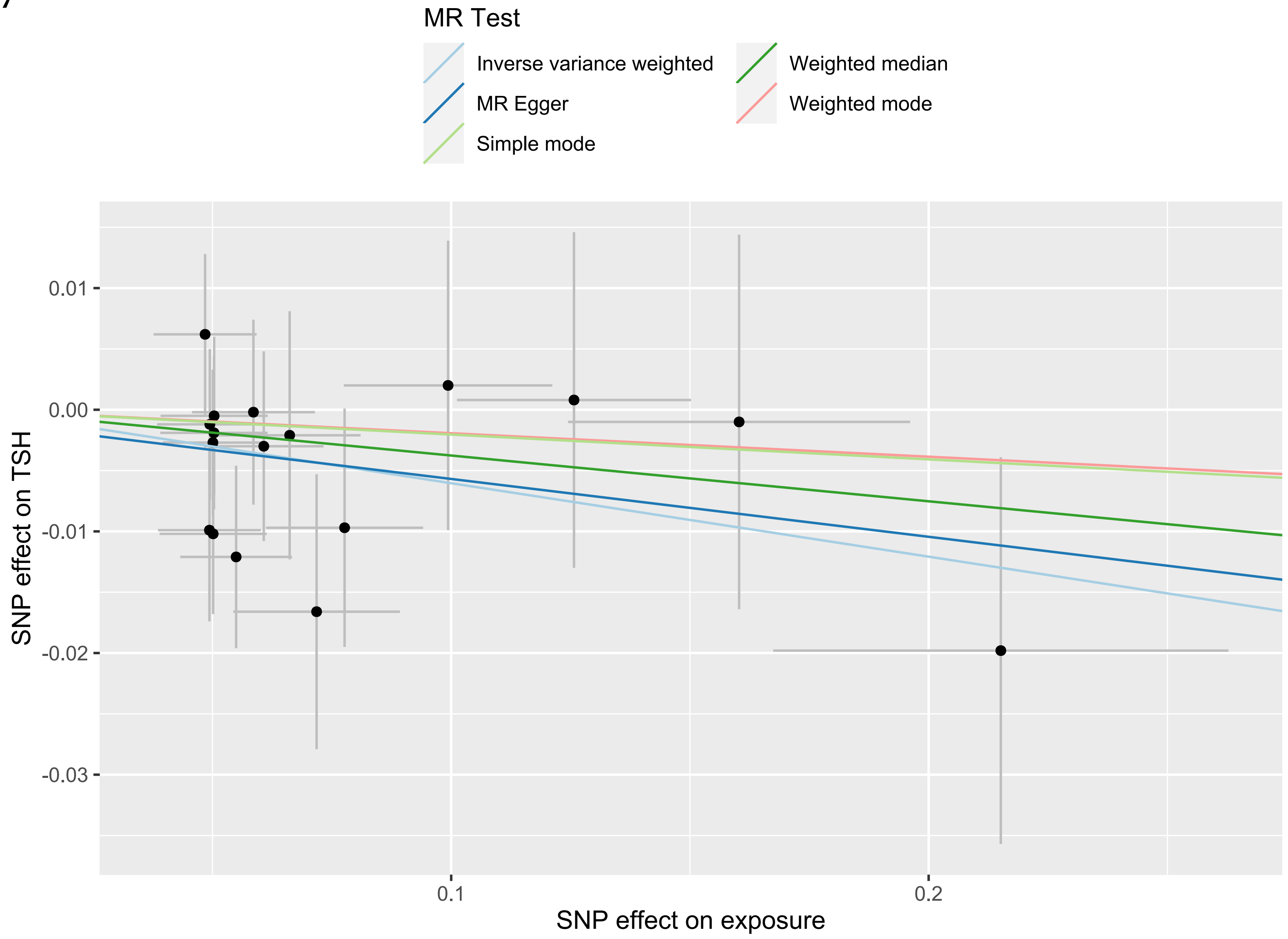

MR Test
Inverse variance weighted
MR Egger
Simple mode
Weighted median
Weighted mode
0.01
0.00
-0.01
-0.02
-0.03
SNP effect on TSH
0.1
0.2
SNP effect on exposure

8

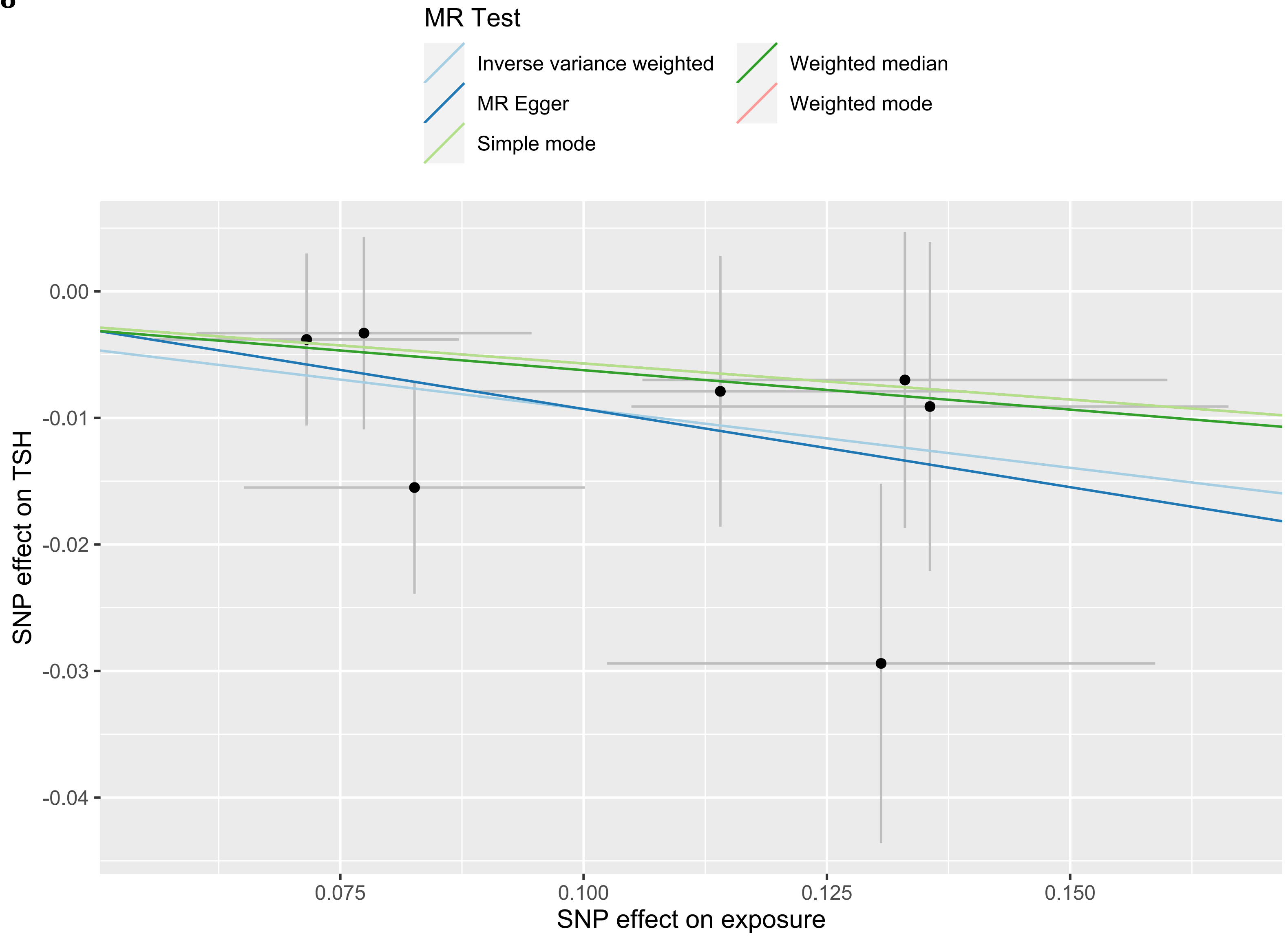

MR Test
Inverse variance weighted
MR Egger
Simple mode
Weighted median
Weighted mode
0.00
-0.01
-0.02
-0.03
-0.04
SNP effect on TSH
0.075
0.100
0.125
0.150
SNP effect on exposure

9

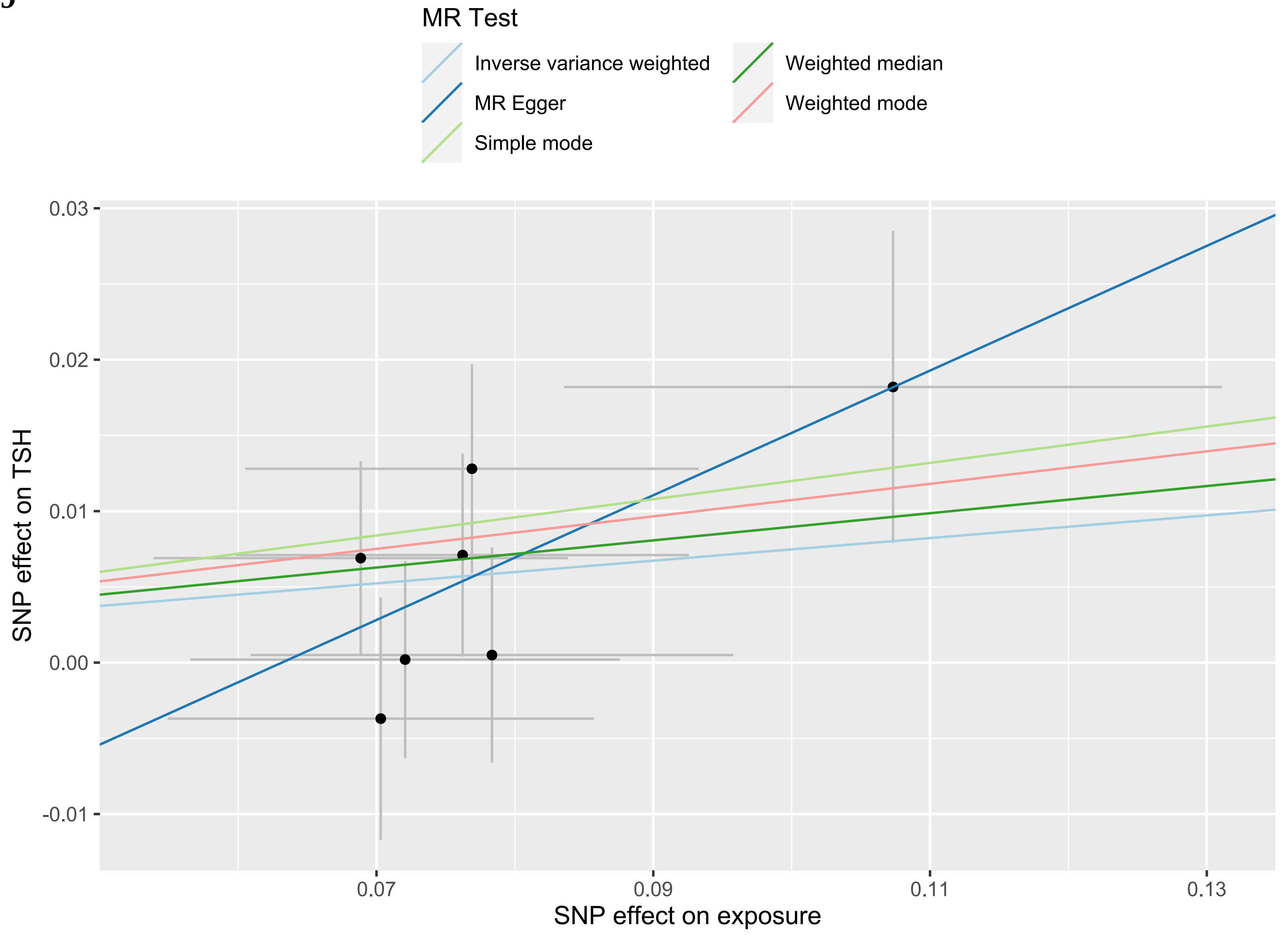
MR Test
Inverse variance weighted
MR Egger
Simple mode
Weighted median
Weighted mode
0.03
0.02
0.01
0.00
-0.01
SNP effect on TSH
0.07
0.09
0.11
0.13
SNP effect on exposure

10

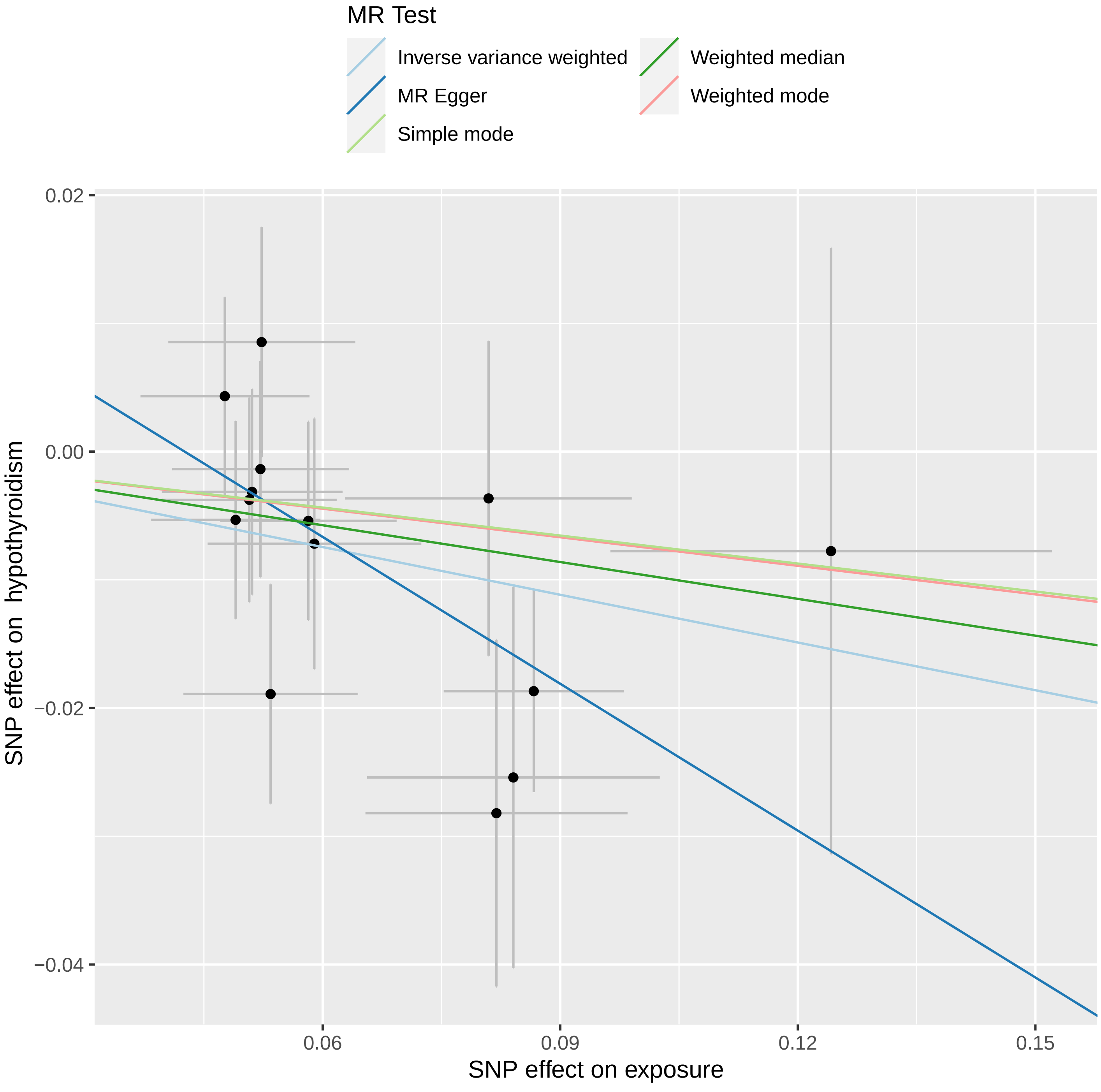

MR Test
Inverse variance weighted
MR Egger
Simple mode
Weighted median
Weighted mode
0.02
0.00
−0.02
−0.04
SNP effect on hypothyroidism
0.06
0.09
0.12
0.15
SNP effect on exposure

11

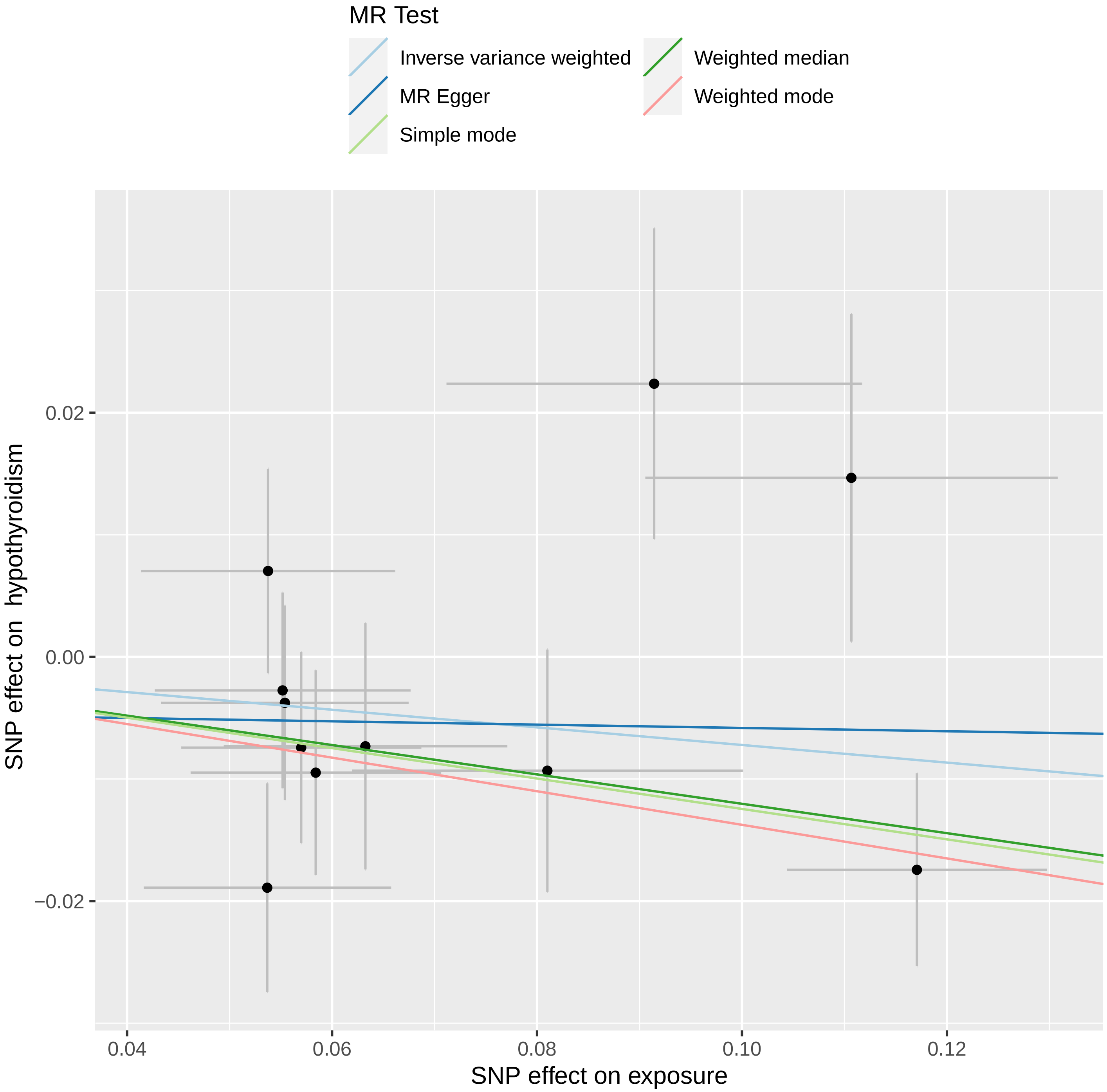
MR Test
Inverse variance weighted
MR Egger
Simple mode
Weighted median
Weighted mode
0.02
0.00
−0.02
SNP effect on hypothyroidism
0.04
0.06
0.08
0.10
0.12
SNP effect on exposure

12

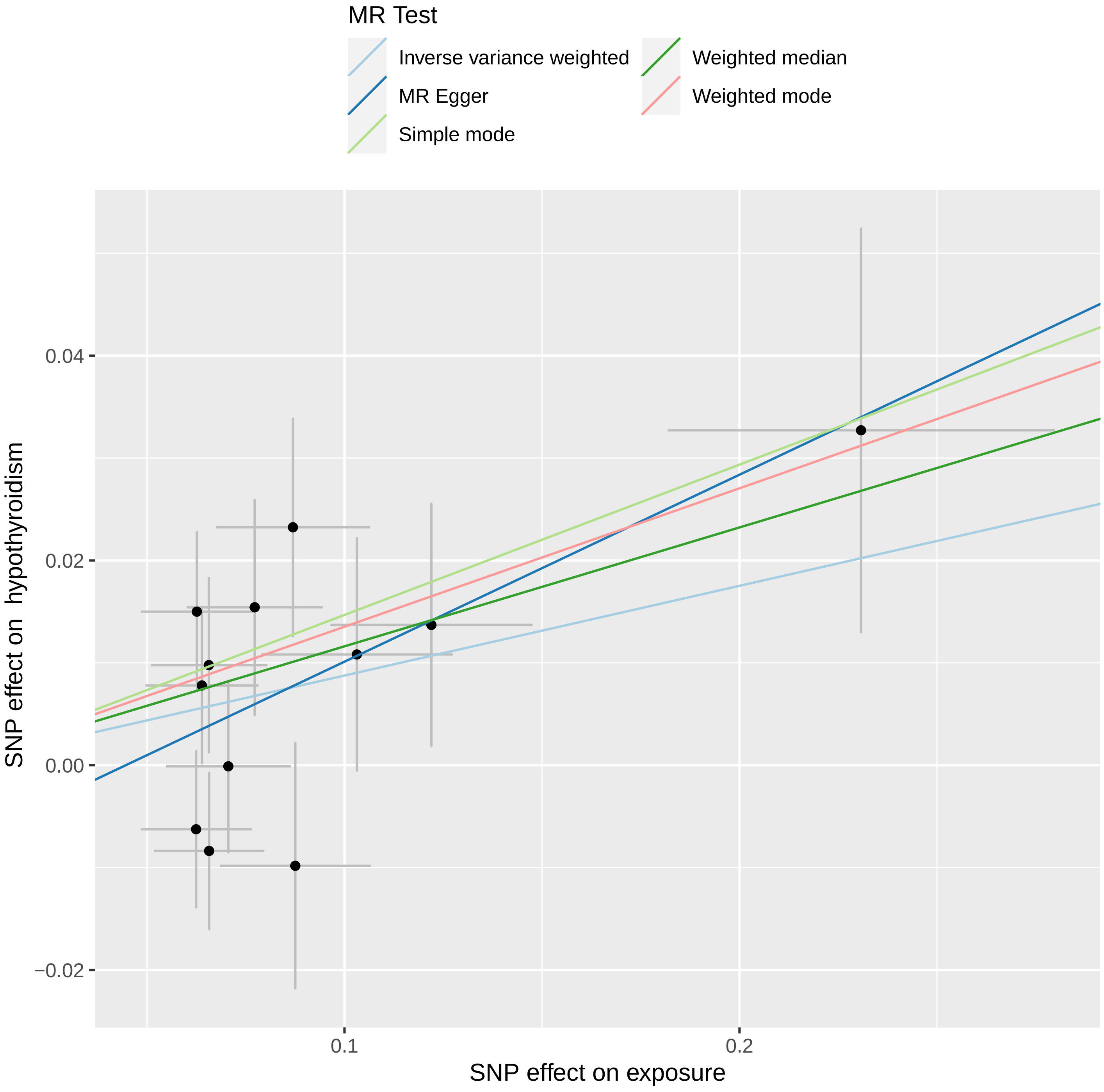

MR Test
Inverse variance weighted
MR Egger
Simple mode
Weighted median
Weighted mode
0.04
0.02
0.00
−0.02
0.1
0.2
SNP effect on hypothyroidism
SNP effect on exposure

13

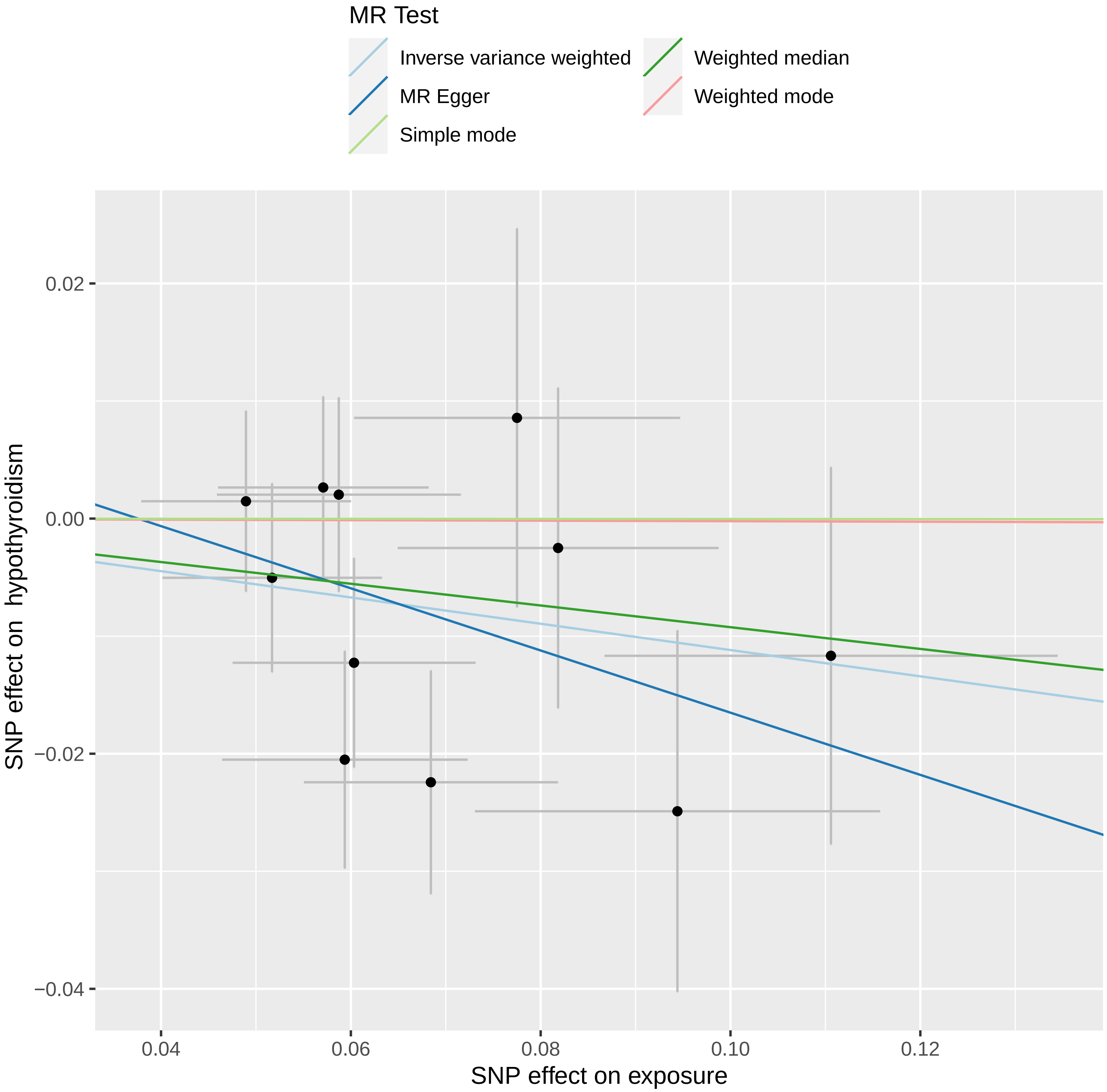
MR Test
Inverse variance weighted
MR Egger
Simple mode
Weighted median
Weighted mode
0.02
0.00
−0.02
−0.04
0.04
0.06
0.08
0.10
0.12
SNP effect on exposure
SNP effect on hypothyroidism

14

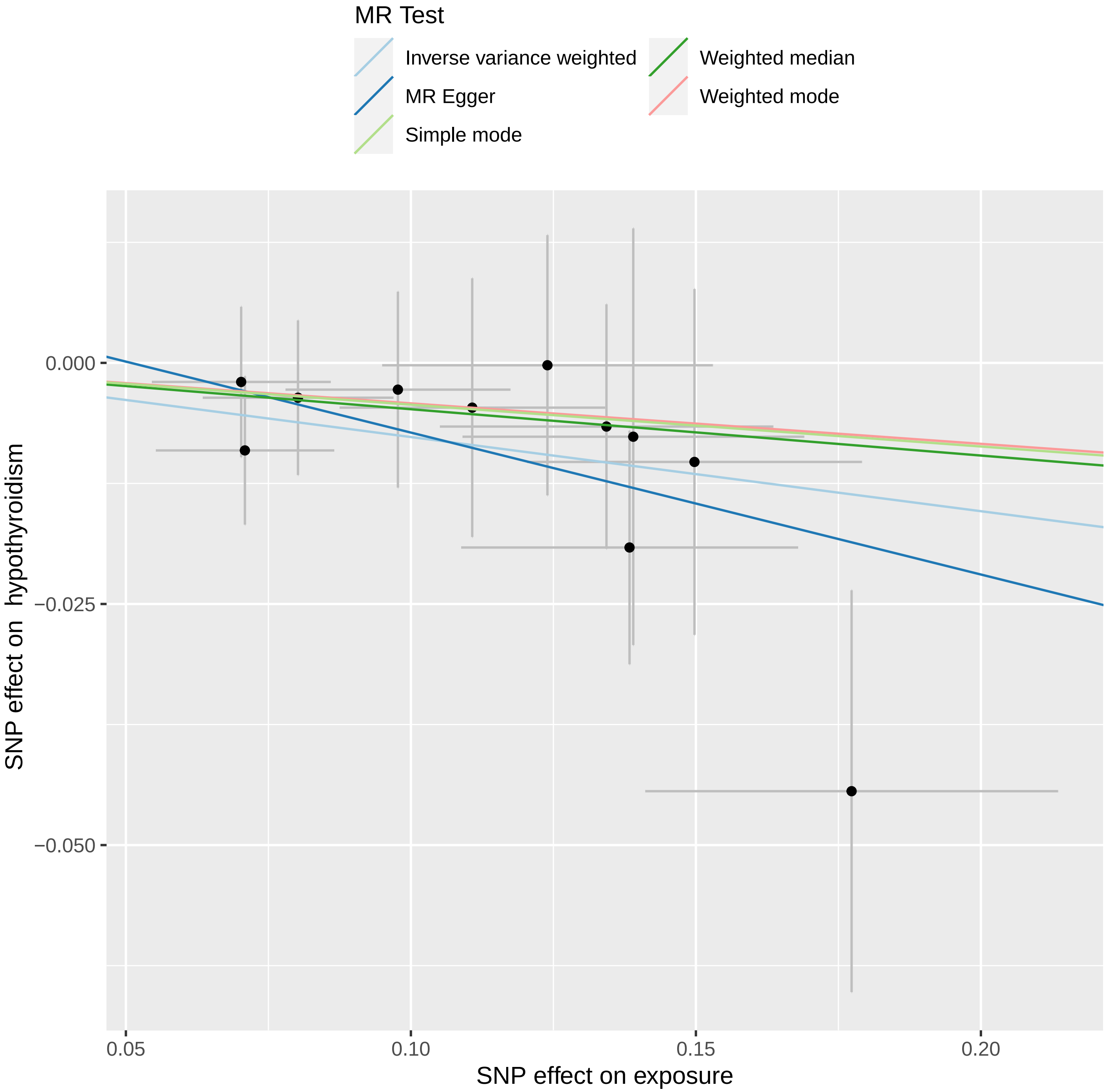

MR Test
Inverse variance weighted
MR Egger
Simple mode
Weighted median
Weighted mode
0.000
−0.025
−0.050
0.05
0.10
0.15
0.20
SNP effect on hypothyroidism
SNP effect on exposure

15

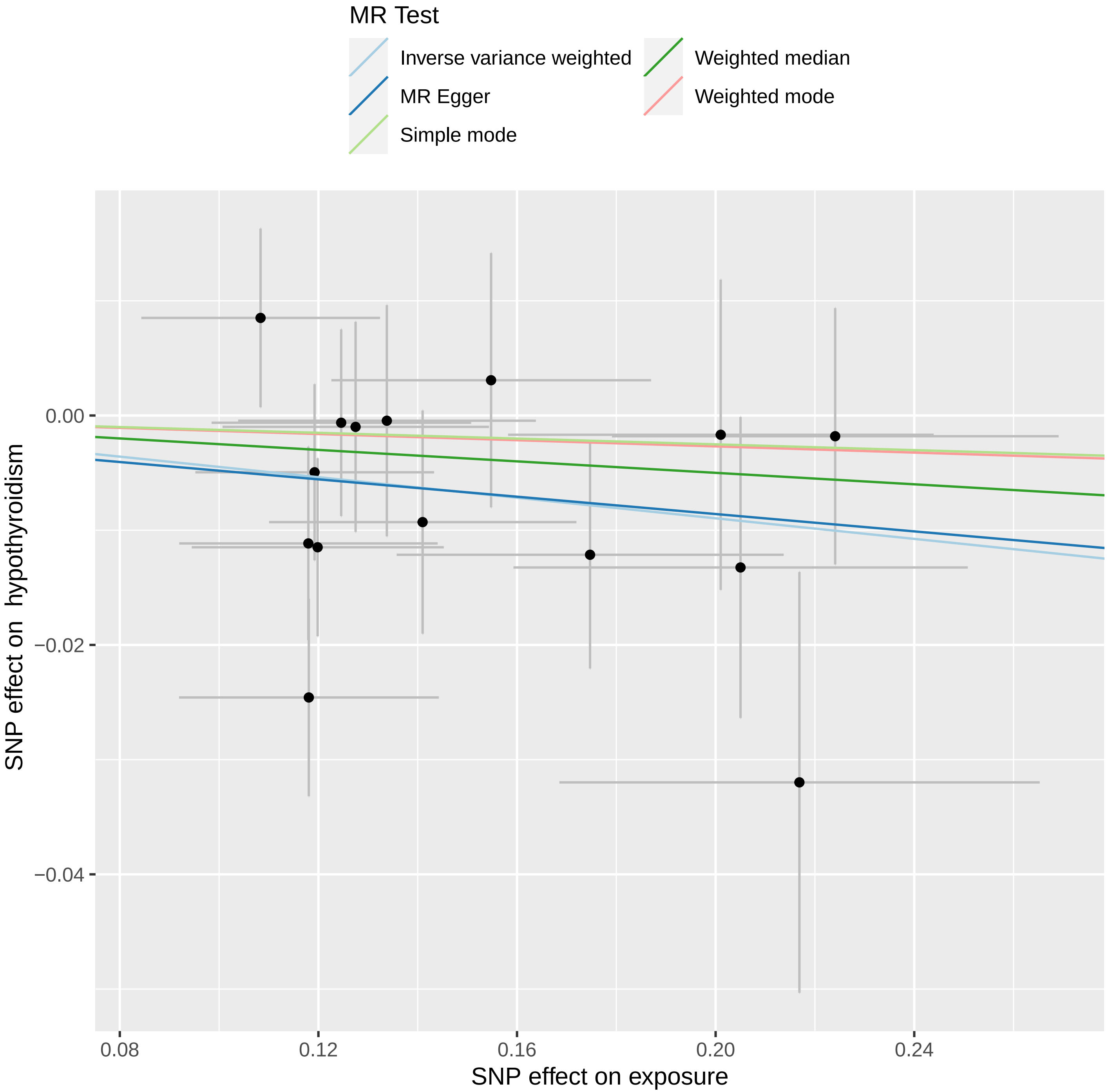

MR Test
Inverse variance weighted
MR Egger
Simple mode
Weighted median
Weighted mode
0.00
−0.02
−0.04
SNP effect on hypothyroidism
0.08
0.12
0.16
0.20
0.24
SNP effect on exposure

16

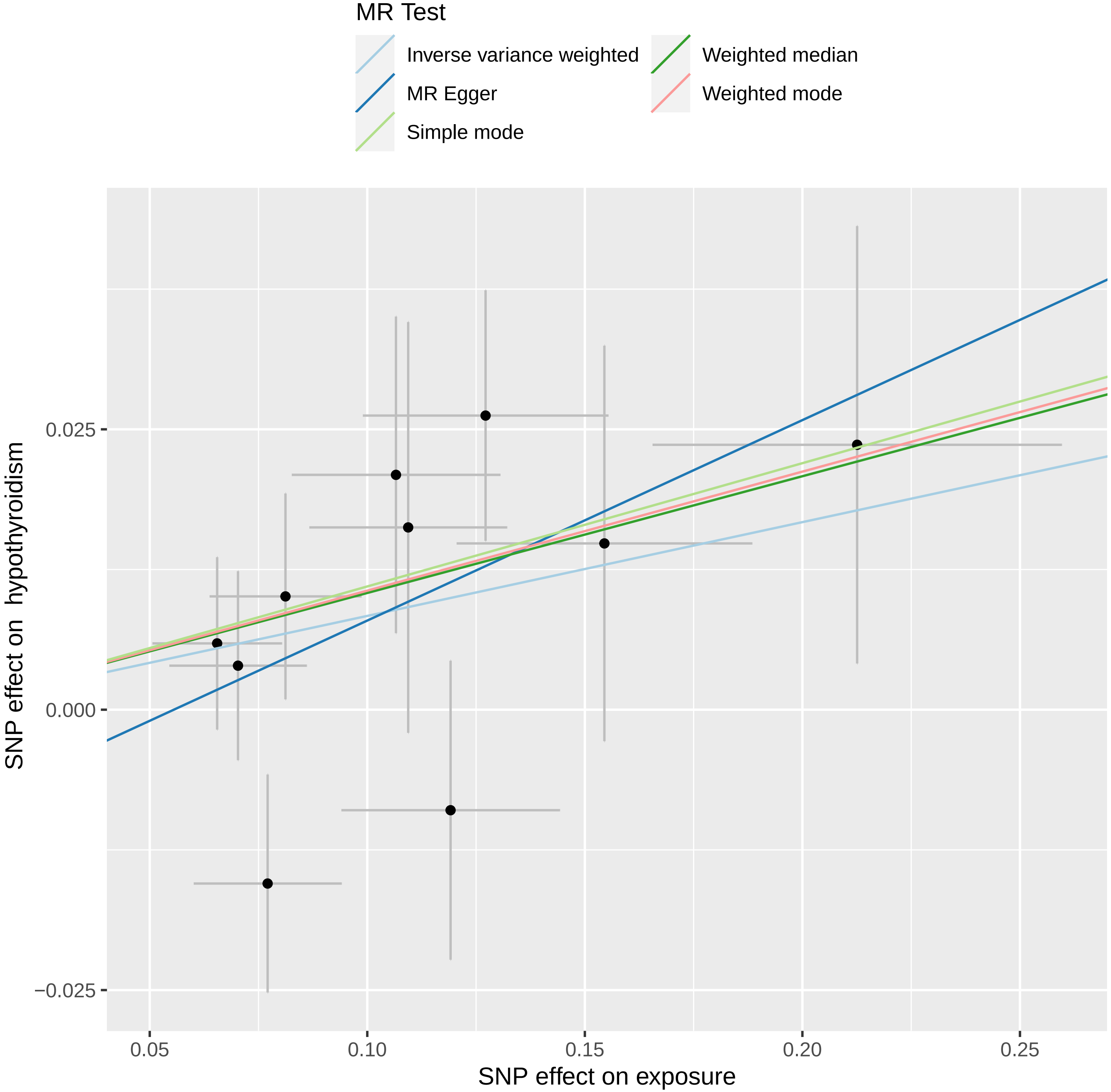

MR Test
Inverse variance weighted
MR Egger
Simple mode
Weighted median
Weighted mode
SNP effect on hypothyroidism
0.025
0.000
−0.025
0.05
0.10
0.15
0.20
0.25
SNP effect on exposure

17

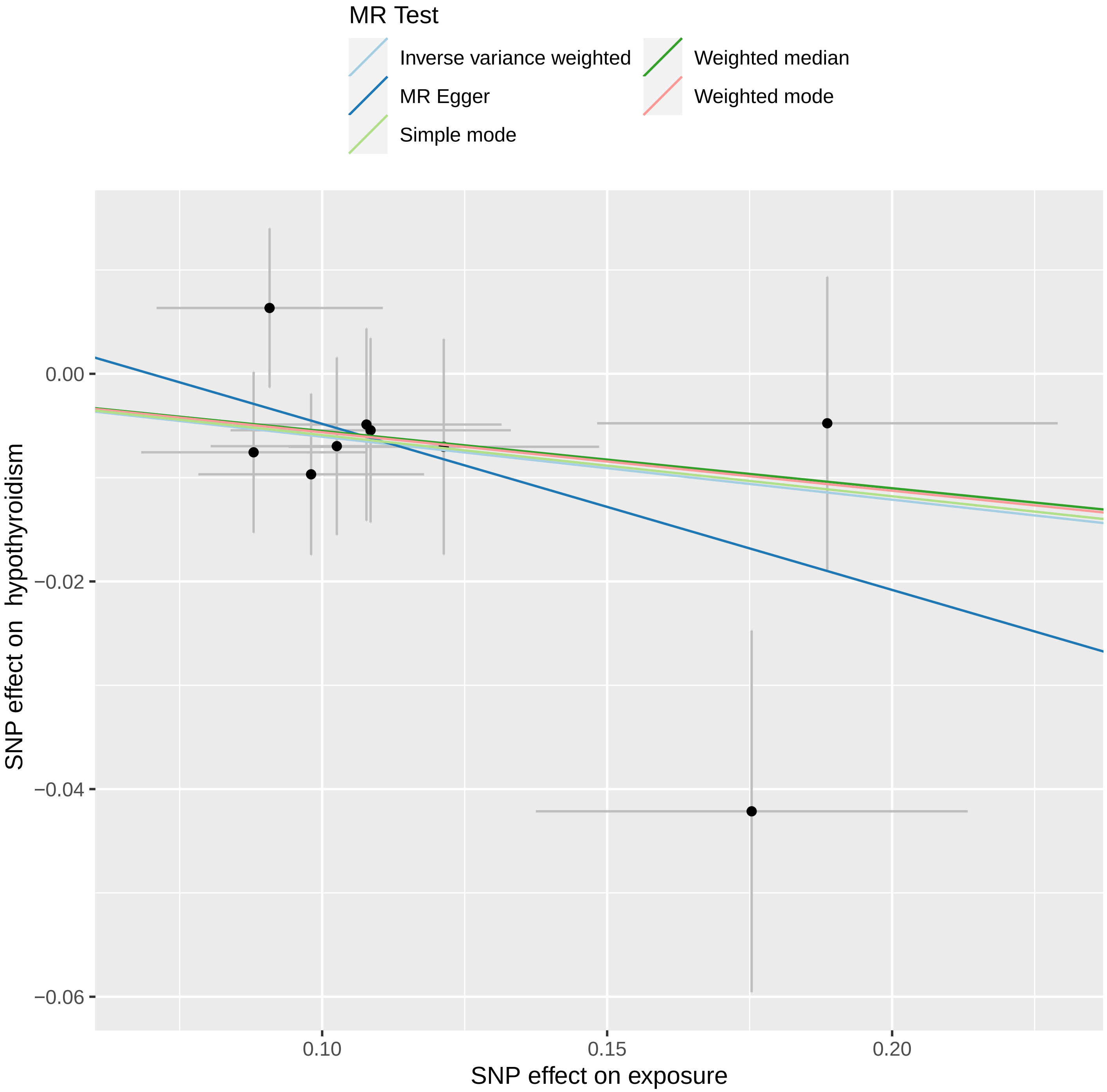
MR Test
Inverse variance weighted
MR Egger
Simple mode
Weighted median
Weighted mode
SNP effect on hypothyroidism
0.00
−0.02
−0.04
−0.06
0.10
0.15
0.20
SNP effect on exposure

18

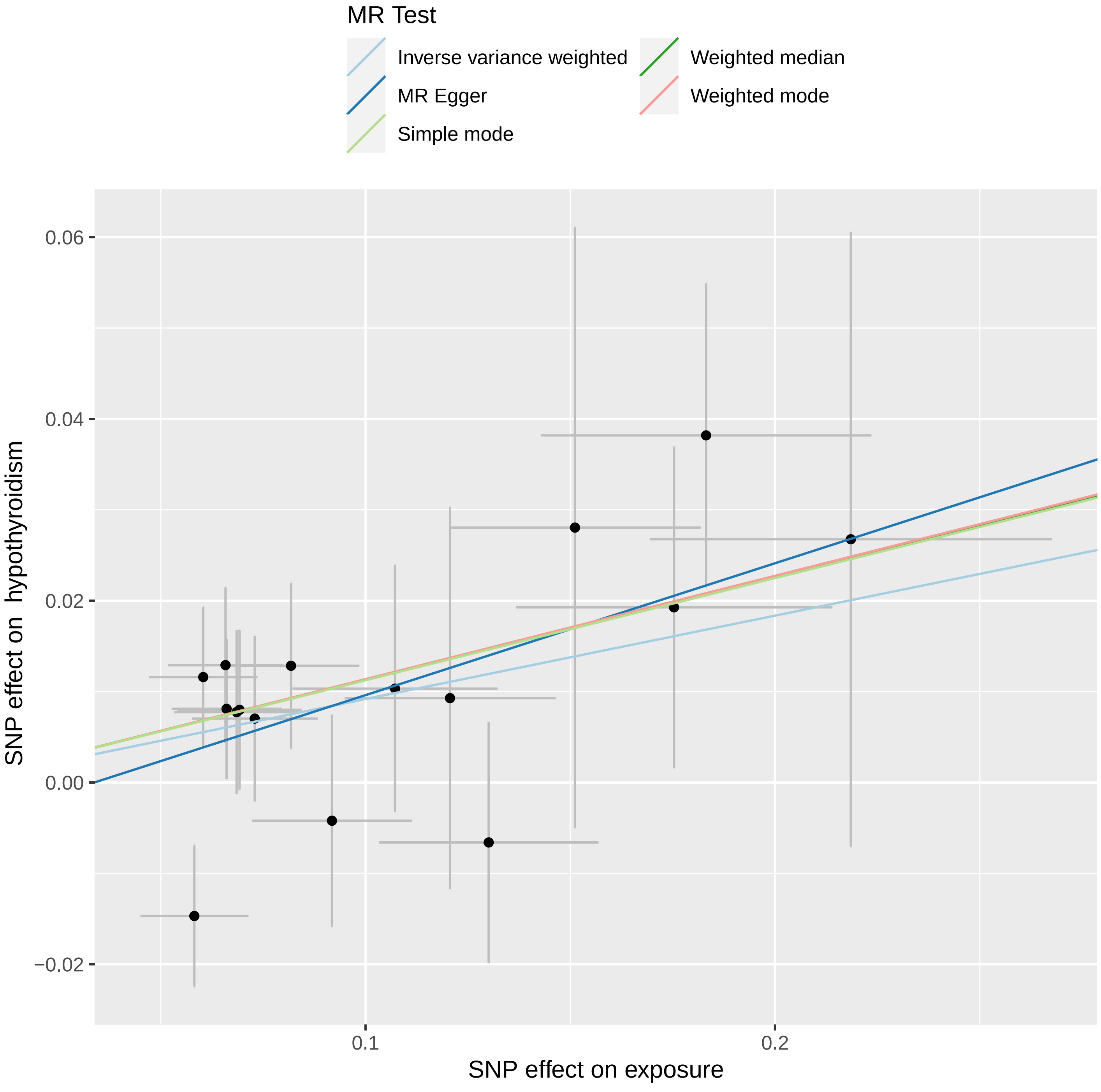

MR Test
Inverse variance weighted
MR Egger
Simple mode
Weighted median
Weighted mode
0.06
0.04
0.02
0.00
−0.02
0.1
0.2
SNP effect on hypothyroidism
SNP effect on exposure

19

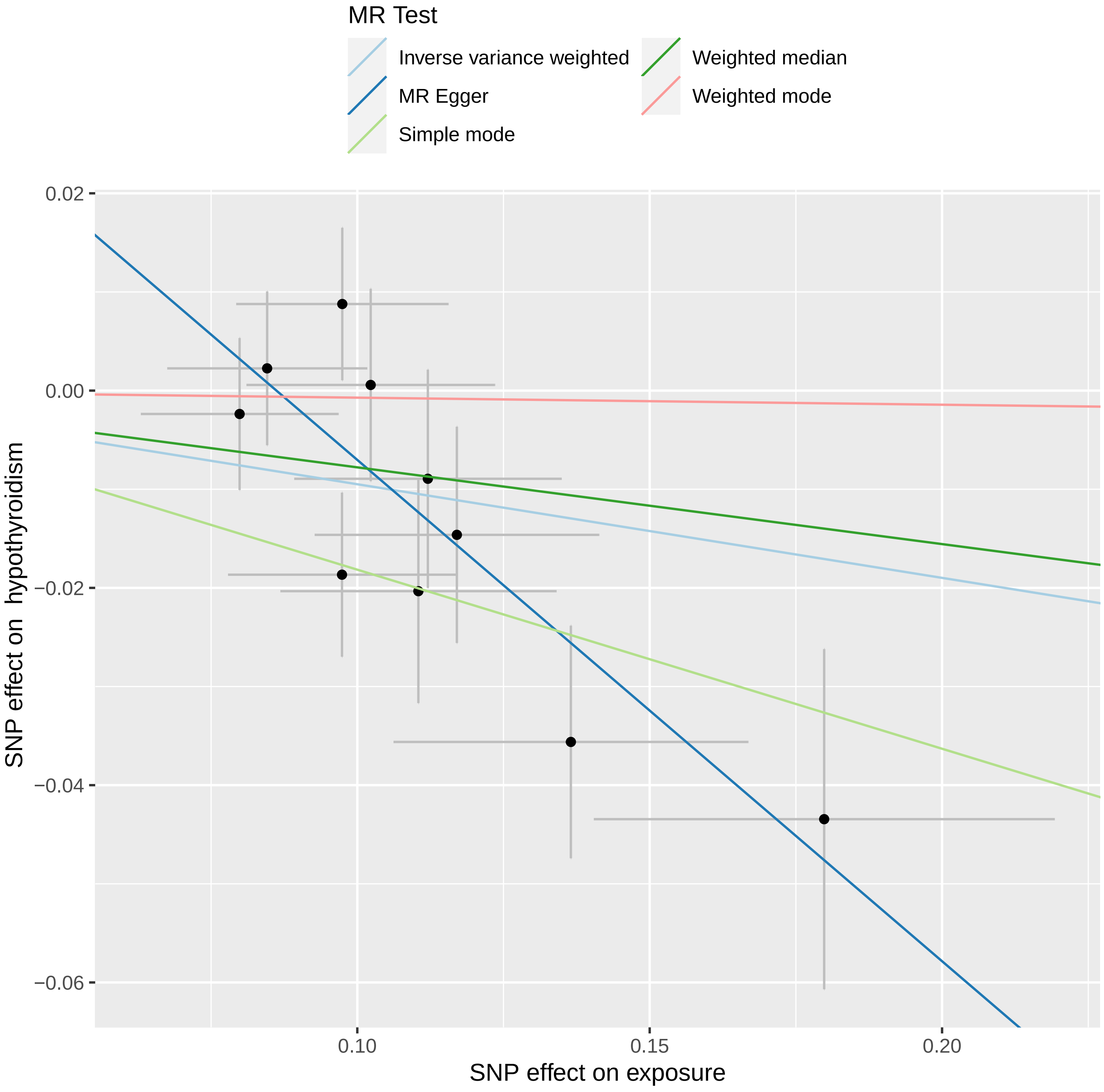
MR Test
Inverse variance weighted
MR Egger
Simple mode
Weighted median
Weighted mode
0.02
0.00
−0.02
−0.04
−0.06
SNP effect on hypothyroidism
0.10
0.15
0.20
SNP effect on exposure

20

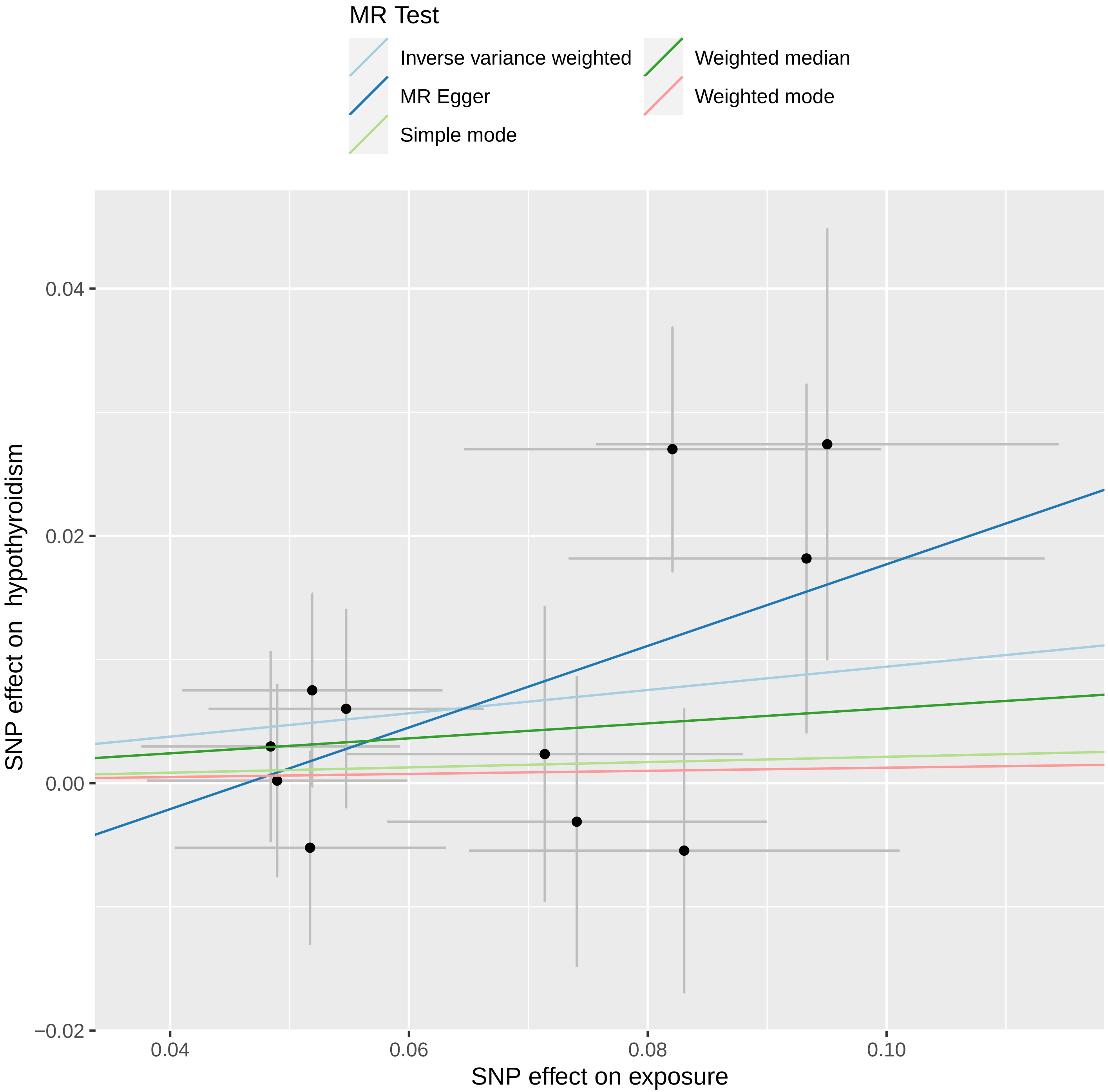
MR Test
Inverse variance weighted
MR Egger
Simple mode
Weighted median
Weighted mode
0.04
0.02
0.00
−0.02
SNP effect on hypothyroidism
0.04
0.06
0.08
0.10
SNP effect on exposure

21

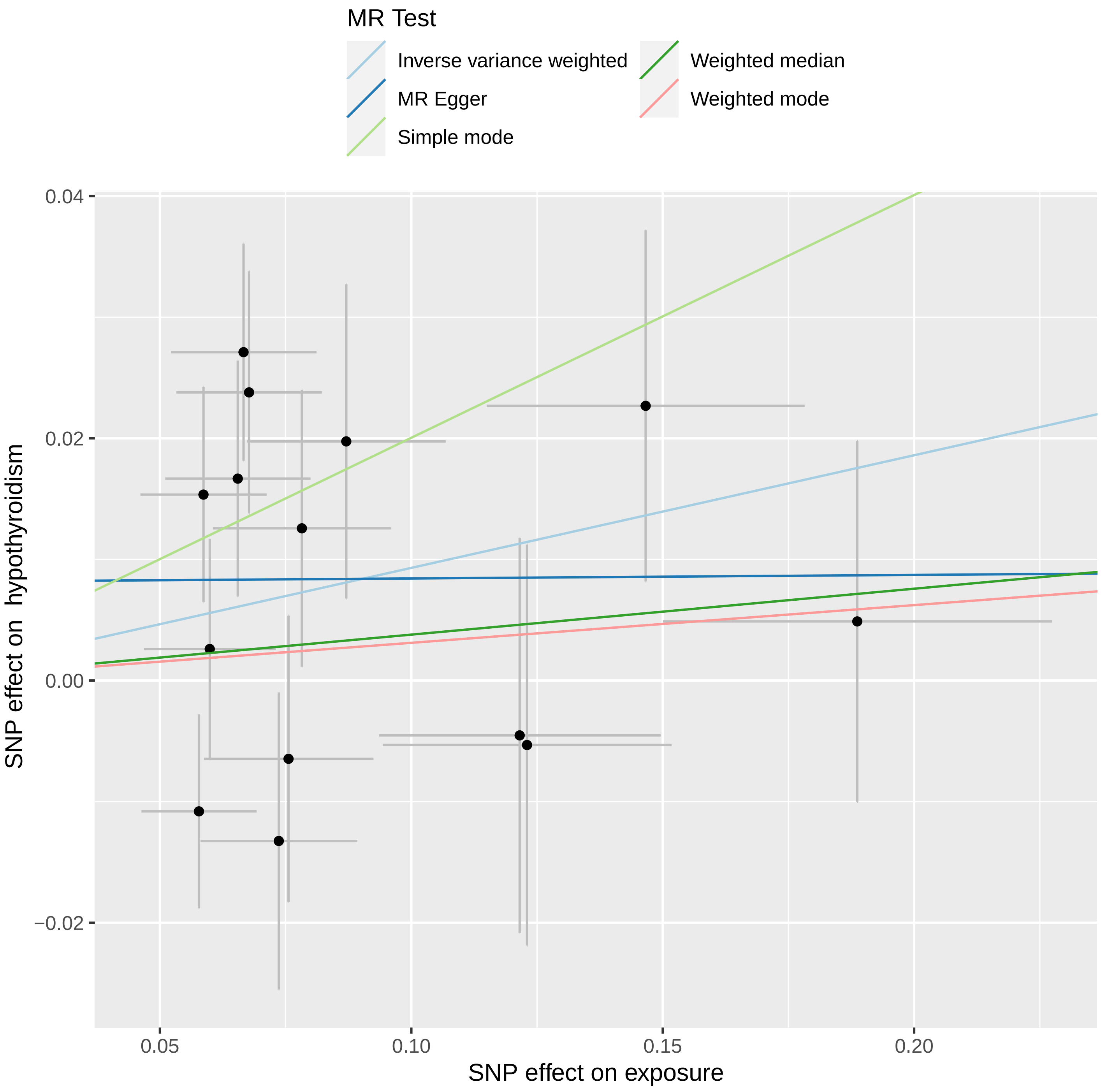

MR Test
Inverse variance weighted
MR Egger
Simple mode
Weighted median
Weighted mode
0.04
0.02
0.00
−0.02
0.05
0.10
0.15
0.20
SNP effect on hypothyroidism
SNP effect on exposure

22

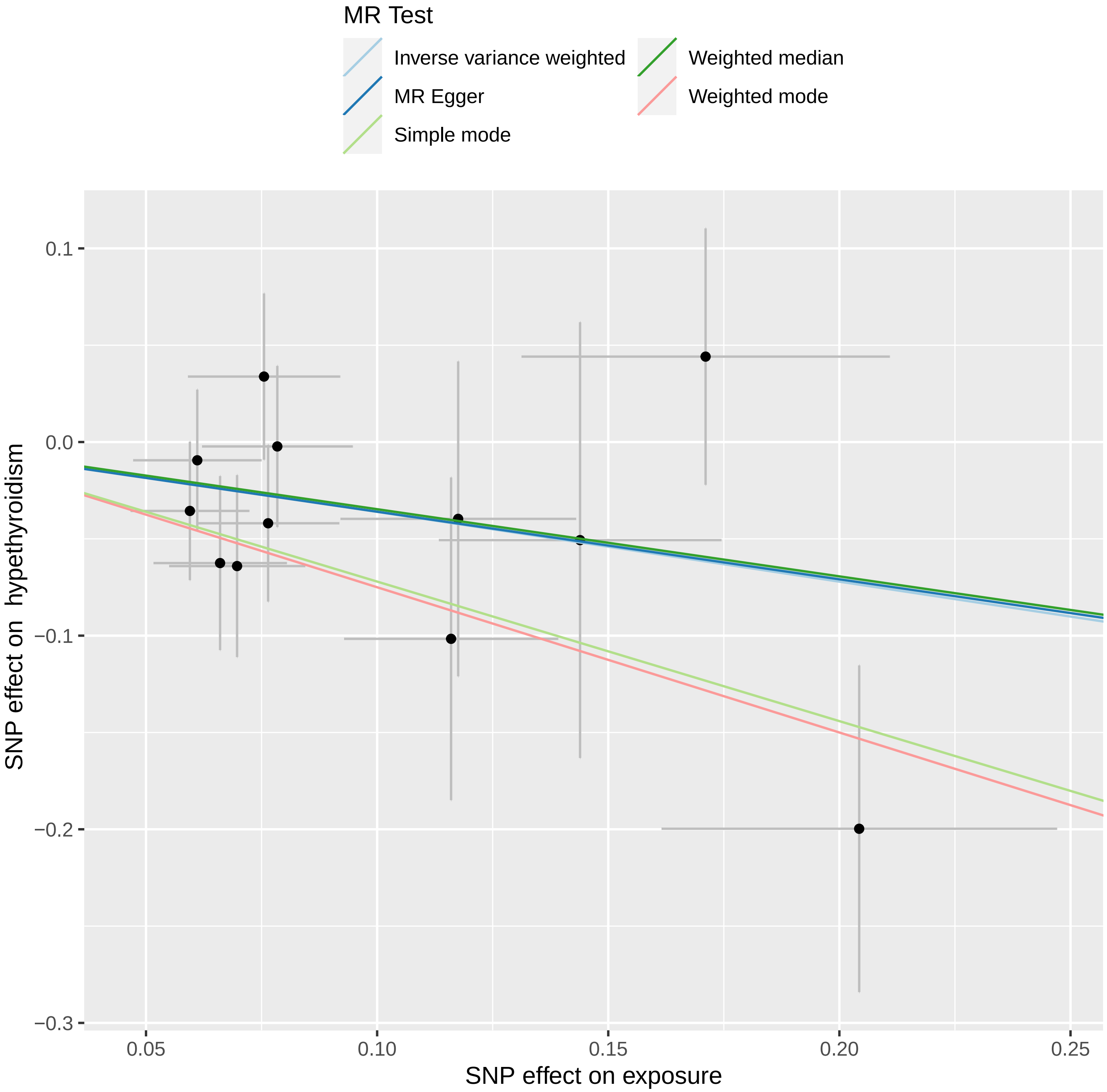

MR Test
Inverse variance weighted
MR Egger
Simple mode
Weighted median
Weighted mode
0.1
0.0
−0.1
−0.2
−0.3
SNP effect on hypethyroidism
0.05
0.10
0.15
0.20
0.25
SNP effect on exposure

23

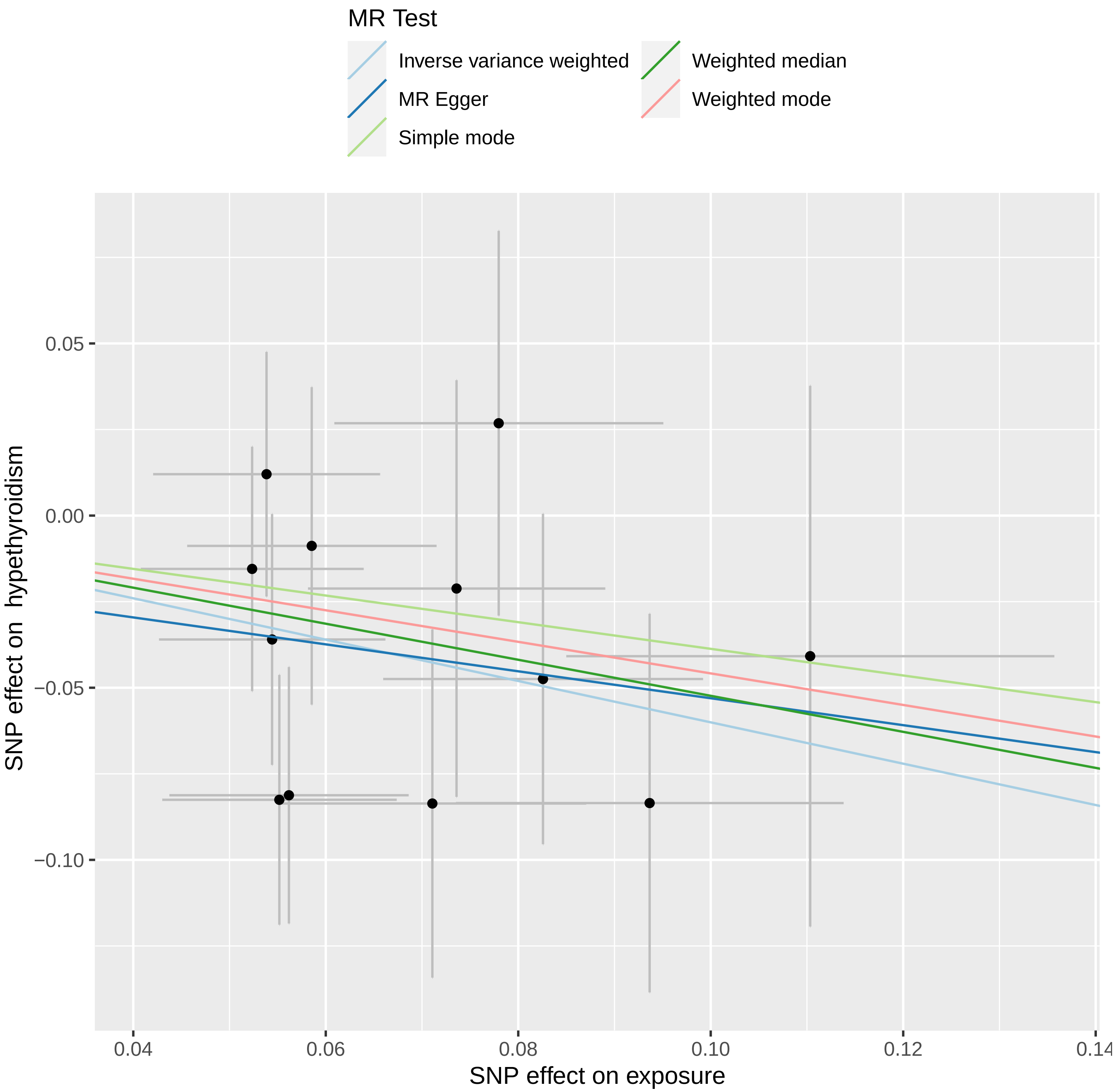
MR Test
Inverse variance weighted
MR Egger
Simple mode
Weighted median
Weighted mode
0.05
0.00
−0.05
−0.10
0.04
0.06
0.08
0.10
0.12
0.14
SNP effect on hypethyroidism
SNP effect on exposure

24

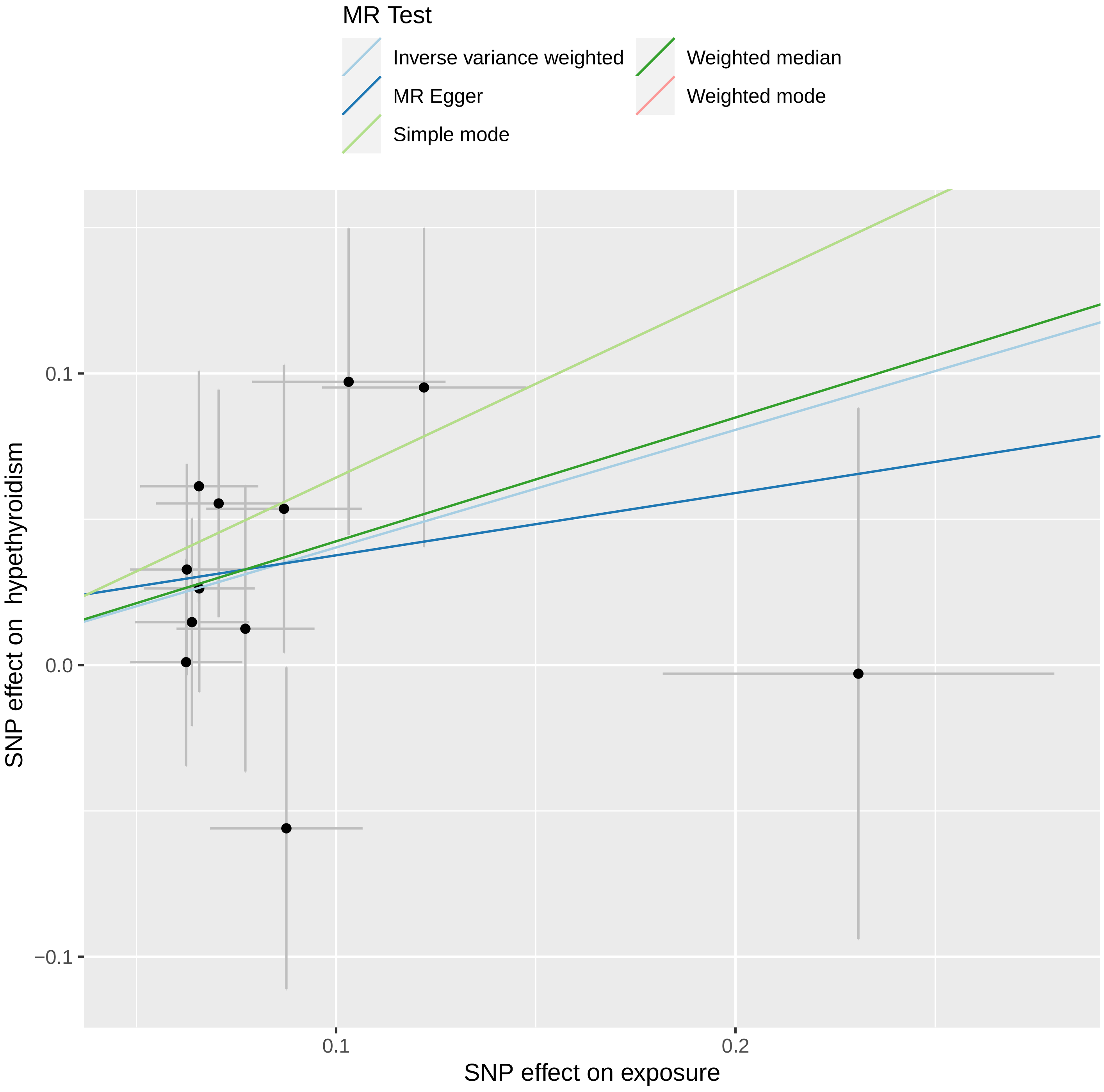

MR Test
Inverse variance weighted
MR Egger
Simple mode
Weighted median
Weighted mode
0.1
0.0
−0.1
0.1
0.2
SNP effect on hypethyroidism
SNP effect on exposure

25

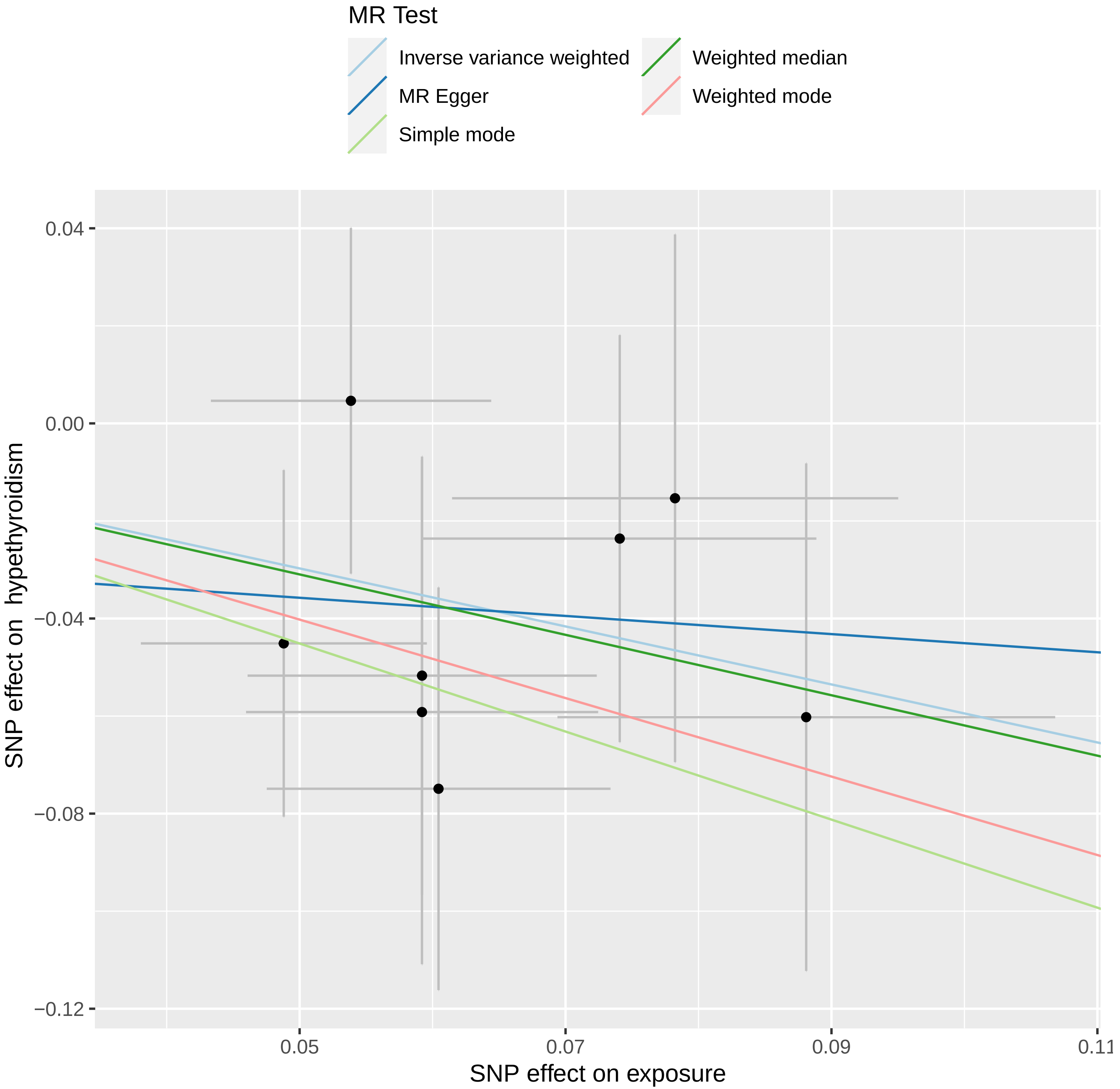
MR Test
Inverse variance weighted
MR Egger
Simple mode
Weighted median
Weighted mode
0.04
0.00
−0.04
−0.08
−0.12
0.05
0.07
0.09
0.11
SNP effect on exposure
SNP effect on hypethyroidism

26

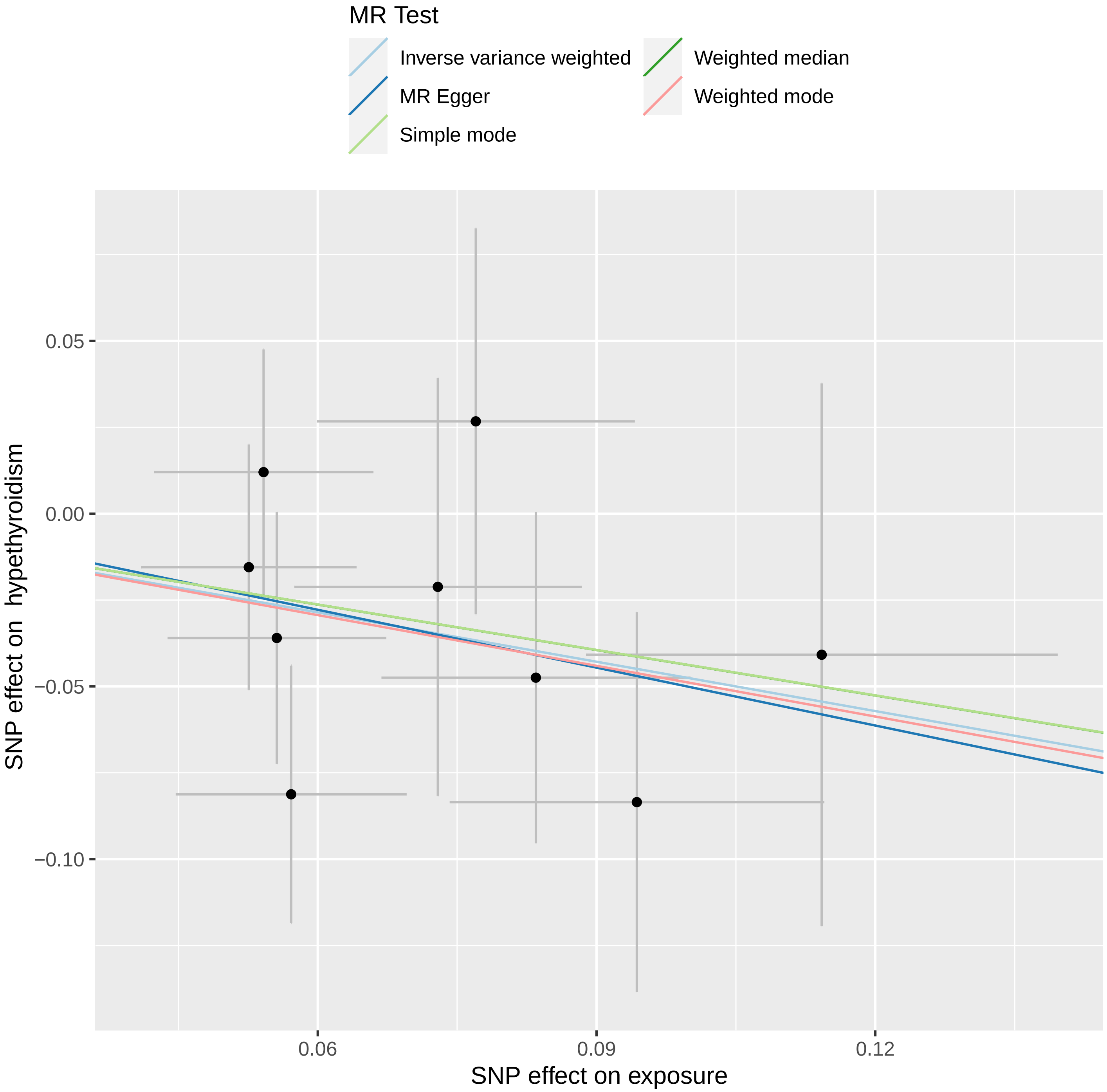

MR Test
Inverse variance weighted
MR Egger
Simple mode
Weighted median
Weighted mode
0.05
0.00
−0.05
−0.10
0.06
0.09
0.12
SNP effect on exposure
SNP effect on hypethyroidism

27

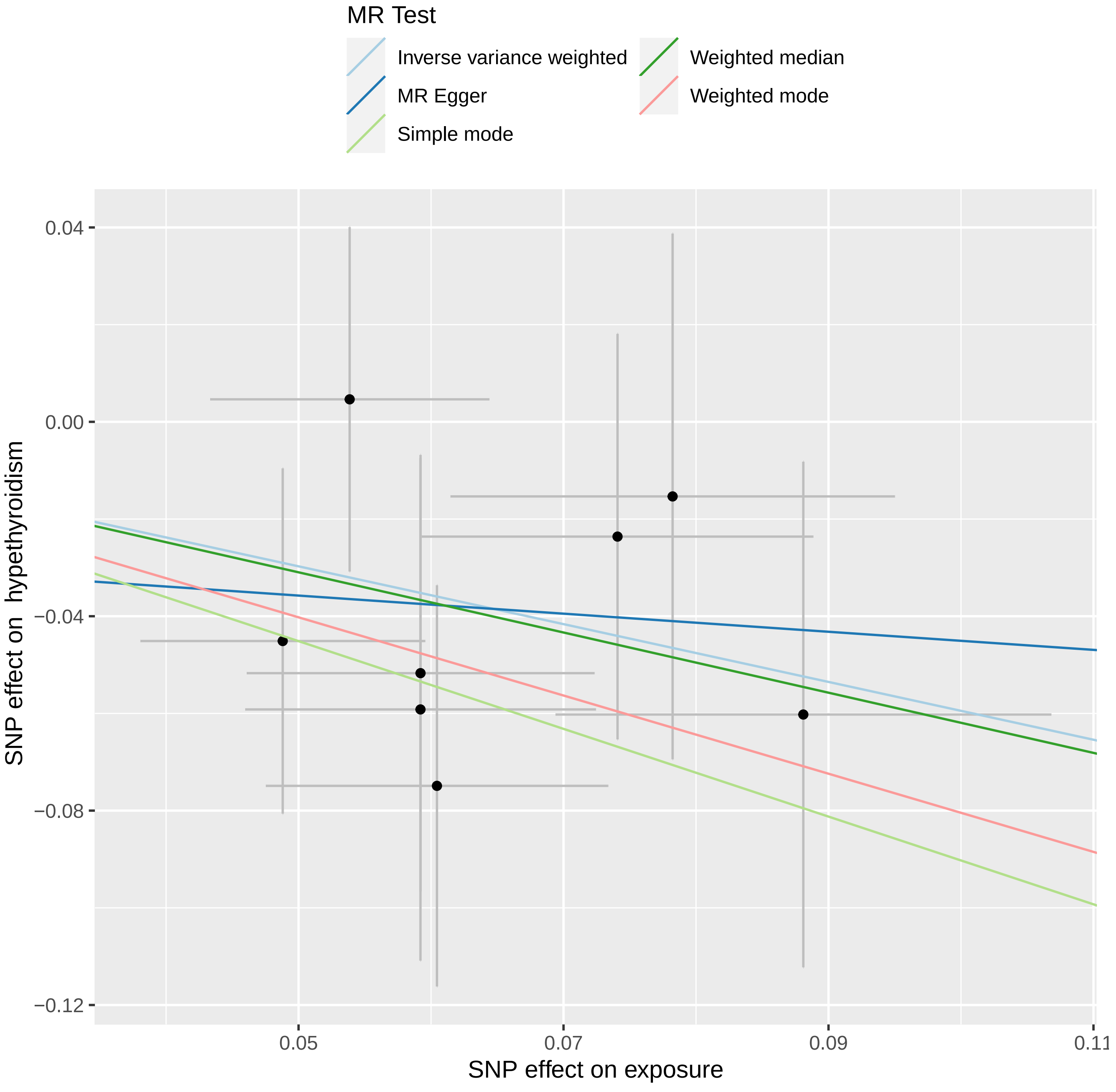
MR Test
Inverse variance weighted
MR Egger
Simple mode
Weighted median
Weighted mode
0.04
0.00
−0.04
−0.08
−0.12
0.05
0.07
0.09
0.11
SNP effect on hypethyroidism
SNP effect on exposure

28

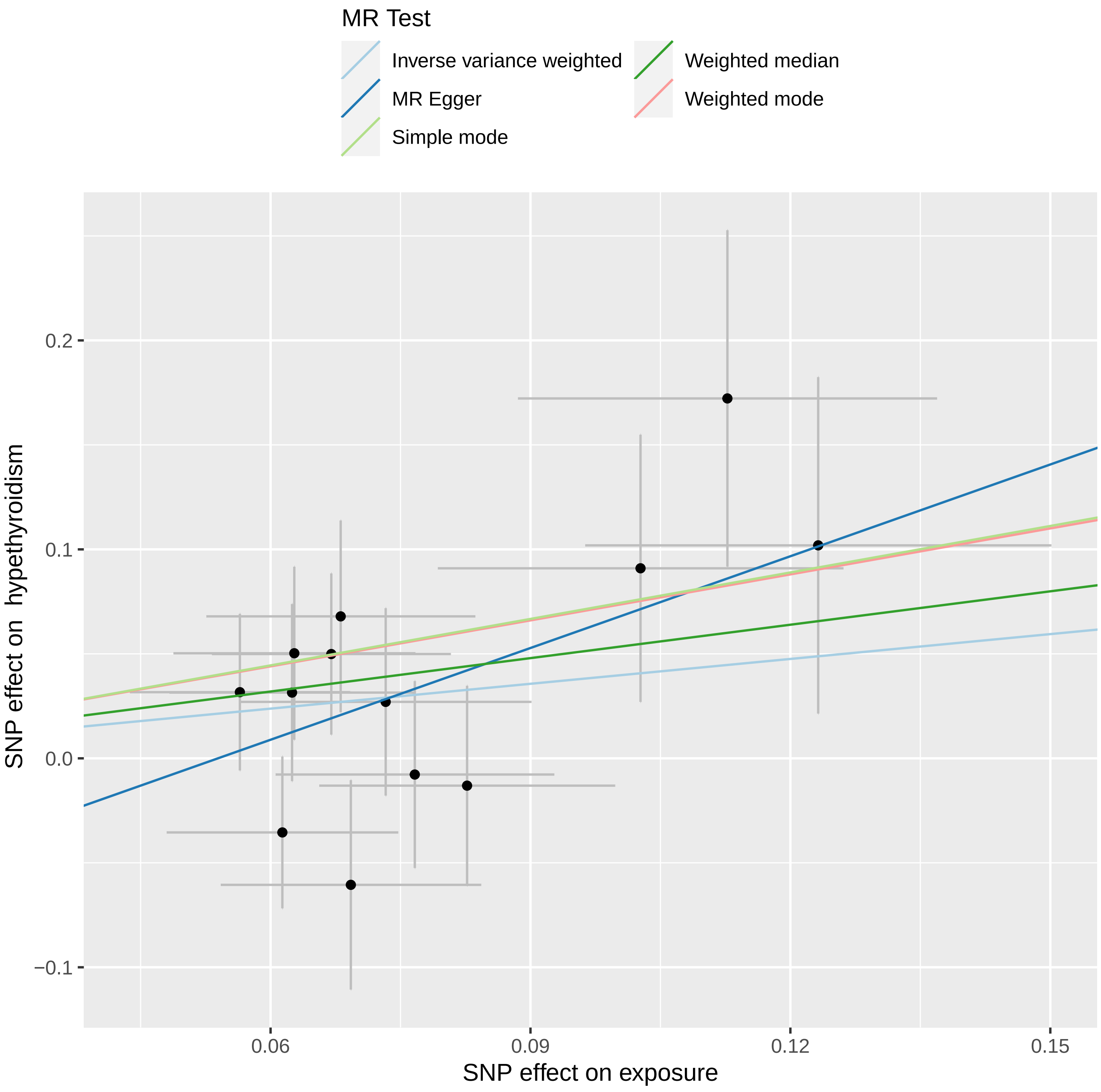
MR Test
Inverse variance weighted
MR Egger
Simple mode
Weighted median
Weighted mode
SNP effect on hypethyroidism
0.2
0.1
0.0
−0.1
0.06
0.09
0.12
0.15
SNP effect on exposure

29

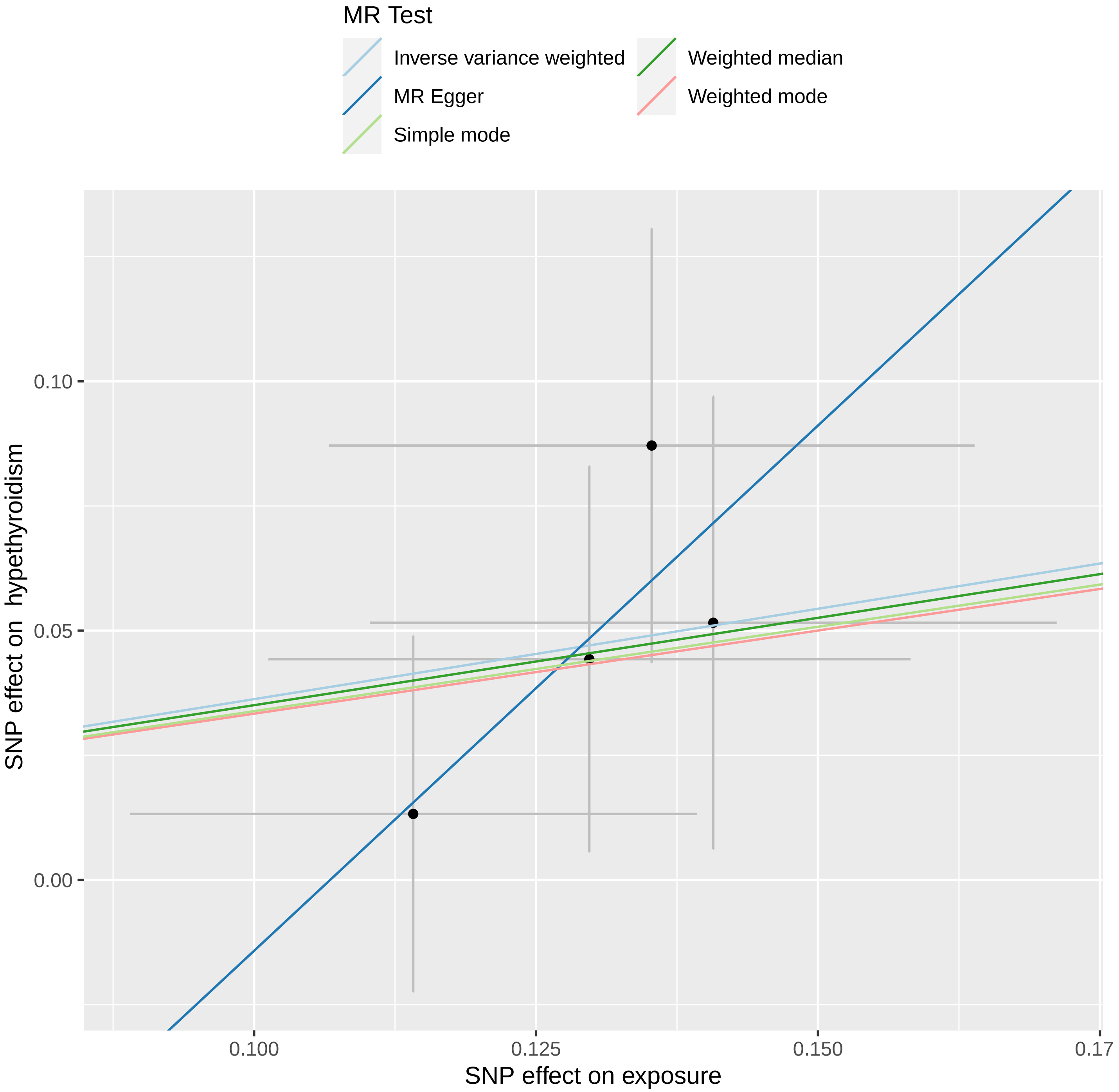
MR Test
Inverse variance weighted
MR Egger
Simple mode
Weighted median
Weighted mode
0.10
0.05
0.00
SNP effect on hypethyroidism
0.100
0.125
0.150
SNP effect on exposure

30

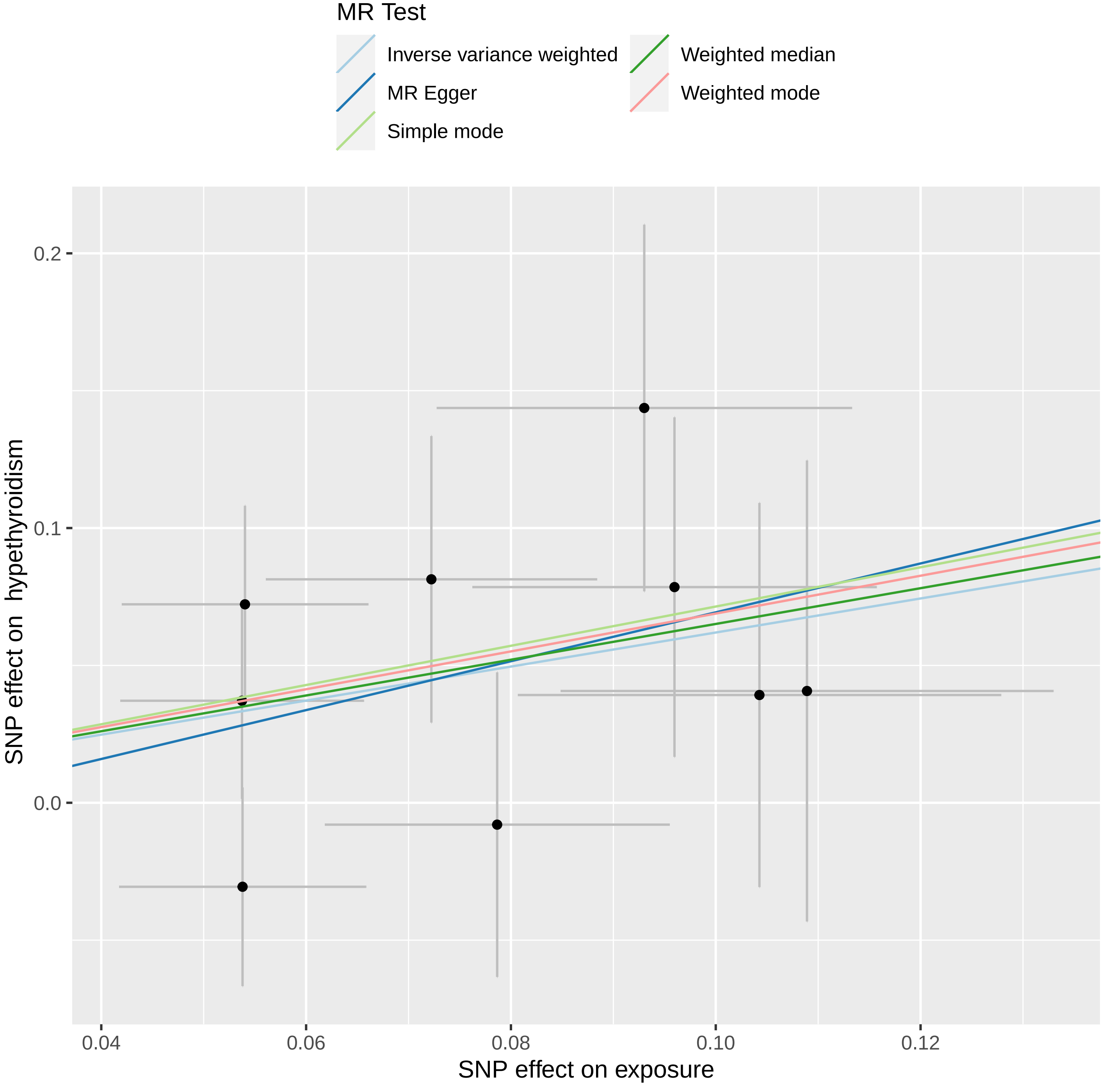
MR Test
Inverse variance weighted
MR Egger
Simple mode
Weighted median
Weighted mode
0.2
0.1
0.0
0.04
0.06
0.08
0.10
0.12
SNP effect on hypethyroidism
SNP effect on exposure

31

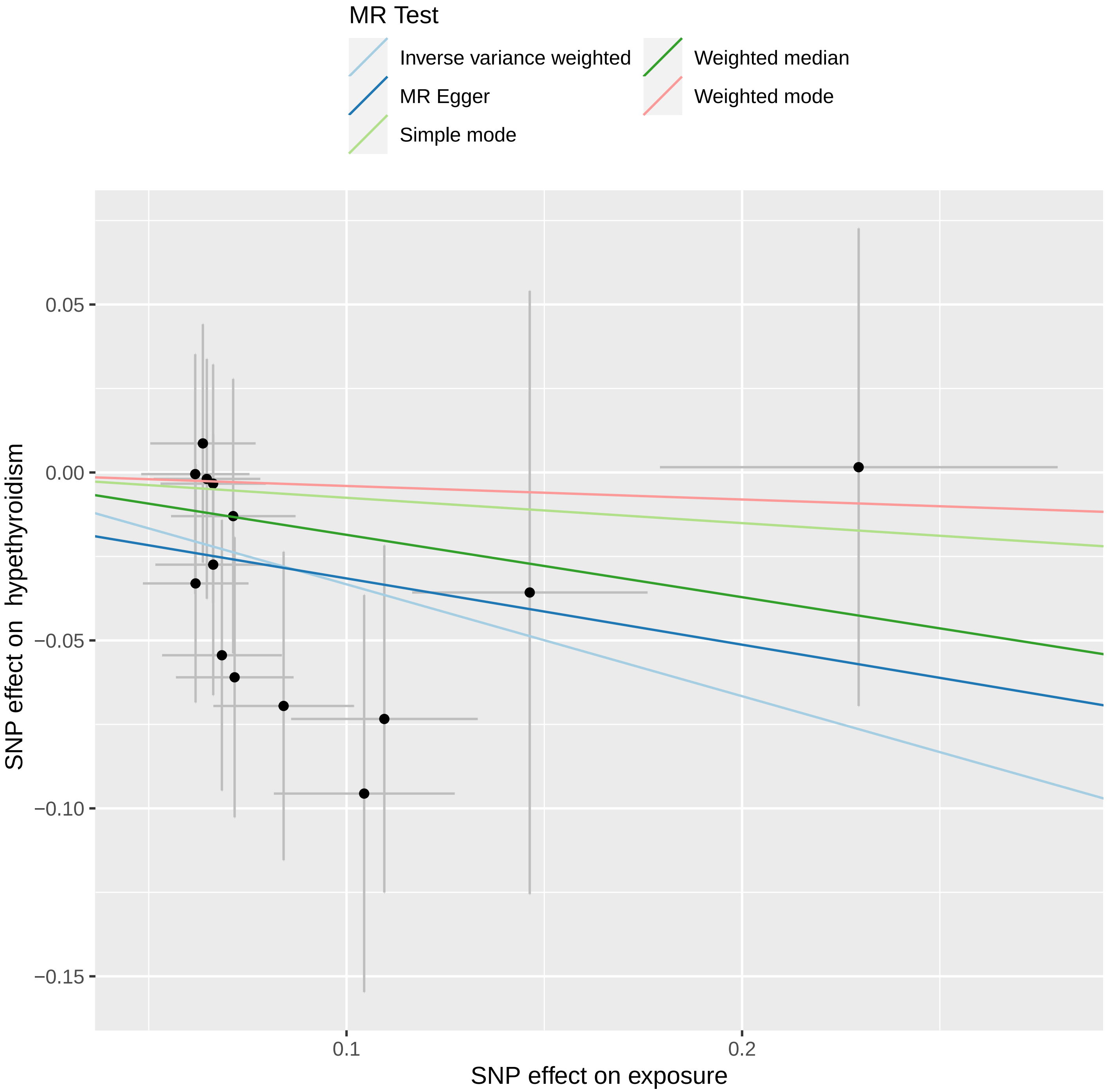
MR Test
Inverse variance weighted
MR Egger
Simple mode
Weighted median
Weighted mode
0.05
0.00
−0.05
−0.10
−0.15
0.1
0.2
SNP effect on hypethyroidism
SNP effect on exposure

32

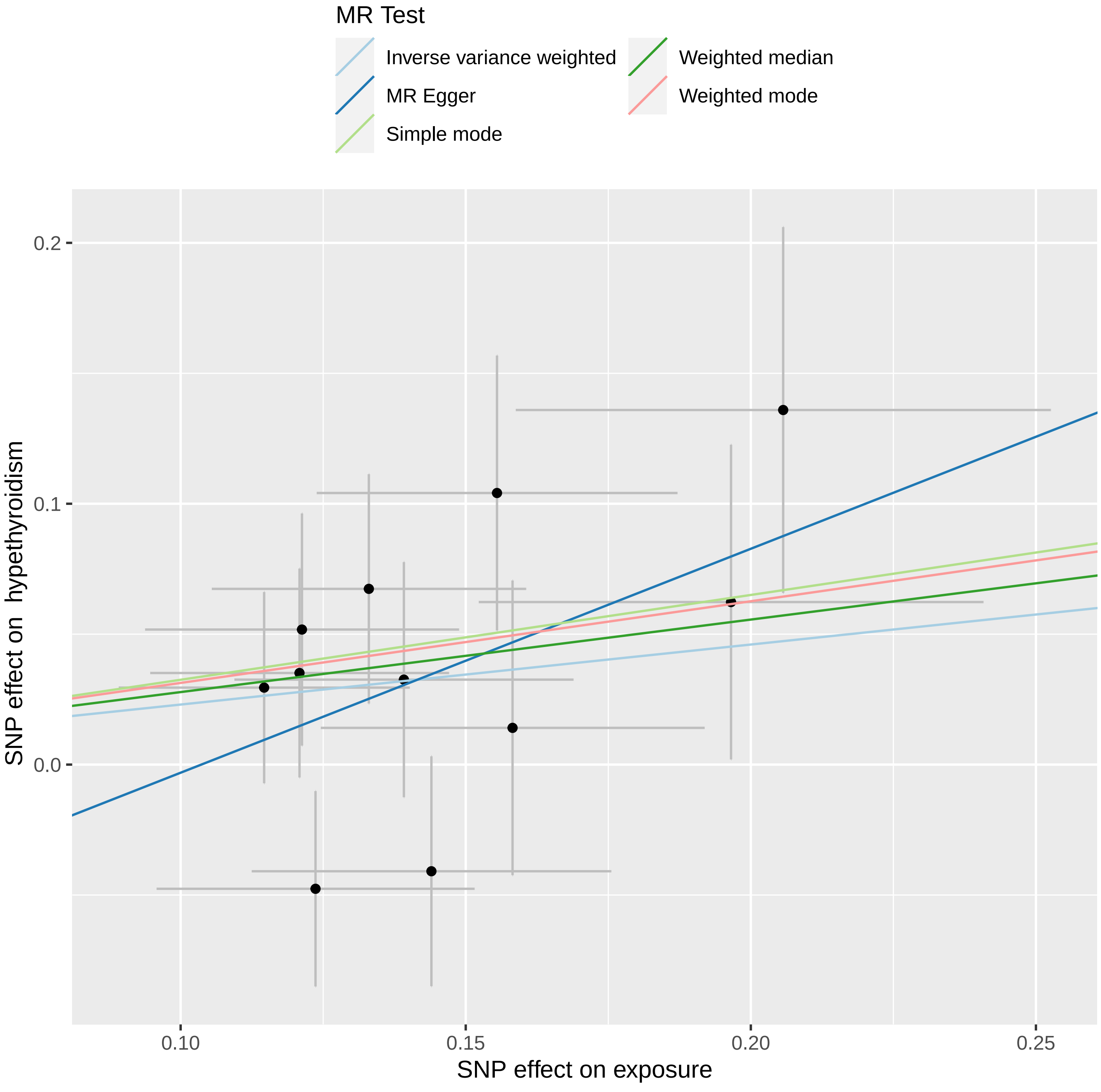
MR Test
Inverse variance weighted
MR Egger
Simple mode
Weighted median
Weighted mode
SNP effect on hypethyroidism
0.2
0.1
0.0
0.10
0.15
0.20
0.25
SNP effect on exposure

33

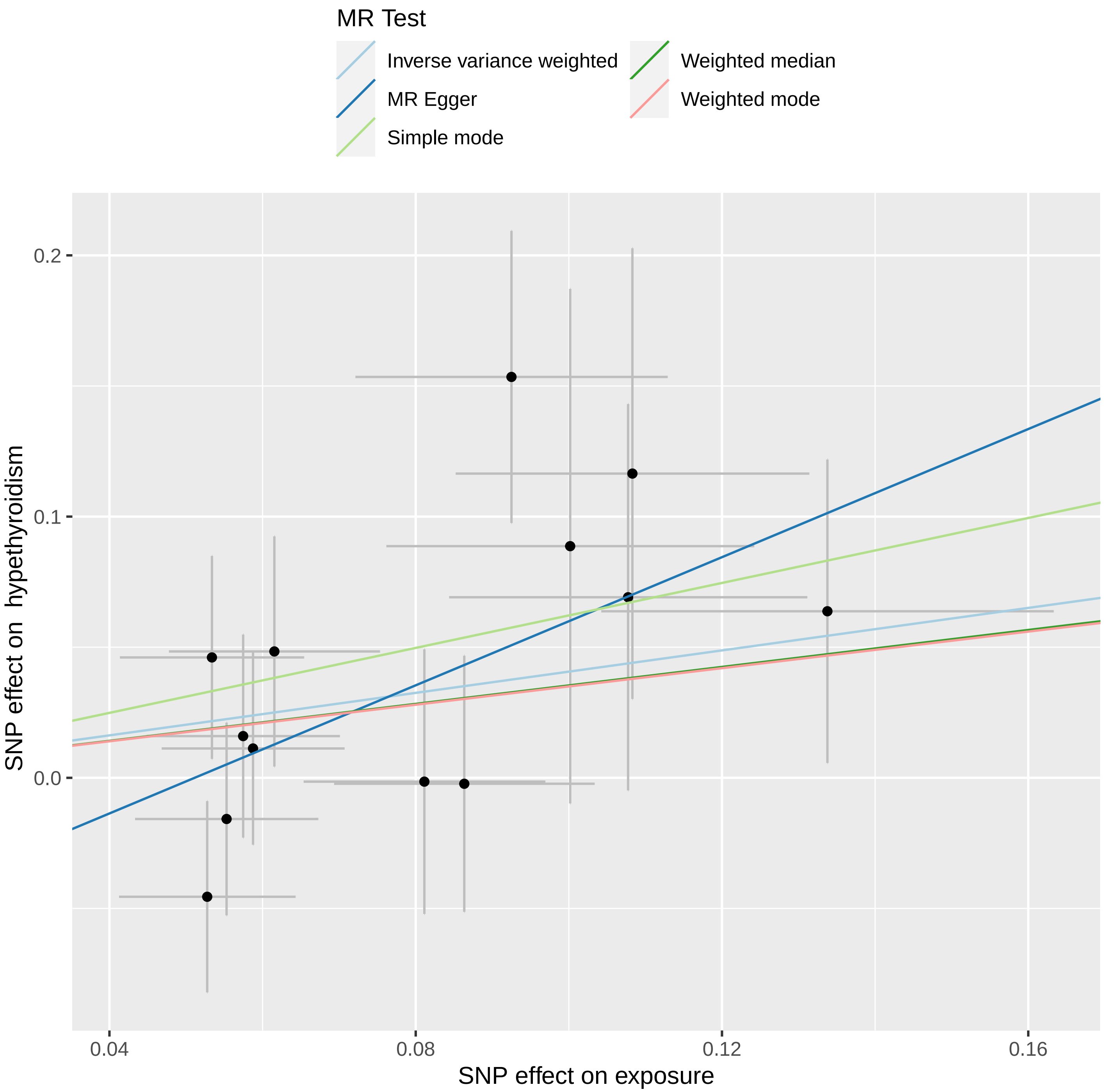
MR Test
Inverse variance weighted
MR Egger
Simple mode
Weighted median
Weighted mode
SNP effect on hypethyroidism
SNP effect on exposure
0.2
0.1
0.0
0.04
0.08
0.12
0.16

34

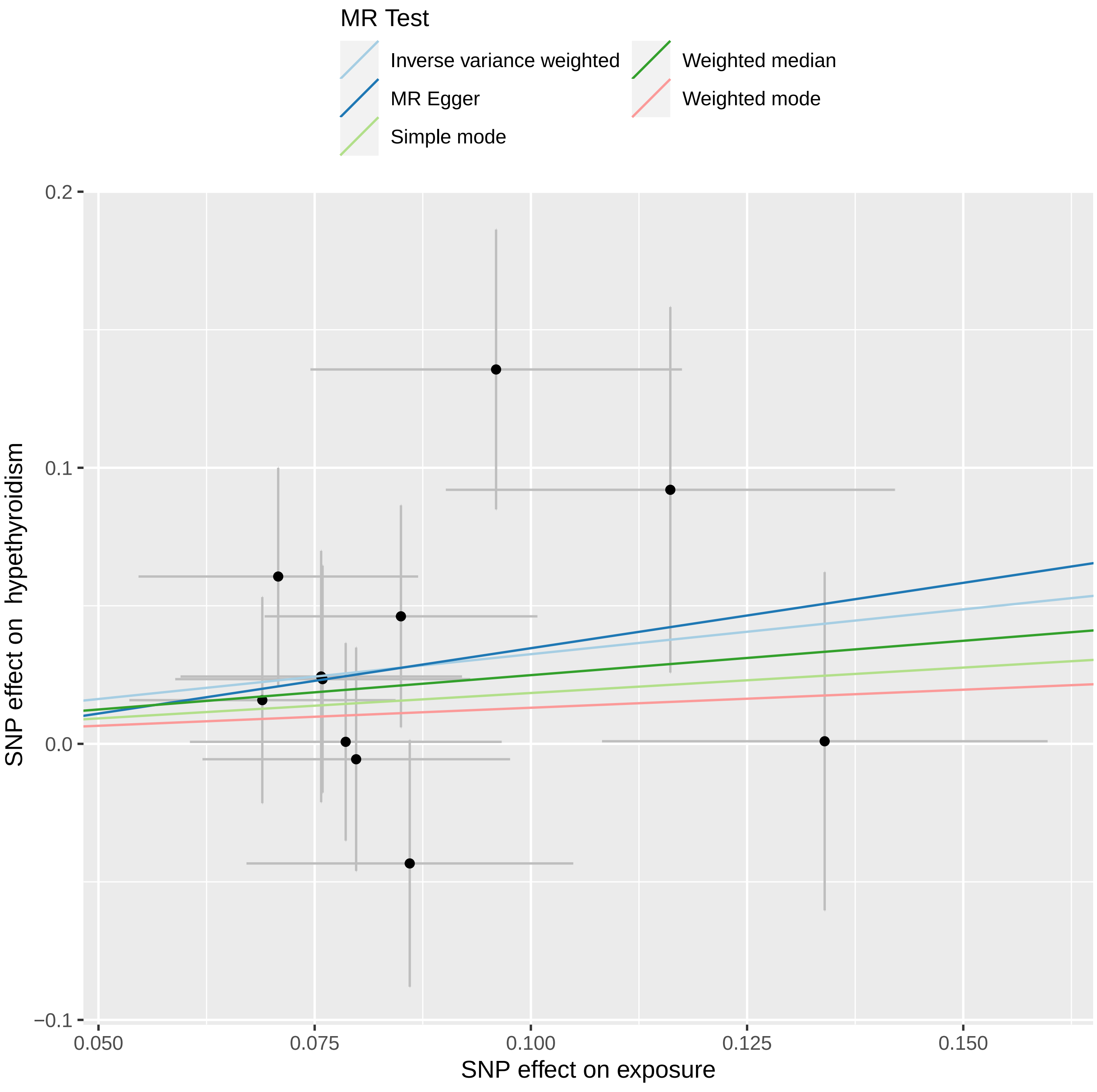
MR Test
Inverse variance weighted
MR Egger
Simple mode
Weighted median
Weighted mode
0.2
0.1
0.0
−0.1
0.050
0.075
0.100
0.125
0.150
SNP effect on hypethyroidism
SNP effect on exposure

35

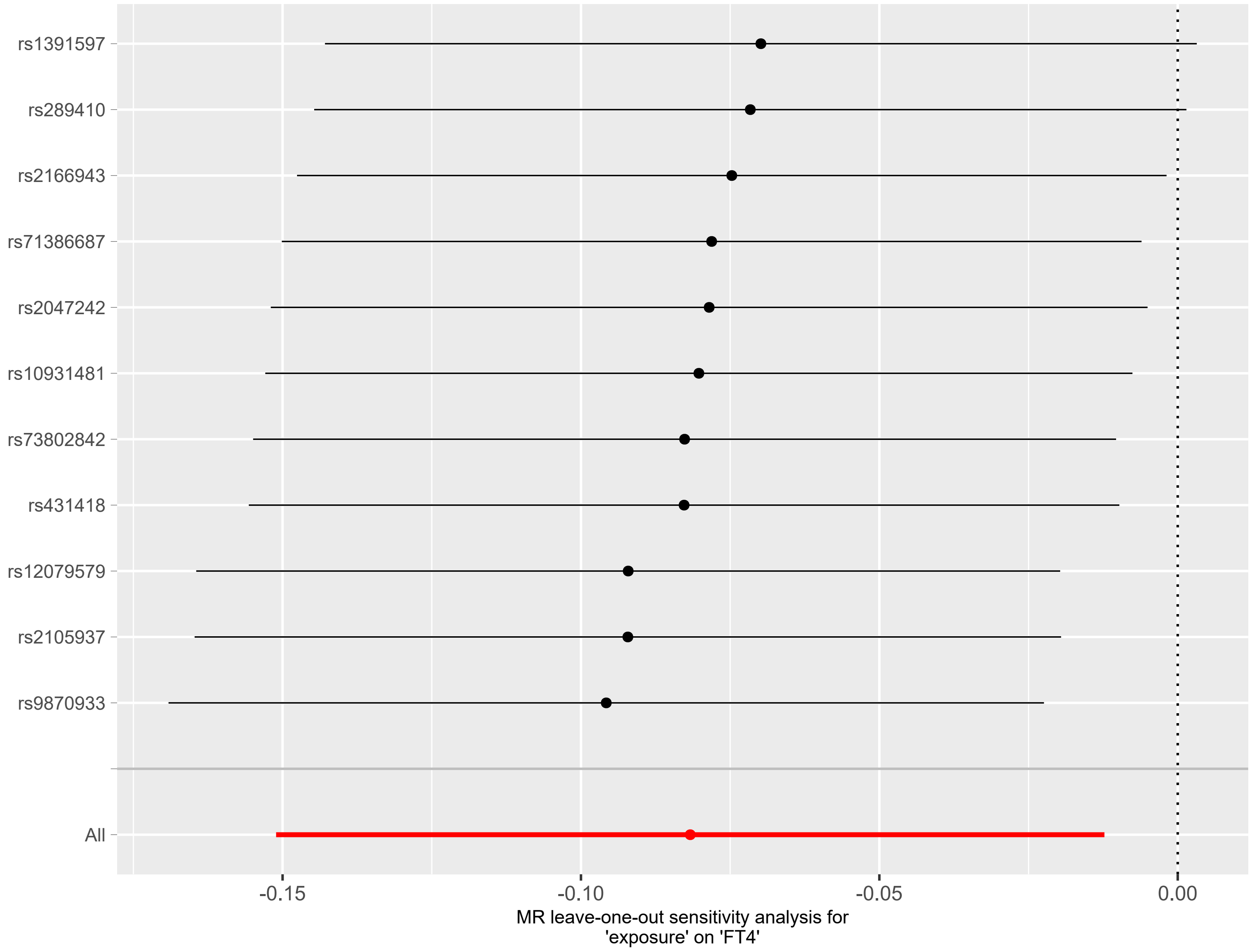
rs1391597
rs289410
rs2166943
rs71386687
rs2047242
rs10931481
rs73802842
rs431418
rs12079579
rs2105937
rs9870933
All
-0.15
-0.10
-0.05
0.00
MR leave-one-out sensitivity analysis for
'exposure' on 'FT4'

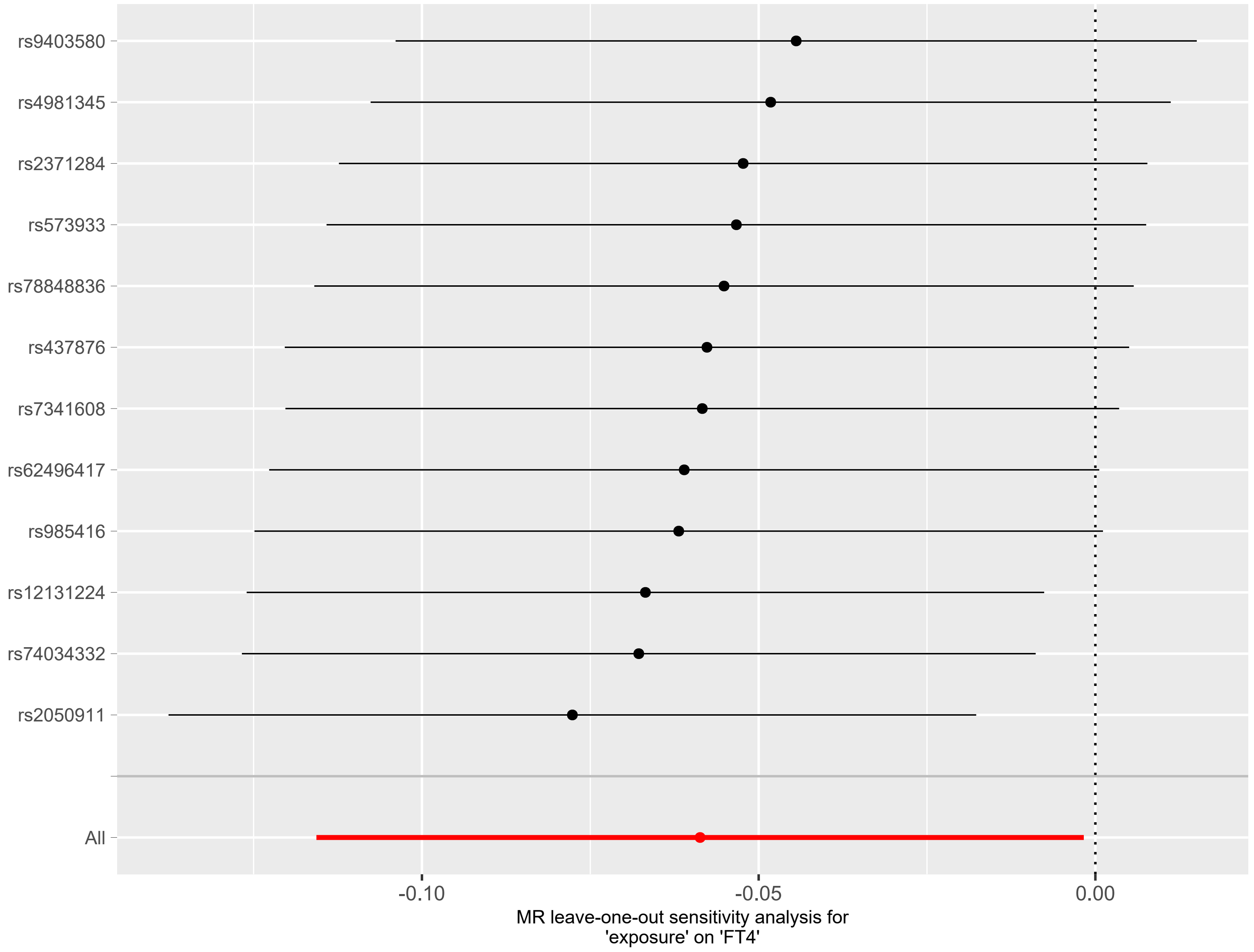
rs9403580
rs4981345
rs2371284
rs573933
rs78848836
rs437876
rs7341608
rs62496417
rs985416
rs12131224
rs74034332
rs2050911
All
-0.10
-0.05
0.00
MR leave-one-out sensitivity analysis for
'exposure' on 'FT4'

37

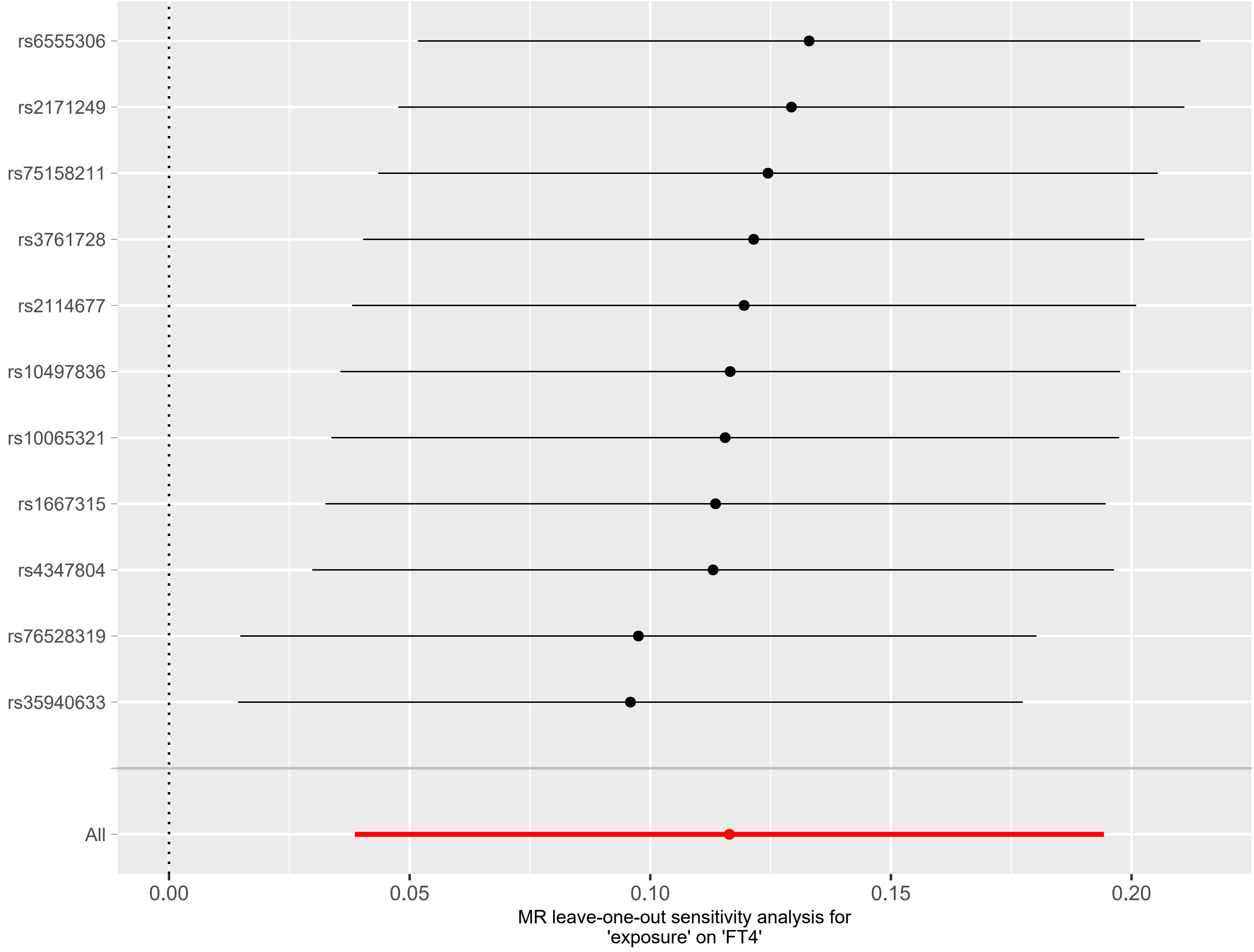
rs6555306
rs2171249
rs75158211
rs3761728
rs2114677
rs10497836
rs10065321
rs1667315
rs4347804
rs76528319
rs35940633
All
0.00
0.05
0.10
0.15
0.20
MR leave-one-out sensitivity analysis for
'exposure' on 'FT4'

38

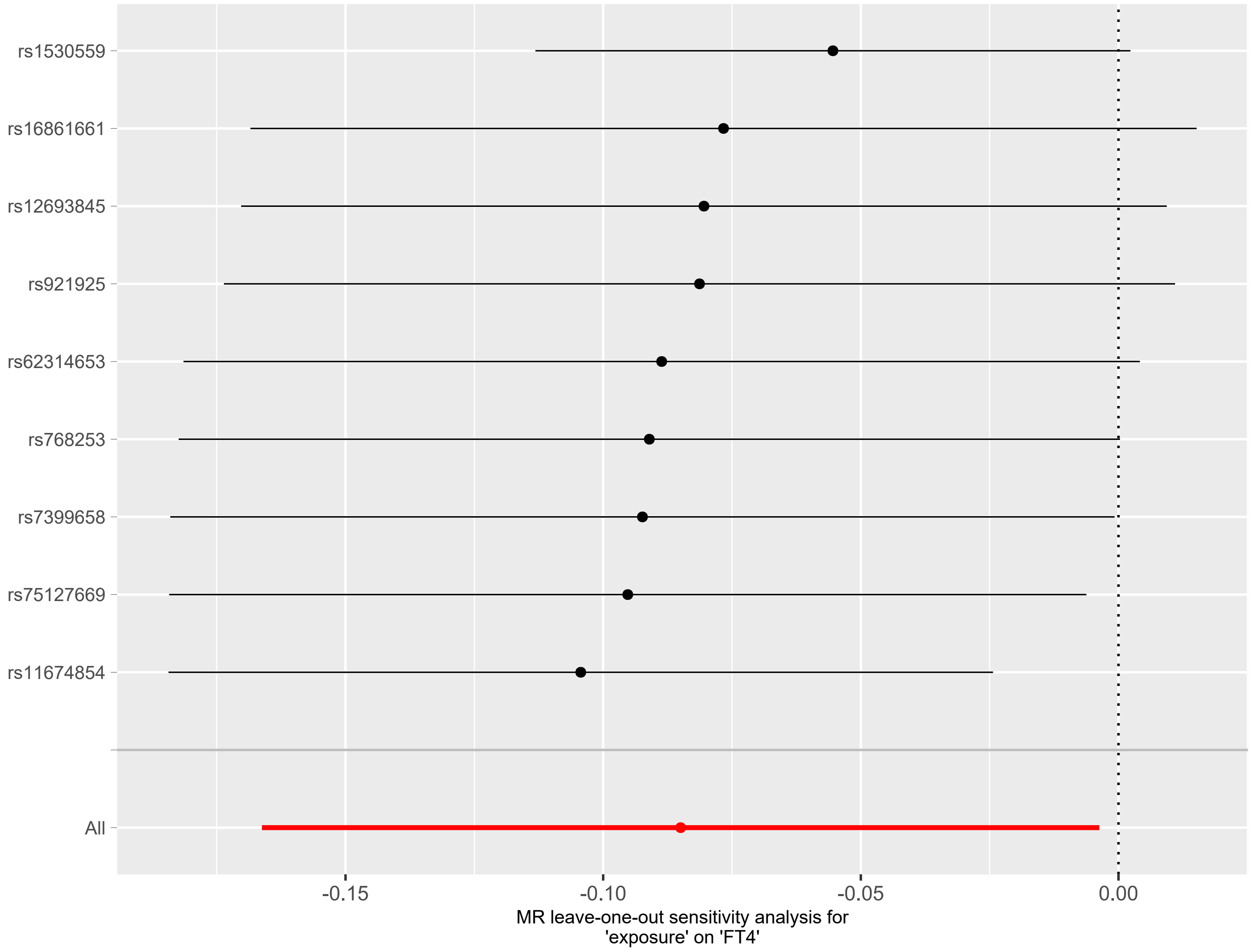
rs1530559
rs16861661
rs12693845
rs921925
rs62314653
rs768253
rs7399658
rs75127669
rs11674854
All
-0.15
-0.10
-0.05
0.00
MR leave-one-out sensitivity analysis for
'exposure' on 'FT4'

39

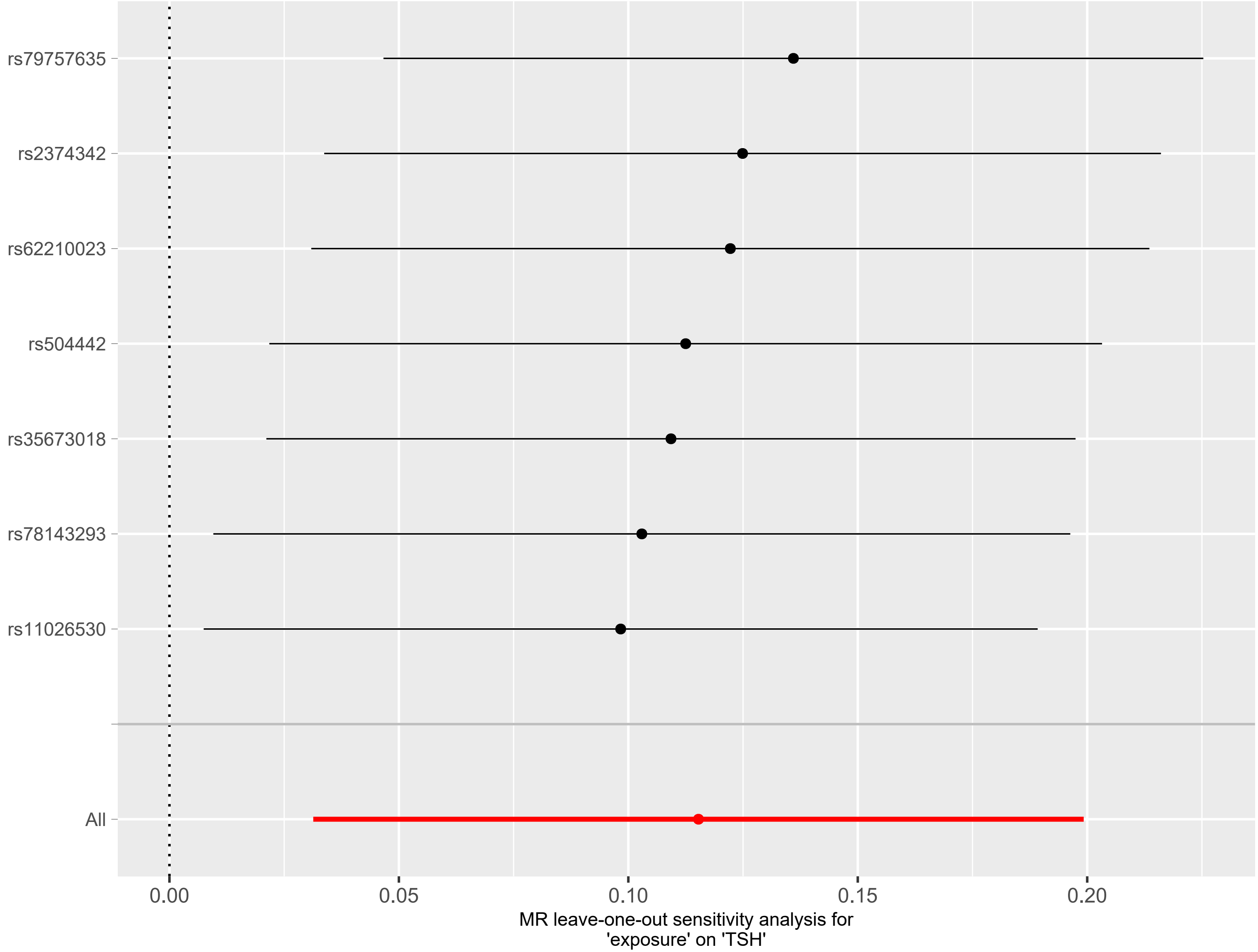

rs79757635
rs2374342
rs62210023
rs504442
rs35673018
rs78143293
rs11026530
All
0.00
0.05
0.10
0.15
0.20
MR leave-one-out sensitivity analysis for
'exposure' on 'TSH'

40

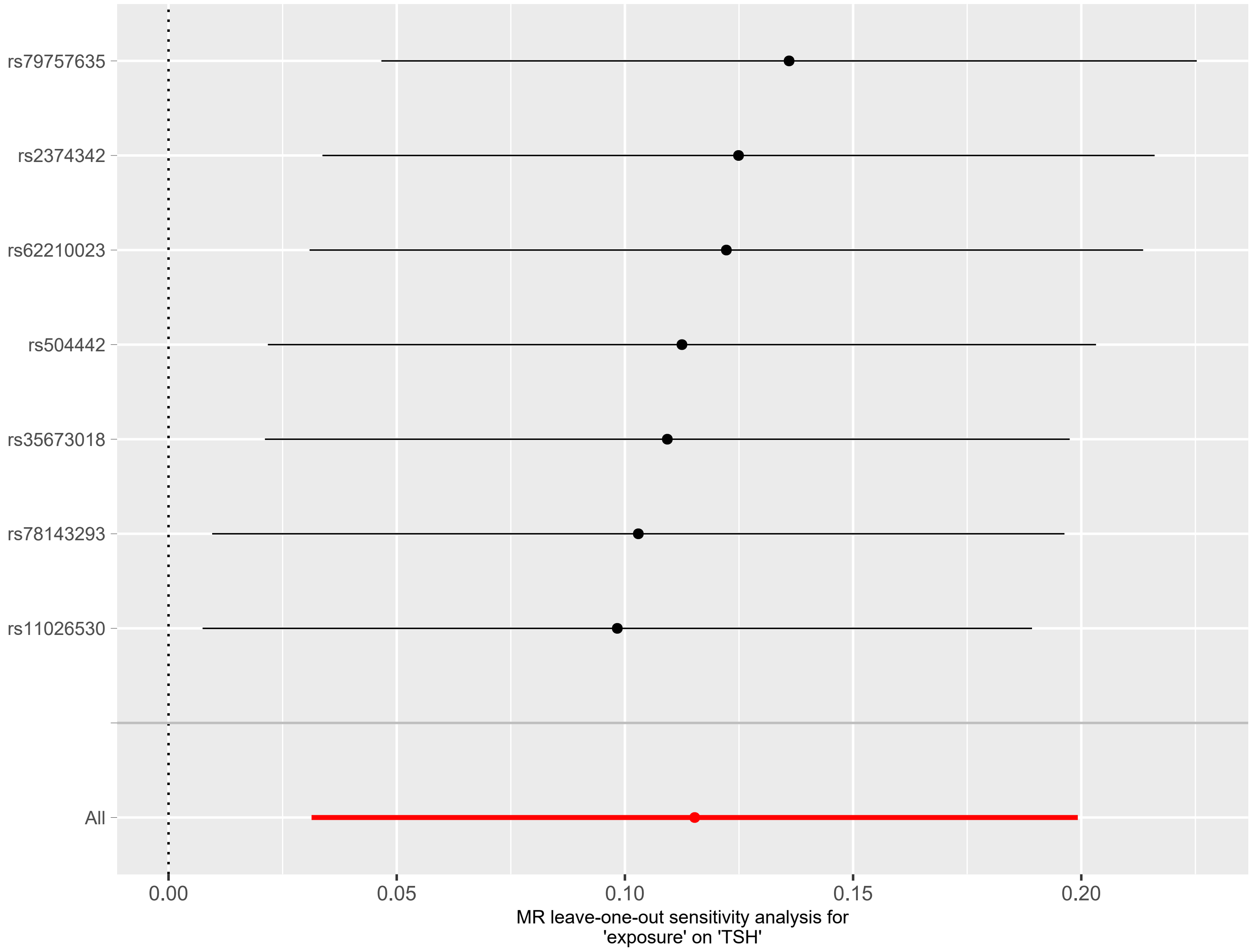

rs79757635
rs2374342
rs62210023
rs504442
rs35673018
rs78143293
rs11026530
All
0.00
0.05
0.10
0.15
0.20
MR leave-one-out sensitivity analysis for
'exposure' on 'TSH'

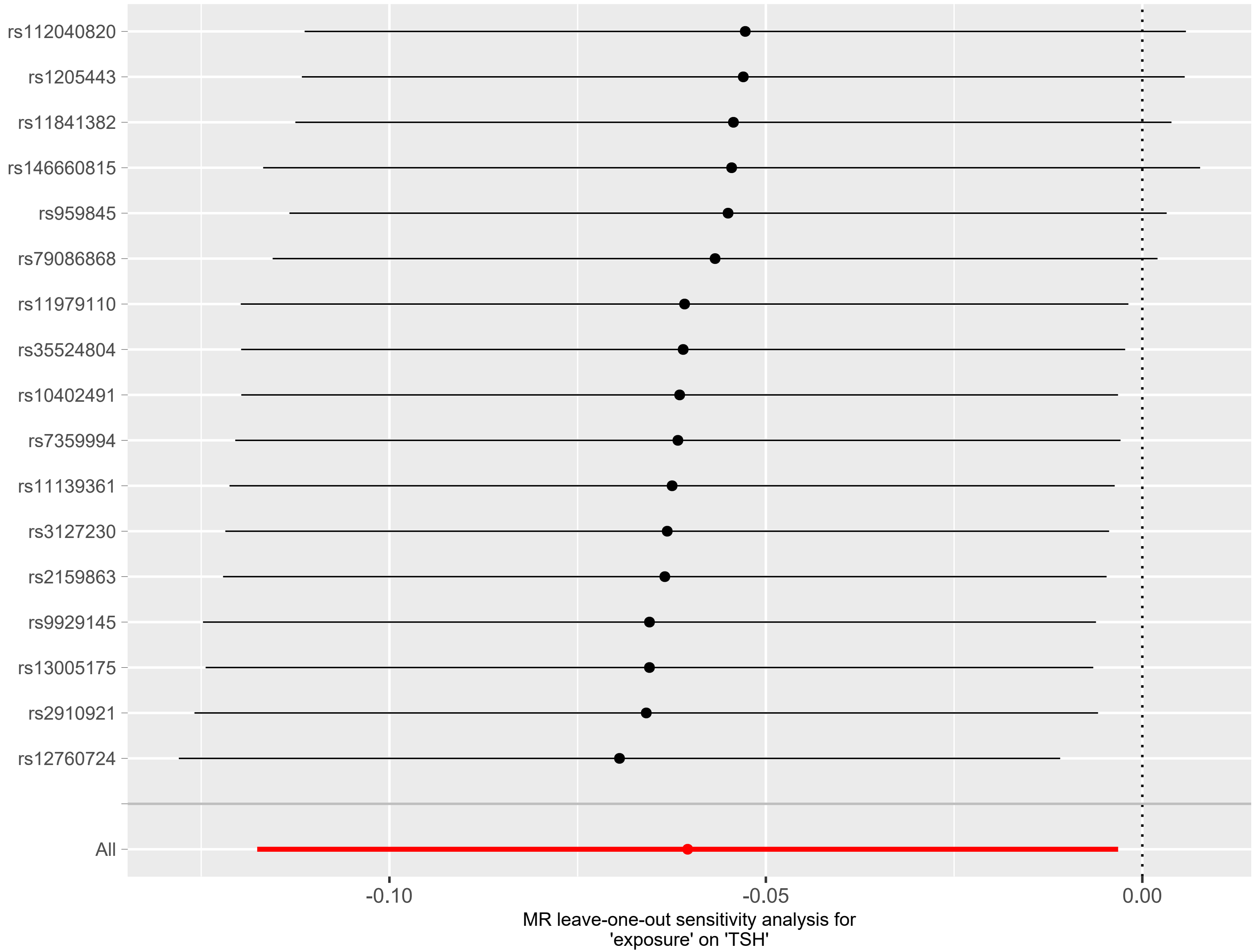
rs112040820
rs1205443
rs11841382
rs146660815
rs959845
rs79086868
rs11979110
rs35524804
rs10402491
rs7359994
rs11139361
rs3127230
rs2159863
rs9929145
rs13005175
rs2910921
rs12760724
All
-0.10
-0.05
0.00
MR leave-one-out sensitivity analysis for
'exposure' on 'TSH'

42

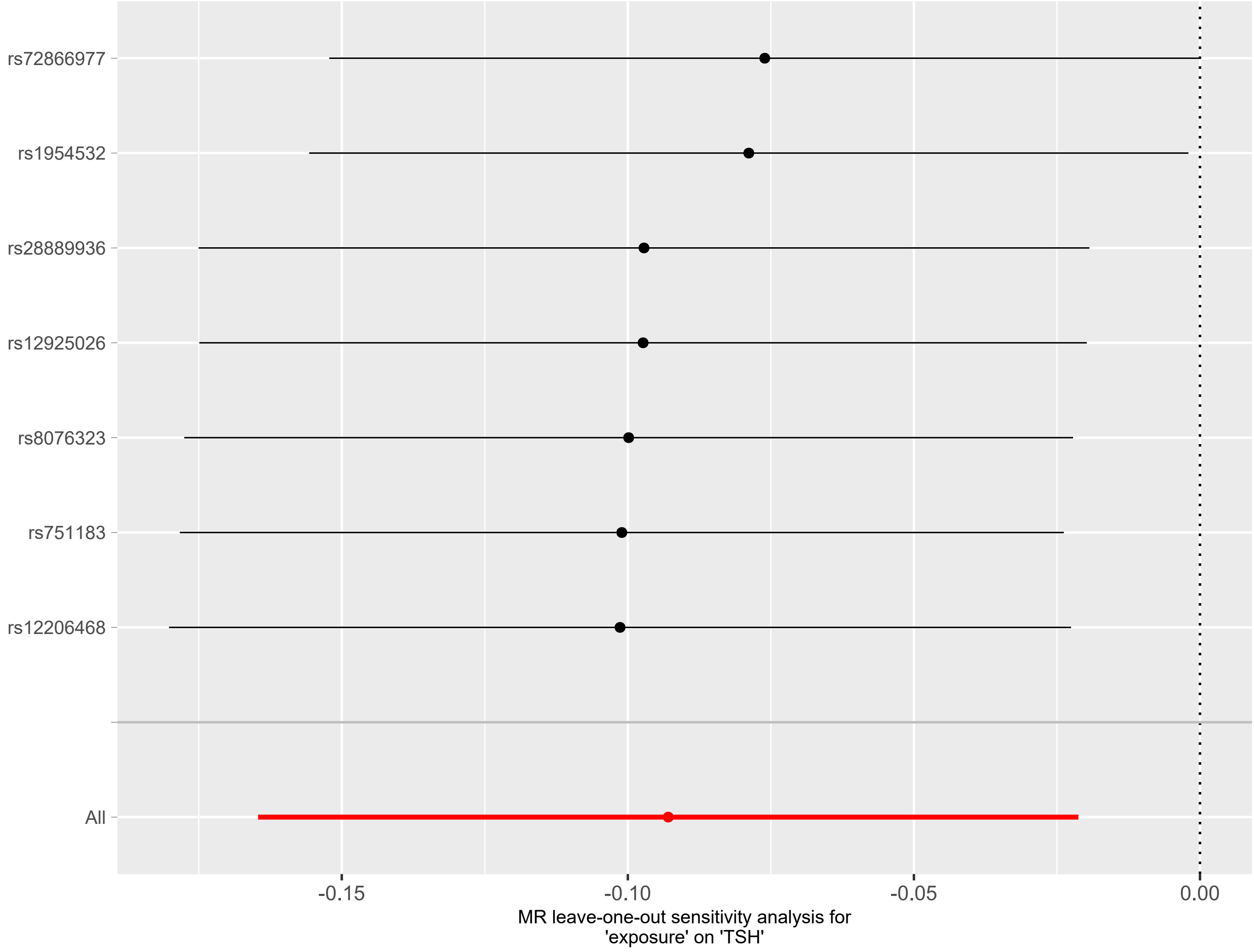

rs72866977
rs1954532
rs28889936
rs12925026
rs8076323
rs751183
rs12206468
All
-0.15
-0.10
-0.05
0.00
MR leave-one-out sensitivity analysis for
'exposure' on 'TSH'

43

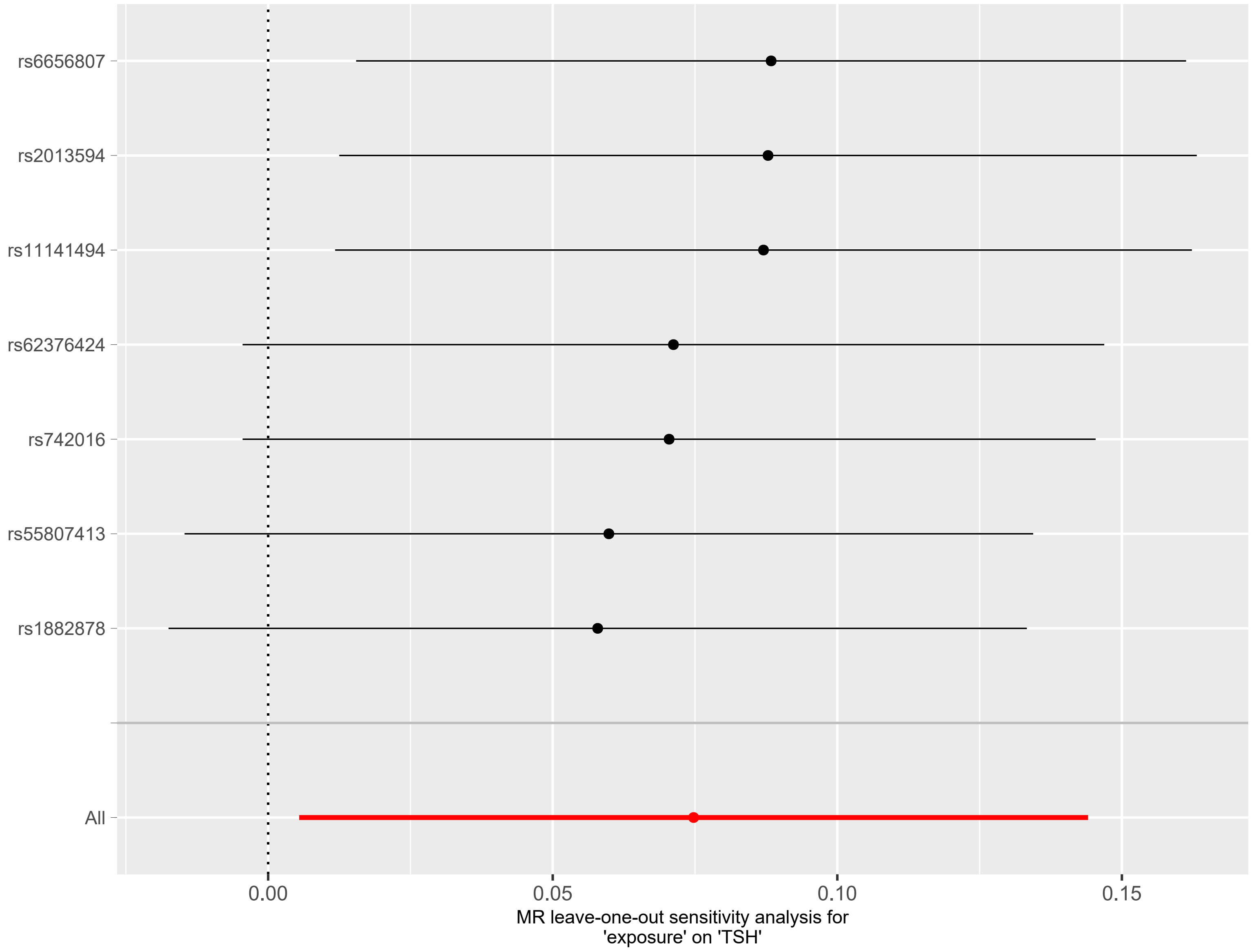
rs6656807
rs2013594
rs11141494
rs62376424
rs742016
rs55807413
rs1882878
All
0.00
0.05
0.10
0.15
MR leave-one-out sensitivity analysis for
'exposure' on 'TSH'

44

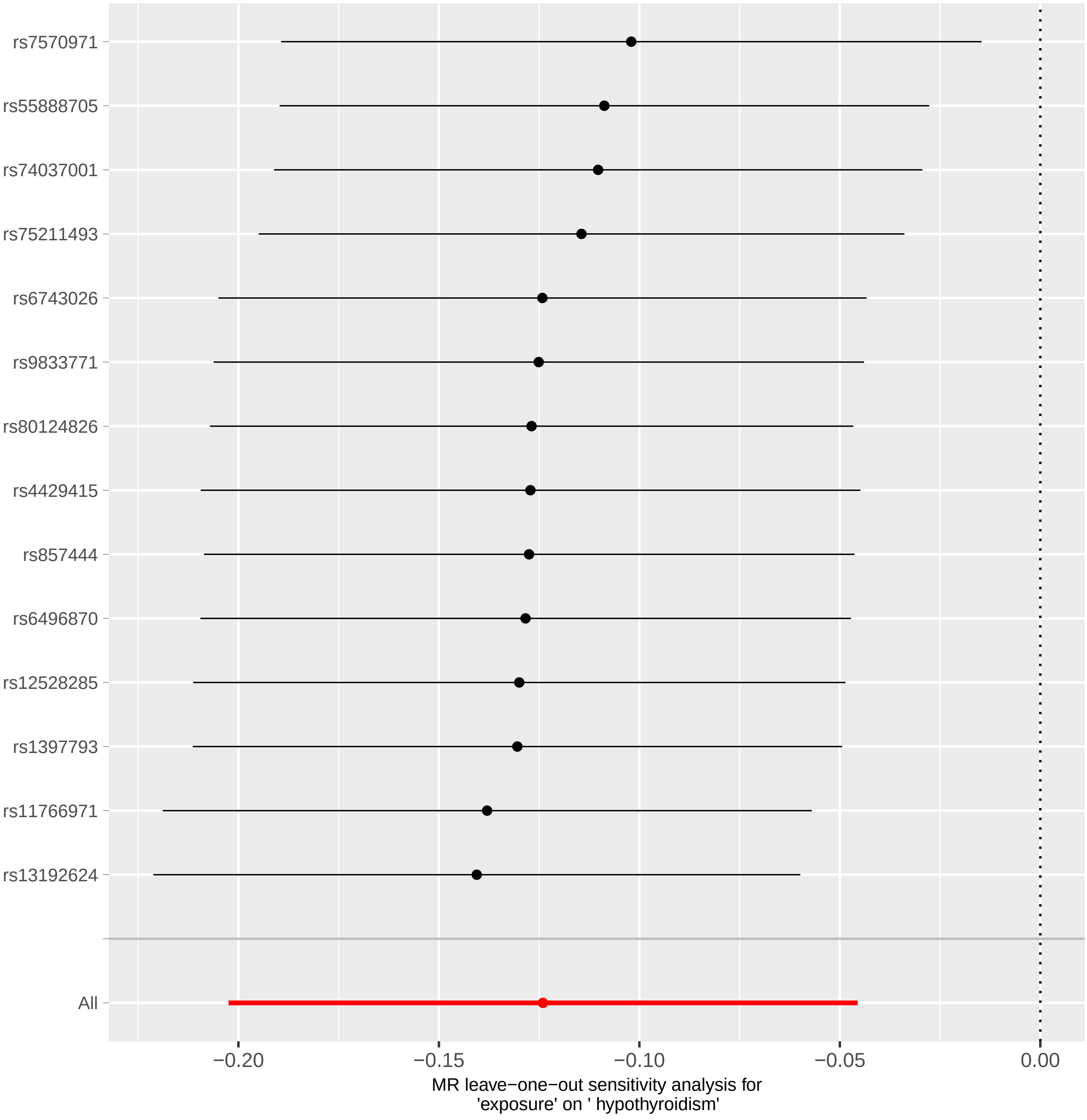

rs7570971
rs55888705
rs74037001
rs75211493
rs6743026
rs9833771
rs80124826
rs4429415
rs857444
rs6496870
rs12528285
rs1397793
rs11766971
rs13192624
All
−0.20
−0.15
−0.10
−0.05
0.00
MR leave−one−out sensitivity analysis for
'exposure' on ' hypothyroidism'

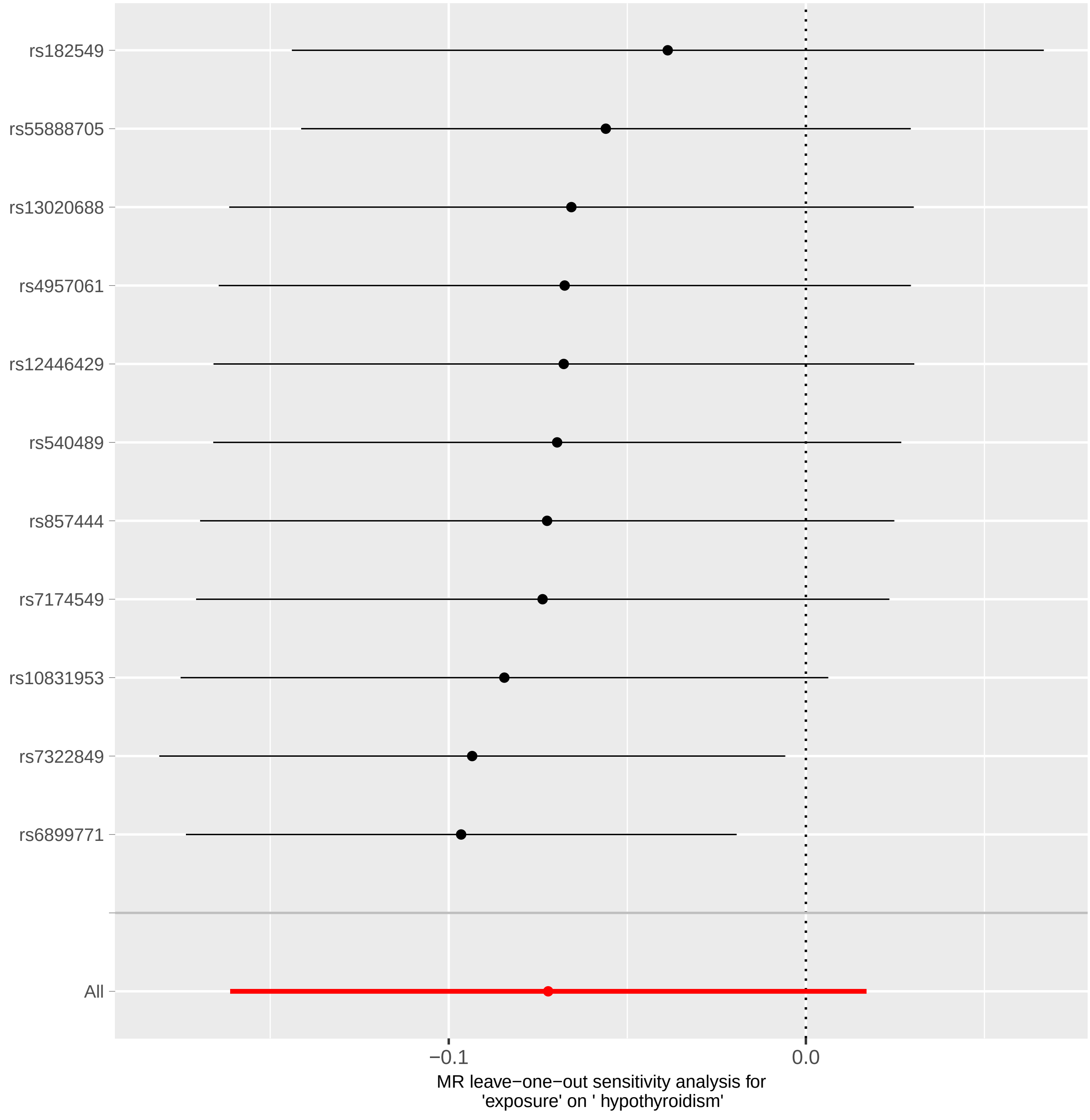
rs182549
rs55888705
rs13020688
rs4957061
rs12446429
rs540489
rs857444
rs7174549
rs10831953
rs7322849
rs6899771
All
−0.1
0.0
MR leave−one−out sensitivity analysis for
'exposure' on ' hypothyroidism'

46

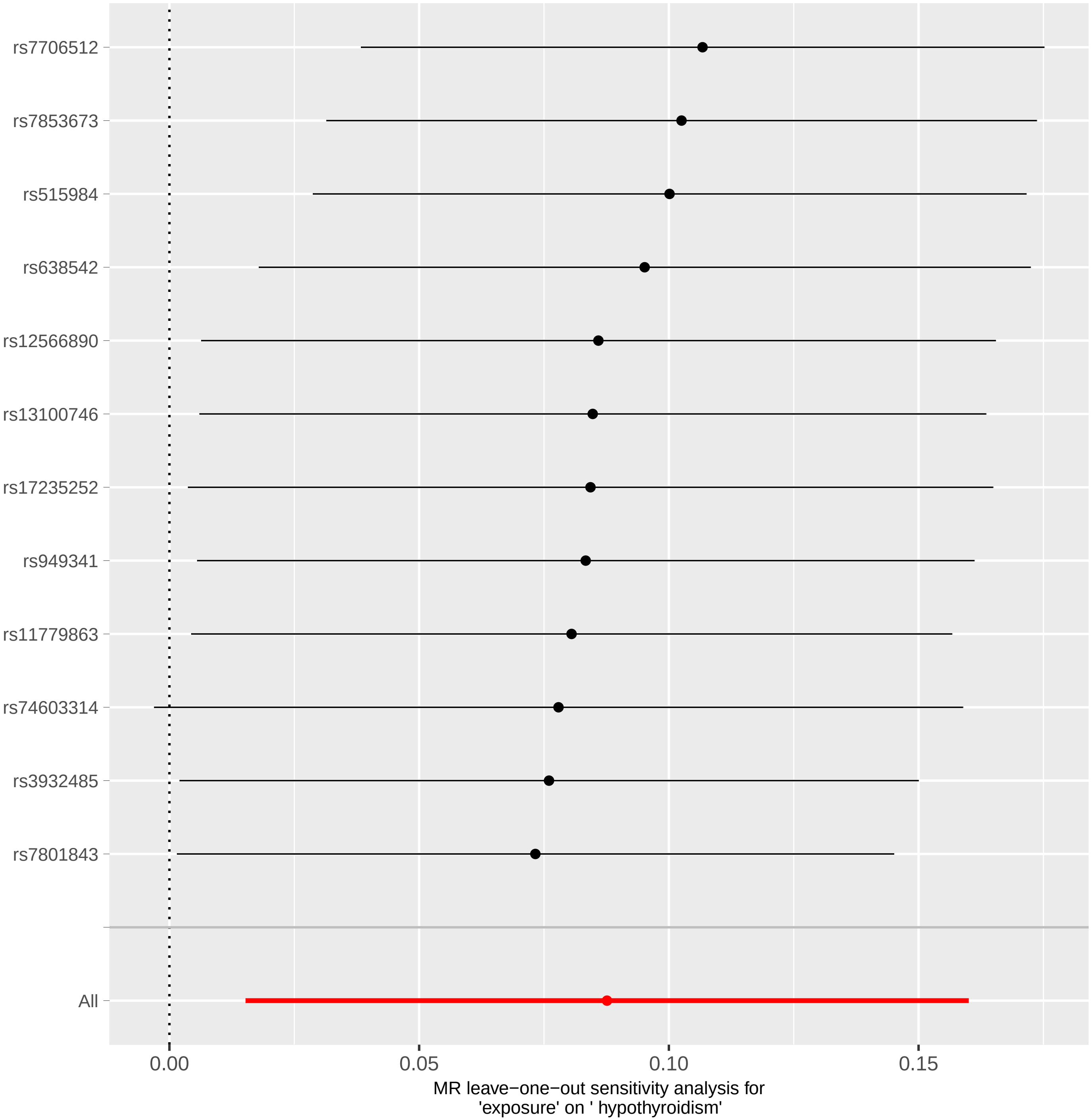
rs7706512
rs7853673
rs515984
rs638542
rs12566890
rs13100746
rs17235252
rs949341
rs11779863
rs74603314
rs3932485
rs7801843
All
0.00
0.05
0.10
0.15
MR leave−one−out sensitivity analysis for
'exposure' on ' hypothyroidism'

47

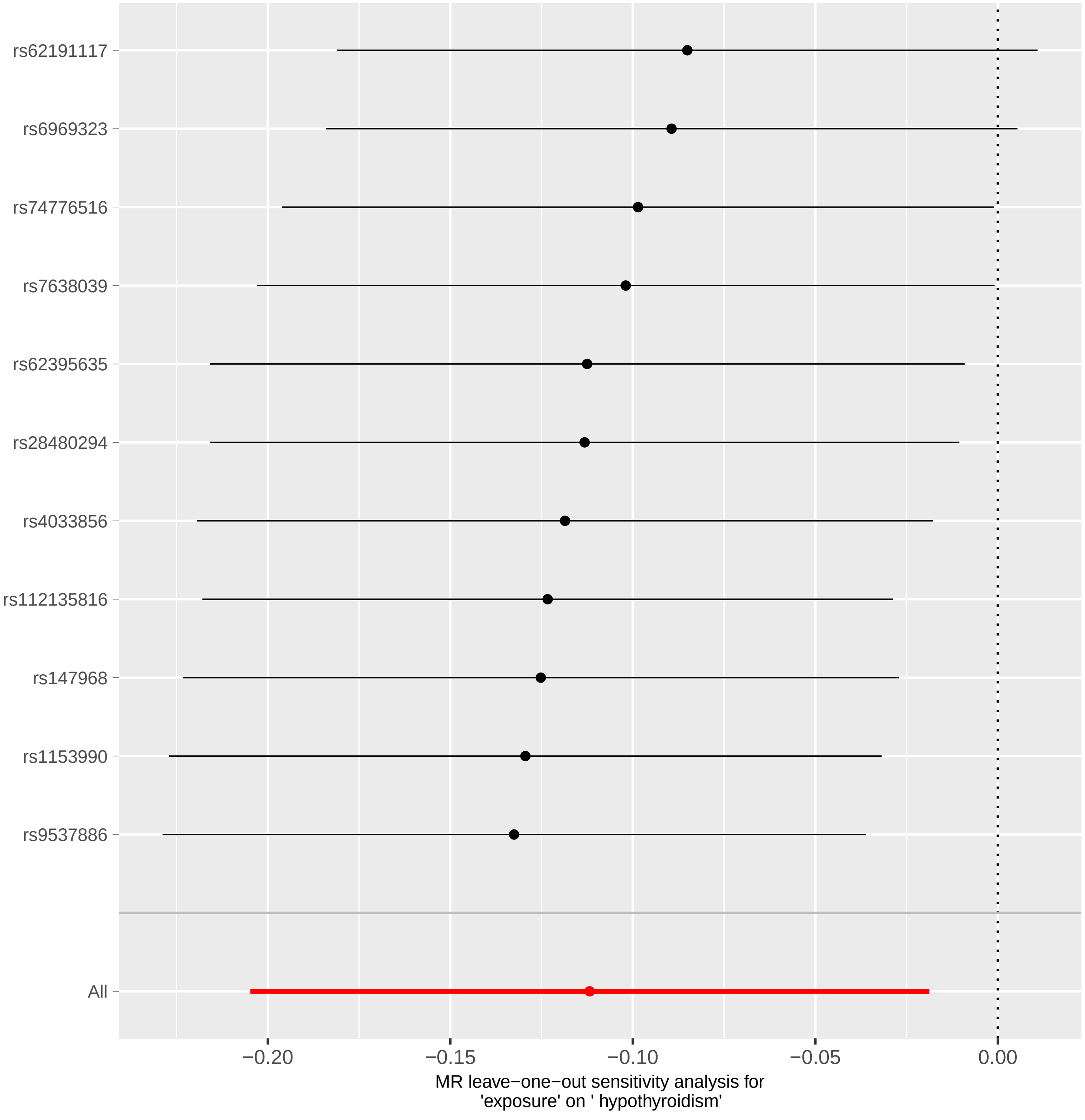
rs62191117
rs6969323
rs74776516
rs7638039
rs62395635
rs28480294
rs4033856
rs112135816
rs147968
rs1153990
rs9537886
All
−0.20
−0.15
−0.10
−0.05
0.00
MR leave−one−out sensitivity analysis for
'exposure' on ' hypothyroidism'

48

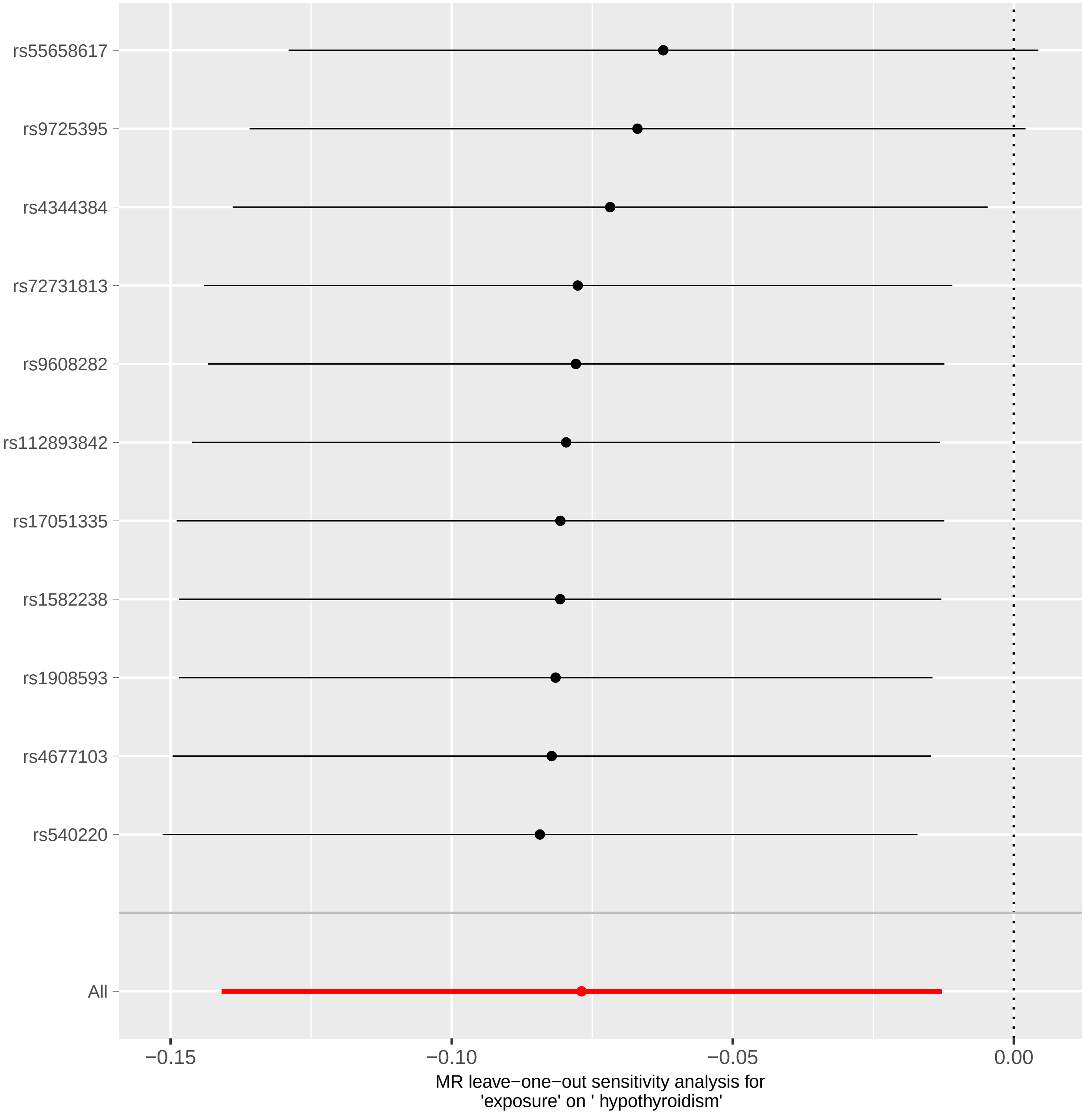
rs55658617
rs9725395
rs4344384
rs72731813
rs9608282
rs112893842
rs17051335
rs1582238
rs1908593
rs4677103
rs540220
All
−0.15
−0.10
−0.05
0.00
MR leave−one−out sensitivity analysis for
'exposure' on ' hypothyroidism'

49

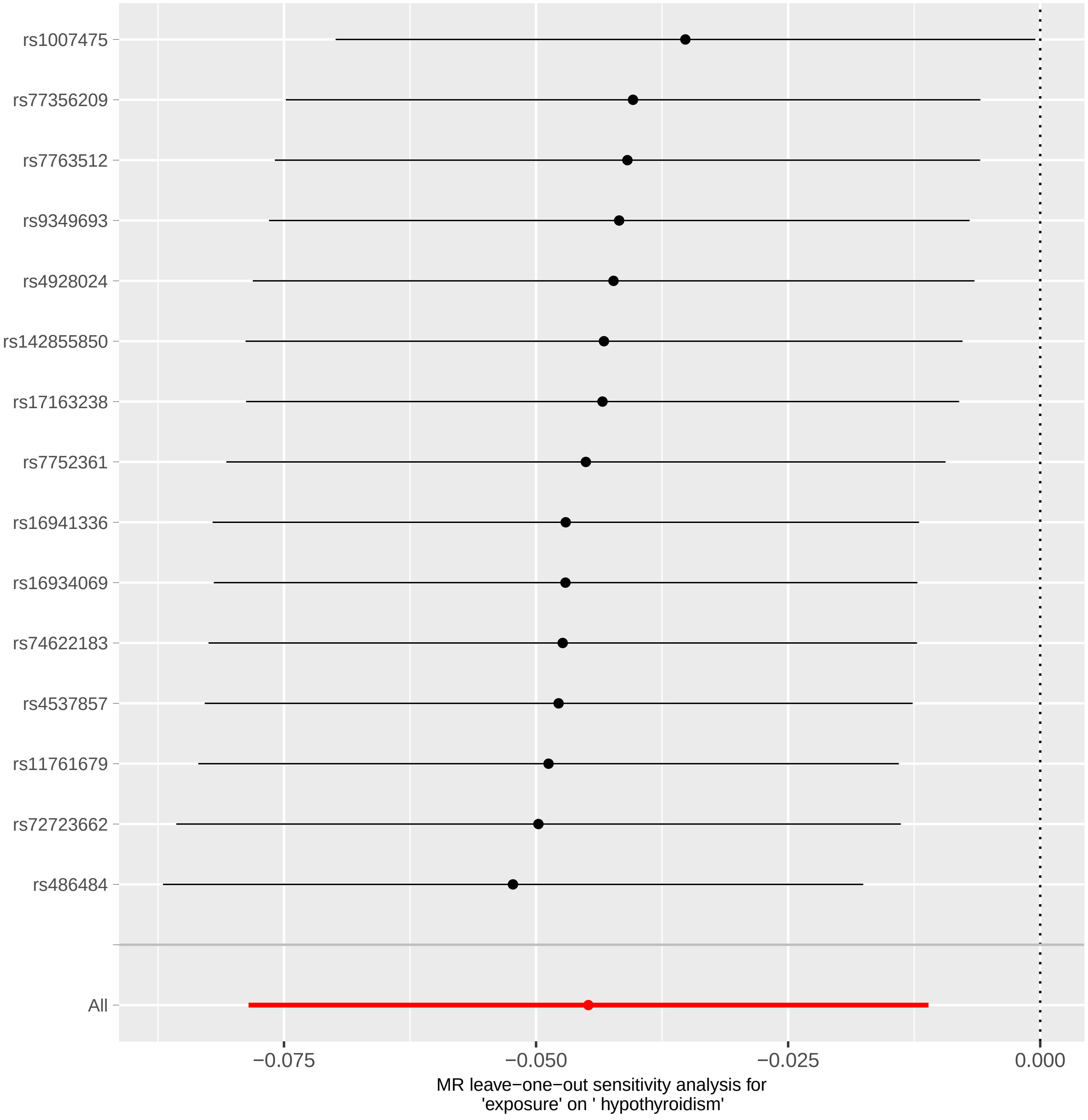

rs1007475
rs77356209
rs7763512
rs9349693
rs4928024
rs142855850
rs17163238
rs7752361
rs16941336
rs16934069
rs74622183
rs4537857
rs11761679
rs72723662
rs486484
All
−0.075
−0.050
−0.025
0.000
MR leave−one−out sensitivity analysis for
'exposure' on ' hypothyroidism'

50

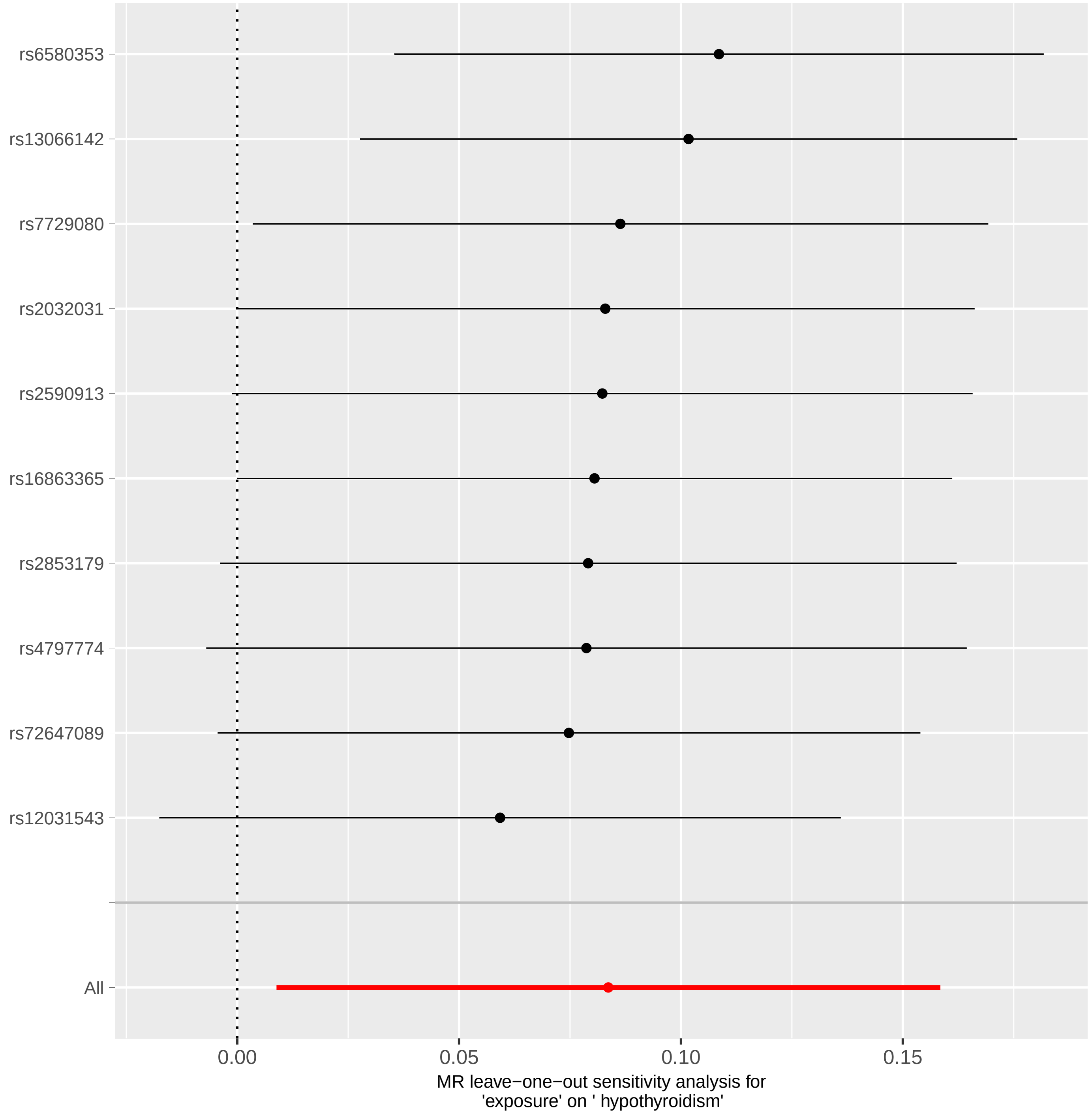
rs6580353
rs13066142
rs7729080
rs2032031
rs2590913
rs16863365
rs2853179
rs4797774
rs72647089
rs12031543
All
0.00
0.05
0.10
0.15
MR leave−one−out sensitivity analysis for
'exposure' on ' hypothyroidism'

51

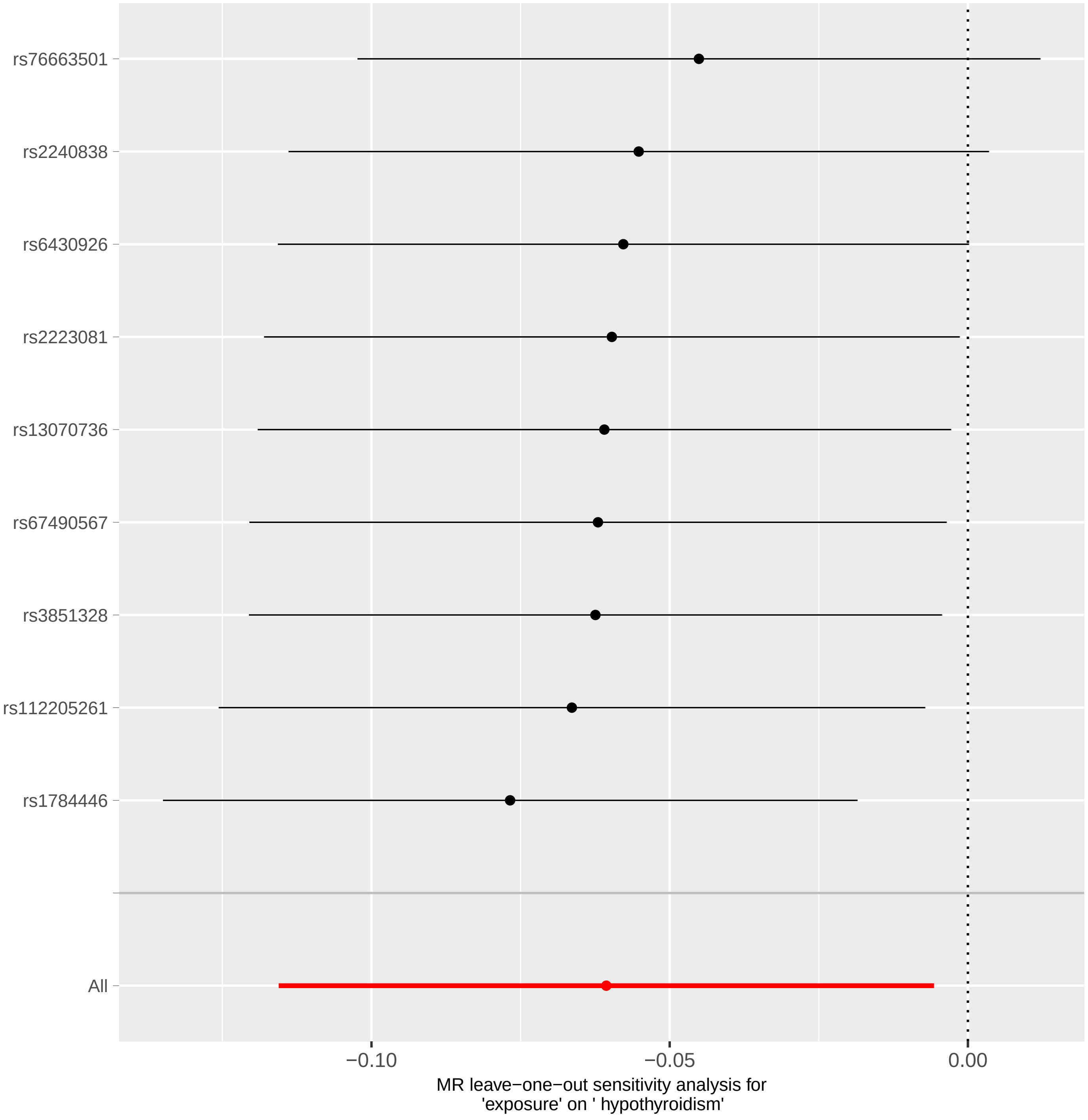

rs76663501
rs2240838
rs6430926
rs2223081
rs13070736
rs67490567
rs3851328
rs112205261
rs1784446
All
−0.10
−0.05
0.00
MR leave−one−out sensitivity analysis for
'exposure' on ' hypothyroidism'

52

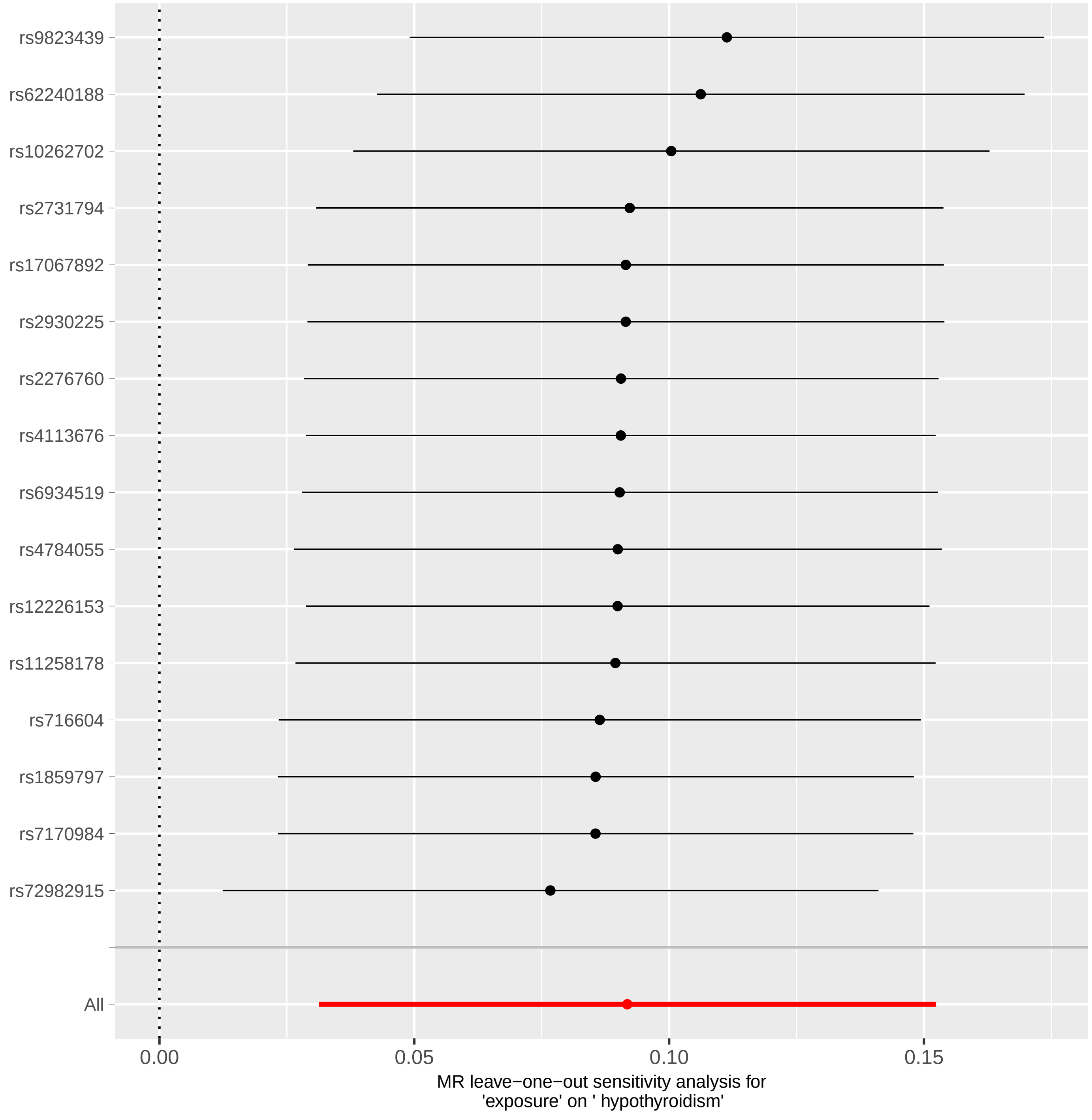
rs9823439
rs62240188
rs10262702
rs2731794
rs17067892
rs2930225
rs2276760
rs4113676
rs6934519
rs4784055
rs12226153
rs11258178
rs716604
rs1859797
rs7170984
rs72982915
All
0.00
0.05
0.10
0.15
MR leave−one−out sensitivity analysis for
'exposure' on ' hypothyroidism'

53

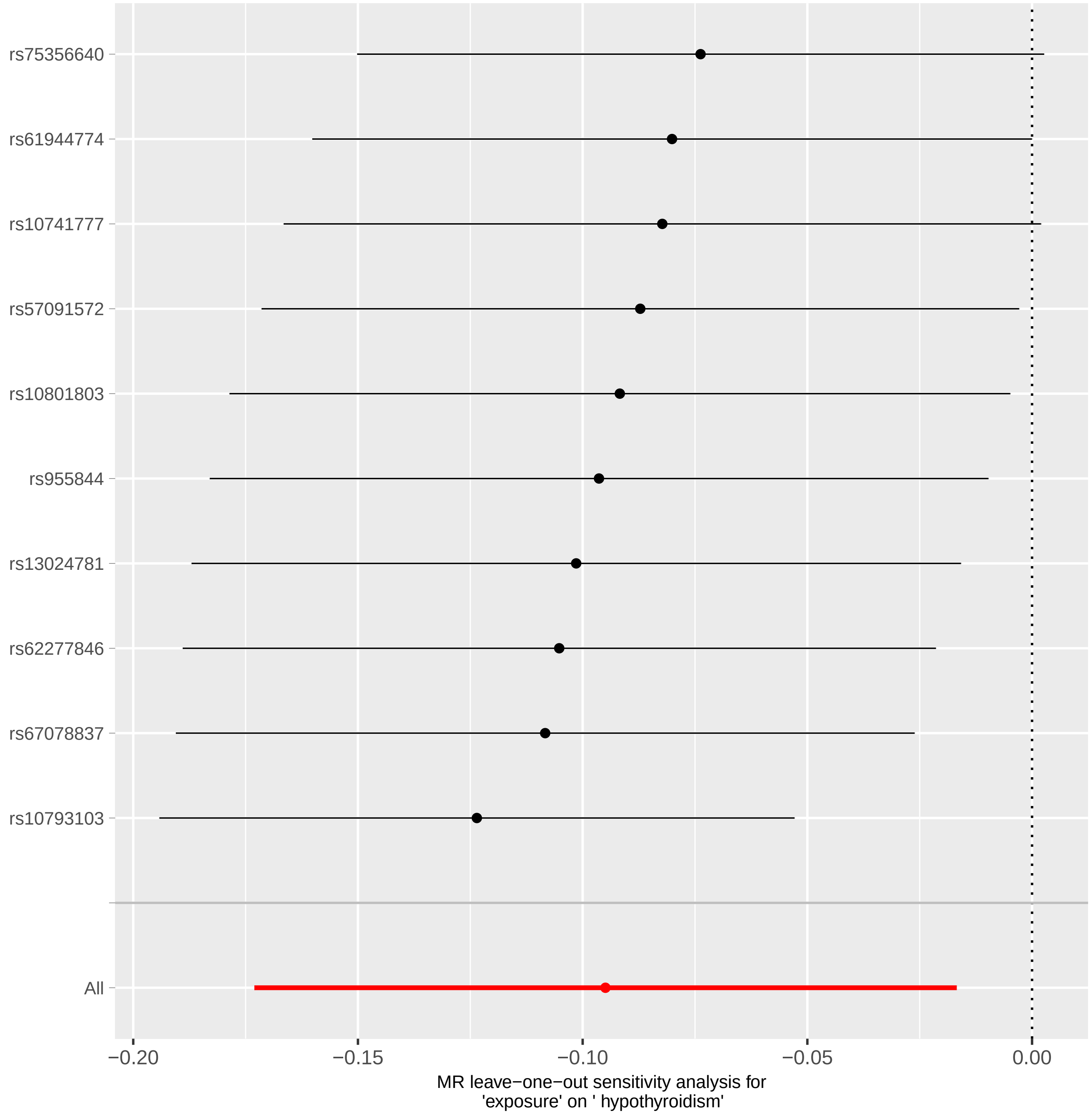
rs75356640
rs61944774
rs10741777
rs57091572
rs10801803
rs955844
rs13024781
rs62277846
rs67078837
rs10793103
All
−0.20
−0.15
−0.10
−0.05
0.00
MR leave−one−out sensitivity analysis for
'exposure' on ' hypothyroidism'

54

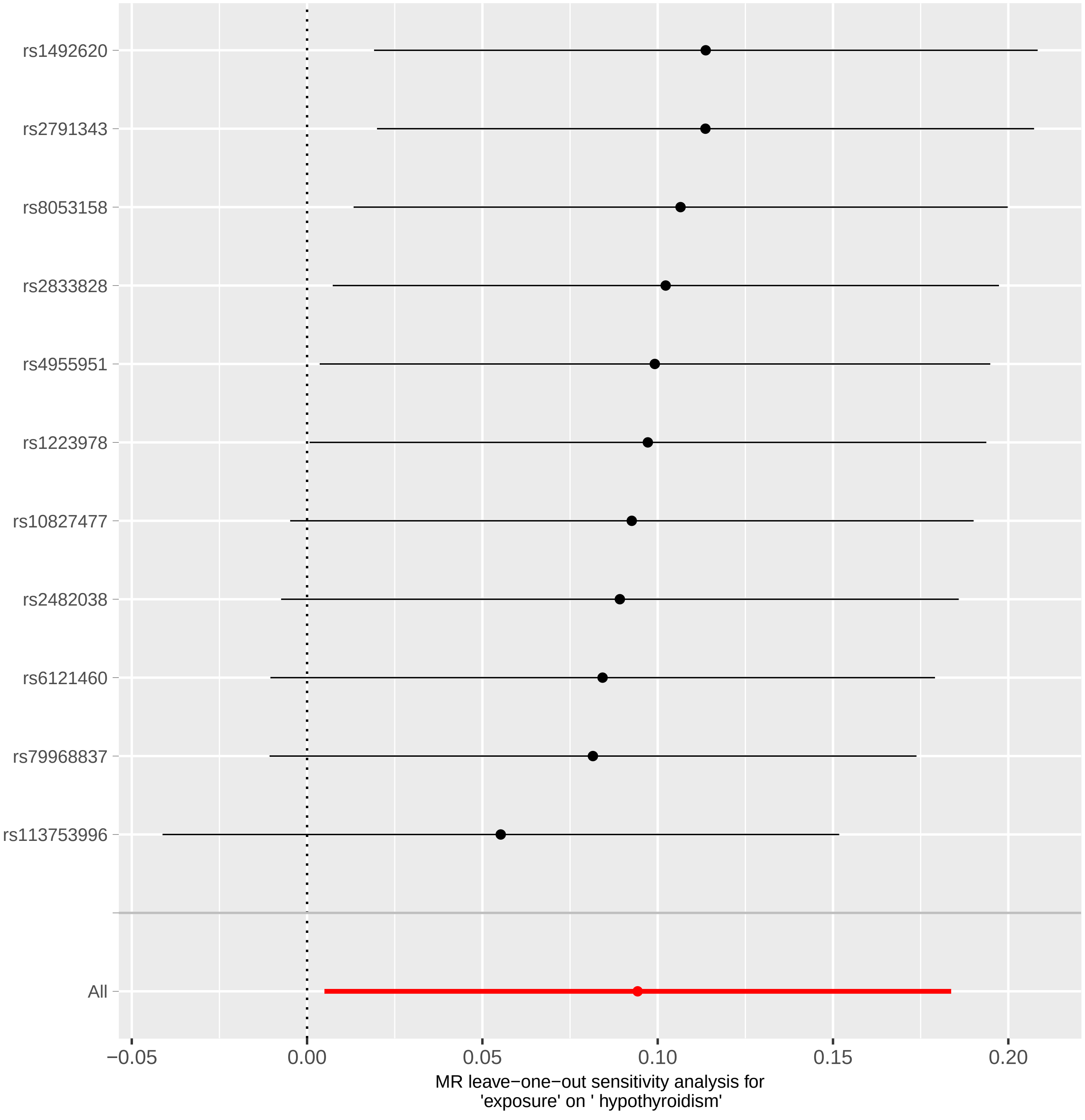

rs1492620
rs2791343
rs8053158
rs2833828
rs4955951
rs1223978
rs10827477
rs2482038
rs6121460
rs79968837
rs113753996
All
−0.05
0.00
0.05
0.10
0.15
0.20
MR leave−one−out sensitivity analysis for
'exposure' on ' hypothyroidism'

55

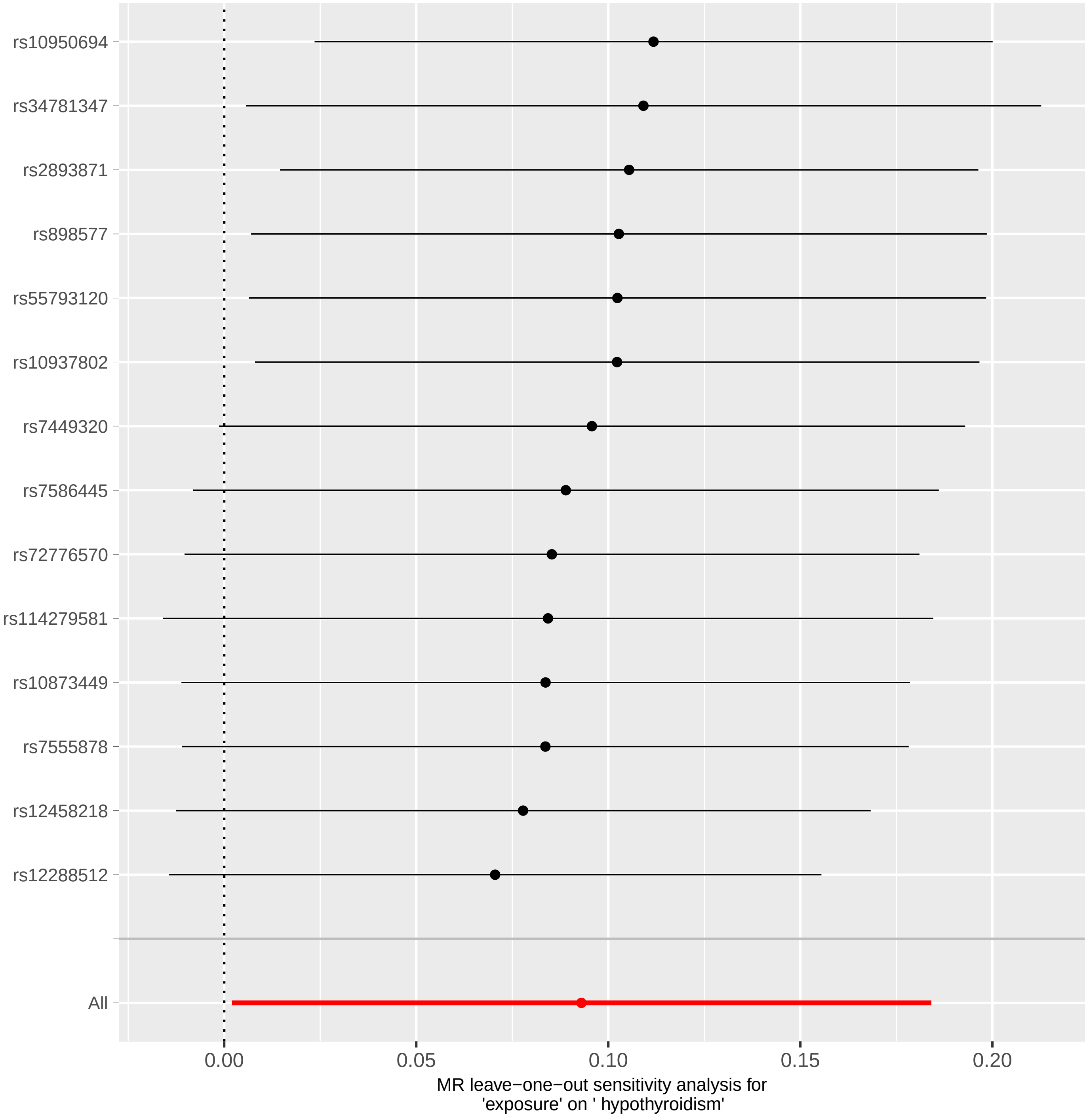

rs10950694
rs34781347
rs2893871
rs898577
rs55793120
rs10937802
rs7449320
rs7586445
rs72776570
rs114279581
rs10873449
rs7555878
rs12458218
rs12288512
All
0.00
0.05
0.10
0.15
0.20
MR leave−one−out sensitivity analysis for
'exposure' on ' hypothyroidism'

56

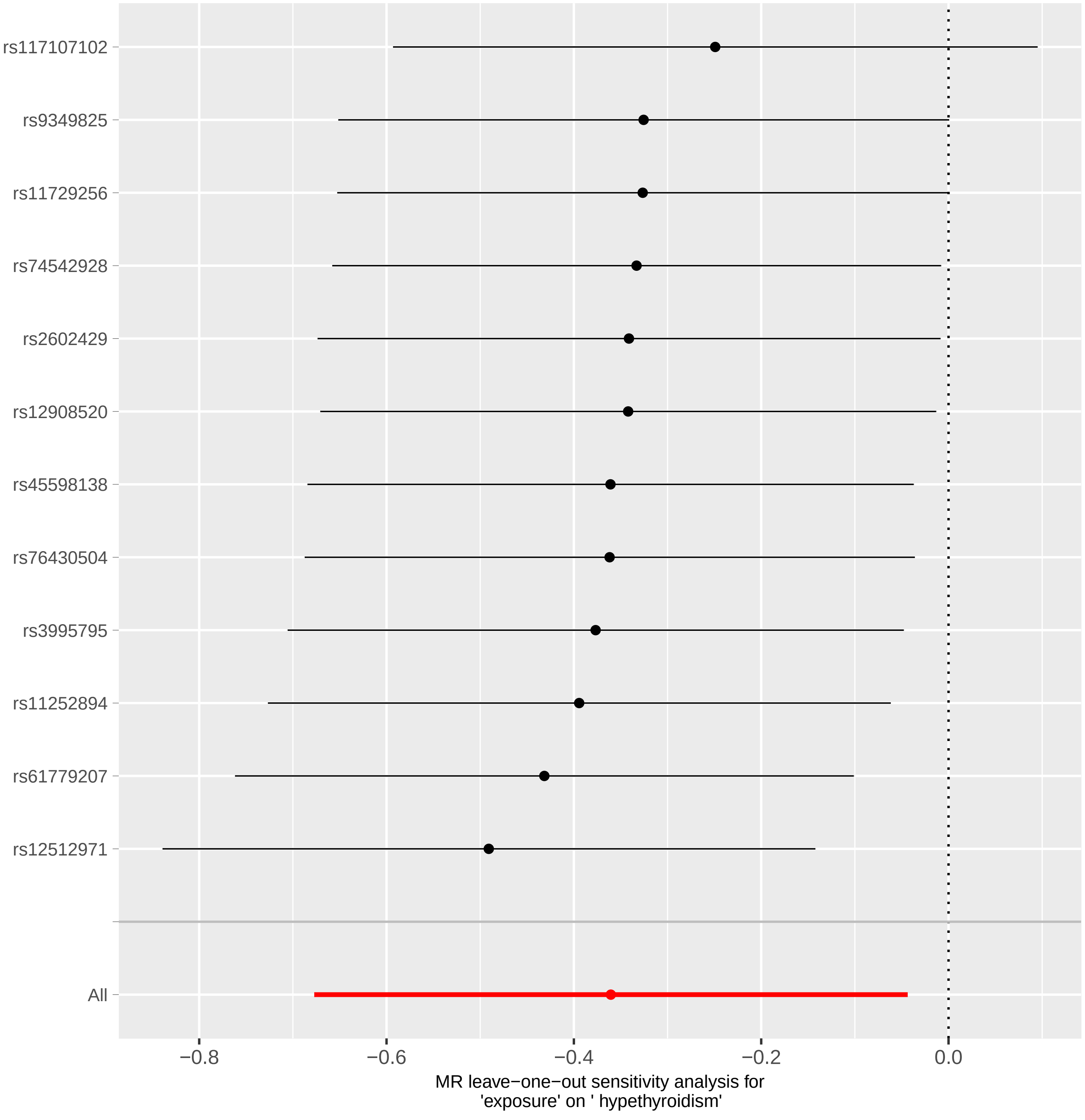
rs117107102
rs9349825
rs11729256
rs74542928
rs2602429
rs12908520
rs45598138
rs76430504
rs3995795
rs11252894
rs61779207
rs12512971
All
−0.8
−0.6
−0.4
−0.2
0.0
MR leave−one−out sensitivity analysis for
'exposure' on ' hypethyroidism'

57

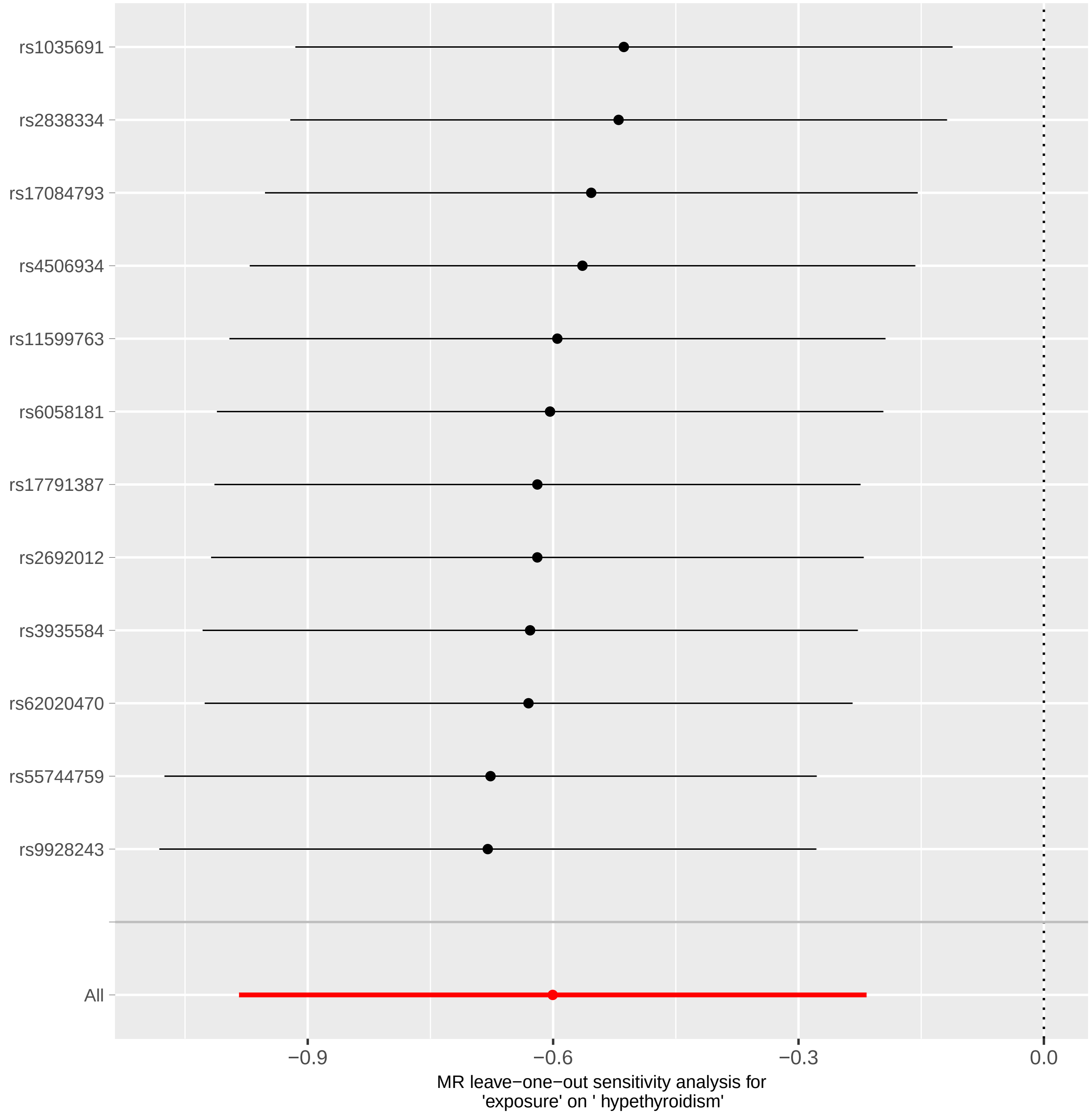

rs1035691
rs2838334
rs17084793
rs4506934
rs11599763
rs6058181
rs17791387
rs2692012
rs3935584
rs62020470
rs55744759
rs9928243
All
−0.9
−0.6
−0.3
0.0
MR leave−one−out sensitivity analysis for
'exposure' on ' hypethyroidism'

58

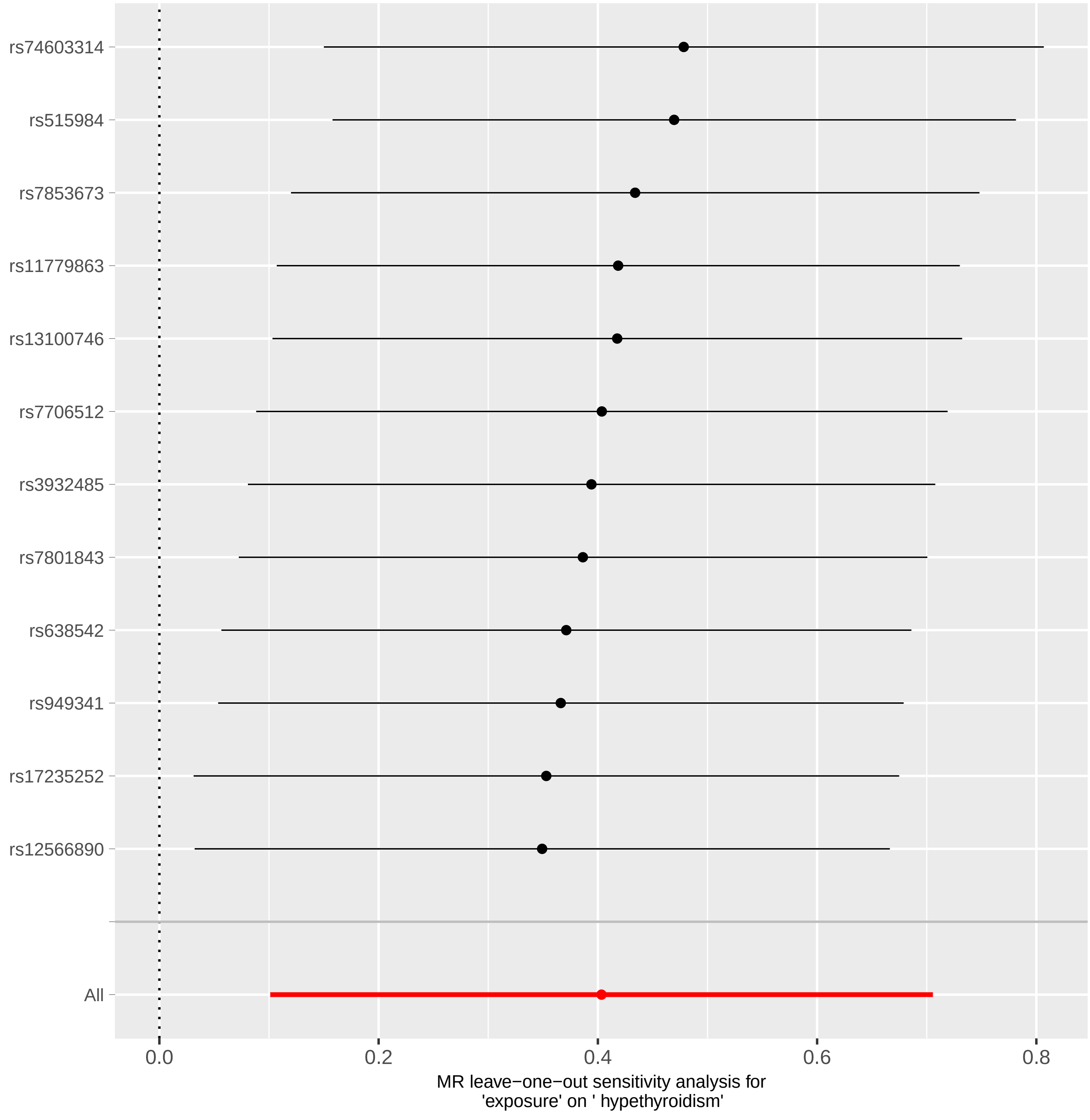

rs74603314
rs515984
rs7853673
rs11779863
rs13100746
rs7706512
rs3932485
rs7801843
rs638542
rs949341
rs17235252
rs12566890
All
0.0
0.2
0.4
0.6
0.8
MR leave−one−out sensitivity analysis for
'exposure' on ' hypethyroidism'

59

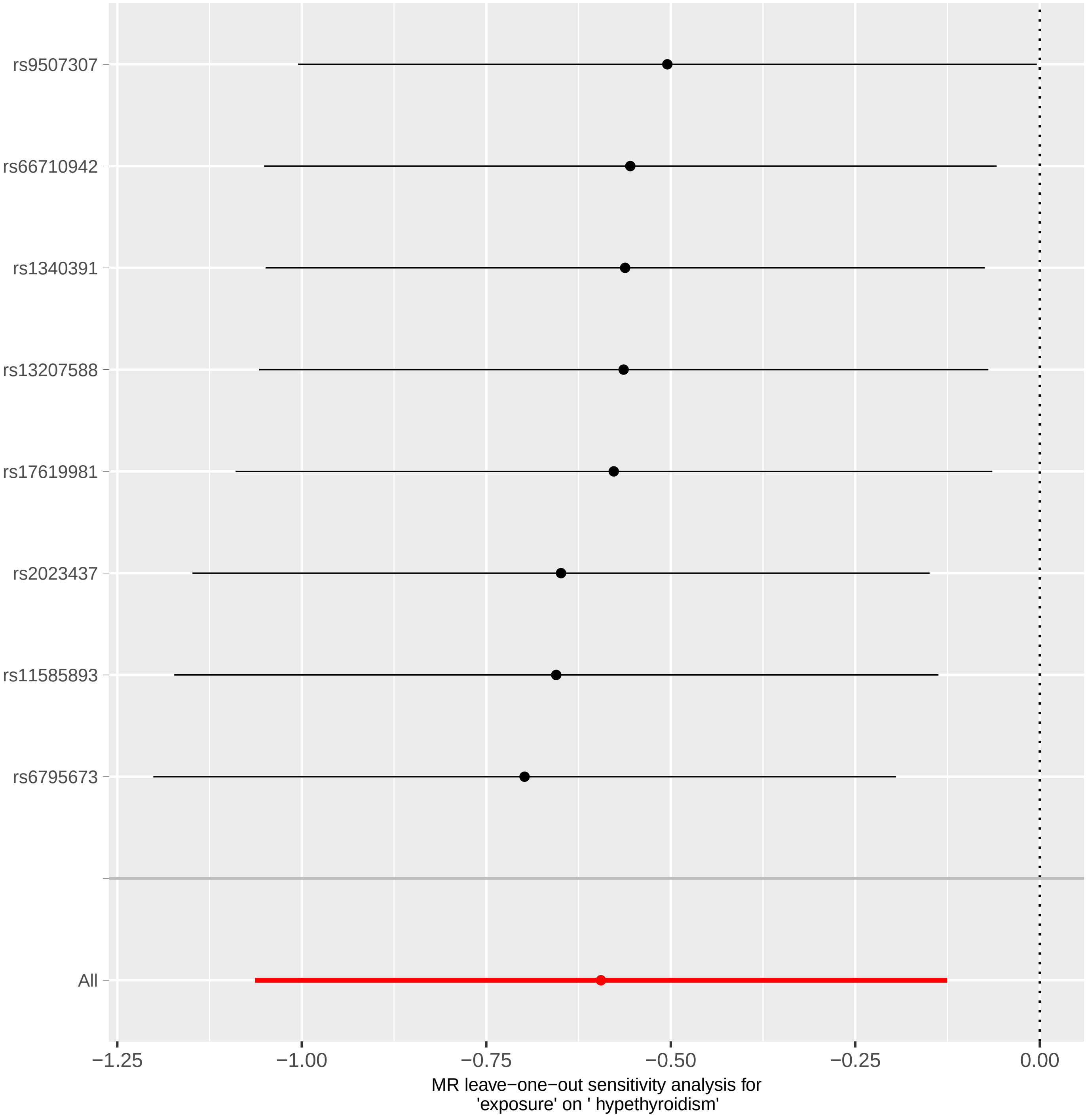
rs9507307
rs66710942
rs1340391
rs13207588
rs17619981
rs2023437
rs11585893
rs6795673
All
−1.25
−1.00
−0.75
−0.50
−0.25
0.00
MR leave−one−out sensitivity analysis for
'exposure' on ' hypethyroidism'

60

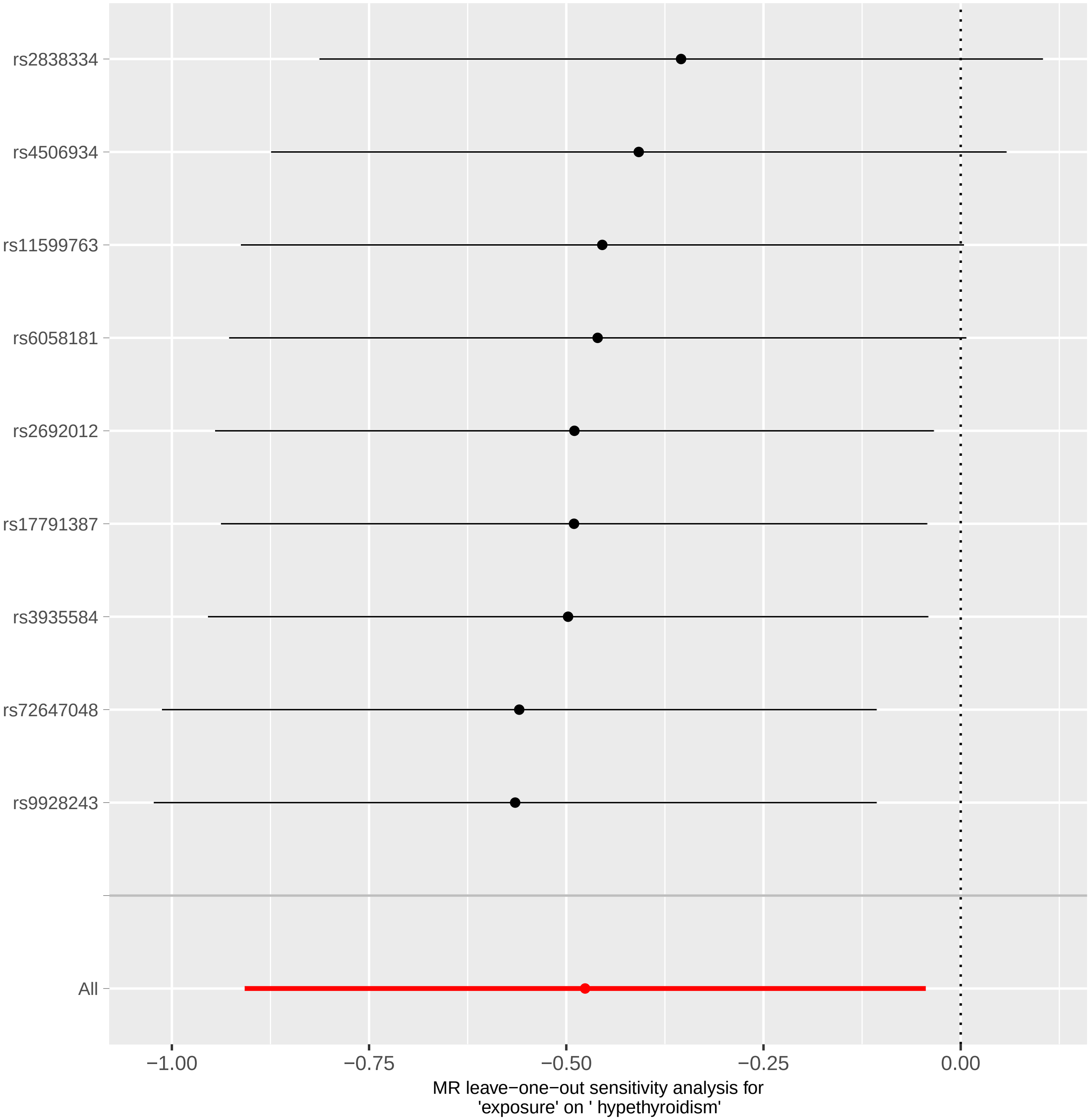

rs2838334
rs4506934
rs11599763
rs6058181
rs2692012
rs17791387
rs3935584
rs72647048
rs9928243
All
−1.00
−0.75
−0.50
−0.25
0.00
MR leave−one−out sensitivity analysis for
'exposure' on ' hypethyroidism'

61

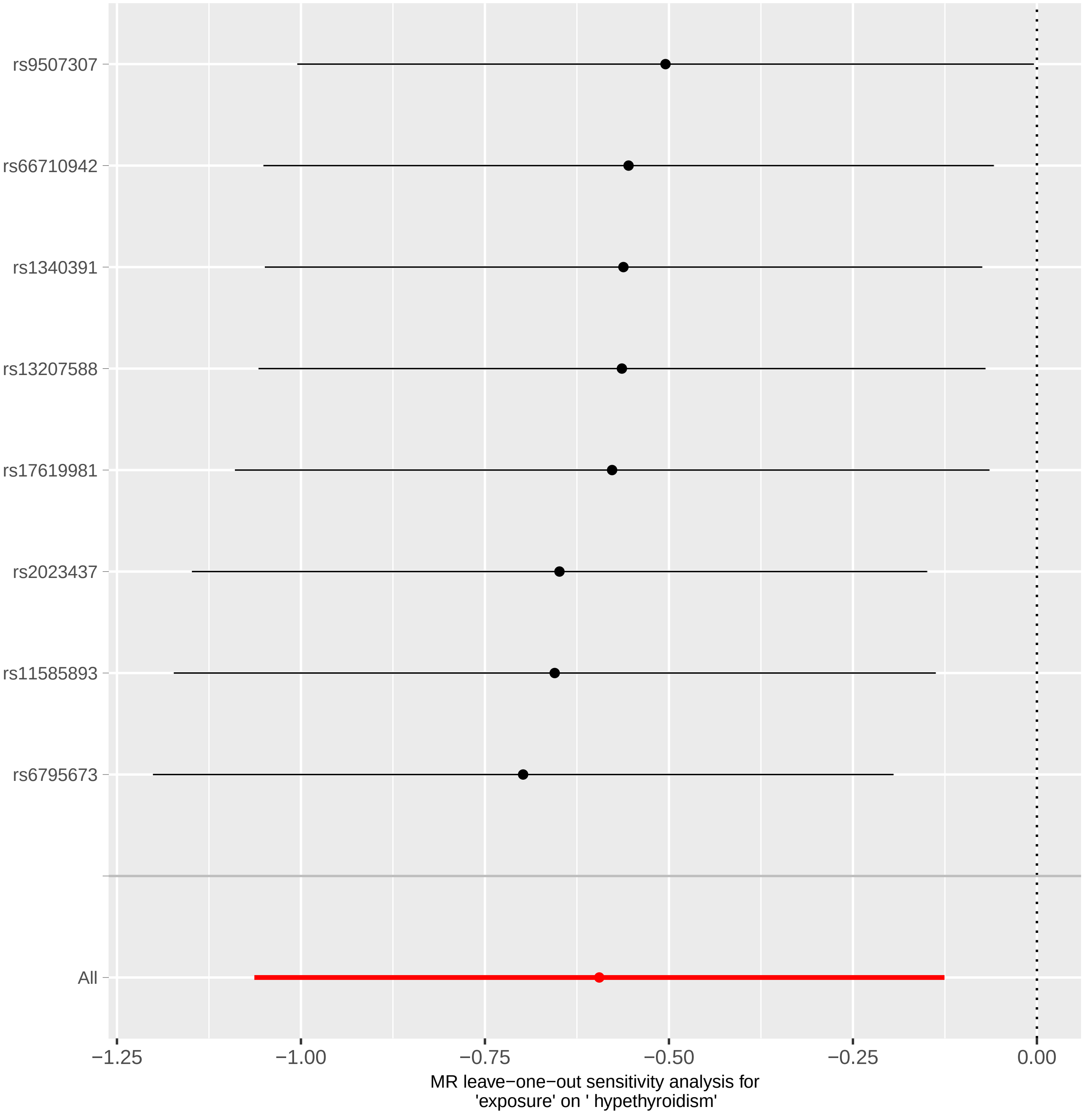
rs9507307
rs66710942
rs1340391
rs13207588
rs17619981
rs2023437
rs11585893
rs6795673
All
−1.25
−1.00
−0.75
−0.50
−0.25
0.00
MR leave−one−out sensitivity analysis for
'exposure' on ' hypethyroidism'

62

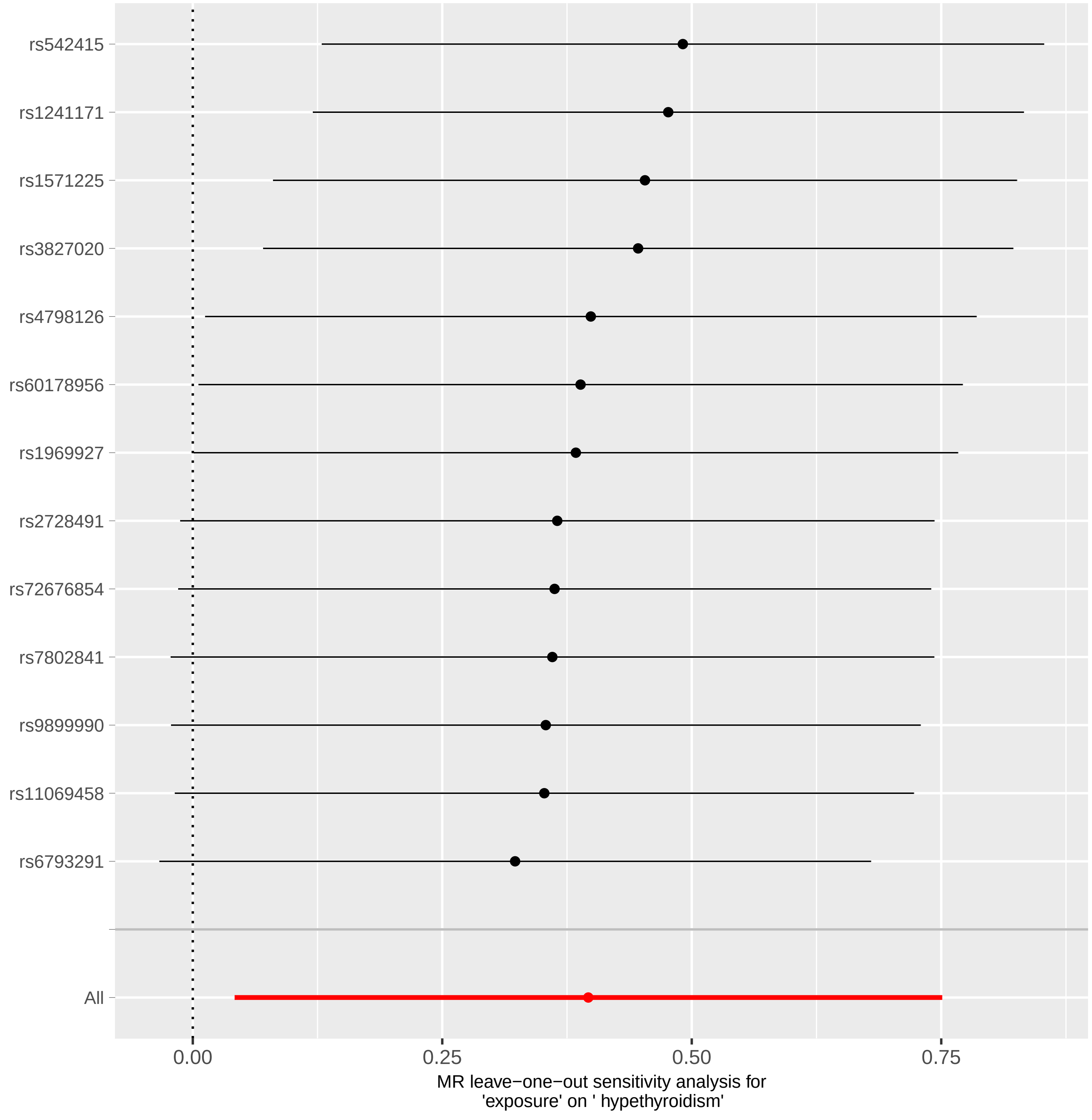

rs542415
rs1241171
rs1571225
rs3827020
rs4798126
rs60178956
rs1969927
rs2728491
rs72676854
rs7802841
rs9899990
rs11069458
rs6793291
All
0.00
0.25
0.50
0.75
MR leave−one−out sensitivity analysis for
'exposure' on ' hypethyroidism'

63

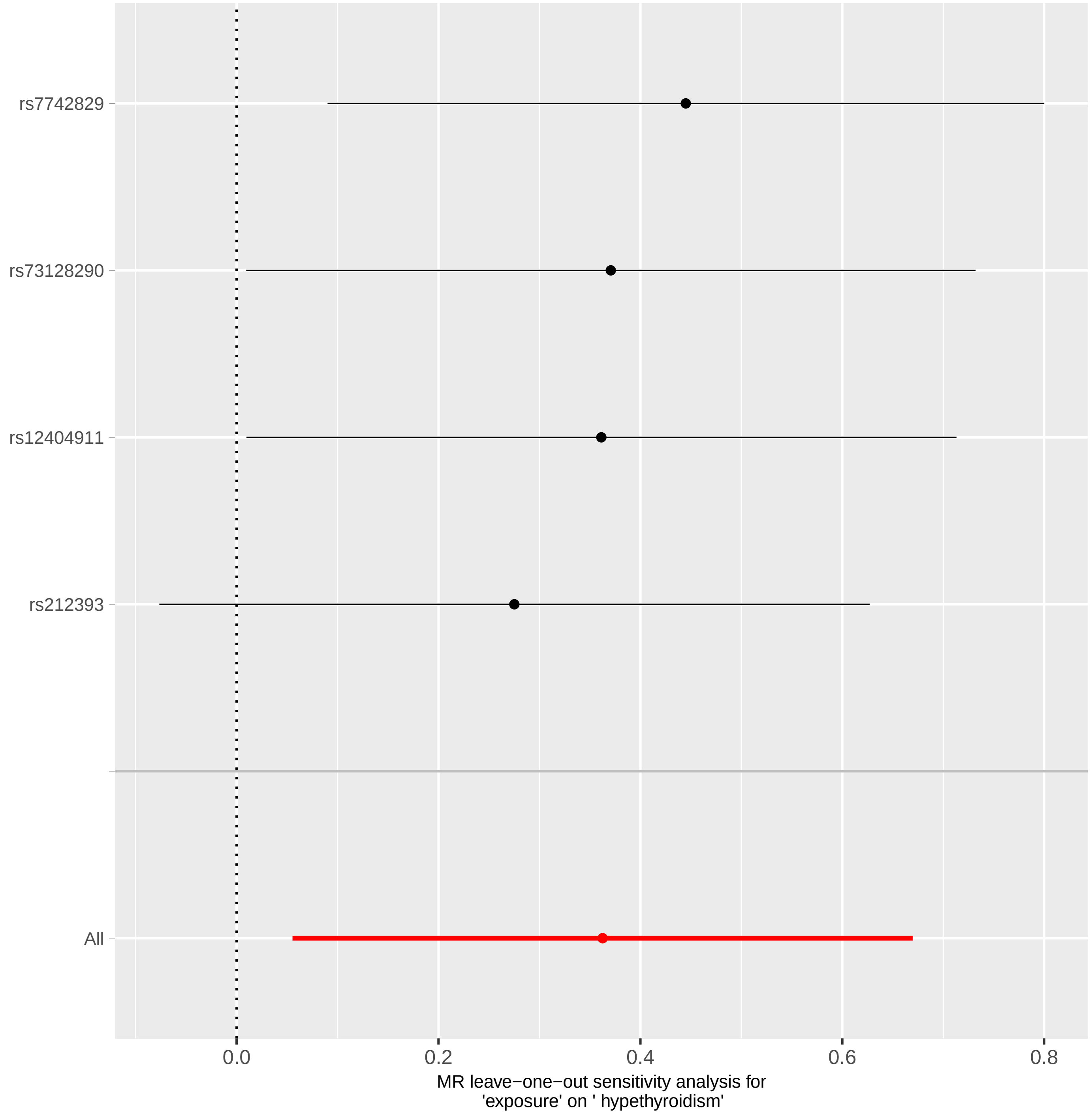
rs7742829
rs73128290
rs12404911
rs212393
All
0.0
0.2
0.4
0.6
0.8
MR leave−one−out sensitivity analysis for
'exposure' on ' hypethyroidism'

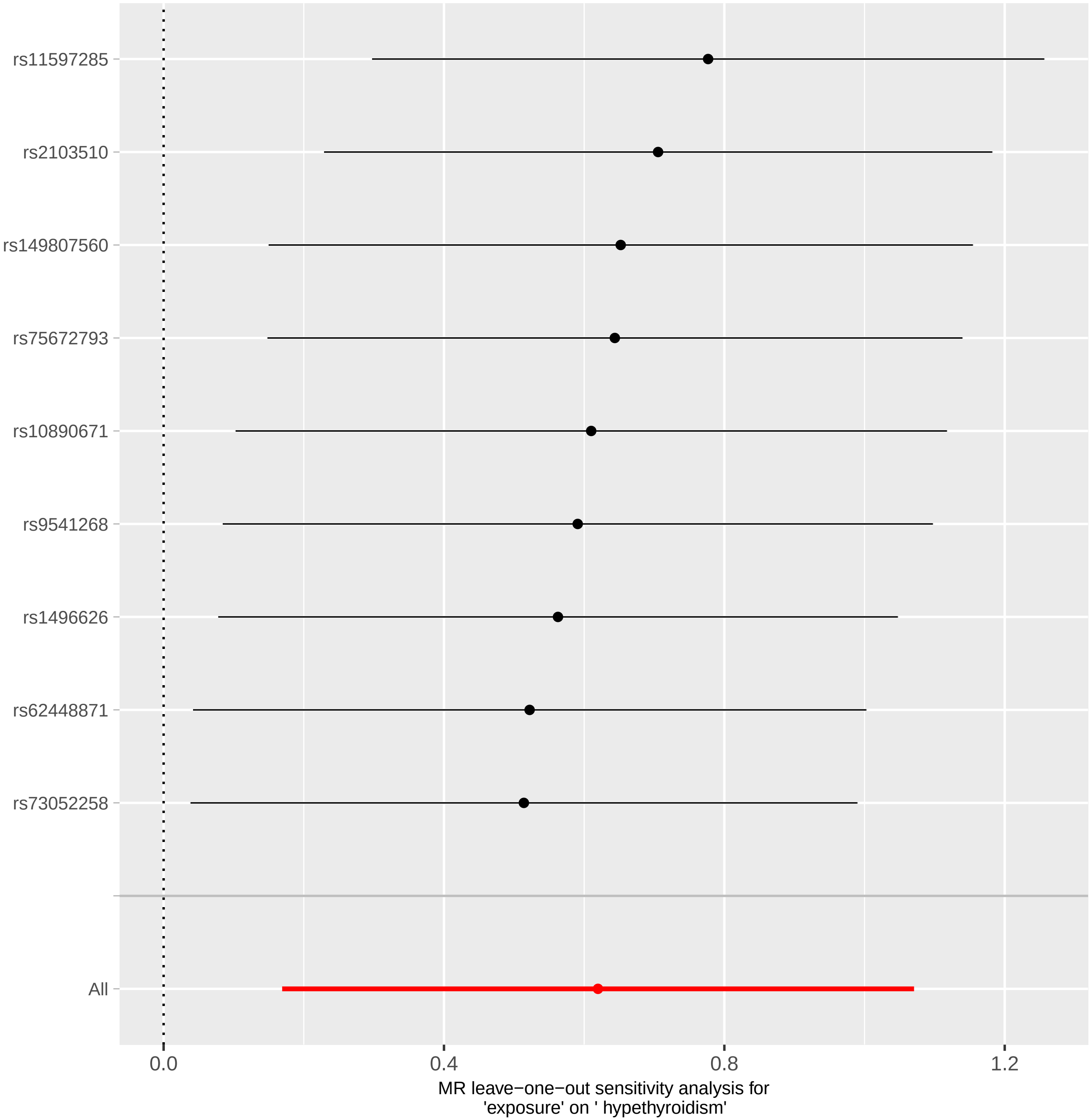
rs11597285
rs2103510
rs149807560
rs75672793
rs10890671
rs9541268
rs1496626
rs62448871
rs73052258
All
0.0
0.4
0.8
1.2
MR leave−one−out sensitivity analysis for
'exposure' on ' hypethyroidism'

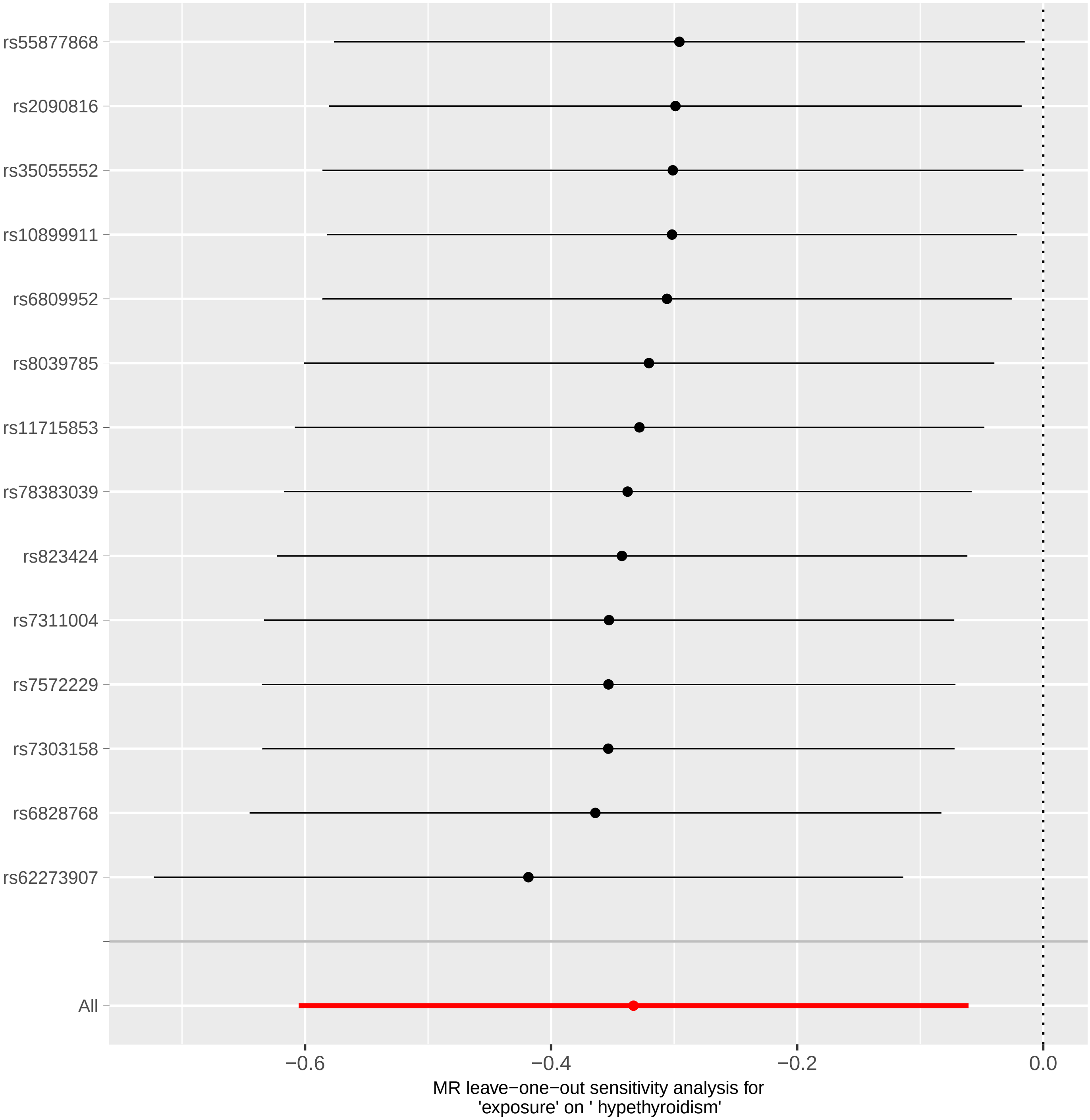
rs55877868
rs2090816
rs35055552
rs10899911
rs6809952
rs8039785
rs11715853
rs78383039
rs823424
rs7311004
rs7572229
rs7303158
rs6828768
rs62273907
All
−0.6
−0.4
−0.2
0.0
MR leave−one−out sensitivity analysis for
'exposure' on ' hypethyroidism'

66

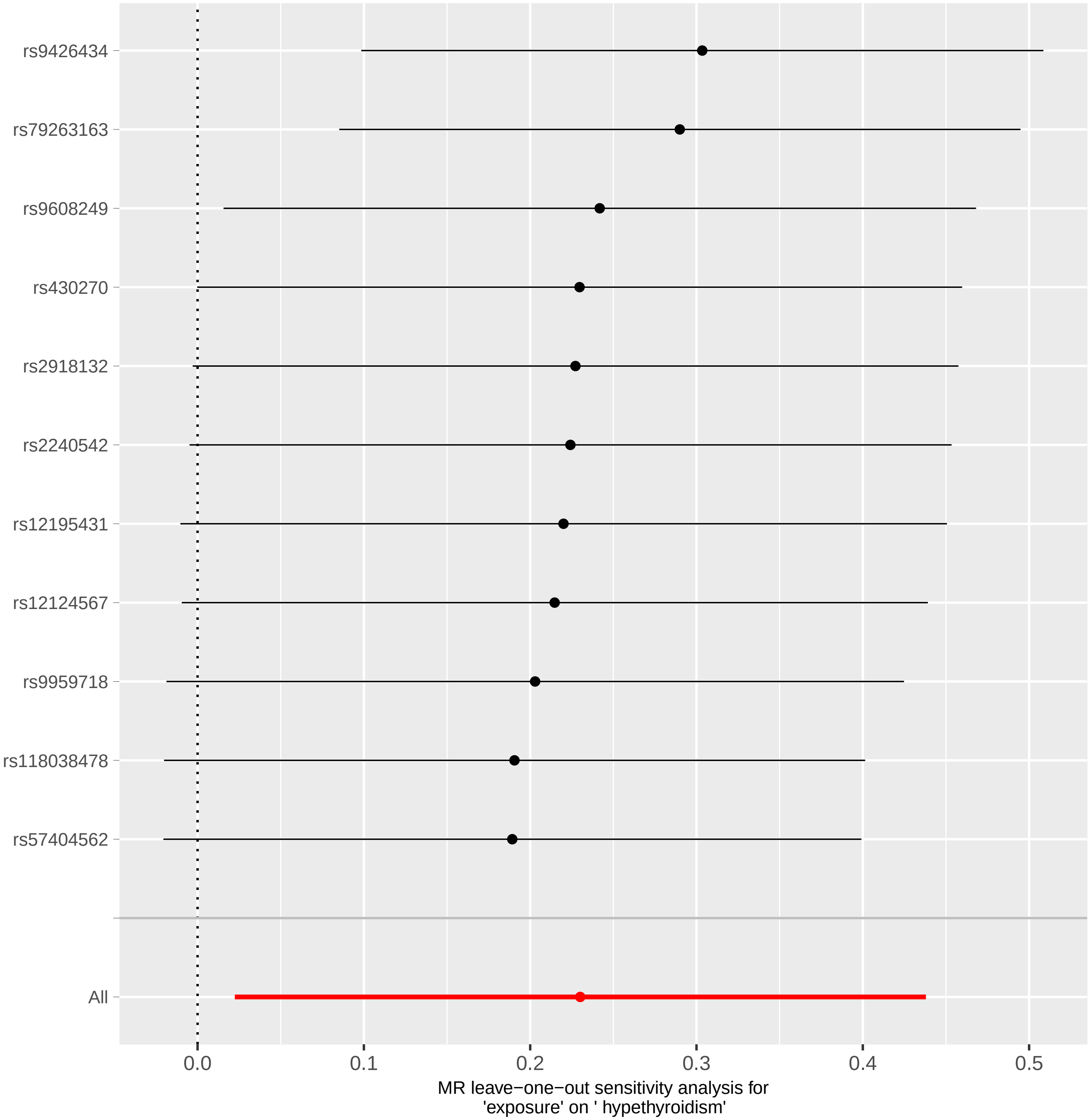
rs9426434
rs79263163
rs9608249
rs430270
rs2918132
rs2240542
rs12195431
rs12124567
rs9959718
rs118038478
rs57404562
All
0.0
0.1
0.2
0.3
0.4
0.5
MR leave−one−out sensitivity analysis for
'exposure' on ' hypethyroidism'

67

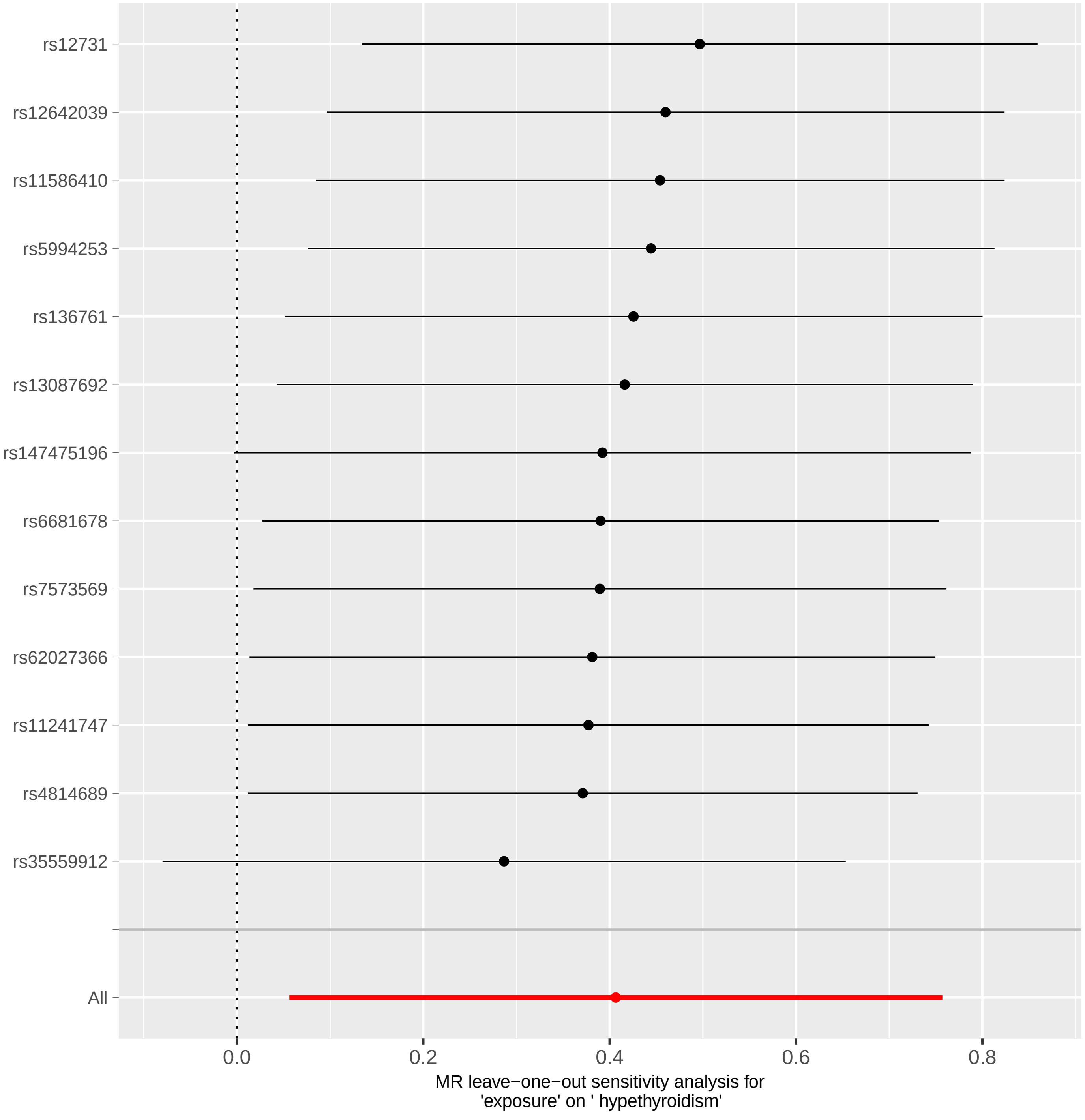

rs12731
rs12642039
rs11586410
rs5994253
rs136761
rs13087692
rs147475196
rs6681678
rs7573569
rs62027366
rs11241747
rs4814689
rs35559912
All
0.0
0.2
0.4
0.6
0.8
MR leave−one−out sensitivity analysis for
'exposure' on ' hypethyroidism'

68

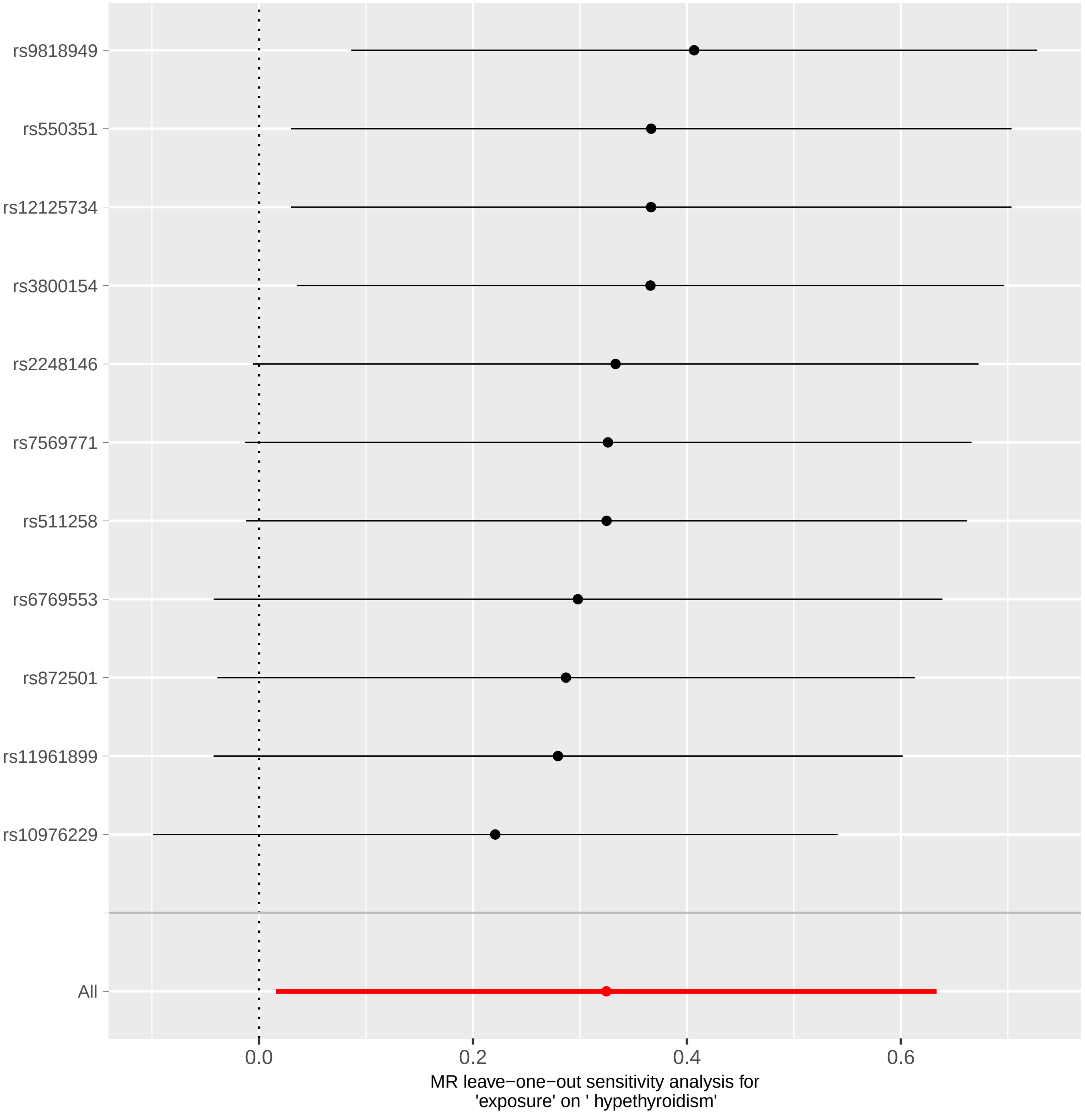

rs9818949
rs550351
rs12125734
rs3800154
rs2248146
rs7569771
rs511258
rs6769553
rs872501
rs11961899
rs10976229
All
0.0
0.2
0.4
0.6
MR leave−one−out sensitivity analysis for
'exposure' on ' hypethyroidism'

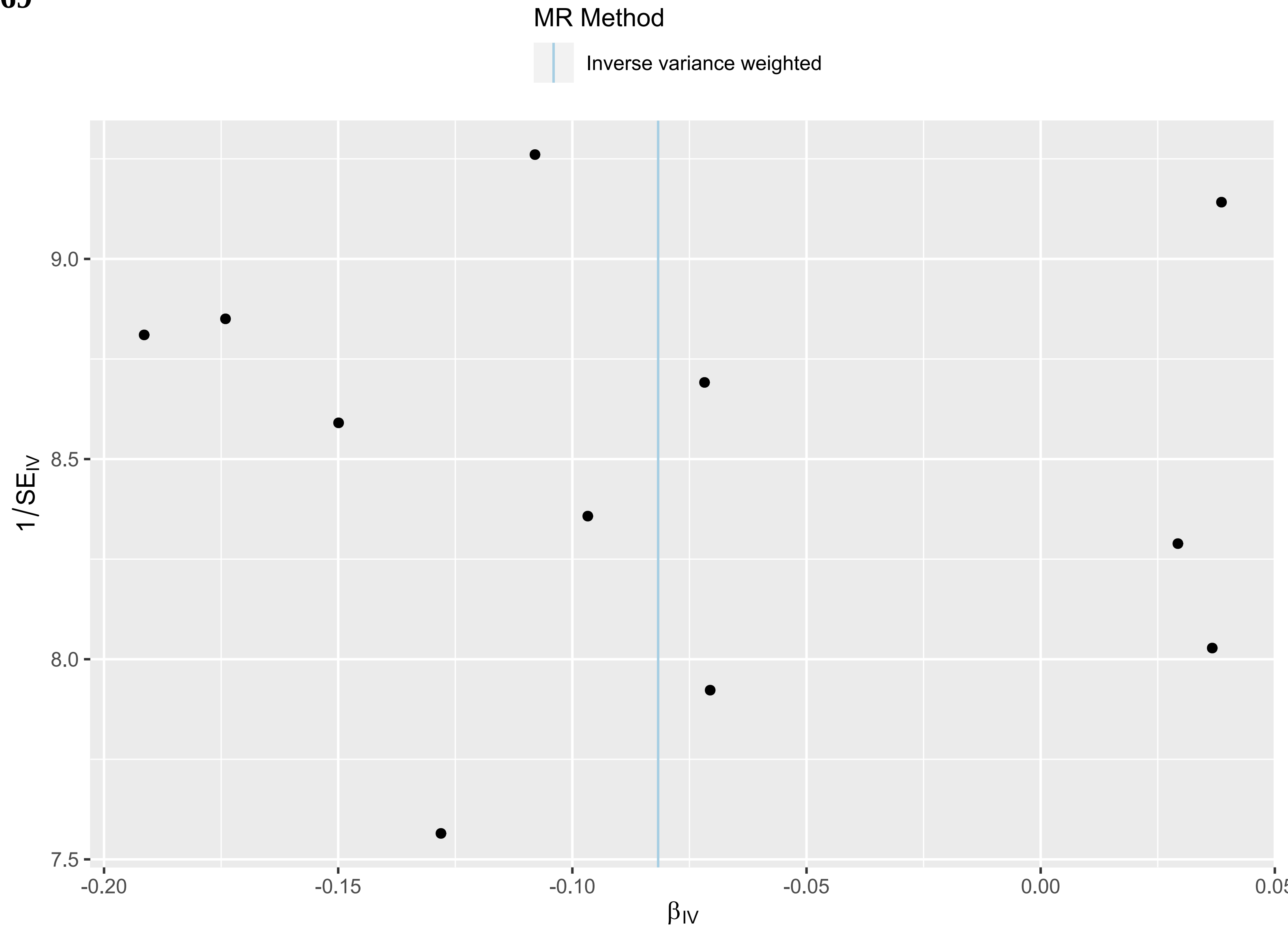
MR Method
Inverse variance weighted
9.0
8.5
8.0
7.5
1/SE$_{IV}$
-0.20
-0.15
-0.10
-0.05
0.00
0.05
β$_{IV}$

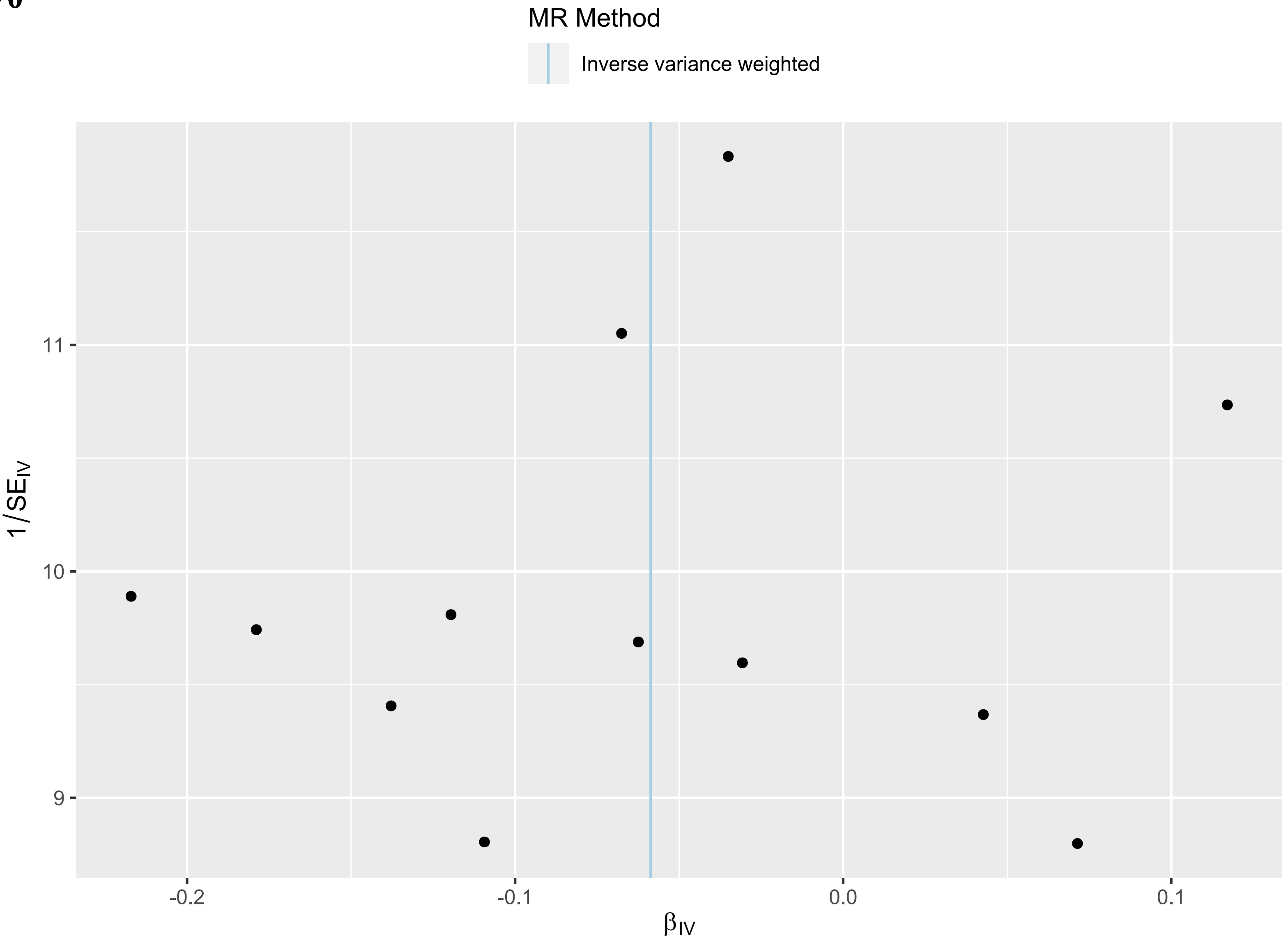
MR Method
Inverse variance weighted
11
10
9
$1/SE_{IV}$
-0.2
-0.1
0.0
0.1
β_{IV}

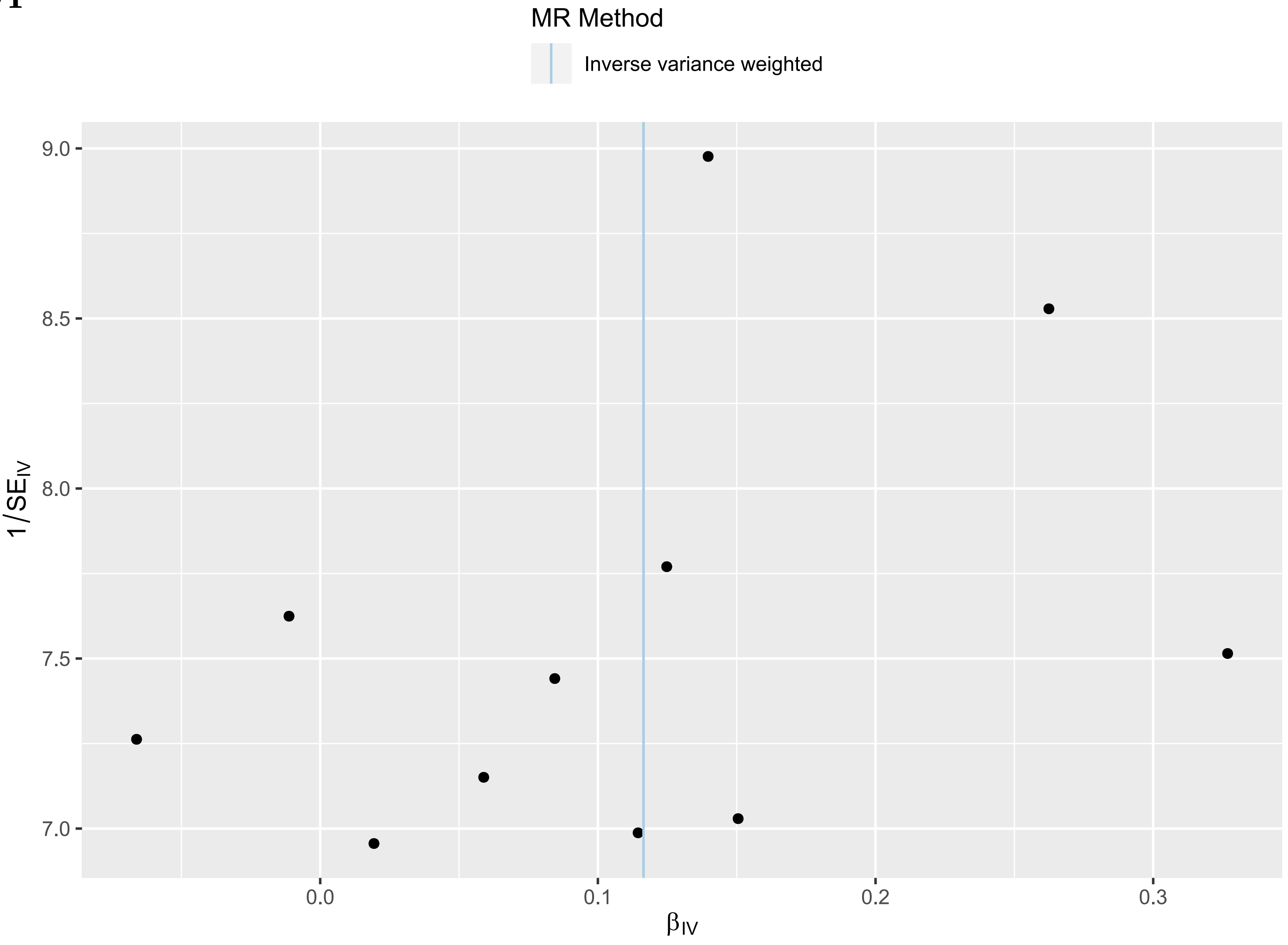
MR Method
Inverse variance weighted
9.0
8.5
8.0
7.5
7.0
1/SE$_{IV}$
0.0
0.1
0.2
0.3
β_{IV}

72

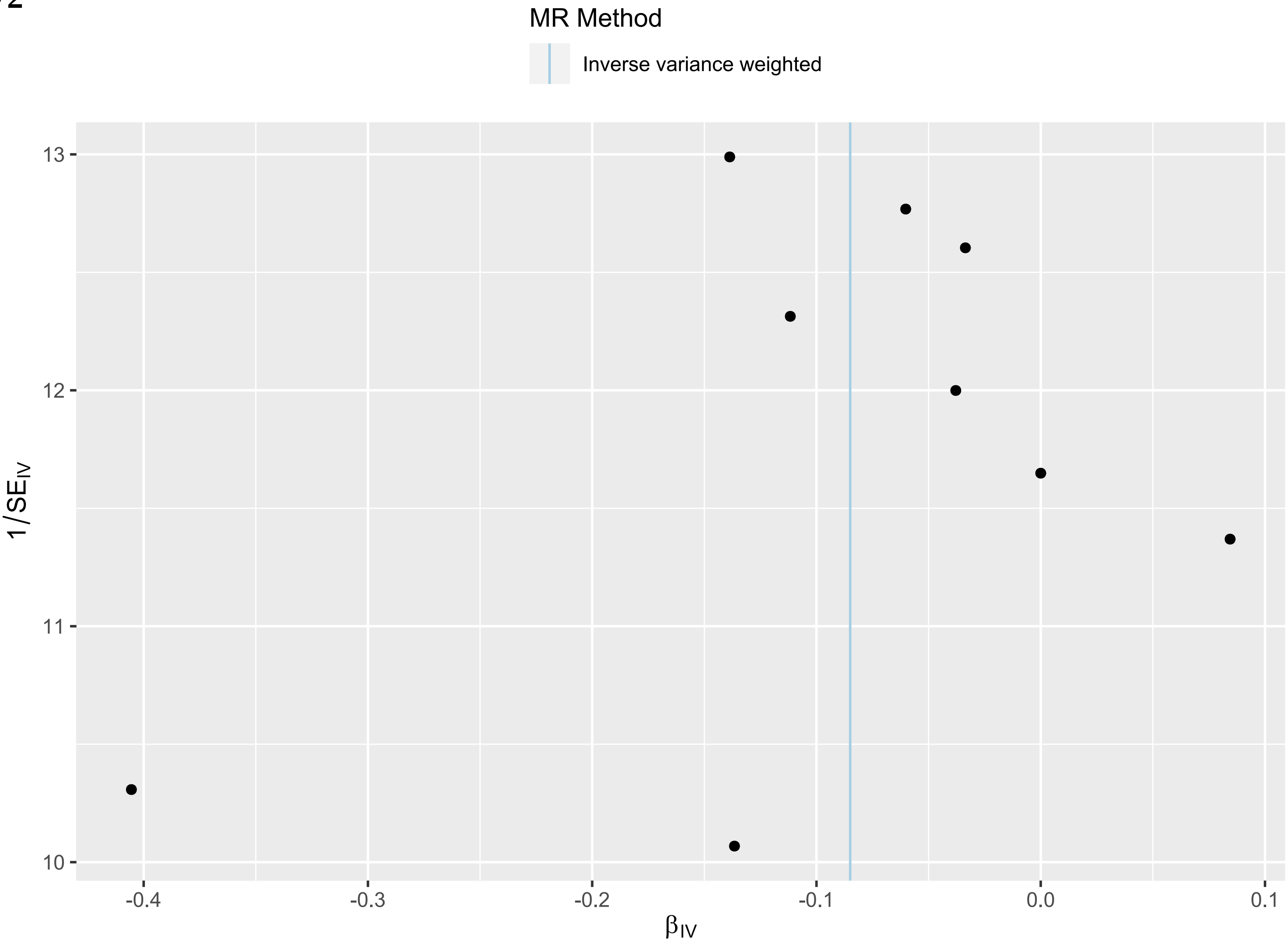
MR Method
Inverse variance weighted
13
12
11
10
1/SE$_{IV}$
-0.4
-0.3
-0.2
-0.1
0.0
0.1
β$_{IV}$

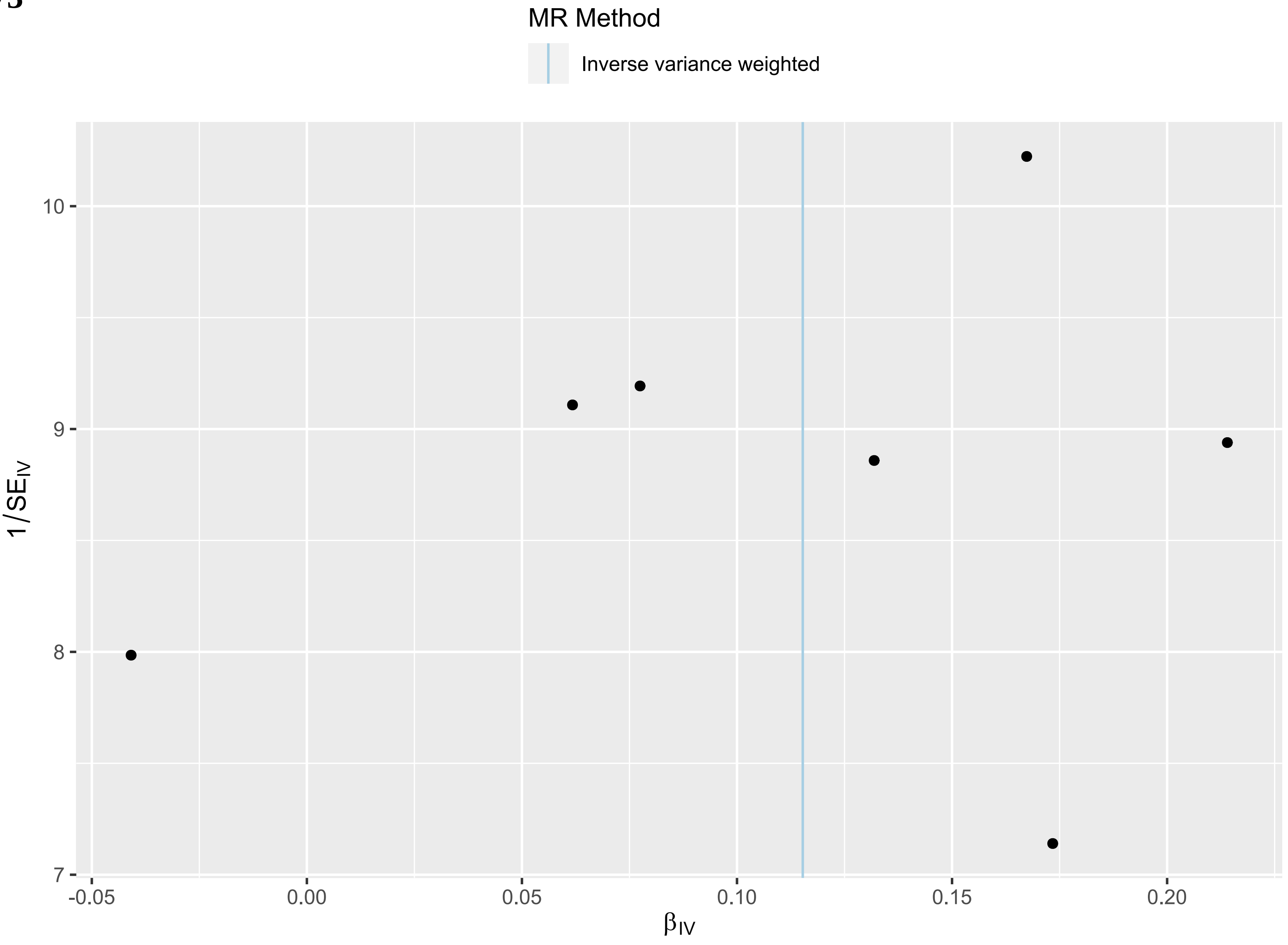
MR Method
Inverse variance weighted
1/SE_IV
β_IV
10
9
8
7
-0.05
0.00
0.05
0.10
0.15
0.20

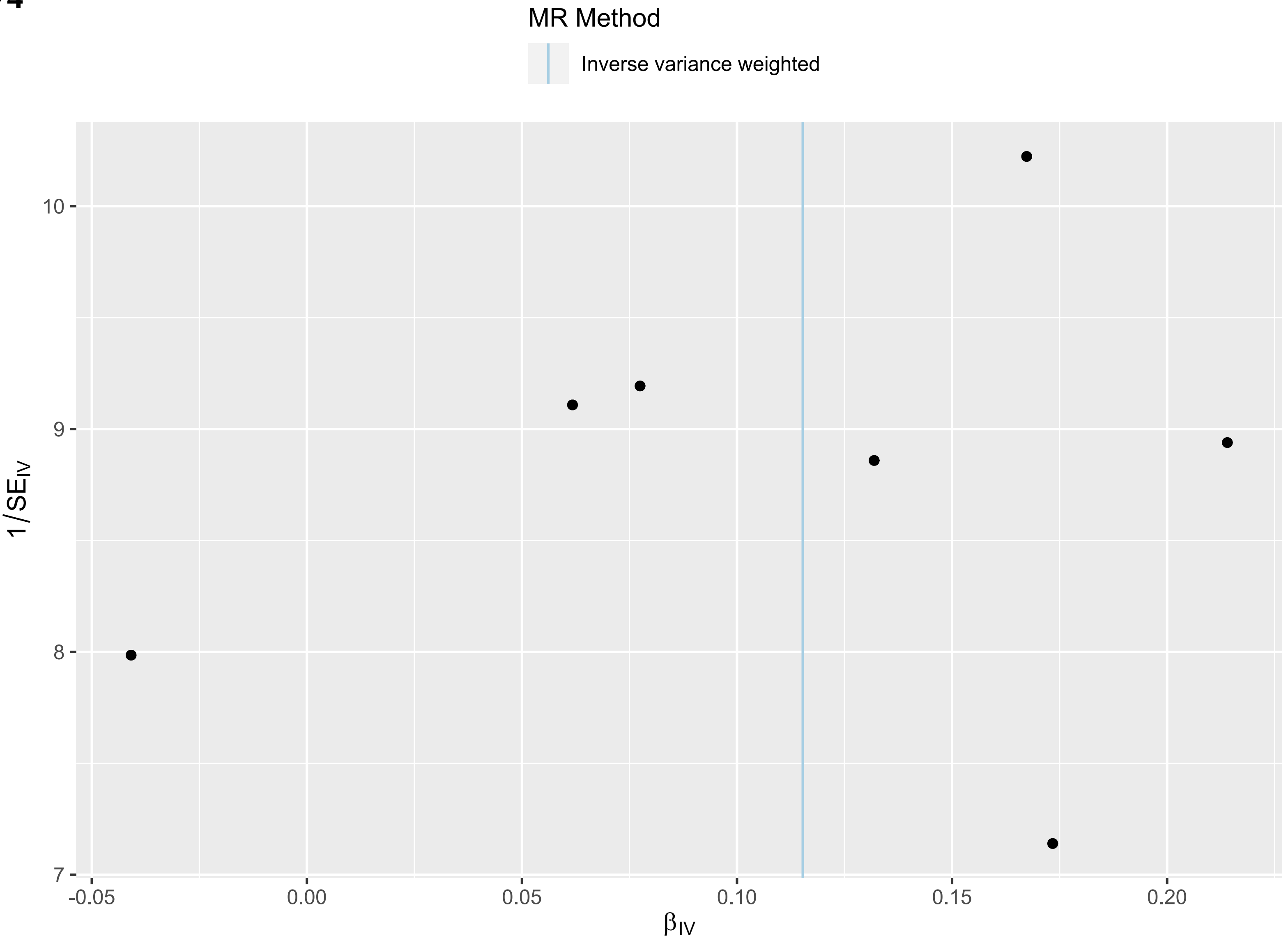
MR Method
Inverse variance weighted
10
9
8
7
1/SE$_{IV}$
-0.05
0.00
0.05
0.10
0.15
0.20
β$_{IV}$

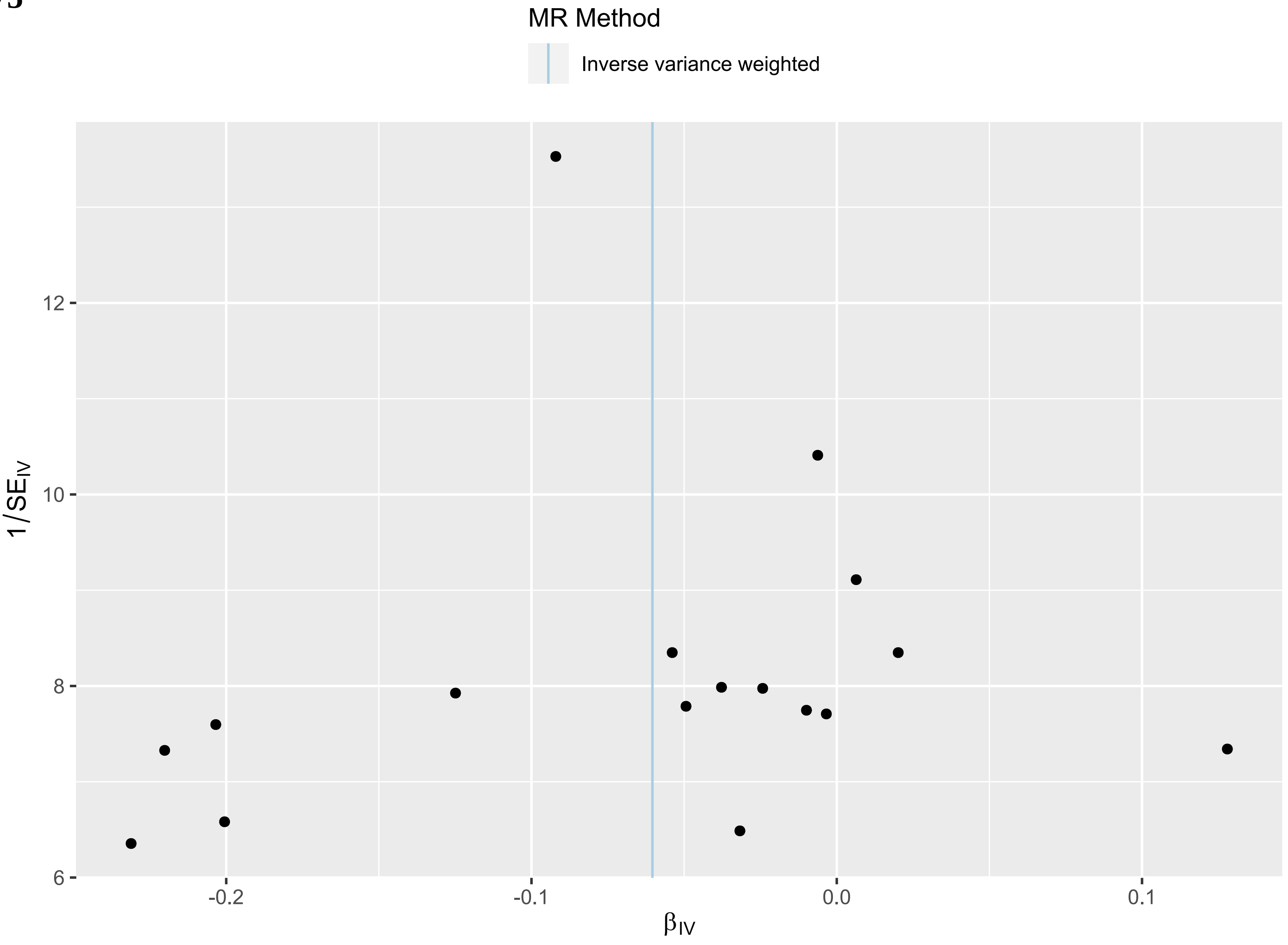
MR Method
Inverse variance weighted
12
10
8
6
1/SE$_{IV}$
-0.2
-0.1
0.0
0.1
β$_{IV}$

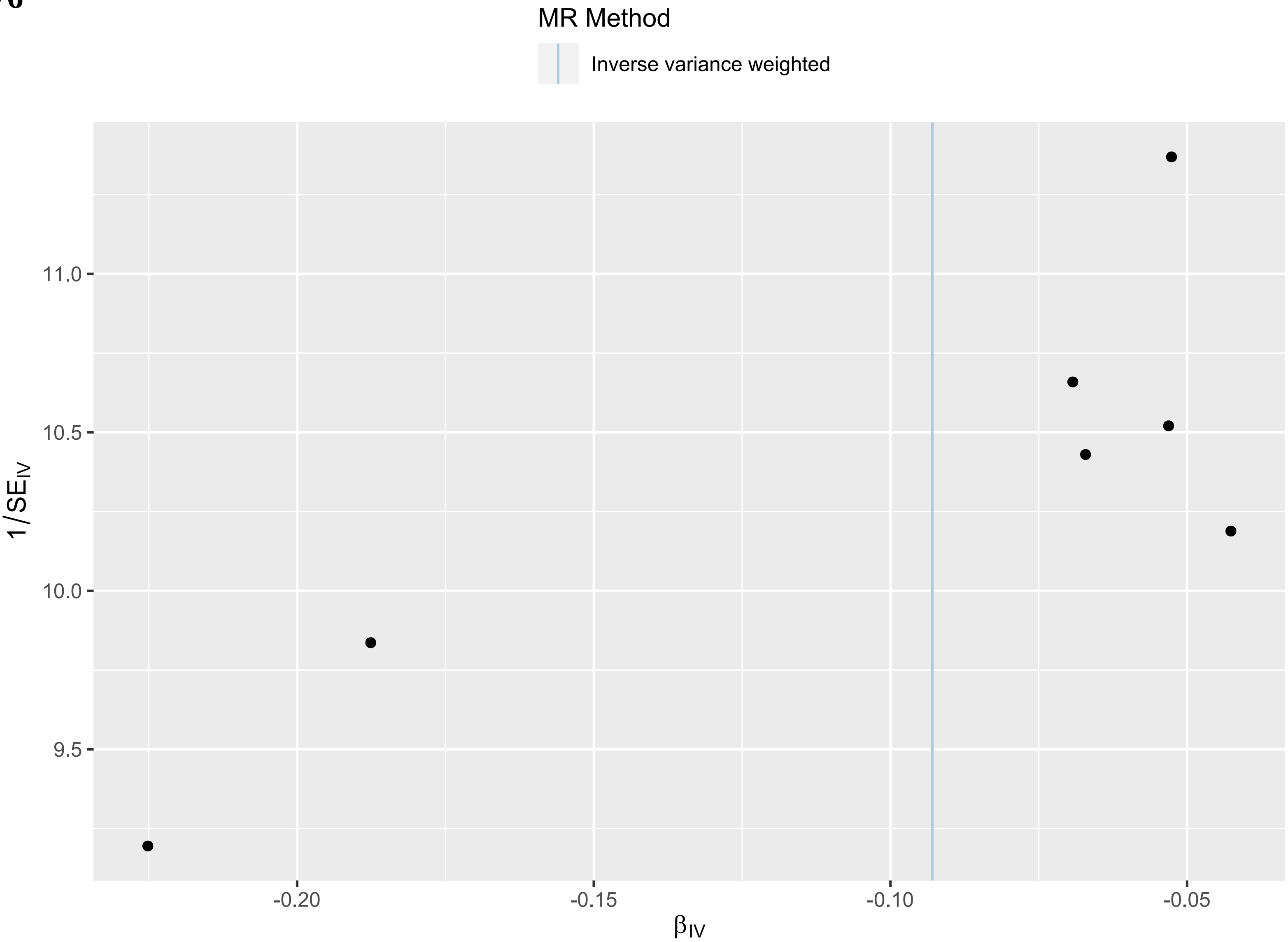
MR Method
Inverse variance weighted
11.0
10.5
10.0
9.5
1/SE$_{IV}$
-0.20
-0.15
-0.10
-0.05
β$_{IV}$

77

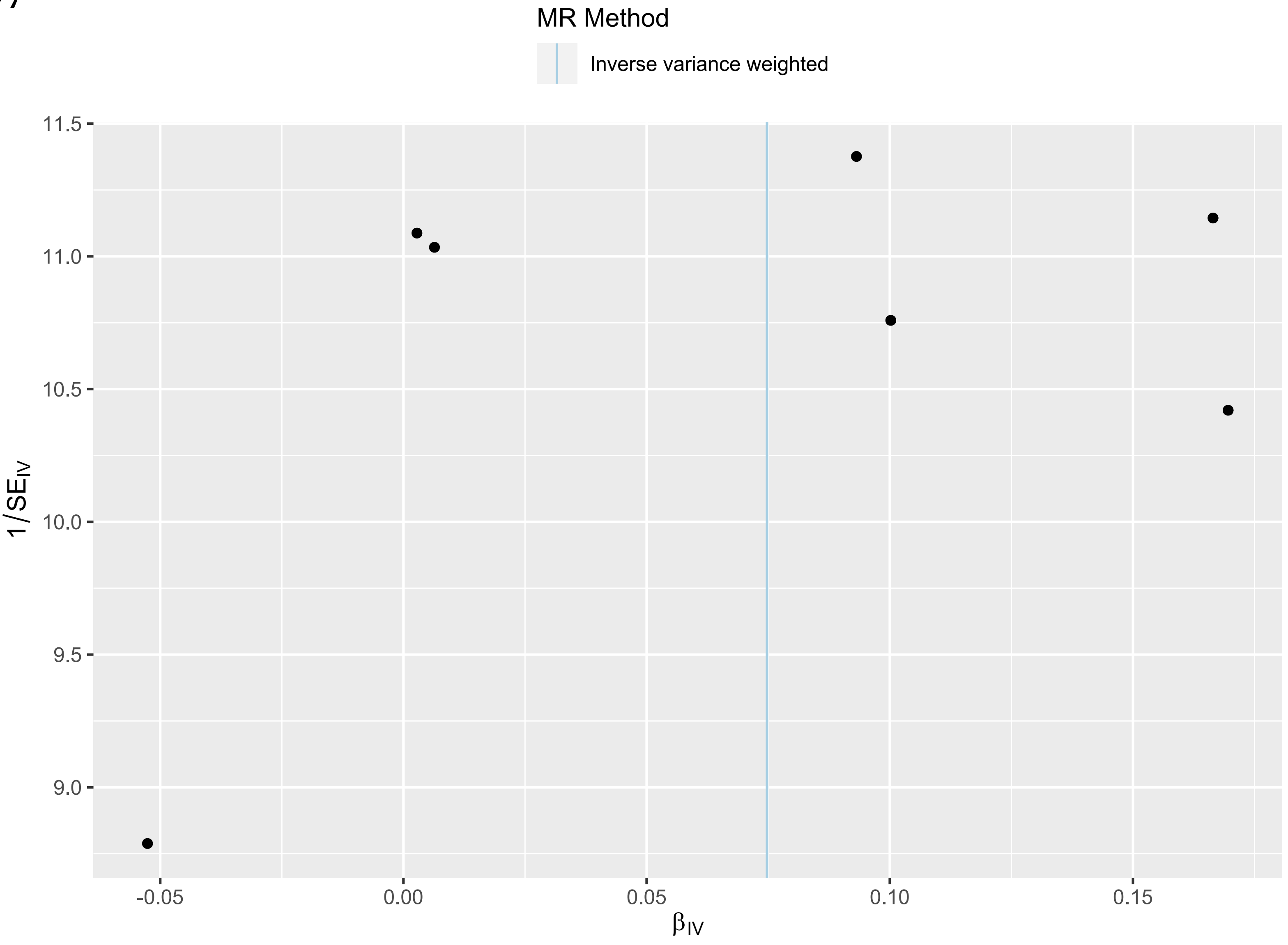
MR Method
Inverse variance weighted
11.5
11.0
10.5
10.0
9.5
9.0
1/SE$_{IV}$
-0.05
0.00
0.05
0.10
0.15
β$_{IV}$

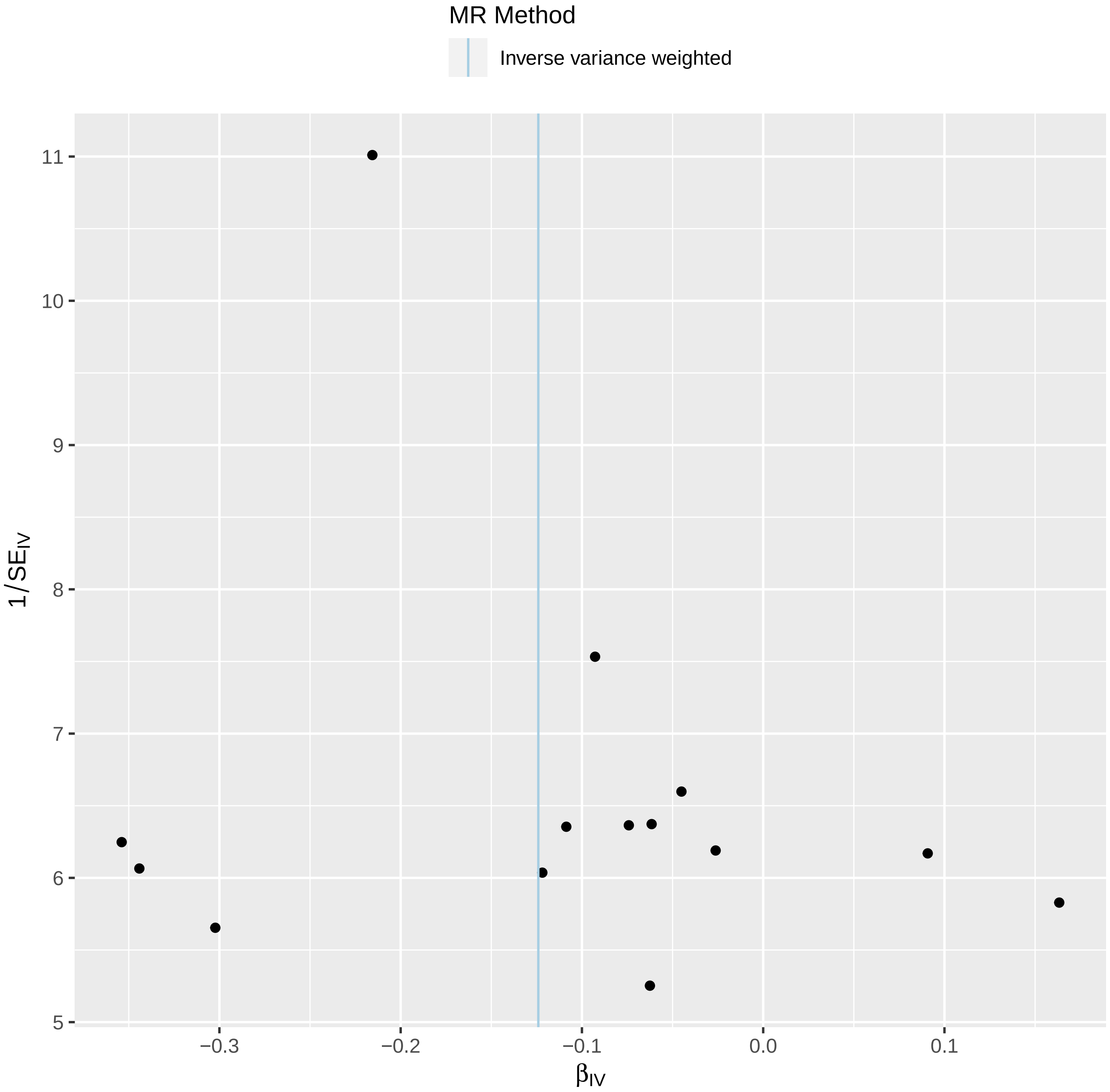
MR Method
Inverse variance weighted
11
10
9
8
7
6
5
1/SE$_{IV}$
−0.3
−0.2
−0.1
0.0
0.1
β$_{IV}$

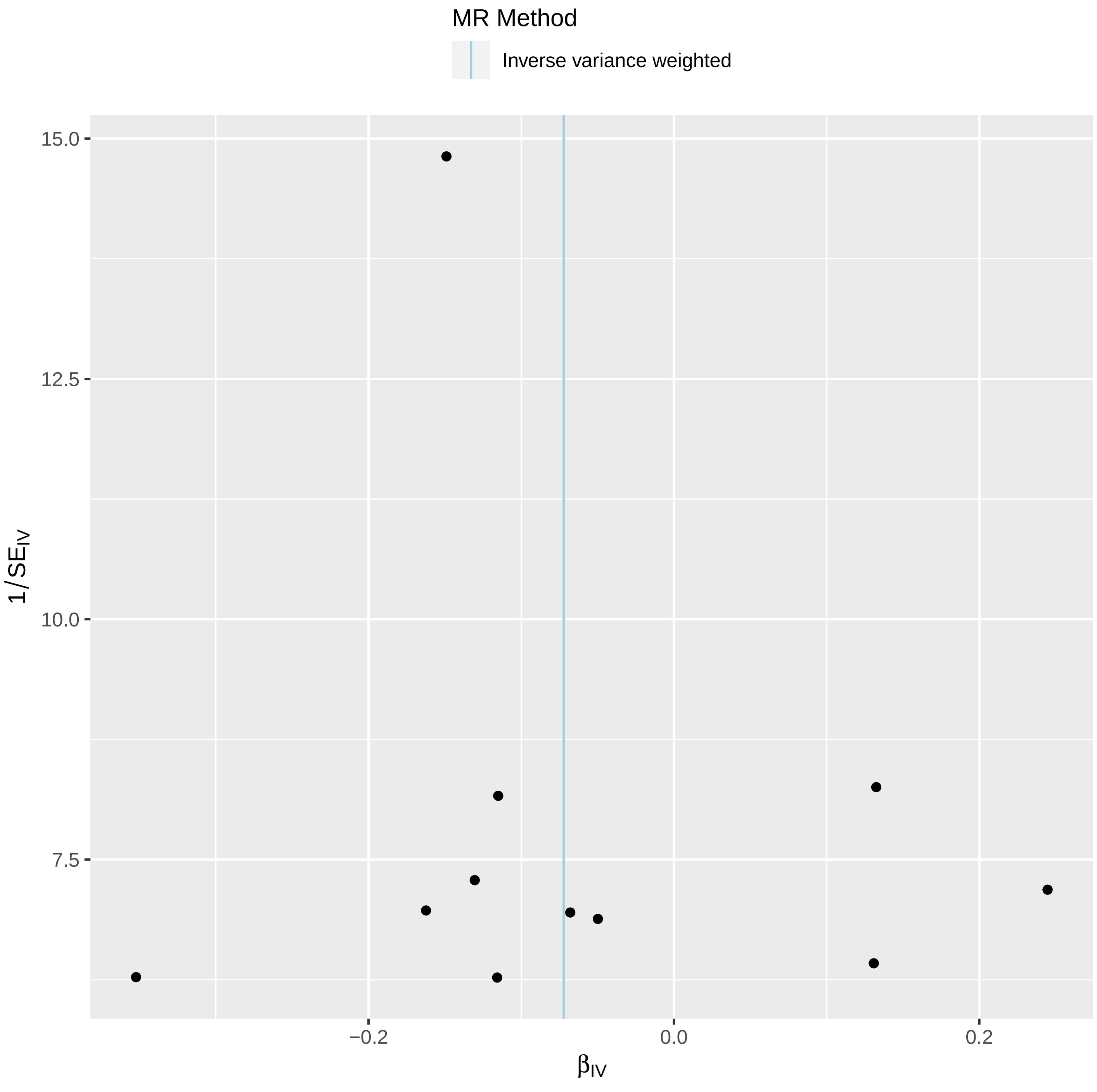
MR Method
Inverse variance weighted
15.0
12.5
10.0
7.5
1/SE_IV
−0.2
0.0
0.2
β_IV

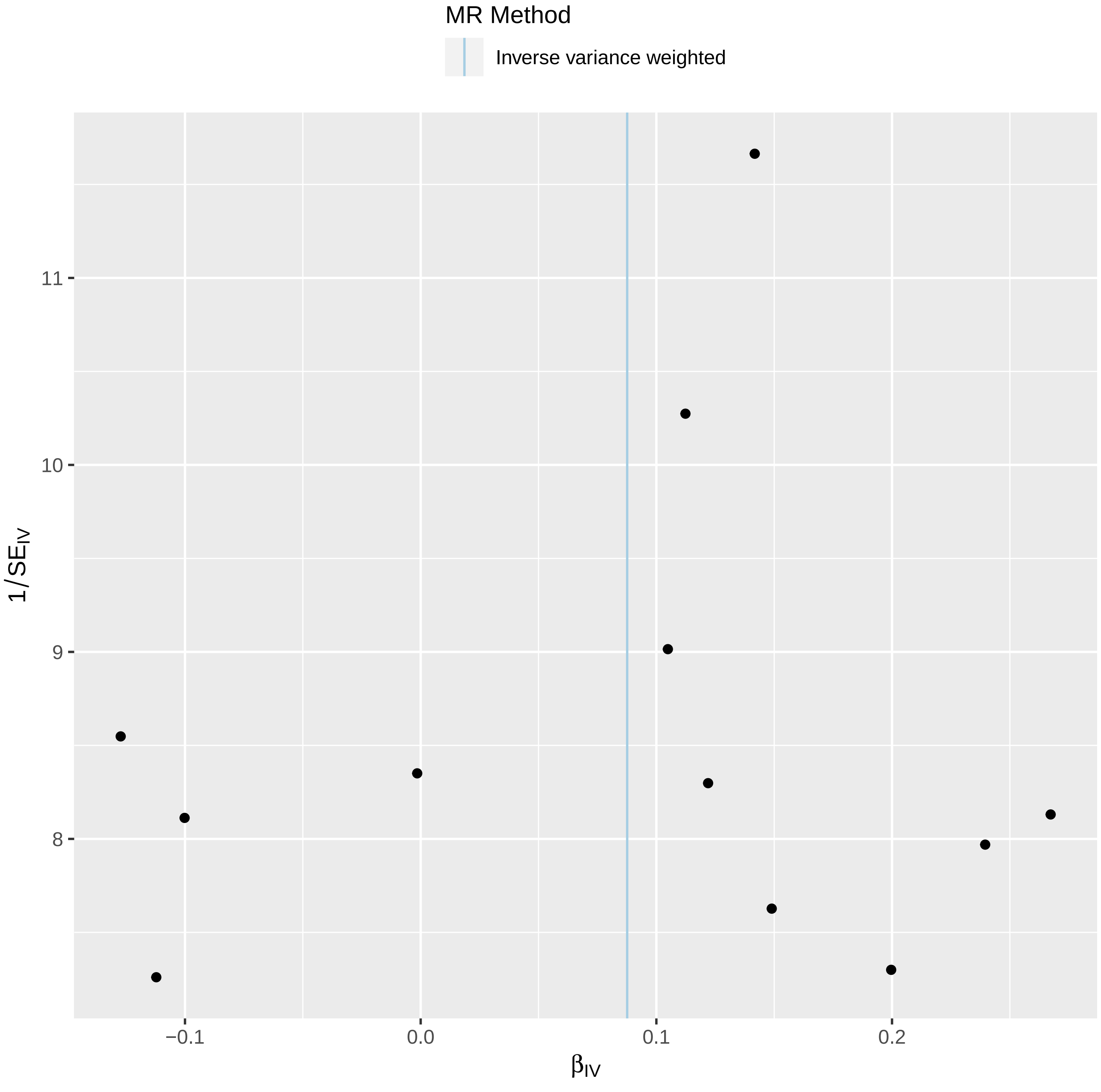
MR Method
Inverse variance weighted
1/SE_IV
β_IV
11
10
9
8
−0.1
0.0
0.1
0.2

81

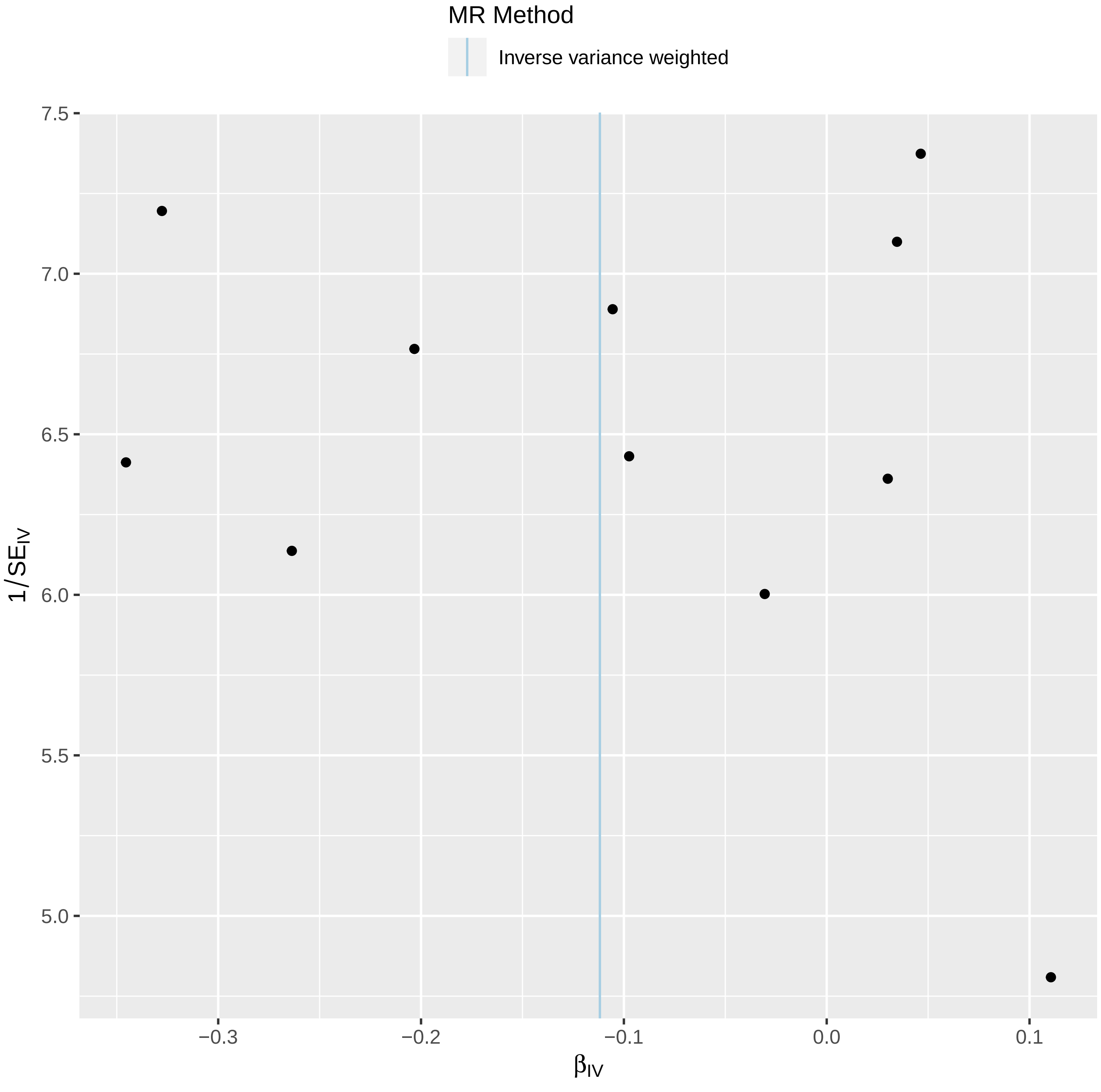
MR Method
Inverse variance weighted
7.5
7.0
6.5
6.0
5.5
5.0
1/SE$_{IV}$
−0.3
−0.2
−0.1
0.0
0.1
β$_{IV}$

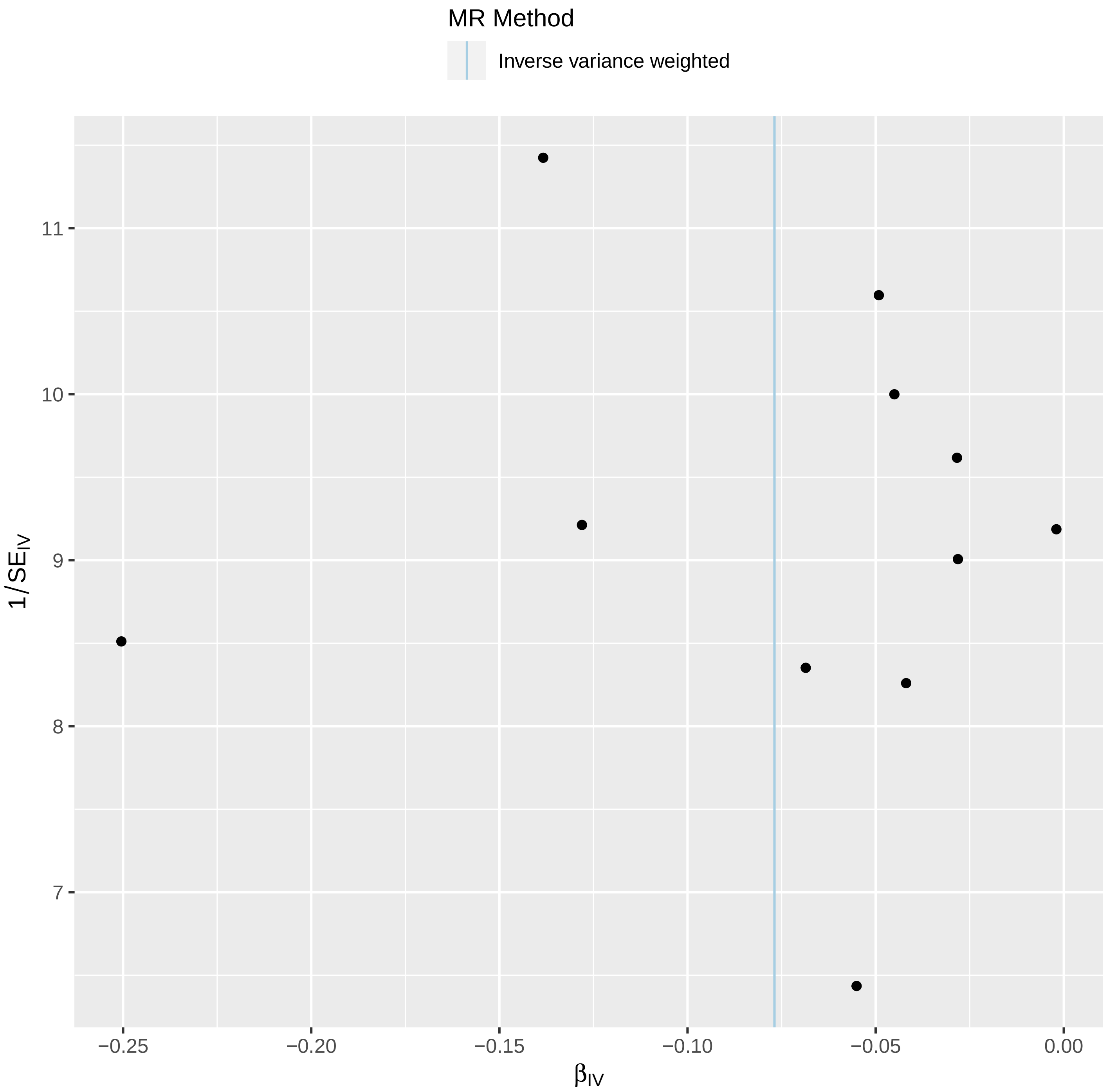
MR Method
Inverse variance weighted
1/SE_IV
β_IV
11
10
9
8
7
−0.25
−0.20
−0.15
−0.10
−0.05
0.00

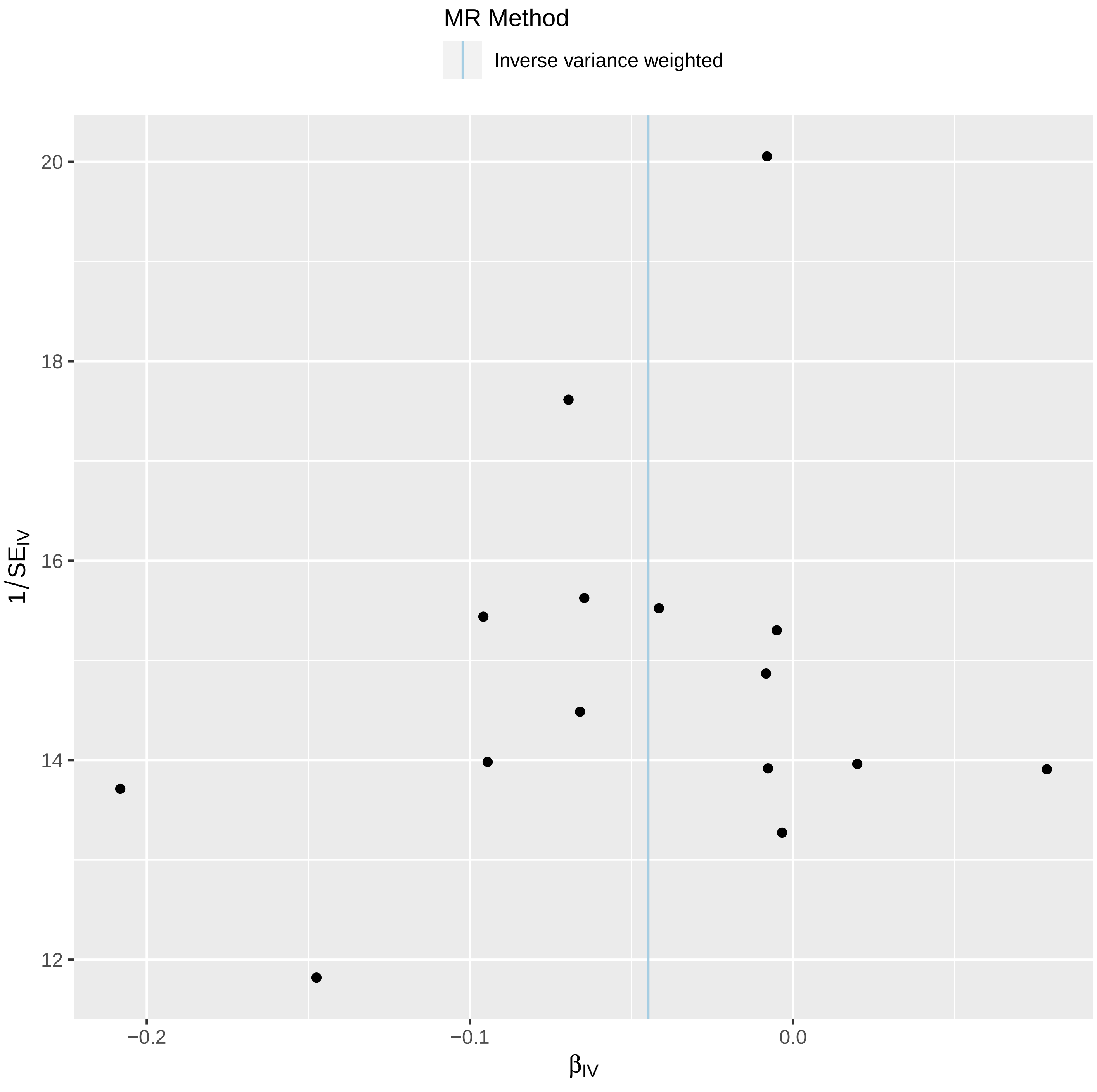
MR Method
Inverse variance weighted
1/SE_IV
20
18
16
14
12
−0.2
−0.1
0.0
β_IV

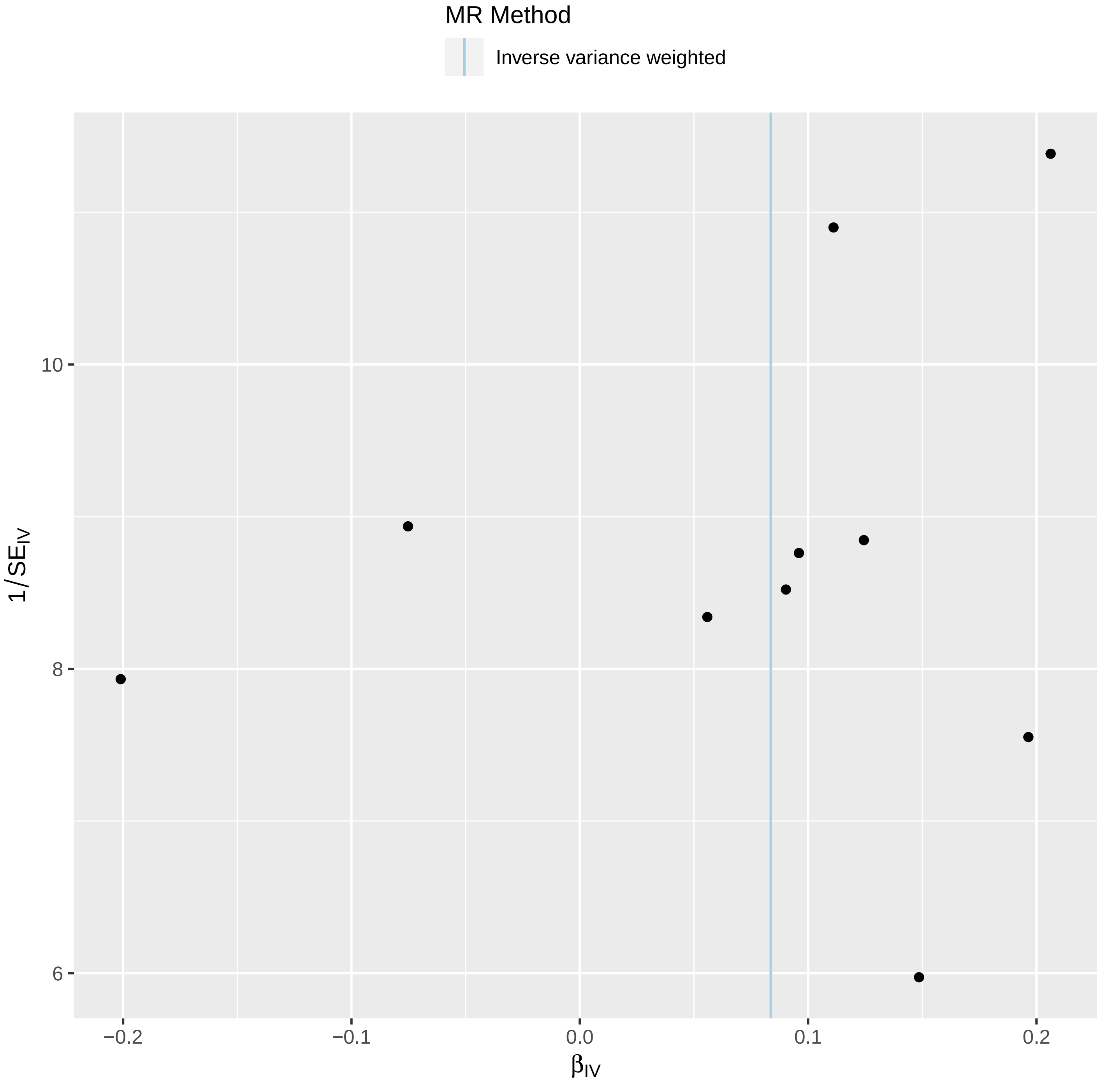
MR Method
Inverse variance weighted
10
8
6
1/SE_IV
−0.2
−0.1
0.0
0.1
0.2
β_IV

85

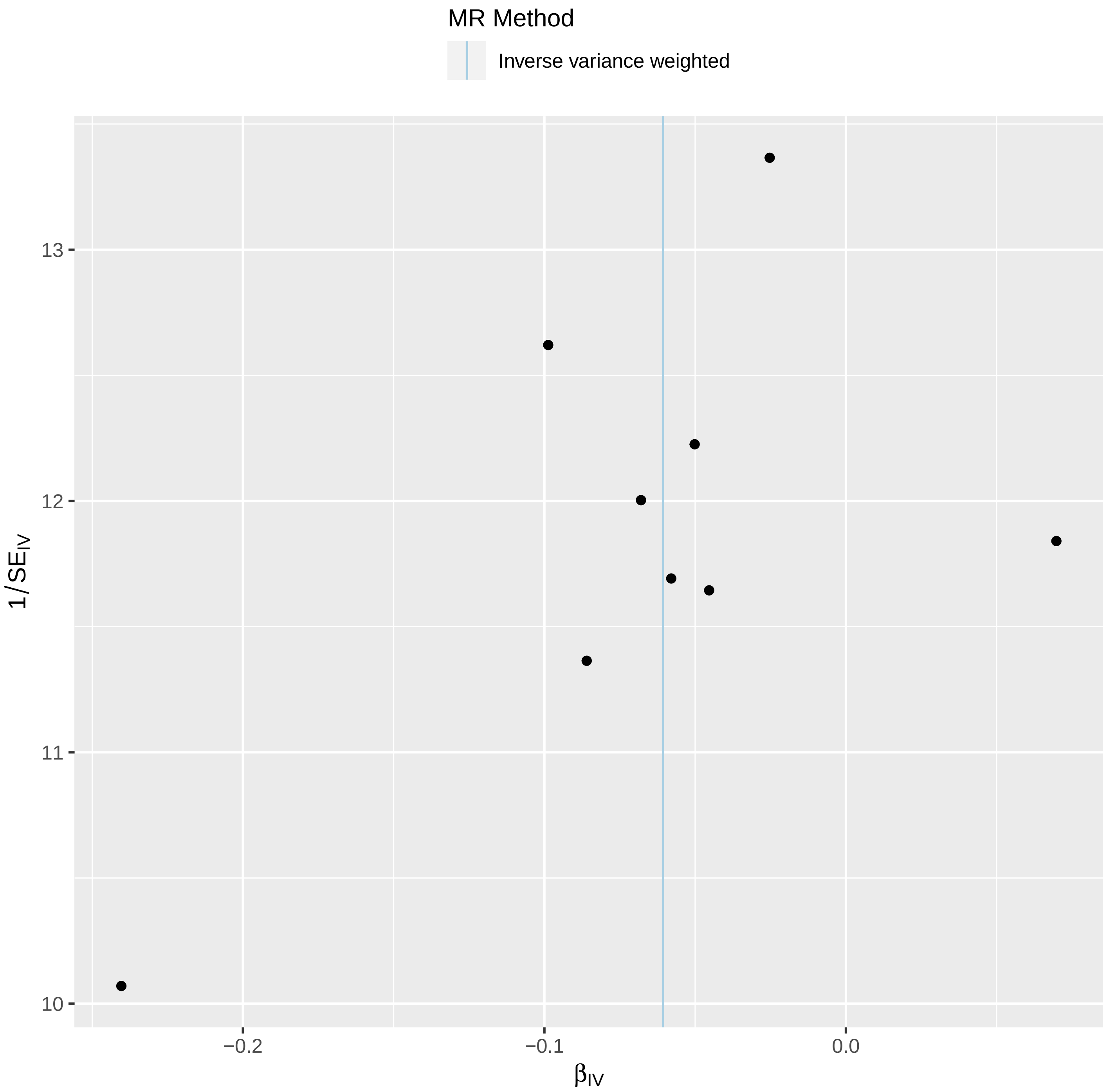

MR Method
Inverse variance weighted
13
12
11
10
1/SE$_{IV}$
−0.2
−0.1
0.0
β$_{IV}$

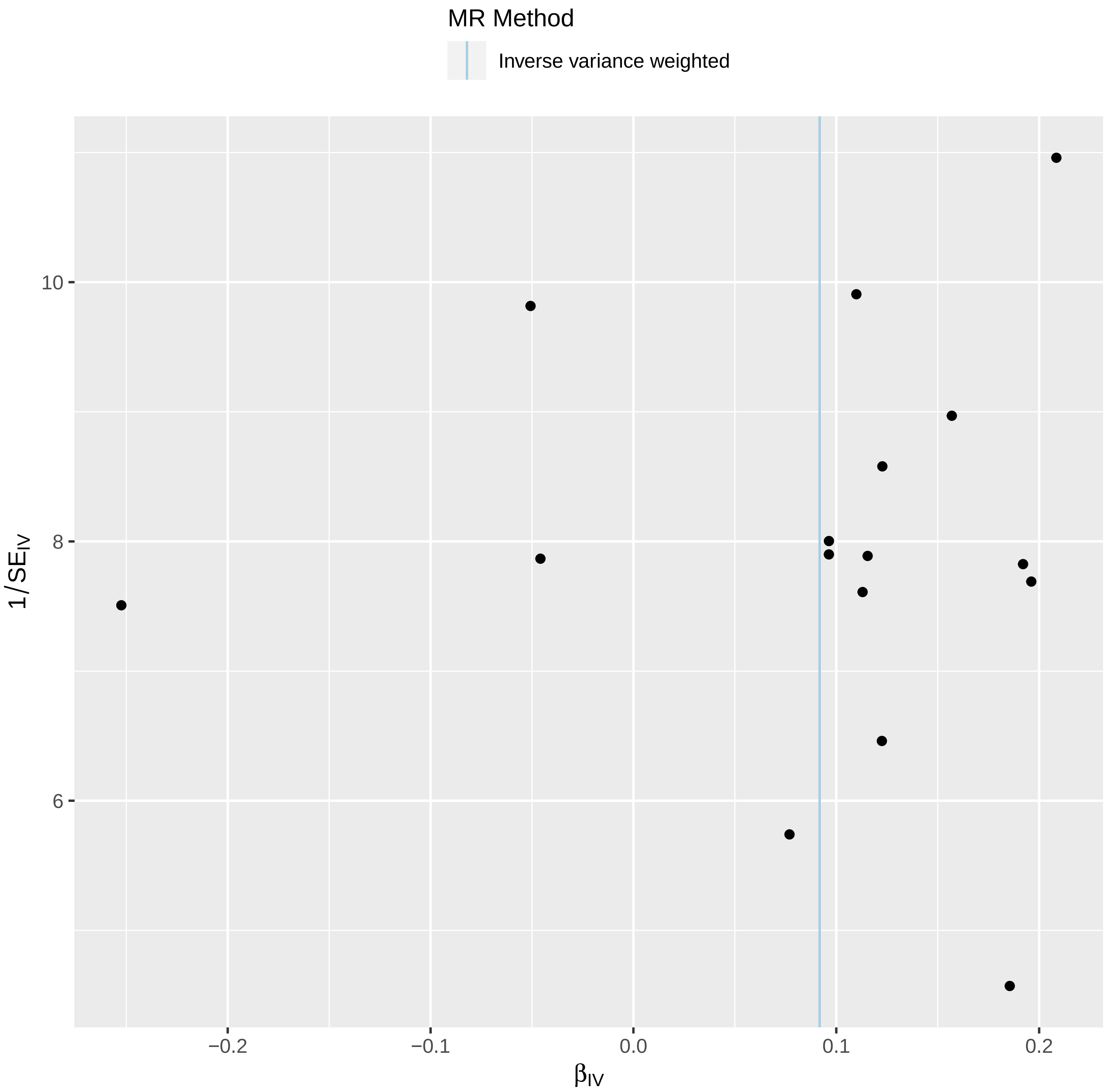
MR Method
Inverse variance weighted
10
8
6
1/SE$_{IV}$
−0.2
−0.1
0.0
0.1
0.2
β$_{IV}$

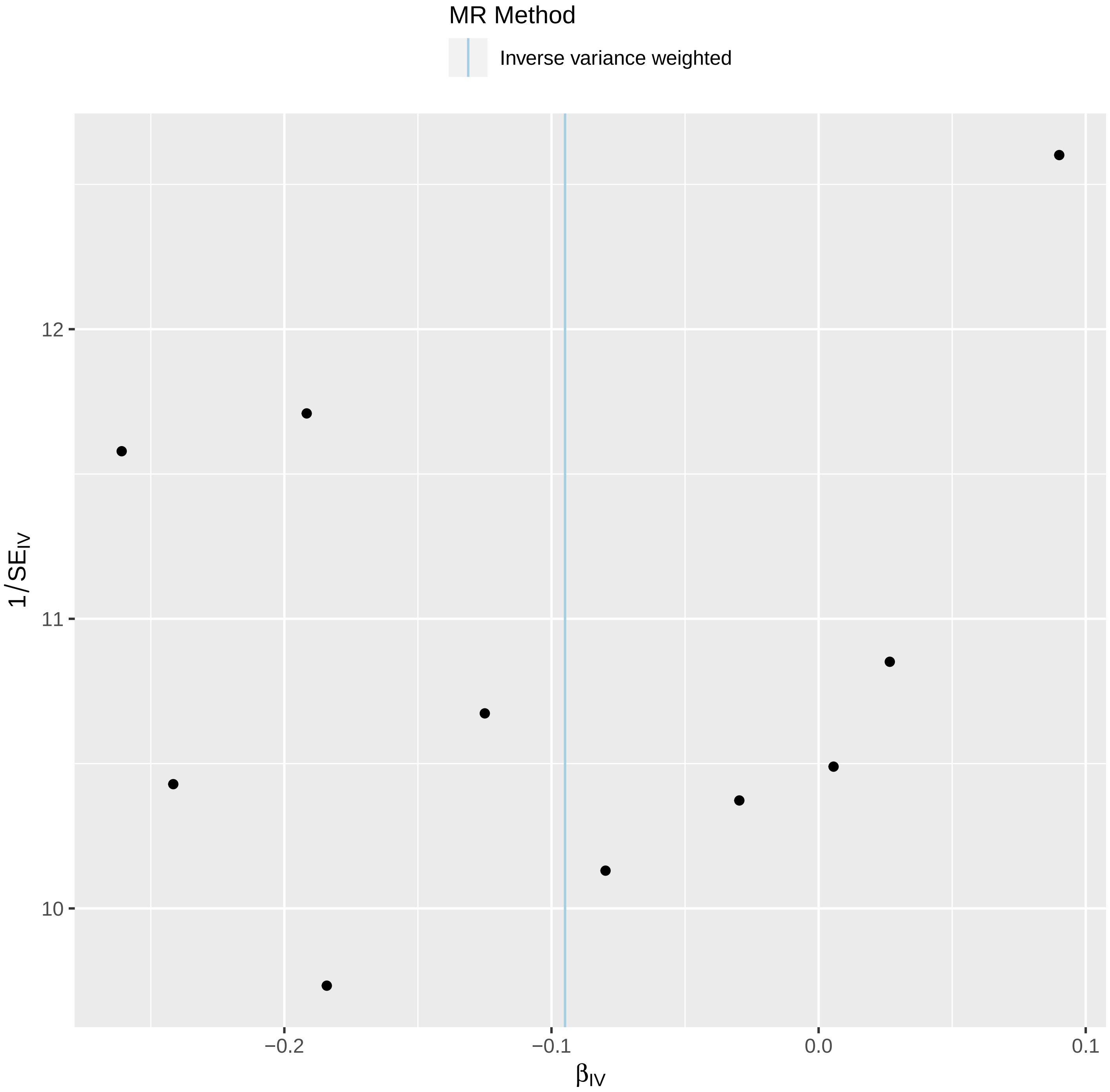
MR Method
Inverse variance weighted
12
11
10
1/SE_IV
−0.2
−0.1
0.0
0.1
β_IV

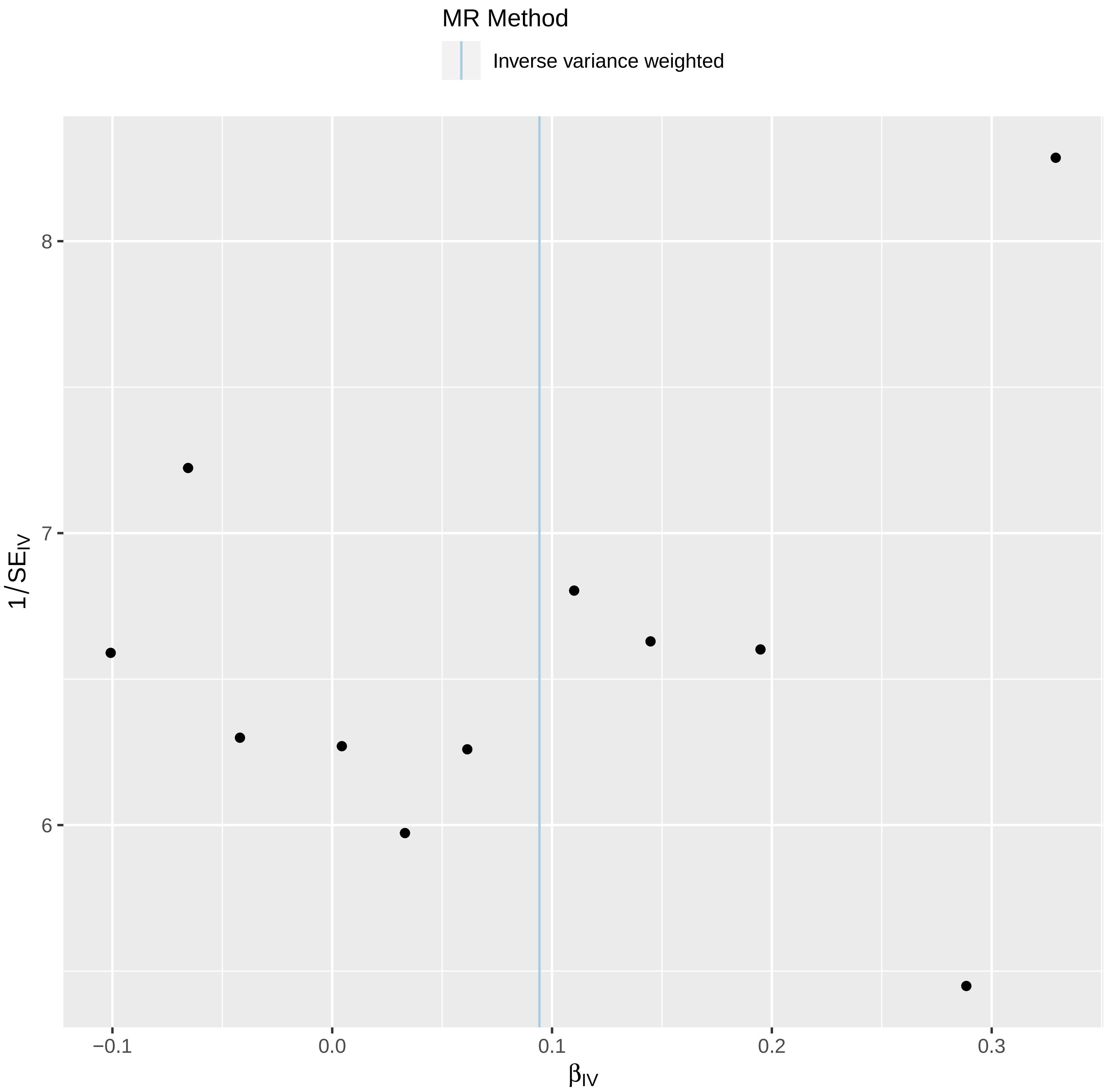
MR Method
Inverse variance weighted
8
7
6
1/SE_IV
−0.1
0.0
0.1
0.2
0.3
β_IV

89

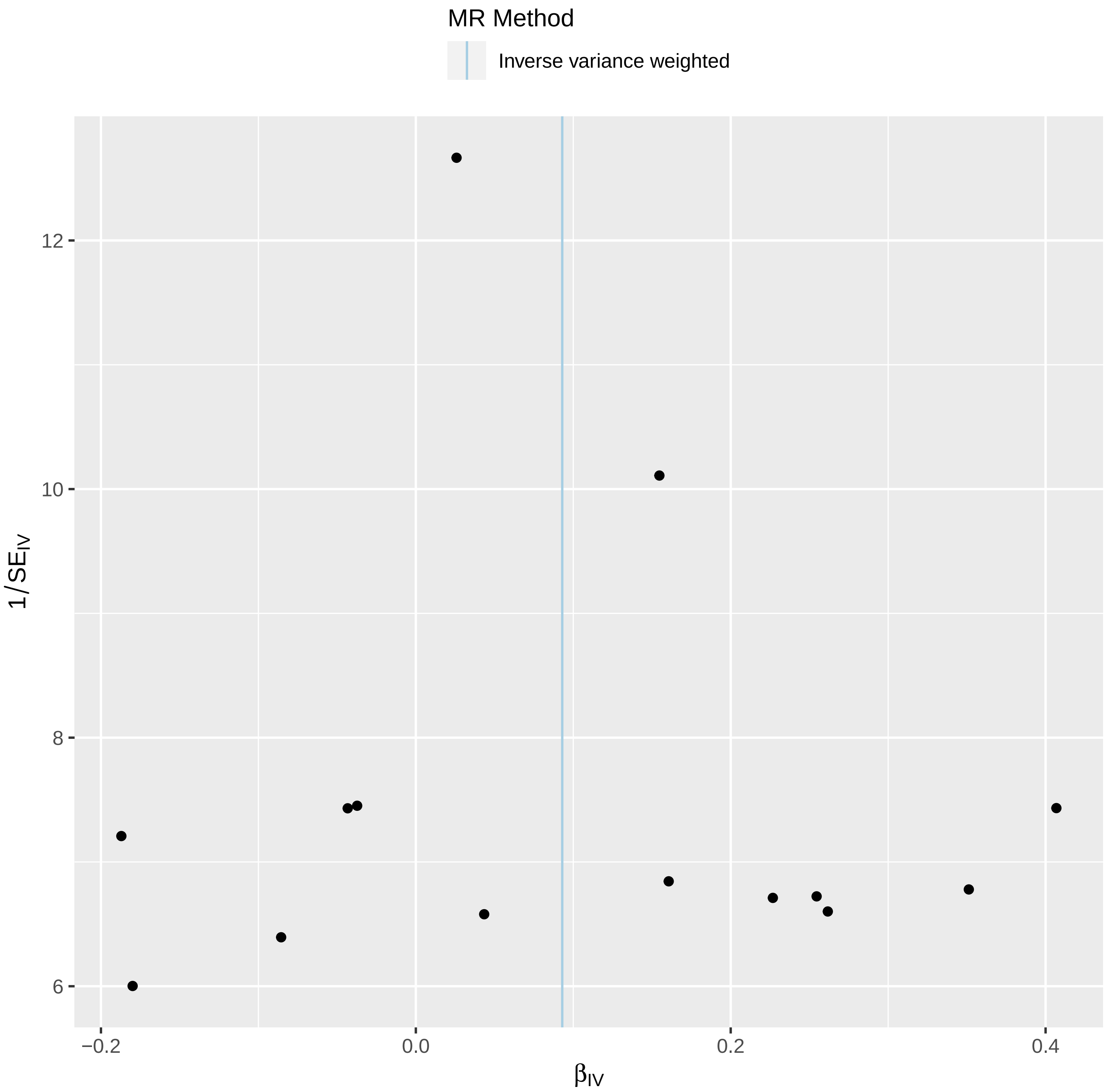
MR Method
Inverse variance weighted
12
10
8
6
1/SE$_{IV}$
−0.2
0.0
0.2
0.4
β$_{IV}$

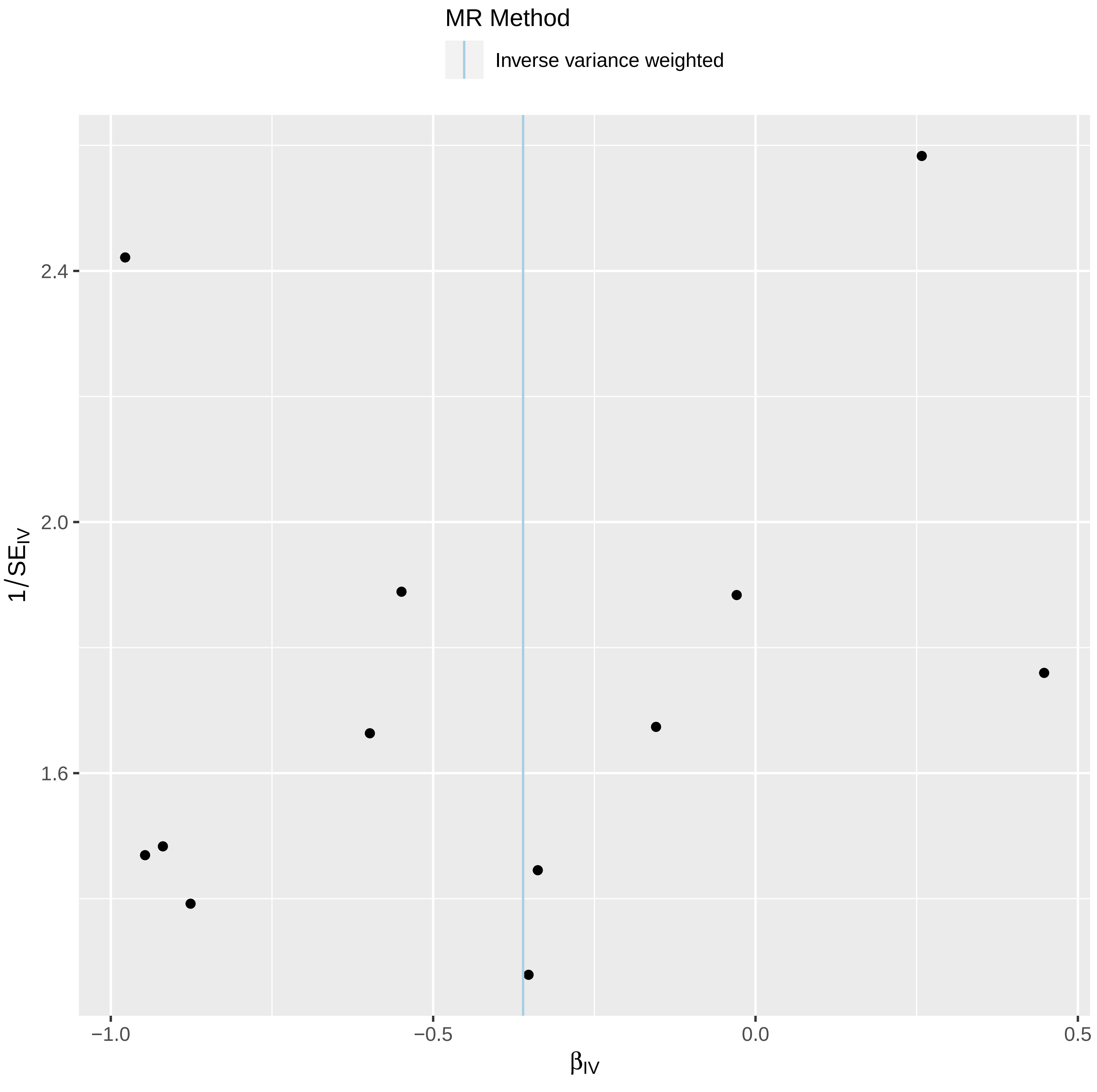
MR Method
Inverse variance weighted
2.4
2.0
1.6
1/SE$_{IV}$
−1.0
−0.5
0.0
0.5
β$_{IV}$

91

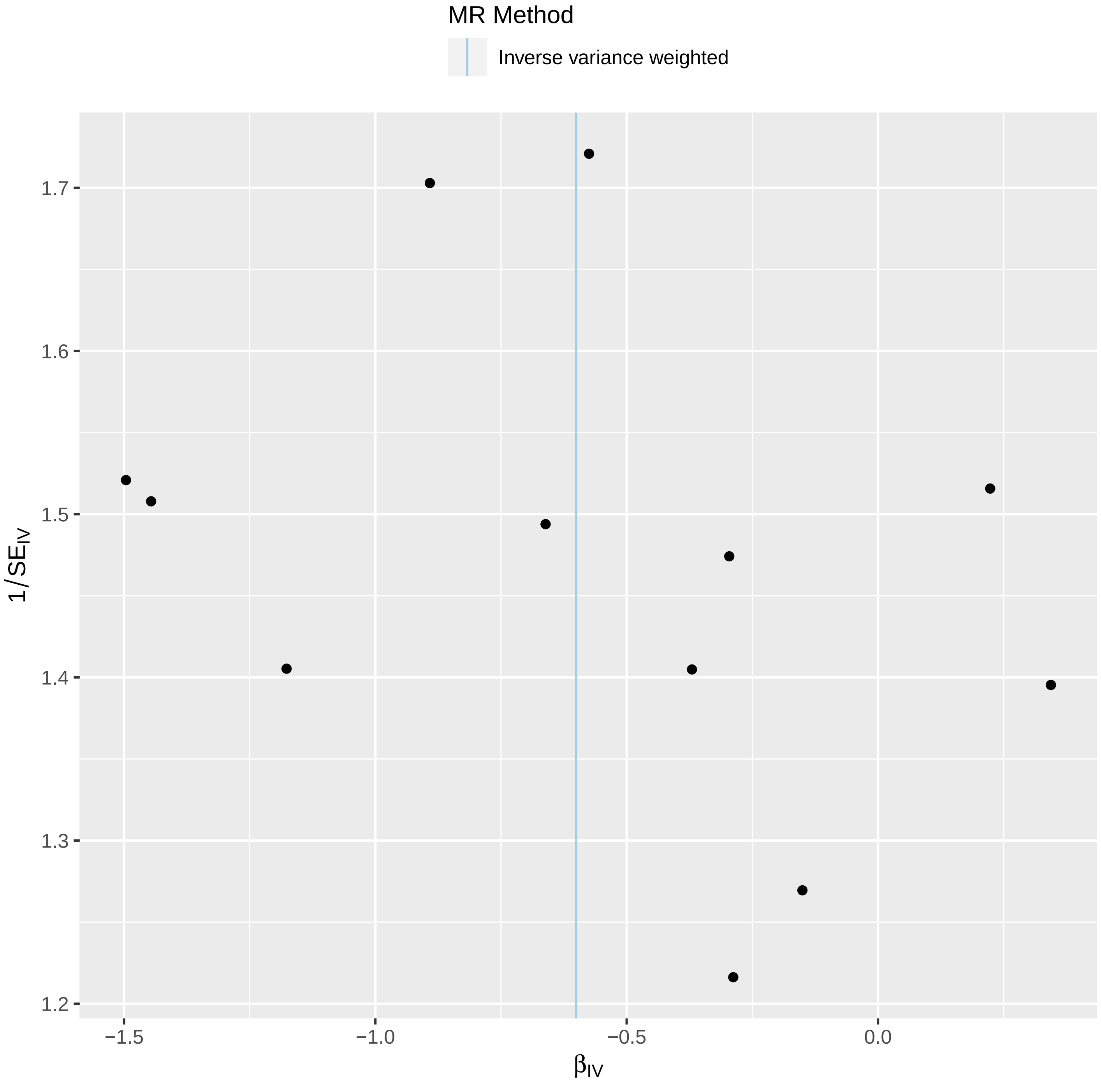
MR Method
Inverse variance weighted
1.7
1.6
1.5
1.4
1.3
1.2
1/SE_IV
−1.5
−1.0
−0.5
0.0
β_IV

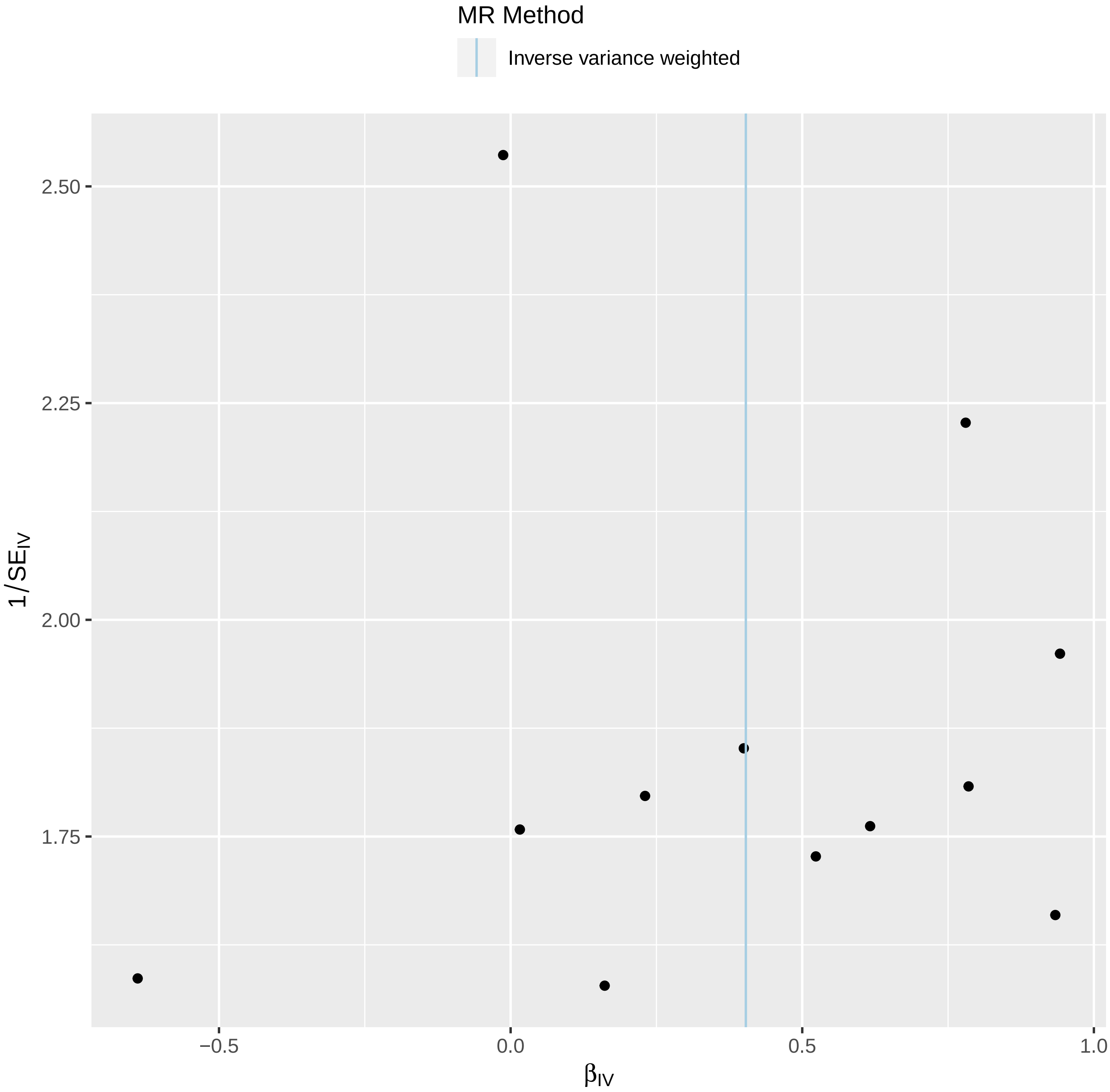
MR Method
Inverse variance weighted
2.50
2.25
2.00
1.75
1/SE$_{IV}$
−0.5
0.0
0.5
1.0
β$_{IV}$

93

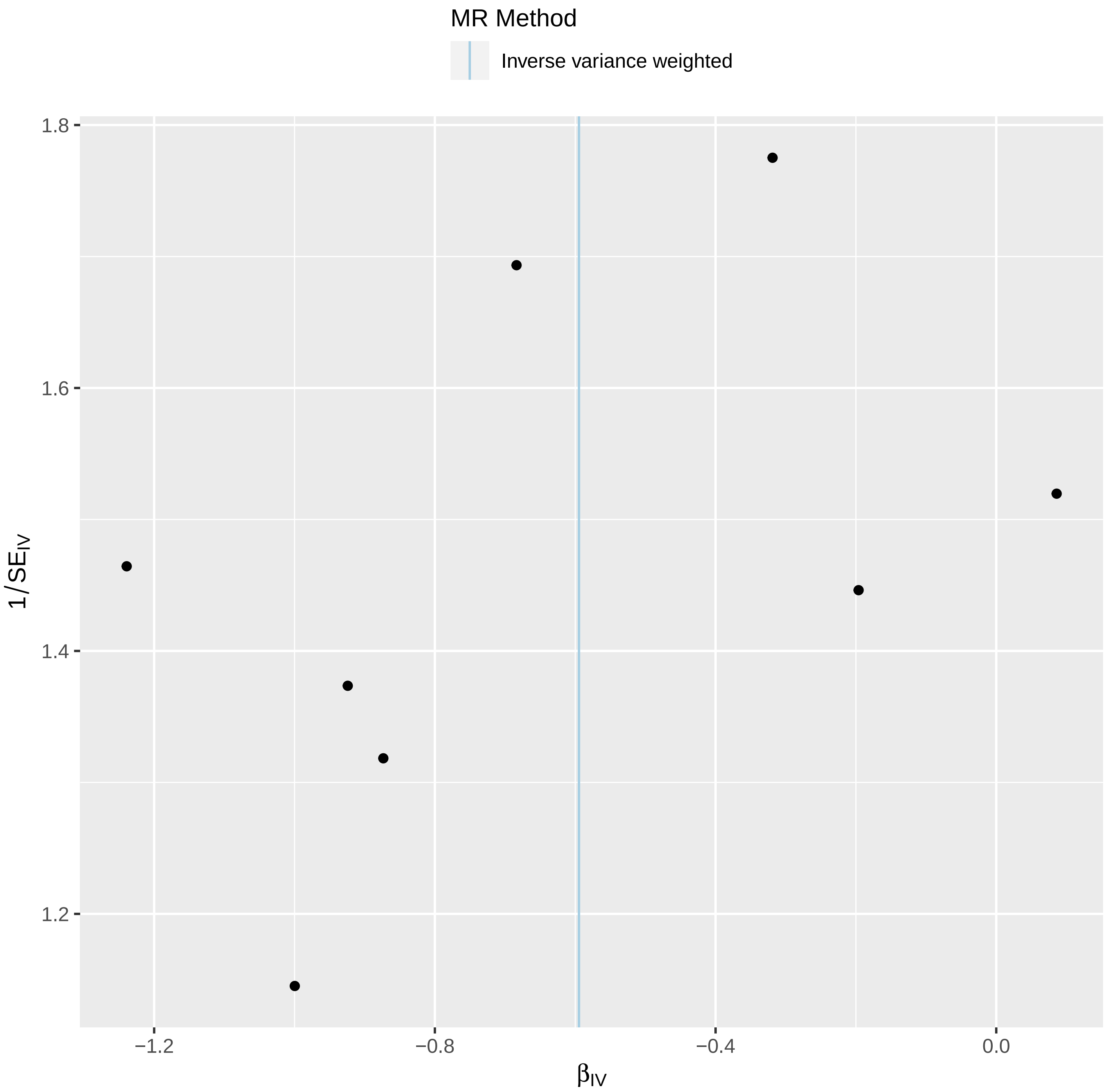

MR Method
Inverse variance weighted
1.8
1.6
1.4
1.2
1/SE$_{IV}$
−1.2
−0.8
−0.4
0.0
β$_{IV}$

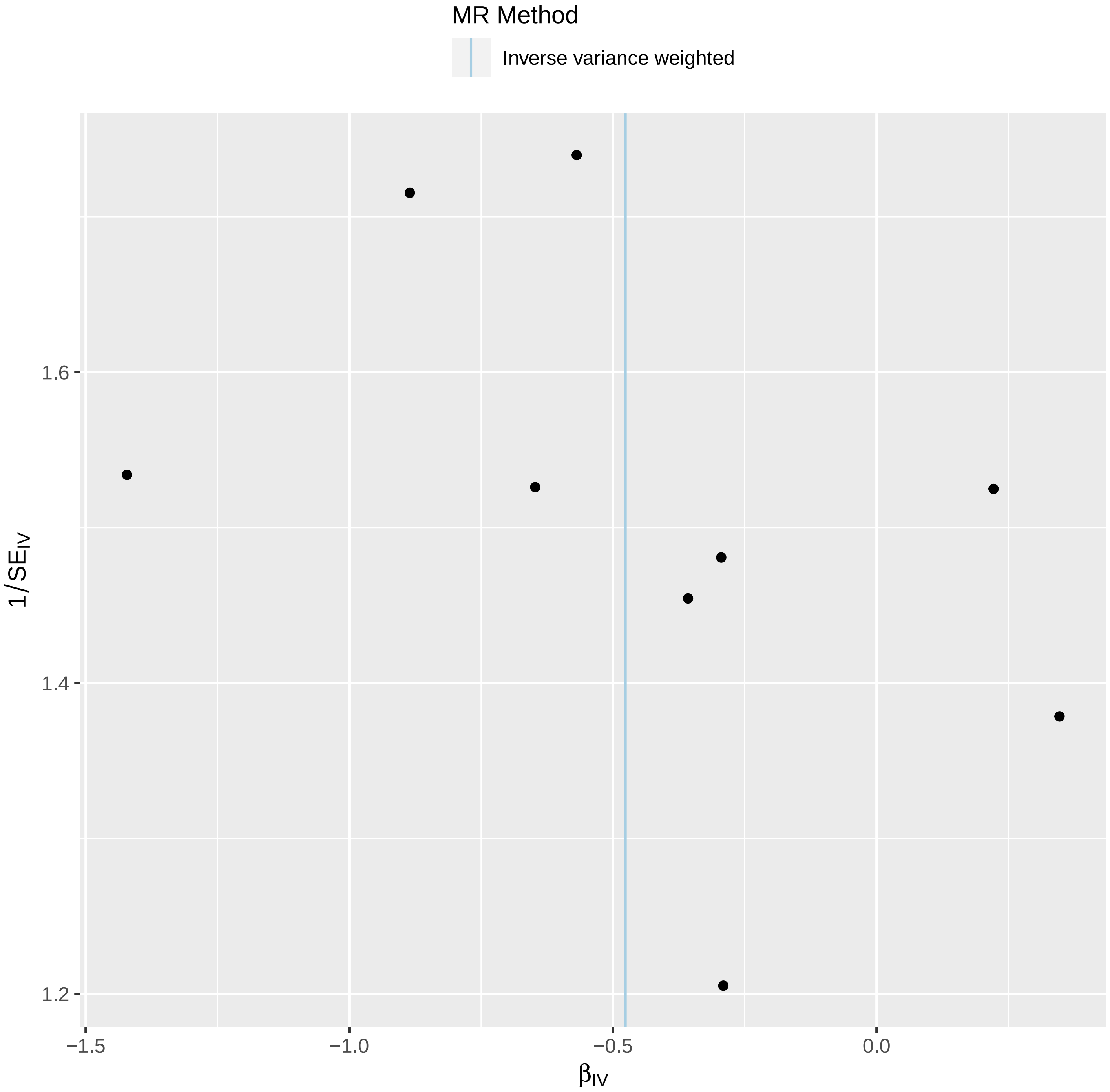
MR Method
Inverse variance weighted
1.6
1.4
1.2
$1/SE_{IV}$
−1.5
−1.0
−0.5
0.0
β_{IV}

95

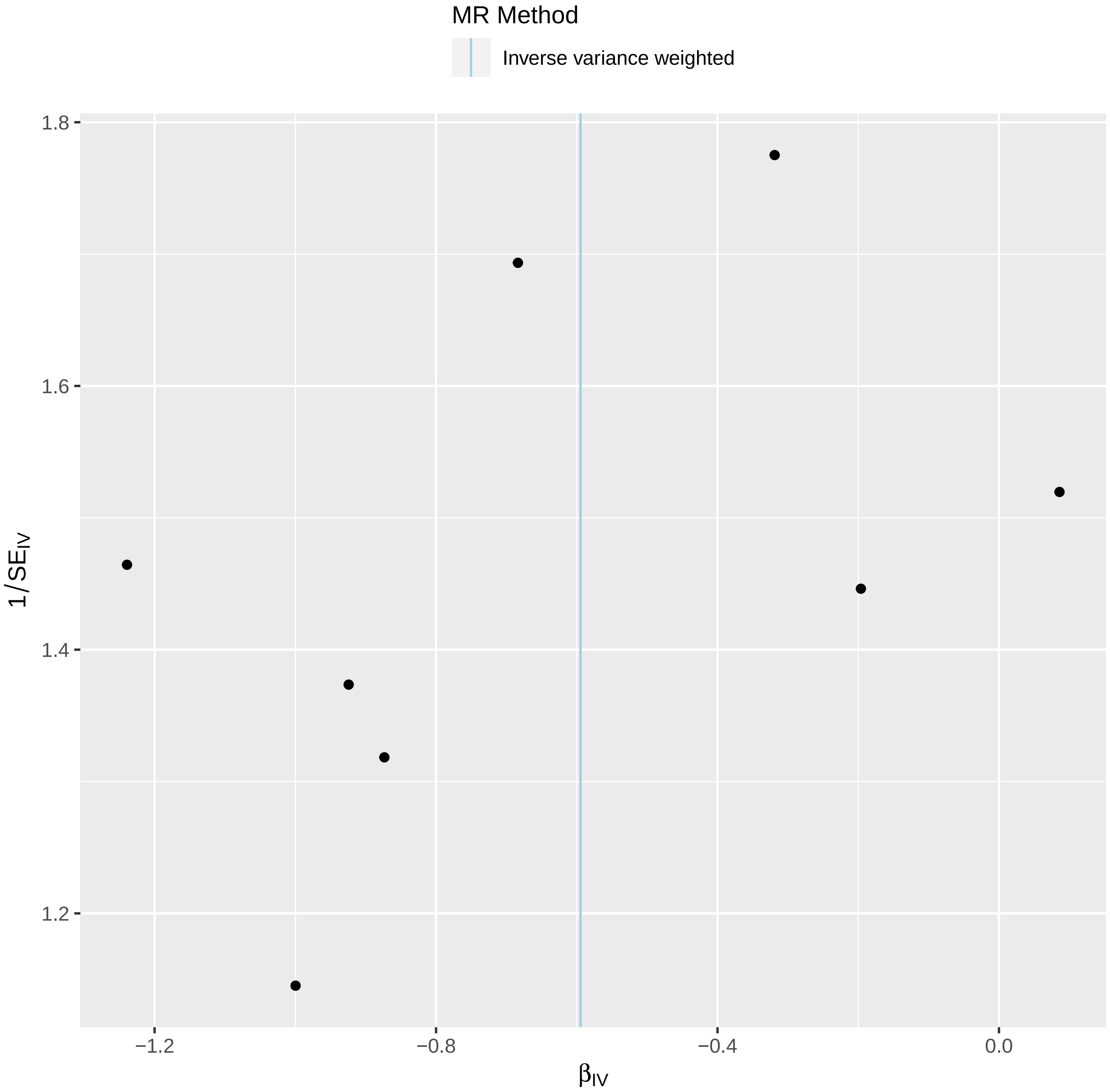
MR Method
Inverse variance weighted
1.8
1.6
1.4
1.2
1/SE$_{IV}$
−1.2
−0.8
−0.4
0.0
β$_{IV}$

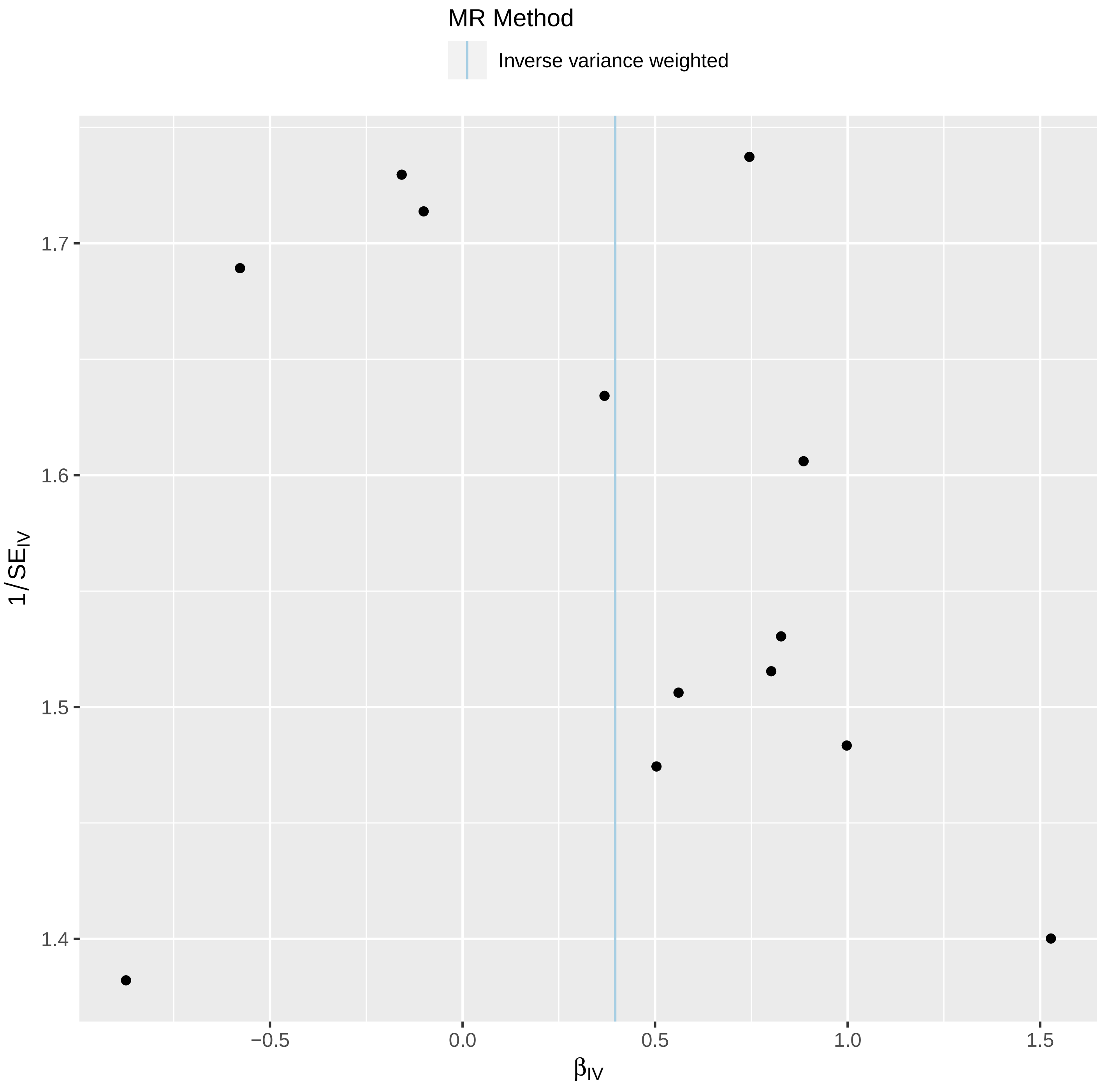
MR Method
Inverse variance weighted
1.7
1.6
1.5
1.4
−0.5
0.0
0.5
1.0
1.5
1/SE_IV
β_IV

97

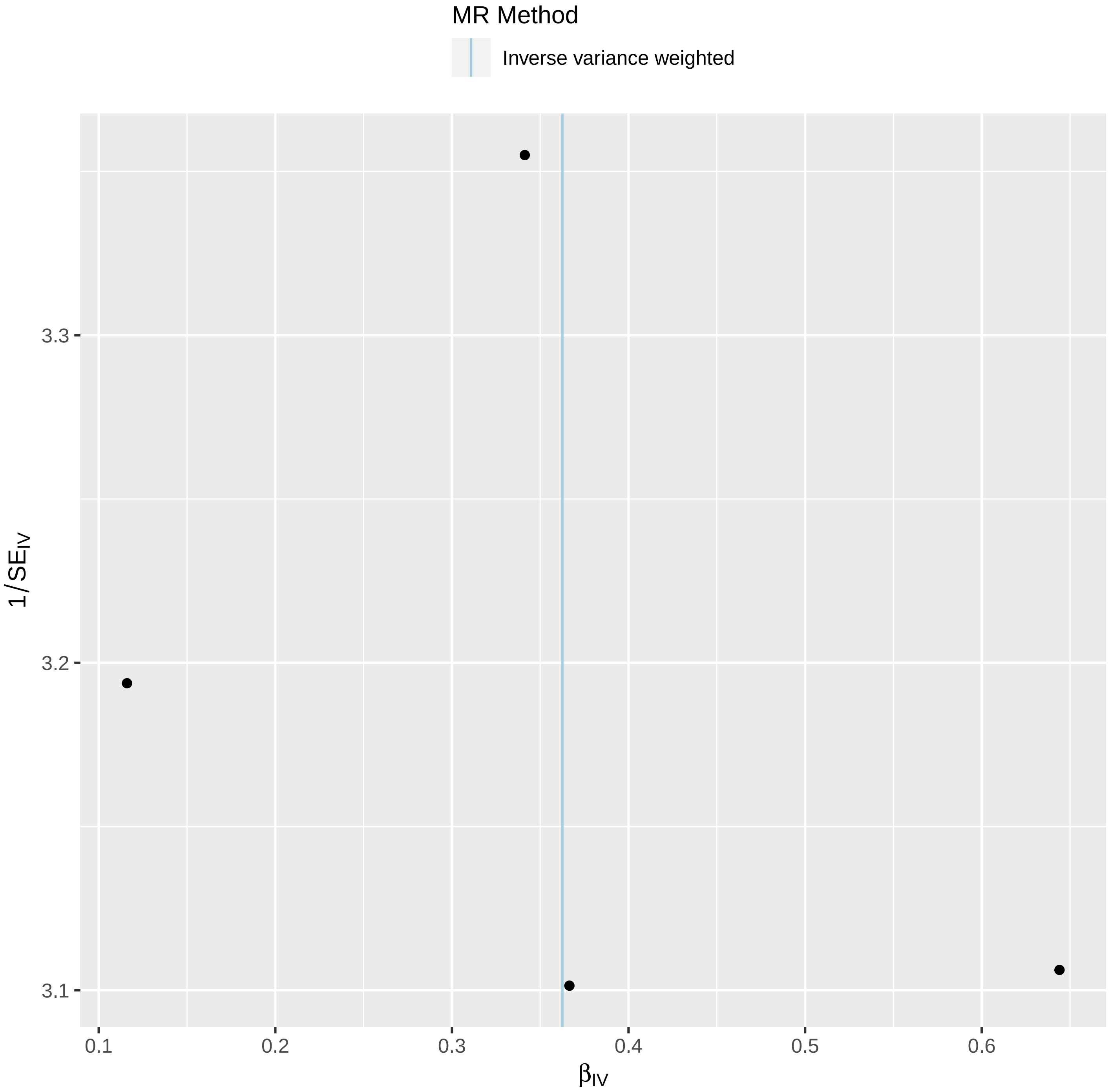

MR Method
Inverse variance weighted
3.3
3.2
3.1
1/SE$_{IV}$
0.1
0.2
0.3
0.4
0.5
0.6
β$_{IV}$

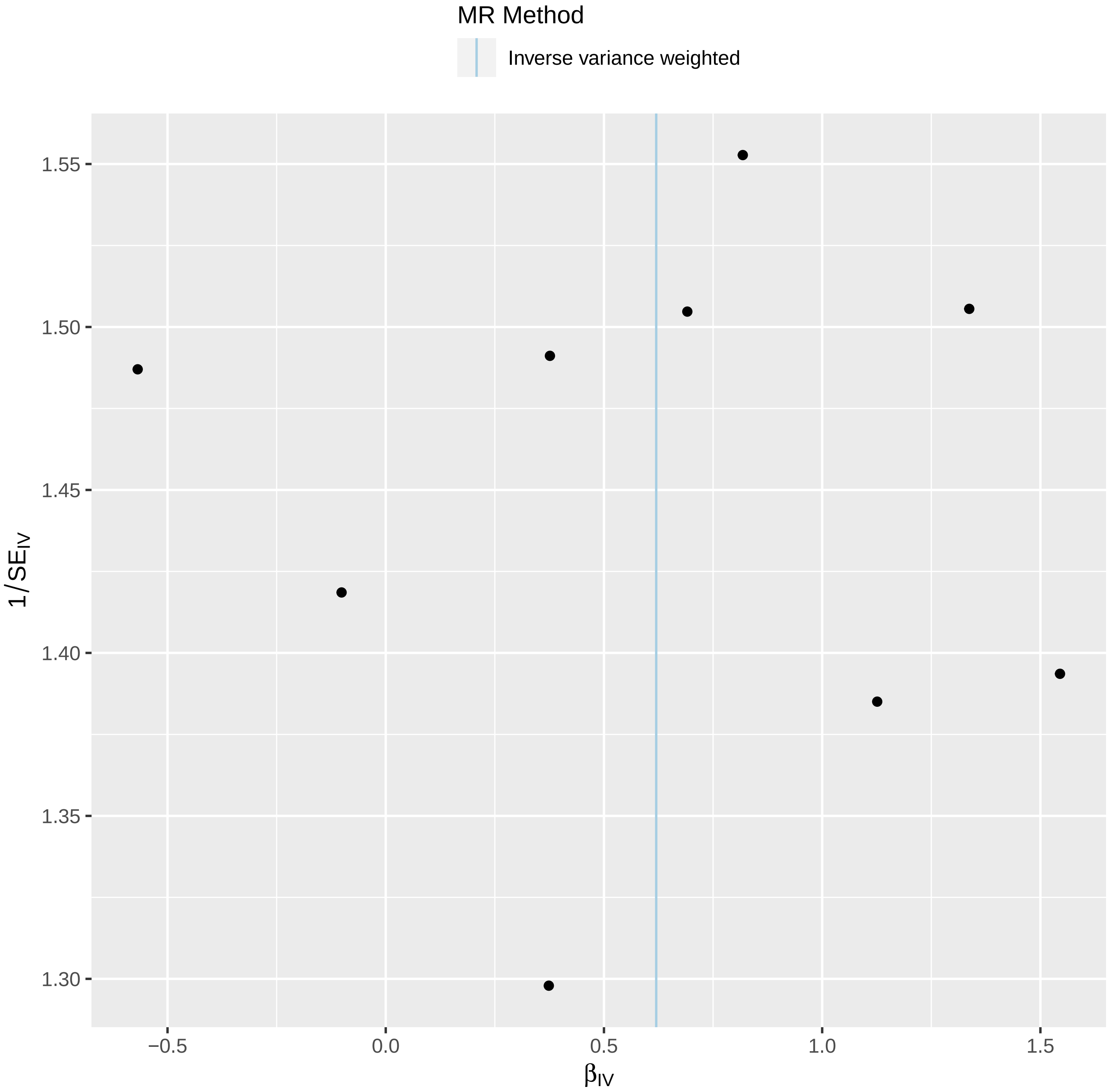
MR Method
Inverse variance weighted
1.55
1.50
1.45
1.40
1.35
1.30
1/SE_IV
−0.5
0.0
0.5
1.0
1.5
β_IV

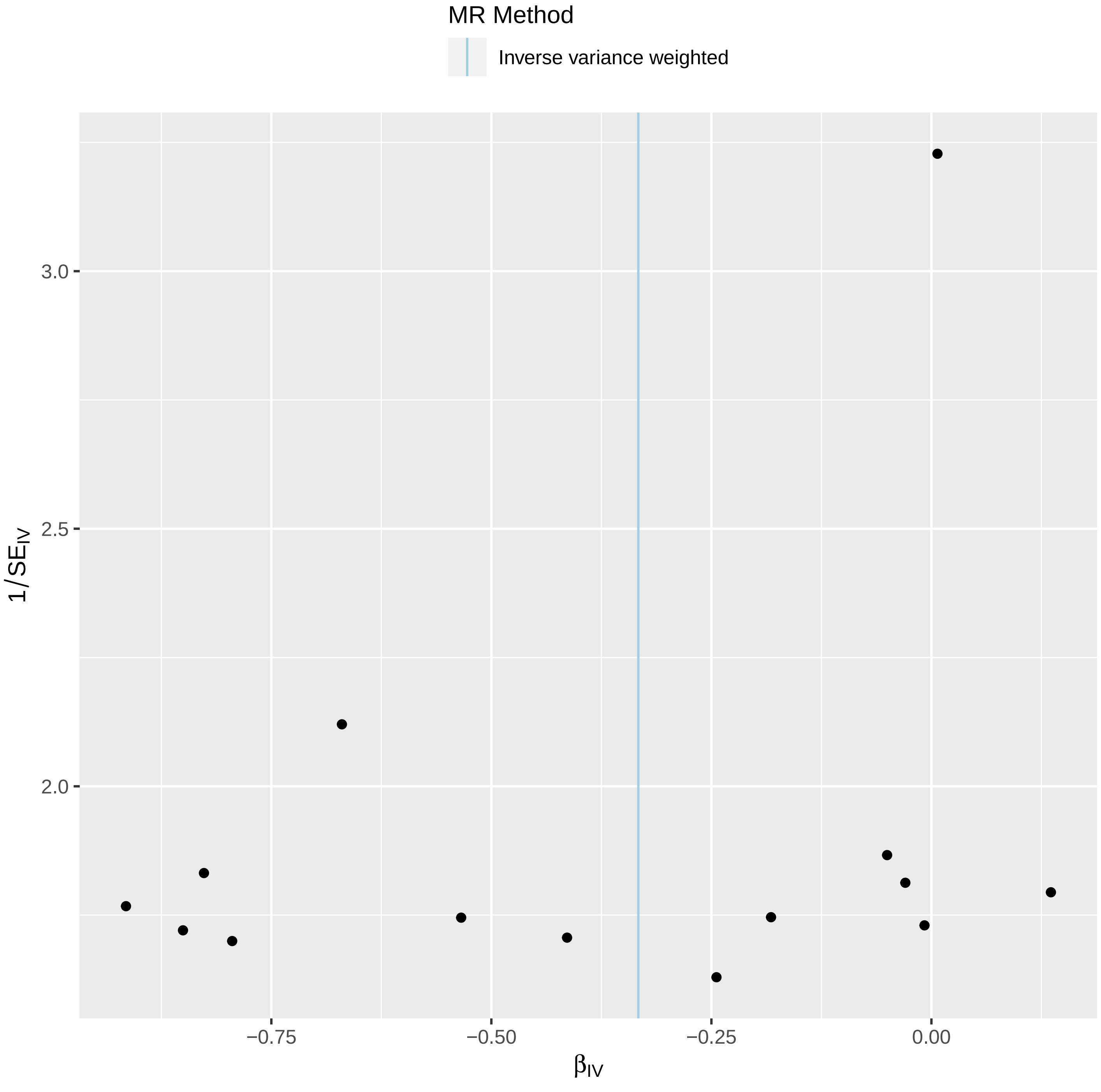
MR Method
Inverse variance weighted
3.0
2.5
2.0
1/SE$_{IV}$
−0.75
−0.50
−0.25
0.00
β_{IV}

100

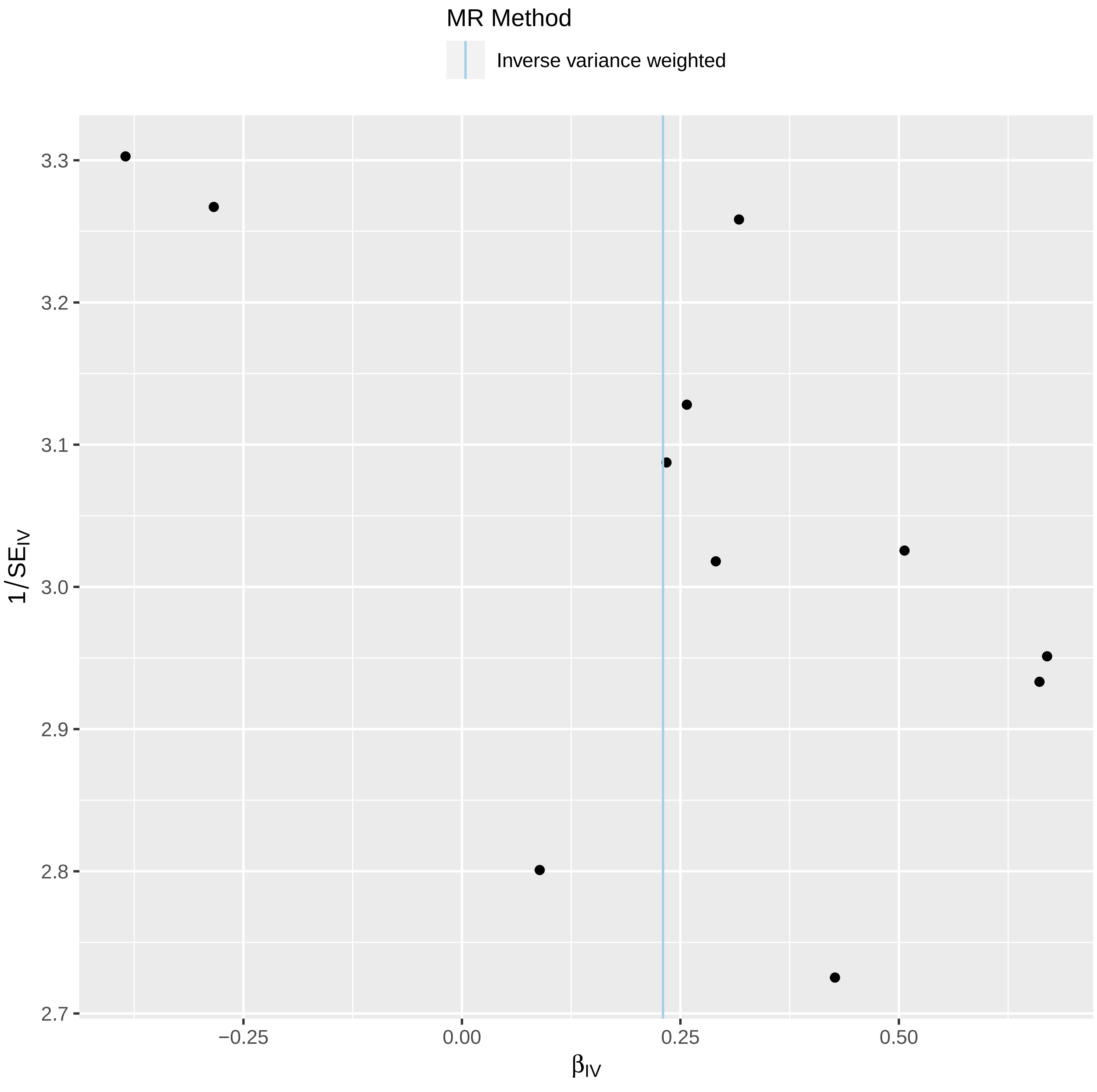

MR Method
Inverse variance weighted
3.3
3.2
3.1
3.0
2.9
2.8
2.7
1/SE$_{IV}$
−0.25
0.00
0.25
0.50
β$_{IV}$

101

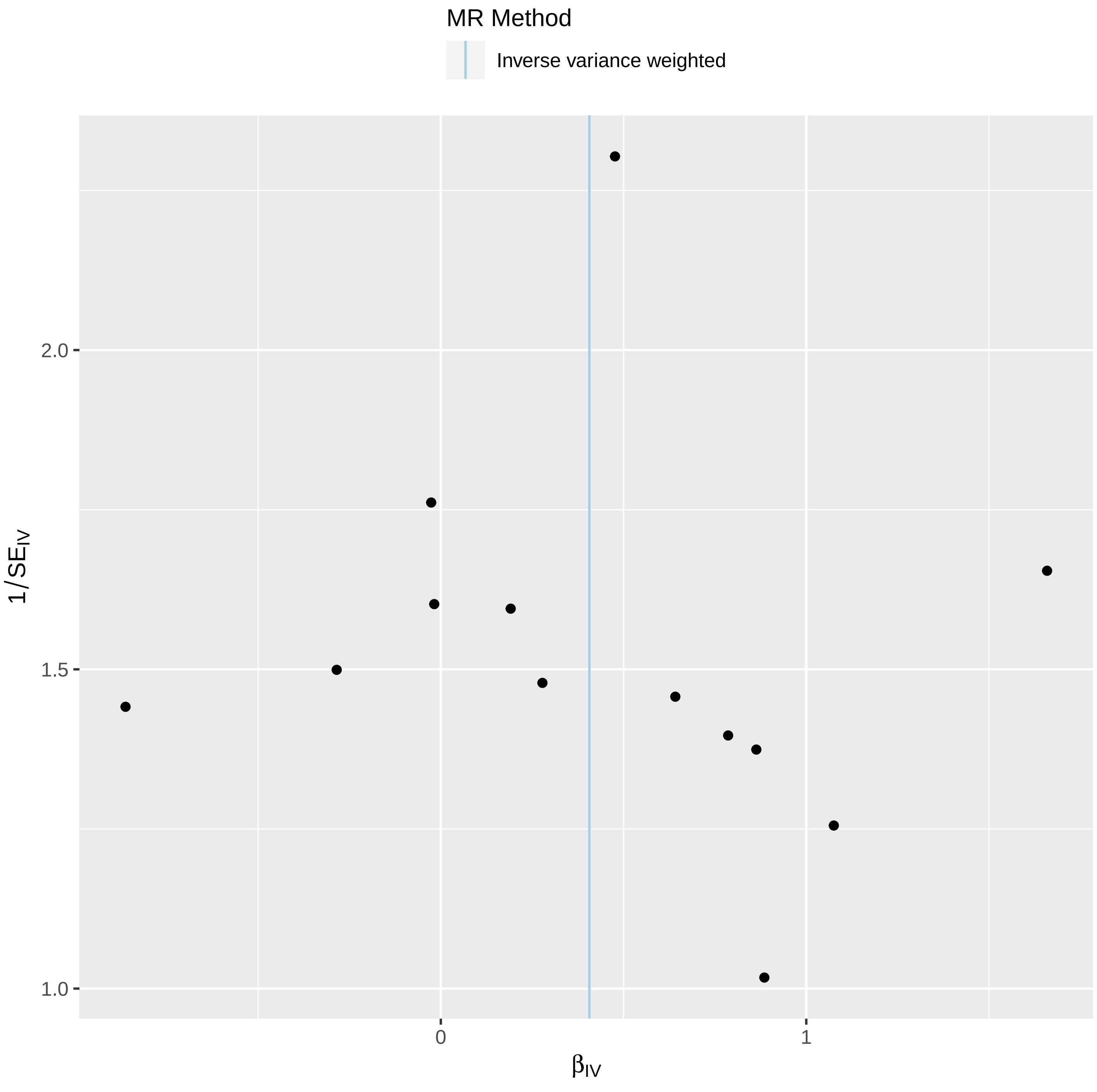

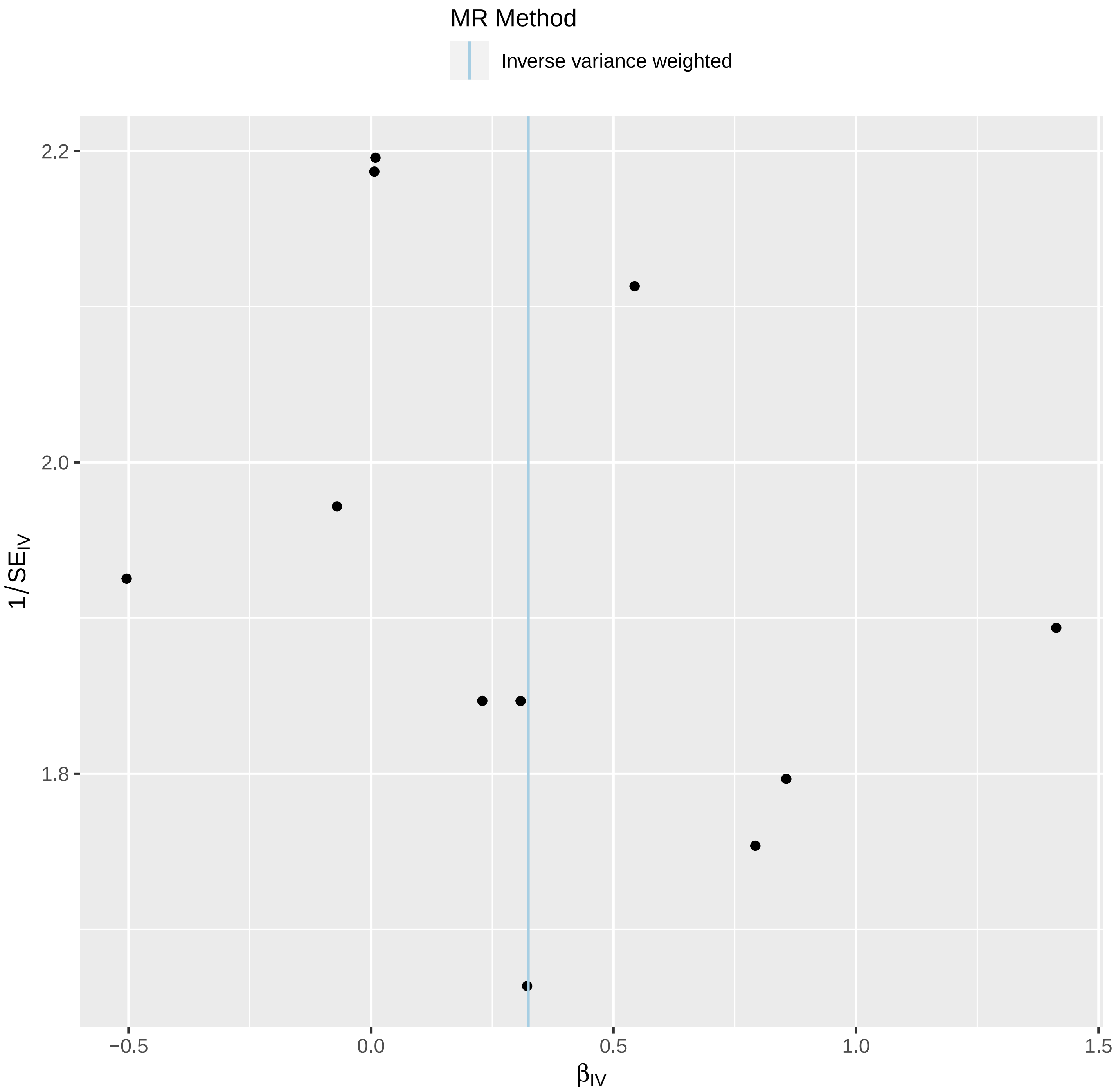
MR Method
Inverse variance weighted
2.2
2.0
1.8
1/SE_IV
−0.5
0.0
0.5
1.0
1.5
β_IV
